# Comparative analyses of the Conserved Oligomeric Golgi (COG) complex in vertebrates

**DOI:** 10.1186/1471-2148-10-212

**Published:** 2010-07-15

**Authors:** Rita Quental, Luísa Azevedo, Rune Matthiesen, António Amorim

**Affiliations:** 1 IPATIMUP -Institute of Molecular Pathology and Immunology of the University of Porto, Porto, Portugal; 2 Faculty of Sciences, University of Porto, Porto, Portugal

## Abstract

**Background:**

The Conserved Oligomeric Golgi (COG) complex is an eight-subunit assembly that localizes peripherally to Golgi membranes and is involved in retrograde vesicular trafficking. COG subunits are organized in two heterotrimeric groups, Cog2, -3, -4 and Cog5, -6, -7, linked by a dimeric group formed by Cog1 and Cog8. Dysfunction of COG complex in humans has been associated with new forms of Congenital Disorders of Glycosylation (CDG), therefore highlighting its essential role. In the present study, we intended to gain further insights into the evolution of COG subunits in vertebrates, using comparative analyses of all eight COG proteins.

**Results:**

We used protein distances and *d*_N_/*d*_S _ratios as a measure of the rate of proteins evolution. The results showed that all COG subunits are evolving under strong purifying selection, although COG1 seems to evolve faster than the remaining proteins. In addition, we also tested the expression of *COG *genes in 20 human tissues, and demonstrate their ubiquitous nature.

**Conclusions:**

COG complex has a critical role in Golgi structure and function, which, in turn, is involved in protein sorting and glycosylation. The results of this study suggest that COG subunits are evolutionary constrained to maintain the interactions between each other, as well with other partners involved in vesicular trafficking, in order to preserve both the integrity and function of the complex.

## Background

Most cellular processes are carried out by multiprotein complexes that constitute important functional units in the cell [1]. This fact has motivated a number of studies aiming to investigate the structure, function and evolution of such multisubunit molecular machines [e.g., [1-4]].

A cellular process in which protein complexes are known to be involved is the transport of proteins between cellular compartments (vesicular trafficking) [5,6]. Proteins synthesised in the secretory pathway are transported inside vesicles that move from the endoplasmic reticulum to the Golgi apparatus, from where polypeptides are then sorted to several cellular compartments [7]. As progression through the Golgi occurs, proteins may undergo modifications like glycosylation, a necessary step for their stability and function [8]. Several large protein complexes play an important role in the fidelity of vesicle fusion, acting as tethering factors through the formation of physical links between membranes prior to fusion [5,6,9]. One of these is the Conserved Oligomeric Golgi (COG) complex [10], which localizes at the cytoplasmic surface of the Golgi apparatus [11-14].

Several studies have been performed demonstrating the involvement of COG in retrograde vesicular trafficking of Golgi resident proteins [15-18], including enzymes that participate in glycans biosyntesis [19,20]. Consequently, COG impairment results in abnormal Golgi morphology (dilated cisternae and accumulation of vesicles [10,17,21,22]) and function (glycosylation defects [reviewed in [23] and references therein]). However, its precise mechanism of action is not completely understood.

**Figure 1:**
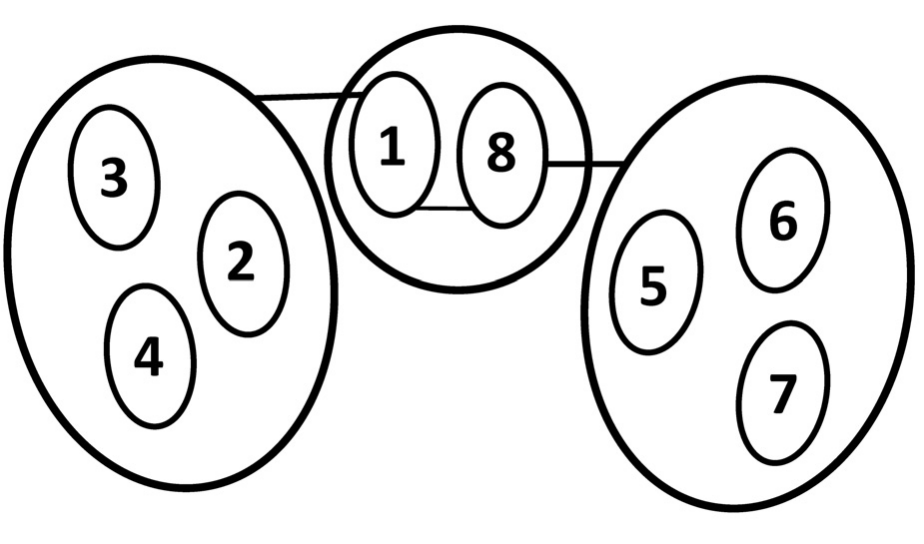
**Schematic architectural representation of mammalian COG complex**. COG is organized in two heterotrimeric subcomplexes (Cog2 to Cog4 and Cog5 to Cog7), which are connected by a heterodimer (Cog1 and Cog8). Numbers represent COG subunits. Adapted from [29].

COG complex is composed by eight distinct subunits [10,12,13,24-26], Cog1 to Cog8, arranged in two lobes consisting of Cog1 to Cog4 (lobe A) and Cog5 to Cog8 (lobe B) [10]. Although several models have been advanced refining the architecture of COG [22,27-29], the most recent studies converge in suggesting that mammalian COG members are organized in two heterotrimeric groups, Cog2-Cog3-Cog4 and Cog5-Cog6-Cog7, which are linked by the dimeric group formed by Cog1 and Cog8. In particular, Cog1 associates with Cog2-Cog3-Cog4, whereas Cog8 interacts with Cog5-Cog6-Cog7 (Figure 1) [22,29].

Previous studies have shown the possibility that COG components have different roles within the complex, since mutations or deletion of individual subunits cause sharply distinct phenotypes [25,26]. In yeast, the deletion of any one of the four lobe A subunits causes a severe growth defect, whilst disruption of the remaining genes (*COG5 *to *COG8*) does not substantially interfere with normal cell growth [25]. In mammals, COG1- and COG2-deficient cells present several dilated cisternae [10] and pleiotropic defects in the synthesis of *N*-, *O*- and lipid-linked glycans [8]. A similar phenotype is observed in COG5-deficient cells, although the alterations in glycosylation are subtle [22]. In addition, COG3 depletion entails the accumulation of vesicles distributed throughout the cytoplasm [17]. More recently, COG dysfunction caused by mutations in specific subunits has been associated with new forms of Congenital Disorders of Glycosylation (CDG) in humans [reviewed in [30,31]].

In the present study, we used a broad range of comparative sequence analyses to track the evolutionary profile of this complex in vertebrates.

## Methods

### *In silico *orthologs retrieval

Human protein sequences corresponding to each COG subunit (COG1-COG8) were retrieved from the National Center for Biotechnology Information (NCBI) [32]. These sequences were used as queries to search orthologous proteins from the reference proteins database (RefSeq) using the BLASTP algorithm [33], with an E-value cutoff of 10^-3 ^and using the reciprocal best-hit approach [34]. Alternatively, sequences were retrieved from Ensembl genome browser [35], release 57 from March 2010, via the *orthogues *option. Available sequences from the following taxa were considered in this study: mammals (*Homo sapiens*, *Pan troglodytes*, *Pongo pygmaeus, Macaca mulatta, Callithrix jacchus, Mus musculus*, *Rattus norvegicus*, *Oryctolagus cuniculus, Equus caballus, Canis familiaris, Bos taurus *and *Monodelphis domestica*), birds (*Gallus gallus *and *Taeniopygia guttata*), reptiles (*Anolis carolinensis*), amphibians (*Xenopus tropicalis*) and fishes (*Danio rerio, Takifugu rubripes, Gasterosteus aculeatus, Tetraodon nigroviridis *and *Oryzias latipes*). COG3 from *G. aculeatus*, COG5 from *X. tropicalis *and COG6 from *D. rerio *were assembled based on the genomic sequence through the comparison with the orthologous protein from other species, and through TBLASTN searches on ESTs database. Accession numbers are available in the additional file 1. In addition, due to probable assembling errors, residues 82 to 124 from *P. troglodytes *COG4, residues 57 to 146 and 436 to 470 from *P. pygmaeus *COG7 as well as residues 493 to 522 from *M. mulatta *COG7 were replaced by missing data.

### Protein Distances

Protein sequences were aligned with MUSCLE [36] and manually refined by removing sites at which all sequences except one or two have alignment gaps. Additionally, the initial and terminal regions of the multiple sequences alignment had, in some cases, to be removed because they were poorly aligned. This step was particularly important in COG5 and COG8. The final alignments of each group of orthologous sequences are available at additional file 2. Protein distances were calculated using Protdist from PHYLIP 3.69 package [37], with the Jones-Taylor-Thornton (JTT) evolutionary model and gamma distribution of rates with a fixed shape parameter of 1. Divergence times between species were obtained with TimeTree [38,39].

### Phylogenetic analysis and *d*_N_/*d*_S _(ω) ratios

To reconstruct species phylogeny, the protein-coding sequences were aligned with ClustalW program [40] implemented in Bioedit version 7.0.9.0 [41], using protein alignments as template to avoid out-of-frame gaps. The poorly aligned positions and divergent regions in each alignment were eliminated with GBlocks program [42] and the resulting blocks were concatenated in a single alignment of 15195 positions. To identify the model of nucleotide substitution that best fits the data the Akaike Information Criterion (AIC) was applied, using the jModelTest 0.1.1 [43]. The selected model (GTR +I +G) was used to reconstruct the maximum likelihood phylogeny in Phyml 3.0 [44]. The tree was drawn with FigTree program [45]. The resulting tree topology, but not branch lengths, was used to fit different models in PAML.

The number of synonymous substitutions per synonymous site (*d*_S_) and the number of nonsynonymous substitutions per nonsynonymous sites (*d*_N_) have been estimated with CODEML from the PAML v.4.4 package [46], using the F3 × 4 codon frequency model and treating alignment gaps as ambiguity characters (*cleandata = 0*). The input alignments were modified by removing positions that showed evidence to represent true indels, while keeping those that appear to be missing data. Alignments are available in additional file 3. Several models that allow for different levels of heterogeneity in the *d*_N_/*d*_S _ratio (ω) among lineages have been applied: the one-ratio model that assumes the same ω ratio for all branches in the phylogeny; the free-ratios model that allows ω to vary on every lineage; and the two-ratio model, which assumes that the branch of interest has an ω value (ω_1_) different from the ratio of the other lineages (ω_0_- background ratio). The above models can be compared using a likelihood ratio test (LRT) to test different hypothesis, as described by Yang [47]. Because synonymous sites saturation prevents comparisons of too divergent sequences, only species from humans to birds were considered in this analysis.

### Expression analyses

Expressed sequence tag (EST) profiles from several human and murine tissue samples were extracted for *COG *genes from the UniGene database [48] as EST counts per million transcripts and were log2 transformed (*Homo sapiens*: UniGene Build #223 and *Mus musculus*: UniGene Build #183). A number of erroneously assigned ESTs for human and mouse *COG8 *were manually removed. For simplicity, only homologous tissues for which information was available in both organisms were included, resulting in a total of 29 tissues. The clusters on the heatmaps were made by an in house tool, using correlation as the measure of similarity.

In addition, because in several tissues some *COG *genes have no detectable expression (absence of EST counts), we have further tested the presence of all *COG *transcripts in 20 different human tissues included in a RNA panel obtained from Ambion (FirstChoice Human Total RNA Survey Panel). cDNA was synthesized from 1 μg of RNA using the First Strand cDNA synthesis Kit (Fermentas Life Sciences, Burlington, Ontario, Canada), according to the manufacturer's instructions. Primers specific for *COG *transcripts, as well as for the positive control *GAPDH*, were designed to avoid amplification of contaminating genomic DNA, either because they span an intron or the forward primer anneals with an exon/exon boundary (additional file 4). PCR amplifications were performed using the QIAGEN multiplex PCR kit (Qiagen, Hilden, Germany) at 1× Qiagen multiplex PCR master mix with 0.5 μl of cDNA in a 12.5 μl final reaction volume. Final primer concentration in the reaction was 0.4 μM. Thermocycling conditions used included pre-incubation for 15 min at 95°C, followed by 35 cycles of 30 s at 94°C, 90 s at 58°C, and 60 s at 72°C, with a final incubation for 10 min at 72°C. Amplification products ranged from 100 to 253 bp and were separated by horizontal electrophoresis in 12% polyacrylamide gels and visualized by silver staining. RT-PCR products from one sample were confirmed by direct sequencing.

## Results

### Evolutionary Analyses

**Figure 2:**
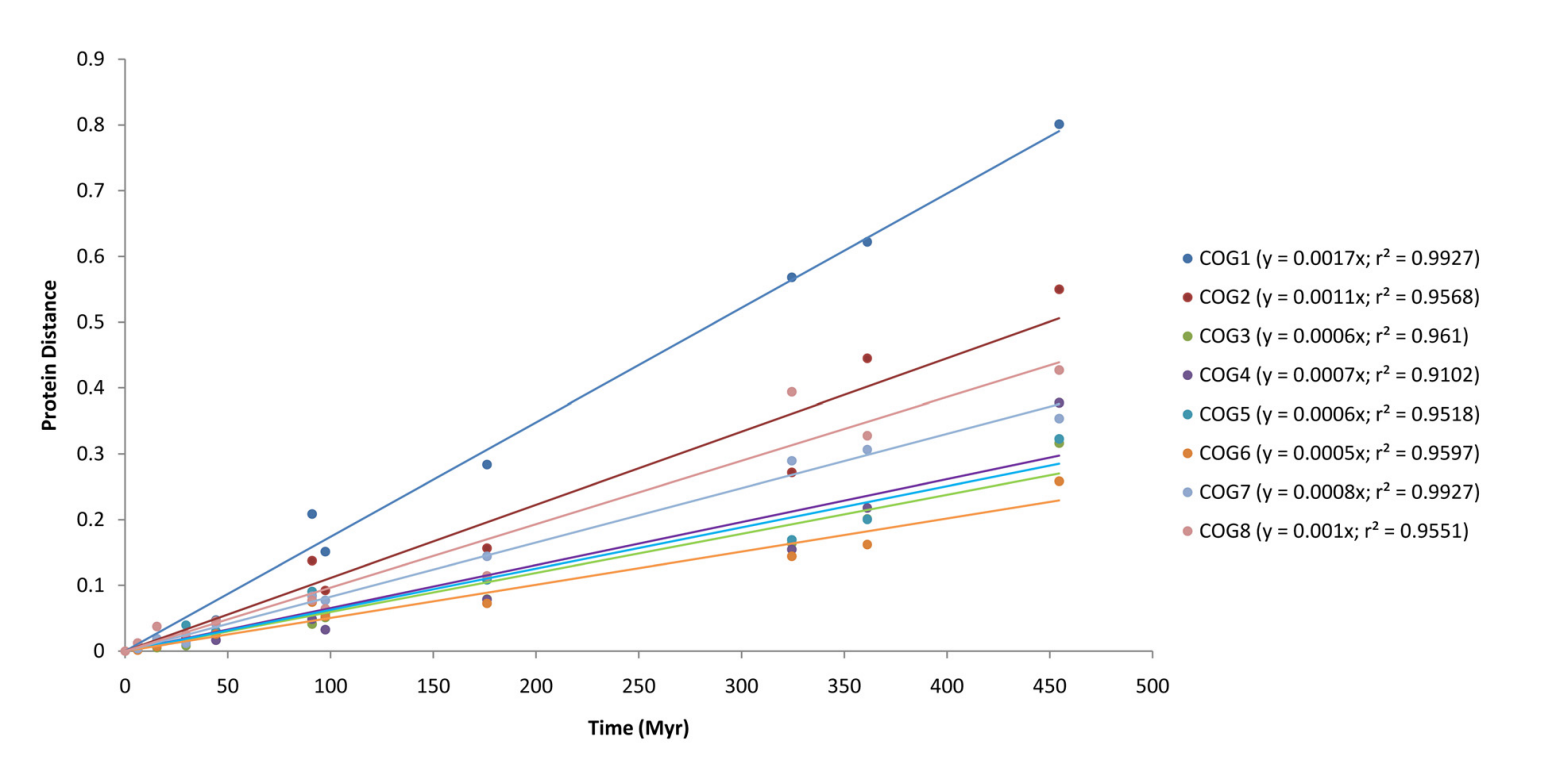
**Protein distances vs divergence times between human and different species**. Divergence times between humans and other species were obtained with TimeTree [38] and are as follow: Ptr: 6.13 Myr; Ppy: 15.44 Myr; Mac: 29.6 Myr; Cja: 44.2 Myr; Glires (average of Mmu, Rno and Ocu): 91 Myr; Laurasiatheria (average of Eca, Bta and Cfa): 97.4 Myr; Mdo: 176.1 Myr; Sauropsida (average of Tgu, Gga and Aca): 324.5 Myr; Xtr: 361.2 Myr; Actinopterygii (average of Dre, Ola, Gac, Tni and Tru): 454.6 Myr. Linear regression trend lines were set to intercept the origin.

As a first measure of the rate of COG proteins evolution, we calculated the pairwise protein distance for human proteins and each of the 20 orthologs. These values were plotted against the corresponding divergence times for the compared species, and the linear regression trend line was estimated from each group, as shown in Figure 2. From the slope of the lines and the r^2 ^values we are able to compare the rate of proteins evolution and its constancy over time, respectively. The results presented in Figure 2 show that COG1 is the subunit with the fastest rate of evolution; COG6, COG3, COG5 and COG4 have the lowest rates; while the remaining proteins (COG7, COG8 and COG2) have intermediate rates. Although being the most divergent protein, COG1 (along with COG7) is the one in which the rate of evolution has remained most constant (r^2 ^= 0.993).

In addition, we used a classical measure of protein evolution based on the nonsynonymous (*d*_N_) to synonymous (*d*_S_) substitutions rate ratio (*d*_N_/*d*_S _or ω).

**Figure 3:**
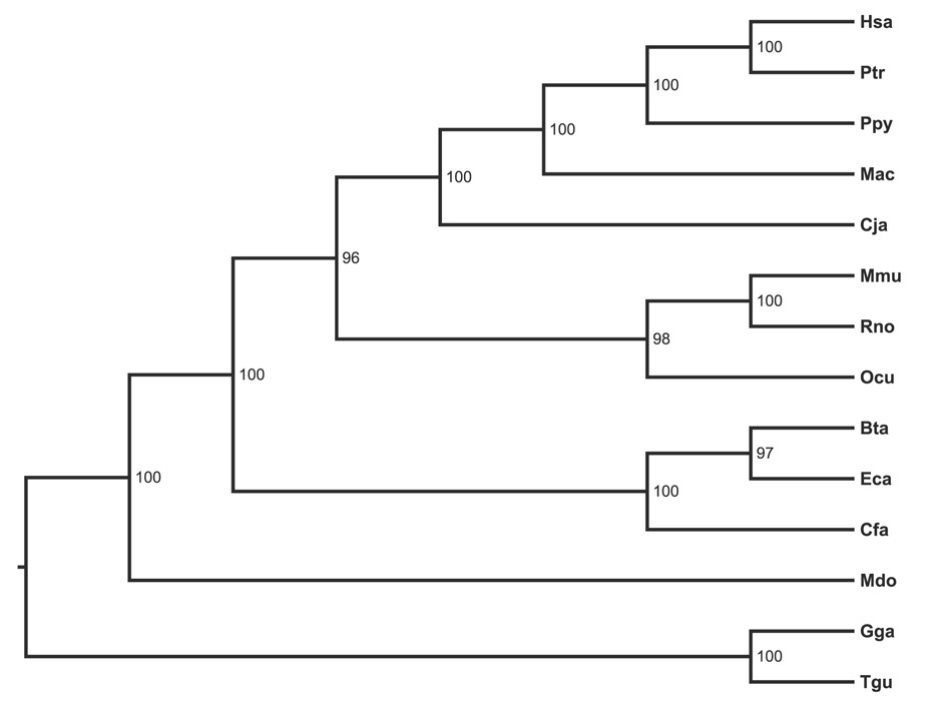
**Maximum Likelihood phylogeny of the 14 species used in the analyses with PAML**. Maximum Likelihood phylogenetic tree was constructed based on the concatenated alignment of the eight independent protein coding sequences alignment (see methods). Tree was midpoint rooted. Bootstrap values are shown at nodes and were calculated from 100 replicates. This tree topology was used to fit different models in PAML.

A ω value higher than 1 can suggest that genes undergo positive selection, while less than 1 is indicative of purifying selection [49]. Following this approach, we tested whether ω ratios for each *COG *gene are different among lineages, based on the maximum likelihood phylogeny previously inferred (Figure 3). Therefore, a likelihood ratio test (LRT) comparing the one-ratio model, that assumes the same ω for all lineages, and the free-ratio model, which assumes independent ω ratios for every branch, was applied. The log likelihoods obtained under each model are presented in Table 1 and indicate significant variation in ω values among lineages in all *COG *genes except for *COG7*, suggesting relaxation of the strong selective constraints in some lineages, yet with low ω. The ω values for branches in the phylogeny for each gene are available at additional file 5. It is interesting to note that the length of the branch that leads to modern rodents (mouse and rat) obtained for *COG2 *and *COG6 *is very long, revealing the accumulation of many substitutions (additional file 5). Notwithstanding, the corresponding ω values are low, even when compared with shorter branches represented by non-rodent species. This suggests that rodent lineage has accumulated mainly synonymous rather than nonsynonymous substitutions, thus preserving the amino acid composition of the encoded protein. In fact, when the same *COG2 *and *COG6 *trees were drawn with branches lengths proportional to the expected nonsynonymous substitutions rate (*d*_N_), the rodent's branch length is more similar to all the other branches (additional file 6).

**Table 1:** Log-likelihood values under the one-ratio and free-ratio models and likelihood ratio statistics (2Δℓ)

	ω^a^	One-ratio (ℓ_0_)	Free-ratios (ℓ_1_)	2Δℓ = 2(ℓ_1_-ℓ_0_)
**COG1**	0.150	-18100.05	-18074.99	50.11*
**COG2**	0.126	-11122.36	-11097.92	48.89*
**COG3**	0.084	-10626.26	-10606.19	40.14*
**COG4**	0.053	-9723.53	-9696.17	54.71*
**COG5**	0.094	-11639.04	-11587.57	102.95*
**COG6**	0.071	-8462.90	-8429.86	66.07*
**COG7**	0.080	-11649.63	-11632.14	34.99
**COG8**	0.094	-9163.24	-9126.69	73.10*

**a **ω ratio obtained under the one-ratio model

b *Significant (*P *< 0.05; $\chi_{24}^{2}$ = 36.415); ℓ: Log likelihood values

Although the ω ratio obtained under the one-ratio model does not fit every branch in the phylogeny, it represents an average over all sites and lineages [50] and therefore can be used to compare the strength of constraints imposed to different *COG *genes. As presented in Table 1, all ω values are very low, indicating that *COG *genes are evolving under strong purifying selection. The highest ratio is observed for *COG1*, while the lowest refers to *COG4 *and *COG6*.

### Expression analyses in human and mouse

Although several studies have been accumulating in the last years about COG complex, particularly in what concerns to the interaction between subunits, thus far the expression profile of different *COG *genes remains uncharacterized. Therefore, as a preliminary approach to study the expression of *COG *genes, tissue dependent expression patterns have been inferred from EST profiles accessible in UniGene database [48]. For comparative purposes, the analysis was performed in homologous tissues in human and mouse for which expression information was available for both organisms.

**Figure 4:**
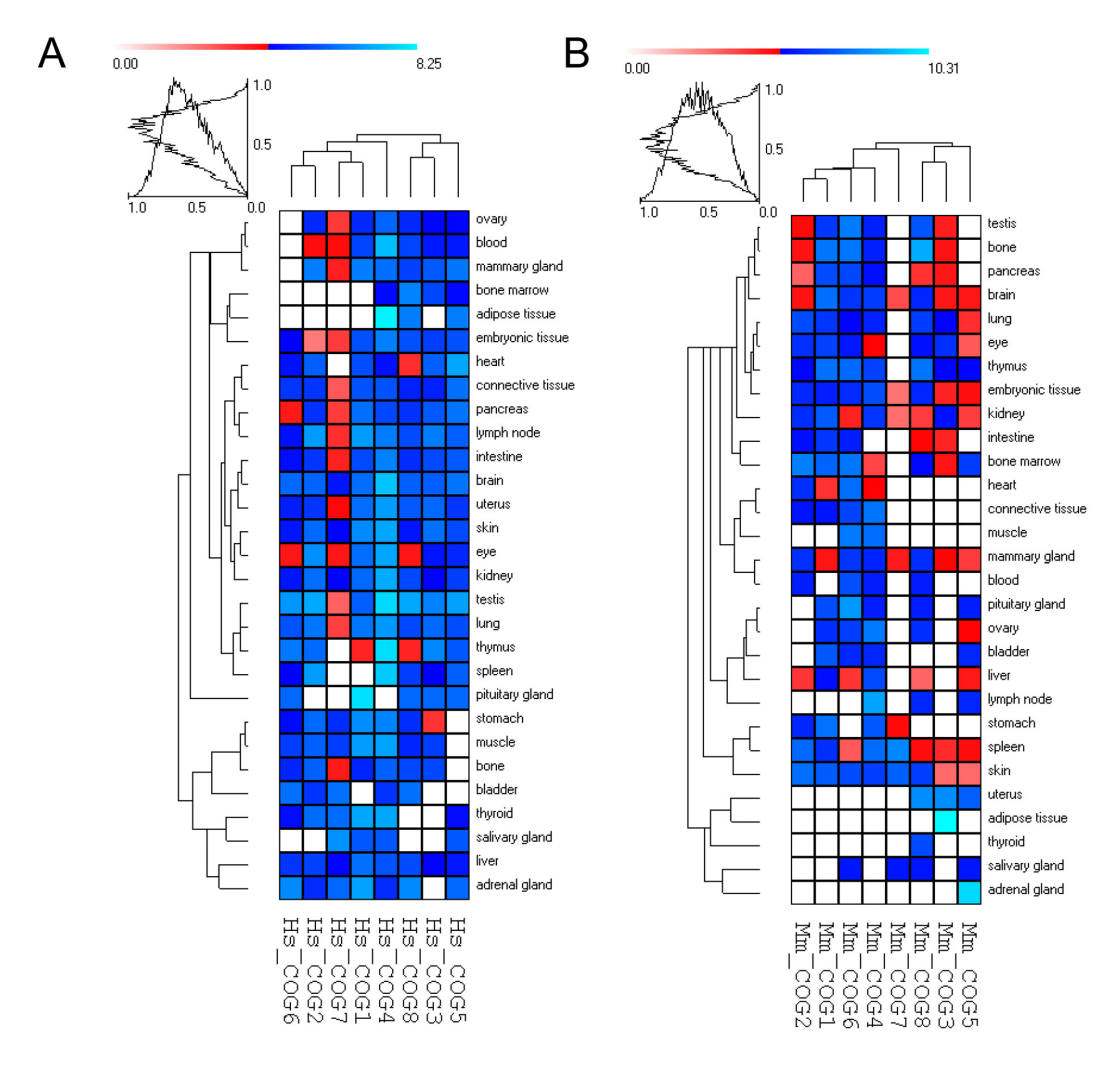
**Cluster of gene expression data obtained from the UniGene database for human (A) and mouse (B)**. EST counts were log2 transformed.

**Figure 5:**
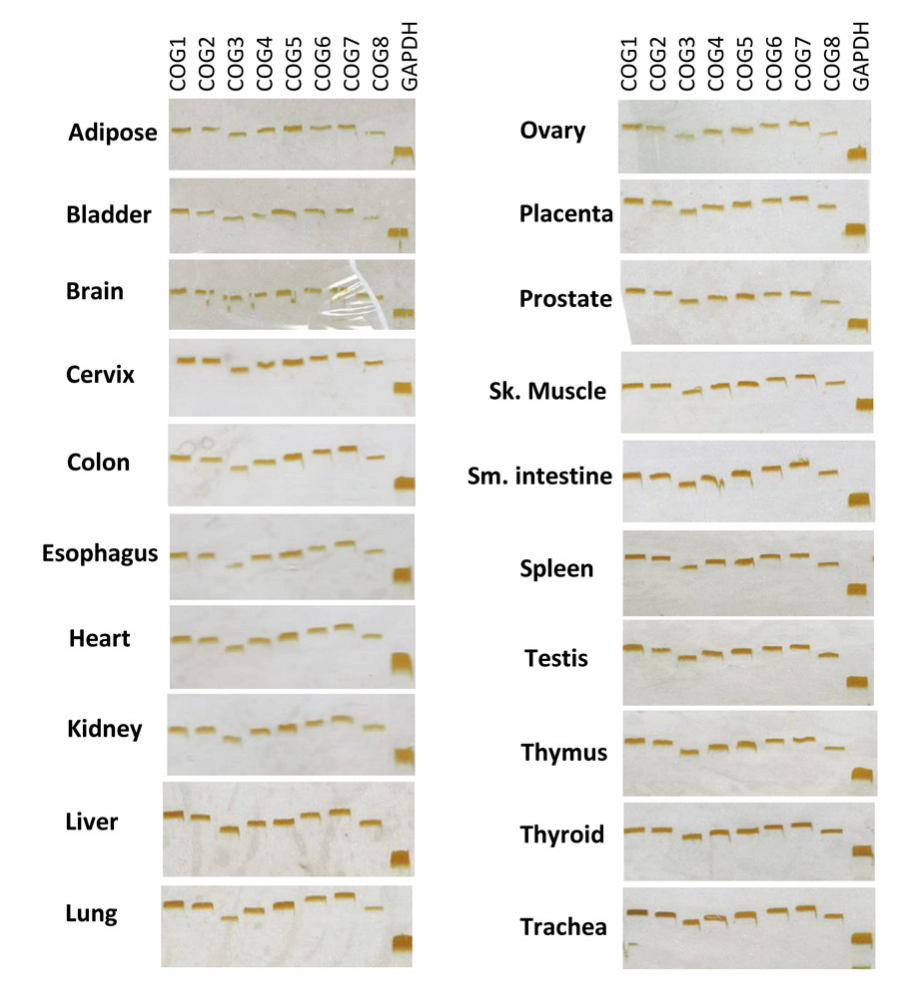
**RT-PCR analyses of the eight *COG *genes expression in 20 human tissues**. *GAPDH *was used as an internal control for the cDNA.

Figure 4 shows the clustering of gene-expression data for human and mouse. In general, *COG *genes appear to have a ubiquitously pattern of expression, yet differences in the level of expression can be observed. However, some of the tissues studied (e.g. adipose tissue) have no detectable expression of specific *COG *genes. In order to assess the presence of *COGs *transcripts in some of those and other human tissues, we performed reverse transcription-PCR (RT-PCR). Although no quantitative inferences could be made, using this simple methodology we were able to detect the eight *COG *transcripts in 20 human tissues (Figure 5) thereby confirming their ubiquitous nature.

It is important to recognize, however, that from EST data and RT-PCR analysis we are not able to infer the precise pattern of expression of *COG *genes, revealing the need for more reliable quantitative data.

## Discussion

COG complex is essential to establish and maintain the structure and function of the Golgi apparatus, which has itself a key role in many cellular processes, such as protein sorting and glycosylation.

In the present study, in order to better understand the evolution of COG subunits in vertebrates, we have applied distinct comparative strategies, including evolutionary and expression analyses.

We demonstrate that all COG proteins are evolving under strong evolutionary constraints, as revealed by the low *d*_N_/*d*_S _values. This pattern of purifying selection must reflect the critical role of COG complex for Golgi function. This is well illustrated by mutations in COG-specific subunits, which give rise to different human diseases belonging to the Congenital Disorders of Glycosylation (CDG). CDGs are a genetically heterogeneous group of disorders characterized by a deficient glycosylation of glycoconjugates, such as proteins and lipids. Since 2004, defects in *COG1 *[51], *COG4 *[52], *COG5 *[53], *COG7 *[19,54-56] and *COG8 *[57,58] have been reported. Recently, a novel mutation in *COG1 *gene was detected in two patients with a cerebrocostomandibular-like syndrome [59], showing that the impact of COG dysfunction is far from being completely known.

Being part of a multi-subunit assembly and having such an important functional role in cells must impose strong constraints on the evolution of COG proteins. On one hand, they must be constrained to maintain the structural integrity of the complex, presumably through the conservation of the residues that are involved in the interaction between subunits. This is expected to be true if we assume that COG structure is maintained by the same type of protein-protein interactions in different species. On the other hand, additional interactions with other functional partners (e.g. other protein related to trafficking, such as SNAREs or small GTPases [reviewed in [60]]) also need to be preserved. The study of the crystallographic structure of the C-terminal region of human COG4 protein, for instance, showed that distinct domains are responsible for the integration of the protein within the complex and for its function [61]. This suggests that a large proportion of the protein sequence of each member of COG complex must be constrained to be evolutionarily conserved.

In fact, the low rate of evolution of COG proteins is consistent with results from more comprehensive studies showing that evolutionary conservation increases from monomeric proteins to members of transient interactions and finally to components of stable complexes (proteins that are permanently associated with each other) [1,62]. Wong and collaborators [4] also demonstrated that as the number of unique proteins in a complex increases, the mean *d*_N_/*d*_S _ratio of the associated genes tends to decrease.

To evaluate the impact of different selective forces on COG proteins, it would be interesting to compare the rates of substitution for interacting and non-interacting residues, and also structural and functional domains. Unfortunately, the structure of fragments of only two COG subunits have been reported [61,63], hampering us to analyze with more detail the evolution of distinct regions of each protein.

Despite all *COG *proteins are evolving under strong selective constraints, COG1 seems to be the one with the highest rate of evolution. This subunit, together with COG8, is the bridging subunits of the mammalian COG complex, bringing together COG2-4 and COG5-7 subcomplexes [22,29]. Interestingly, a quite similar interaction map has been reported in the yeast complex, although in this case only COG1 is required for the association of the two subcomplexes [28]. COG1 from humans and COG1p from yeast share no detectable sequence homology, as happens with COG2 and COG7 [25]. Interestingly, our results revealed that COG1 and COG2 are also the less conserved subunits in vertebrates, suggesting that they are evolving under more relaxed selective constraints. The biological implication of the higher divergence of these proteins is difficult to infer, although we can speculate that it might be related with distinct requirements of COG's function in different species, or with the interactions established by these proteins. The remaining COG proteins (COG3, COG4, COG5, COG6 and COG8), in contrast, have related homologs in human and yeast [25].

In this study we have also demonstrated that the expression of *COG *genes exhibit a ubiquitous nature. These results can be taken as a starting point for more detailed quantitative expression studies that can bring additional insights into COG subunits interaction and function and, eventually, to the understanding of the phenotypic heterogeneity associated with different COG defects.

## Conclusions

In the past years several studies have been focused on the evolution of protein complexes in terms of type of interactions, revealing that proteins in stable complexes are more conserved than those in transient interactions and those with no apparent interacting partners [62].

In this study, in turn, we have investigated the evolution of different subunits belonging to the same protein complex in vertebrates. Our results showed that the eight COG subunits seem to be conserved and evolving under strong purifying selection, in order to maintain the integrity and function of the complex. Finally, we confirm the ubiquitous tissue expression of the eight *COG *transcripts in 20 human tissues.

## Abbreviations

COG: conserved oligomeric Golgi complex; CDG: congenital disorders of glycosylation; EST: expressed sequence tag; Taxa terminology is abbreviated using the first letter of the genus and two letters of the species name (e.g. Hsa corresponds to *Homo sapiens*).

## Authors' contributions

RQ, LA and AA conceived the study and main analyses. RM and RQ carried out the expression analysis. RQ, LA, RM and AA analyzed and interpreted the data. RQ and AA wrote the manuscript. All authors read and approved the final manuscript.

## Supplementary Material

Additional file 1 **Protein accession numbers of COG sequences**. This table provides the accession numbers of the sequences used to perform the analyses. Click here for file

Additional file 2 **Protein sequences alignments used to calculate the proteins distance with Protdist**. Click here for file

Additional file 3 **Protein-coding sequences alignments used in the different analyses performed with PAML**. Click here for file

Additional file 4 **Primer pairs used in PCR reactions**. This table provides the sequences of the primer pairs used to detect the presence of COG transcripts in 20 human tissues. Click here for file

Additional file 5 **Phylogenetic trees with branches drawn in proportion to their lengths, defined as the expected number of nucleotide substitutions per codon, for each *COG *gene**. Values along each branch represent ω ratios (for simplicity of the figure some of them were not indicated) estimated under the free-ratio model. In *COG5 *phylogeny the branch leading to Euarchontoglires (primates and Glires) presents a ω ratio of 4.70 (*d*_N _= 0.0011; *d*_S _= 0.002). A LRT comparing the one-ratio model with the two-ratio model (ω_background; _ω_Euarchontoglires_), revealed that the estimated ω ratio for the Euarchontoglires branch (infinite, indicating the absence of synonymous substitutions) was not significantly higher than the background ratio (0.094). Moreover, the LRT comparing the two-ratio model with and without the constraint ω_Euarchontoglires _≤ 1 revealed that this ratio was not significantly higher than 1 as well. Click here for file

Additional file 6 ***COG2 *and *COG6 *genes phylogeny with branch lengths defined as the estimated nonsynonymous substitutions rate (*d*_N_) or the estimated synonymous substitutions rate (*d_S_*)**. Click here for file

## References

[B1] Pereira-Leal JB Levy ED Teichmann SA The origins and evolution of functional modules: lessons from protein complexes Philos Trans R Soc Lond B Biol Sci 2006 361 1467 507 517 10.1098/rstb.2005.1807 16524839 PMC1609335

[B2] Mintseris J Weng Z Structure, function, and evolution of transient and obligate protein-protein interactions Proc Natl Acad Sci USA 2005 102 31 10930 10935 10.1073/pnas.0502667102 16043700 PMC1182425

[B3] van Dam TJ Snel B Protein complex evolution does not involve extensive network rewiring PLoS Comput Biol 2008 4 7 e1000132 10.1371/journal.pcbi.1000132 18711636 PMC2517612

[B4] Wong P Althammer S Hildebrand A Kirschner A Pagel P Geissler B Smialowski P Blochl F Oesterheld M Schmidt T Strack N Theis F Ruepp A Frishman D An evolutionary and structural characterization of mammalian protein complex organization BMC Genomics 2008 9 1 629 10.1186/1471-2164-9-629 19108706 PMC2645396

[B5] Whyte JRC Munro S Vesicle tethering complexes in membrane traffic J Cell Sci 2002 115 13 2627 2637 12077354 10.1242/jcs.115.13.2627

[B6] Oka T Krieger M Multi-Component Protein Complexes and Golgi Membrane Trafficking J Biochem 2005 137 2 109 114 10.1093/jb/mvi024 15749823

[B7] Rothman JE Mechanisms of intracellular protein transport Nature 1994 372 6501 55 63 10.1038/372055a0 7969419

[B8] Kingsley D Kozarsky K Segal M Krieger M Three types of low density lipoprotein receptor-deficient mutant have pleiotropic defects in the synthesis of N-linked, O-linked, and lipid- linked carbohydrate chains J Cell Biol 1986 102 5 1576 1585 10.1083/jcb.102.5.1576 3700466 PMC2114220

[B9] Lupashin V Sztul E Golgi tethering factors Biochim Biophys Acta 2005 1744 3 325 339 10.1016/j.bbamcr.2005.03.013 15979505

[B10] Ungar D Oka T Brittle EE Vasile E Lupashin VV Chatterton JE Heuser JE Krieger M Waters MG Characterization of a mammalian Golgi-localized protein complex, COG, that is required for normal Golgi morphology and function J Cell Biol 2002 157 3 405 415 10.1083/jcb.200202016 11980916 PMC2173297

[B11] Podos S Reddy P Ashkenas J Krieger M LDLC encodes a brefeldin A-sensitive, peripheral Golgi protein required for normal Golgi function J Cell Biol 1994 127 3 679 691 10.1083/jcb.127.3.679 7962052 PMC2120235

[B12] Walter DM Paul KS Waters MG Purification and characterization of a novel 13 S hetero-oligomeric protein complex that stimulates in vitro Golgi transport J Biol Chem 1998 273 45 29565 29576 10.1074/jbc.273.45.29565 9792665

[B13] Chatterton JE Hirsch D Schwartz JJ Bickel PE Rosenberg RD Lodish HF Krieger M Expression cloning of LDLB, a gene essential for normal Golgi function and assembly of the ldlCp complex Proc Natl Acad Sci USA 1999 96 3 915 920 10.1073/pnas.96.3.915 9927668 PMC15325

[B14] Suvorova ES Kurten RC Lupashin VV Identification of a human orthologue of Sec34p as a component of the cis-Golgi vesicle tethering machinery J Biol Chem 2001 276 25 22810 22818 10.1074/jbc.M011624200 11292827

[B15] Ungar D Oka T Krieger M Hughson FM Retrograde transport on the COG railway Trends Cell Biol 2006 16 2 113 120 10.1016/j.tcb.2005.12.004 16406524

[B16] Suvorova ES Duden R Lupashin VV The Sec34/Sec35p complex, a Ypt1p effector required for retrograde intra-Golgi trafficking, interacts with Golgi SNAREs and COPI vesicle coat proteins J Cell Biol 2002 157 4 631 643 10.1083/jcb.200111081 12011112 PMC2173848

[B17] Zolov SN Lupashin VV Cog3p depletion blocks vesicle-mediated Golgi retrograde trafficking in HeLa cells J Cell Biol 2005 168 5 747 759 10.1083/jcb.200412003 15728195 PMC2171815

[B18] Oka T Ungar D Hughson FM Krieger M The COG and COPI complexes interact to control the abundance of GEARs, a subset of Golgi integral membrane proteins Mol Biol Cell 2004 15 5 2423 2435 10.1091/mbc.E03-09-0699 15004235 PMC404034

[B19] Wu X Steet RA Bohorov O Bakker J Newell J Krieger M Spaapen L Kornfeld S Freeze HH Mutation of the COG complex subunit gene COG7 causes a lethal congenital disorder Nat Med 2004 10 5 518 523 10.1038/nm1041 15107842

[B20] Shestakova A Zolov S Lupashin V COG complex-mediated recycling of Golgi glycosyltransferases is essential for normal protein glycosylation Traffic 2006 7 2 191 204 10.1111/j.1600-0854.2005.00376.x 16420527

[B21] Wuestehube LJ Duden R Eun A Hamamoto S Korn P Ram R Schekman R New mutants of *Saccharomyces cerevisiae *affected in the transport of proteins from the endoplasmic reticulum to the Golgi complex Genetics 1996 142 2 393 406 8852839 10.1093/genetics/142.2.393 PMC1206974

[B22] Oka T Vasile E Penman M Novina CD Dykxhoorn DM Ungar D Hughson FM Krieger M Genetic analysis of the subunit organization and function of the conserved oligomeric golgi (COG) complex: studies of COG5- and COG7-deficient mammalian cells J Biol Chem 2005 280 38 32736 32745 10.1074/jbc.M505558200 16051600

[B23] Smith RD Lupashin VV Role of the conserved oligomeric Golgi (COG) complex in protein glycosylation Carbohydr Res 2008 343 12 2024 2031 10.1016/j.carres.2008.01.034 18353293 PMC2773262

[B24] Loh E Hong W Sec34 is implicated in traffic from the endoplasmic reticulum to the Golgi and exists in a complex with GTC-90 and ldlBp J Biol Chem 2002 277 24 21955 21961 10.1074/jbc.M202326200 11929878

[B25] Whyte JR Munro S The Sec34/35 Golgi transport complex is related to the exocyst, defining a family of complexes involved in multiple steps of membrane traffic Dev Cell 2001 1 4 527 537 10.1016/S1534-5807(01)00063-6 11703943

[B26] Ram RJ Li B Kaiser CA Identification of Sec36p, Sec37p, and Sec38p: components of yeast complex that contains Sec34p and Sec35p Mol Biol Cell 2002 13 5 1484 1500 10.1091/mbc.01-10-0495 12006647 PMC111121

[B27] Loh E Hong W The binary interacting network of the conserved oligomeric Golgi tethering complex J Biol Chem 2004 279 23 24640 24648 10.1074/jbc.M400662200 15047703

[B28] Fotso P Koryakina Y Pavliv O Tsiomenko AB Lupashin VV Cog1p plays a central role in the organization of the yeast conserved oligomeric Golgi complex J Biol Chem 2005 280 30 27613 27623 10.1074/jbc.M504597200 15932880

[B29] Ungar D Oka T Vasile E Krieger M Hughson FM Subunit architecture of the conserved oligomeric Golgi complex J Biol Chem 2005 280 38 32729 32735 10.1074/jbc.M504590200 16020545

[B30] Foulquier F COG defects, birth and rise! Biochimi Biophys Acta 2009 1792 9 896 902 10.1016/j.bbadis.2008.10.020 19028570

[B31] Zeevaert R Foulquier F Jaeken J Matthijs G Deficiencies in subunits of the Conserved Oligomeric Golgi (COG) complex define a novel group of Congenital Disorders of Glycosylation Mol Genet Metab 2008 93 1 15 21 10.1016/j.ymgme.2007.08.118 17904886

[B32] NCBI- National Center for Biotechnology Information http://www.ncbi.nlm.nih.gov/

[B33] Altschul SF Gish W Miller W Myers EW Lipman DJ Basic local alignment search tool J Mol Biol 1990 215 3 403 410 2231712 10.1016/S0022-2836(05)80360-2

[B34] Koonin EV Orthologs, paralogs, and evolutionary genomics Annu Rev Genet 2005 39 1 309 338 10.1146/annurev.genet.39.073003.114725 16285863

[B35] http://www.ensembl.org/index.html

[B36] Edgar RC MUSCLE: multiple sequence alignment with high accuracy and high throughput Nucleic Acids Res 2004 32 5 1792 1797 10.1093/nar/gkh340 15034147 PMC390337

[B37] Felsenstein J PHYLIP (Phylogeny Inference Package) version 3.69 Distributed by the author Department of Genome Sciences University of Washington, Seattle

[B38] Hedges SB Dudley J Kumar S TimeTree: a public knowledge-base of divergence times among organisms Bioinformatics 2006 22 23 2971 2972 10.1093/bioinformatics/btl505 17021158

[B39] Hedges SB Kumar S (eds) The Timetree of Life 2009 New York: Oxford University Press

[B40] Thompson JD Higgins DG Gibson TJ CLUSTAL W: improving the sensitivity of progressive multiple sequence alignment through sequence weighting, position-specific gap penalties and weight matrix choice Nucl Acids Res 1994 22 22 4673 4680 10.1093/nar/22.22.4673 7984417 PMC308517

[B41] Hall TA BioEdit: a user-friendly biological sequence alignment editor and analysis program for Windows 95/98/NT Nucleic Acids Symp Ser 1999 41 95 98

[B42] Castresana J Selection of Conserved Blocks from Multiple Alignments for Their Use in Phylogenetic Analysis Mol Biol Evol 2000 17 4 540 552 10742046 10.1093/oxfordjournals.molbev.a026334

[B43] Posada D jModelTest: Phylogenetic Model Averaging Mol Biol Evol 2008 25 7 1253 1256 10.1093/molbev/msn083 18397919

[B44] Guindon S Gascuel O A simple, fast, and accurate algorithm to estimate large phylogenies by maximum likelihood Syst Biol 2003 52 5 696 704 10.1080/10635150390235520 14530136

[B45] Rambaut A FigTree-Tree Figure Drawing Tool Version 1.2.2 http://tree.bio.ed.ac.uk/software/figtree/

[B46] Yang Z PAML 4: Phylogenetic Analysis by Maximum Likelihood Mol Biol Evol 2007 24 8 1586 1591 10.1093/molbev/msm088 17483113

[B47] Yang Z Likelihood ratio tests for detecting positive selection and application to primate lysozyme evolution Mol Biol Evol 1998 15 5 568 573 9580986 10.1093/oxfordjournals.molbev.a025957

[B48] Pontius J Wagner L Schuler G UniGene: a unified view of the transcriptome The NCBI Handbook 2003 Bethesda (MD): National Center for Biotechnology Information

[B49] Jobling MA Hurles M Tyler-Smith C Human Evolutionary Genetics: Origins, Peoples and Disease 2004 New York: Garland Science

[B50] Yang Z Swanson WJ Vacquier VD Maximum-Likelihood Analysis of Molecular Adaptation in Abalone Sperm Lysin Reveals Variable Selective Pressures Among Lineages and Sites Mol Biol Evol 2000 17 10 1446 1455 11018152 10.1093/oxfordjournals.molbev.a026245

[B51] Foulquier F Vasile E Schollen E Callewaert N Raemaekers T Quelhas D Jaeken J Mills P Winchester B Krieger M Annaert W Matthijs G Conserved oligomeric Golgi complex subunit 1 deficiency reveals a previously uncharacterized congenital disorder of glycosylation type II Proc Natl Acad Sci USA 2006 103 10 3764 3769 10.1073/pnas.0507685103 16537452 PMC1450151

[B52] Reynders E Foulquier F Leao Teles E Quelhas D Morelle W Rabouille C Annaert W Matthijs G Golgi function and dysfunction in the first COG4-deficient CDG type II patient Hum Mol Genet 2009 18 17 3244 3256 10.1093/hmg/ddp262 19494034 PMC2722986

[B53] Paesold-Burda P Maag C Troxler H Foulquier F Kleinert P Schnabel S Baumgartner M Hennet T Deficiency in COG5 causes a moderate form of congenital disorders of glycosylation Hum Mol Genet 2009 18 22 4350 4356 10.1093/hmg/ddp389 19690088

[B54] Ng BG Kranz C Hagebeuk EE Duran M Abeling NG Wuyts B Ungar D Lupashin V Hartdorff CM Poll-The BT Freeze HH Molecular and clinical characterization of a Moroccan Cog7 deficient patient Mol Genet Metab 2007 91 2 201 204 10.1016/j.ymgme.2007.02.011 17395513 PMC1941618

[B55] Morava E Zeevaert R Korsch E Huijben K Wopereis S Matthijs G Keymolen K Lefeber DJ De Meirleir L Wevers RA A common mutation in the COG7 gene with a consistent phenotype including microcephaly, adducted thumbs, growth retardation, VSD and episodes of hyperthermia Eur J Hum Genet 2007 15 6 638 645 10.1038/sj.ejhg.5201813 17356545

[B56] Zeevaert R Foulquier F Cheillan D Cloix I Guffon N Sturiale L Garozzo D Matthijs G Jaeken J A new mutation in COG7 extends the spectrum of COG subunit deficiencies Eur J Med Genet 2009 52 5 303 305 10.1016/j.ejmg.2009.06.006 19577670

[B57] Foulquier F Ungar D Reynders E Zeevaert R Mills P Garcia-Silva MT Briones P Winchester B Morelle W Krieger M Annaert W Matthijs G A new inborn error of glycosylation due to a Cog8 deficiency reveals a critical role for the Cog1-Cog8 interaction in COG complex formation Hum Mol Genet 2007 16 7 717 730 10.1093/hmg/ddl476 17220172

[B58] Kranz C Ng BG Sun L Sharma V Eklund EA Miura Y Ungar D Lupashin V Winkel RD Cipollo JF Costello CE Loh E Hong W Freeze HH COG8 deficiency causes new congenital disorder of glycosylation type IIh Hum Mol Genet 2007 16 7 731 741 10.1093/hmg/ddm028 17331980

[B59] Zeevaert R Foulquier F Dimitrov B Reynders E Van Damme-Lombaerts R Simeonov E Annaert W Matthijs G Jaeken J Cerebrocostomandibular-like syndrome and a mutation in the conserved oligomeric Golgi complex, subunit 1 Hum Mol Genet 2009 18 3 517 524 10.1093/hmg/ddn379 19008299

[B60] Sztul E Lupashin V Role of vesicle tethering factors in the ER-Golgi membrane traffic FEBS Lett 2009 583 23 3770 3783 10.1016/j.febslet.2009.10.083 19887069 PMC2788073

[B61] Richardson BC Smith RD Ungar D Nakamura A Jeffrey PD Lupashin VV Hughson FM Structural basis for a human glycosylation disorder caused by mutation of the COG4 gene Proc Natl Acad Sci USA 2009 106 32 13329 13334 10.1073/pnas.0901966106 19651599 PMC2716380

[B62] Teichmann SA The Constraints Protein-Protein Interactions Place on Sequence Divergence J Mol Biol 2002 324 3 399 407 10.1016/S0022-2836(02)01144-0 12445777

[B63] Cavanaugh LF Chen X Richardson BC Ungar D Pelczer I Rizo J Hughson FM Structural analysis of conserved oligomeric Golgi complex subunit 2 J Biol Chem 2007 282 32 23418 23426 10.1074/jbc.M703716200 17565980

