# Supplementary material for: Comparative analyses of the Conserved Oligomeric Golgi (COG) complex in vertebrates
Source: BMC Evol Biol. 2010 Jul 15;10:212. doi: 10.1186/1471-2148-10-212 (PMC2927914; doi:10.1186/1471-2148-10-212)
Supplement: Additional file 2 — Protein sequences alignments used to calculate the proteins distance with Protdist. [file 1471-2148-10-212-S2.PDF]

```
Hsa_COG1   MATAATSPALKRLDLRDPAALFETHGAEEIRGLERQVRAEIEHKKEELRQMVGERYRDLIEAADTIGQMRRCAVGLVDAVKATDQYCARLRQAGSAAPRPPRAQQPQQPS 110
Ptr_COG1   ........S..........................R.......................................................................... 110
Ppy_COG1   ...G.......................................................................................................... 110
Mac_COG1   ..A....................................................................................................P...... 110
Cja_COG1   ..A....................................................................................................P...... 110
Mmu_COG1   ..A.TA.S..........N......................................................E......Q..............V...V...P...P.. 110
Rno_COG1   ..A.TA.S..........N......................................................E......Q................S.V...P..HP.. 110
Ocu_COG1   ---------------------------------------------------M..W-------------------------------------------------AQPRQL 10
Eca_COG1   ..A..A...................................................................E............................DP...... 110
Cfa_COG1   VTASVGHSV.QAEEQQQ..S.P.MT.SRQRK.IPQRLGST........L........................E.................LRLP.......DP...... 110
Bta_COG1   ..A..A.S.................................................................A......R.....................DP...... 110
Mdo_COG1   AVAM.G.Q.....E...AE....A..............................................C..E..L...R....C......QSH.T.VL.ST-SSKK.. 109
Tgu_COG1   ..APPSGD.R..RP.PEAE....A.T.A.L.AV..RL..G..Q.R.....................AE...S.ER.LG..RGLQR-GGAA.PGP-.GTVRG.G.R.SE.. 108
Gga_COG1   ..ARGA----------EAE......T.A.L.EA..RL..G..Q.R.....................AE..LS.ER.LGS.RGLQR-GGVT.PGP-.P.A...V.EKLY.G 98
Aca_COG1   -GGPGA-----------AE....A.T.A.L.AV..RL..G..G.R.....................AE..LS.QR.LE..RGLQRQQQQQ..GS---AKGR.DPK.MP.P 95
Xtr_COG1   AVAI.MAATM.LSEIK..S....A.S.....V...R......Q........................E...SSGLV.G..RDMEK..GS.KCKQ.TGAAGTQRDRAASQC 110
Dre_COG1   -MATVPAQS.RVSEIK..TV...RY.TD...AV..K..G...Q.................D......E..Q.SESV.HSIQDMYR..HK.K..K-QT.QSSSRAEG.KQ. 108
Gac_COG1   -MAEDPALS.RVSEIK..EV...RYNTA...RV..K..G...Q.................D......E..Q.SESV.QSIQDMHR..HQ.K.GR--.GTTSCR.EDPTQW 107
Ola_COG1   -MAQDPA.V.RLSEMK..LV...RYNT....RI..R..GD..Q.................D......E..Q.SERV.RS.QDMQRF.QL.K.SR-PGAGAAA.E.N.RQV 108
Tni_COG1   EMADDNTLP.RVSEIK.S.V...RYNT.Q..KI..K..G...Q.................D.....RE..Q.SESV.RS.QDMQR..HT.K.GK--SGV.SSRLEN..QL 108
Tru_COG1   -MADGNAFP.RLSEIK...V...RYNT.Q..RI..K..G...Q.................D.....RE..Q.SESV.ES.QDMQ...QT.K.GR.SNSSSSSSLEN..QL 109

Hsa_COG1   QEKFYSMAAQIKLLLEIPEKIWSSMEASQCLHATQLYLLCCHLHSLLQLDSSSSR-YSPVLSRFPILIRQVAAASHFRSTILHESKMLLKCQGVSDQAVAEALCSIMLLE 219
Ptr_COG1   .......................................................-....................................A................. 219
Ppy_COG1   .......................................................-....................................A................. 219
Mac_COG1   .............................Y..............N..........-....................................A................. 219
Cja_COG1   .............................Y.........................-H...................................AM................ 219
Mmu_COG1   -......................A.....H.Q....................N..-...I................................A................. 218
Rno_COG1   -......................C.....H.Q.......................-...I.......V........................A................. 218
Ocu_COG1   SP..................M..A....RY..........R........EAAA..-S....A.....V..........-------------.P.---------------- 90
Eca_COG1   .............................Y..............N.......G.Q-C...................................A............V.... 219
Cfa_COG1   .............................Y..............N.......T..-....................................A...........A..... 219
Bta_COG1   ............................RY...........Y..K.....E....-H...................................A................. 219
Mdo_COG1   .............................Y..............G..R....GC-.....A.....V.................T..I.R.A...........SV.... 218
Tgu_COG1   .QG..GA...L....V..QV.GAV.GGRY.P.AR.H..GA..RRQ...TPRA.-A..I.A.....L.......L....Q...A..R.PTG..........A..... 217
Gga_COG1   GGTAEAA.GHPRAGV.--------RHGGRAIP.RRPP.PAVPPPARPAAARRAP.PLQ.RPRPL...L.............Q...S...S.T..........A..... 200
Aca_COG1   AKAAEAW.N-----.GP..NL--ECHGVLPVPVCHPVVPAVLPPPWAPAVGFFWHPLQ.N-A.....A......NY.S....Q...S...S.T.L..G......AA.... 197
Xtr_COG1   .....CT........V.....A....Y................H....P..-.....A.....V......GT..T...Q...S....PSA......G.....I... 219
Dre_COG1   .....T..S..........RV..A..S..Y.Q............Q...EGGGHQ-..........V......G.......L...SV.RGRA....I....V.T.... 217
Gac_COG1   .....T..S..........R...A....Y.Q.................GAATGGN....V.....V....TTG.......L..RS..QGRA....I....V.T.... 217
Ola_COG1   .DG..C....VA...D...R...A...A.Y.Q..R.........G..R...A.AGHF....A.....V...STTG.......MD..S..RGRA....I....V.T.... 218
Tni_COG1   .KN..T..S......D...R...A.....Y.E................EATPGGH.....V.....V....TTG.......LD..S..RGRA....I....V.T.... 218
Tru_COG1   .....T..S......D...R...A....RY.E.............N....ETTT.GH.....L.....V....TTG.......LD..S..RGRA....I....V.T.... 219

Hsa_COG1   ESSPRQALTDFLLARKATIQKLLNQPHHGAGIKAQICSLVELLATTLKQAHALFYTLPEGLLP--DPALPCGLLFSTLETITGQHPAGKGTGVLQEEMKLCSWFKHLPAS 327
Ptr_COG1   ...............................................................--............................................. 327
Ppy_COG1   ...............................................................--............................................. 327
Mac_COG1   ...............................................................--............................................. 327
Cja_COG1   ..............................................V................--.....................T.........G............. 327
Mmu_COG1   ....................T....S.....................N............V..--..S............V.R...T...I.A..G........R...T. 326
Rno_COG1   ....................T..........................N............V..--..S............V.R...T...ISA..G........R..... 326
Ocu_COG1   -----------------------------------------------------------------------DVSTV.A.EQSQAGRER..A....G........R..... 129
Eca_COG1   ........A........A.....S..L....................N...............--..S..................T...IS.........G........ 327
Cfa_COG1   .................A............S................N............Q..--..S..................T..................Y.... 327
Bta_COG1   .................A.............................N...............--..L...........DS..S...S...VW...D............. 327
Mdo_COG1   D................A.H...........................Y............V..--...............DSV.S.QL....IINVLQ............ 326
Tgu_COG1   D.......A.......LA..Q..............V...M...S...Y........V...MA.--E..............SS..N...R.-...E.DL..S....Y..E. 324
```

```
Gga_COG1    D.......A.......LA..Q..............V...M...T...Y.......MM...VP.--...............ST..Q.....-...ED.V..S...RY..E. 307
Aca_COG1    D.......A.......SA..Q.......D......V....D......F............TPA--..S..............S...T...IT..N..S..S.S..Y..P. 305
Xtr_COG1    G.T.......Y......G..Q........L.....V........N..Y........QS.EMK.--E.S.T.....TM.D.V...QGG...IN..K....SV.......P. 327
Dre_COG1    D.......A........S..Q.....Q....V...V.......V...Y..Y.V..VP...QVS--.TG.G.....T...NV.SNTSS..EKK..DK..STG.......P. 325
Gac_COG1    D.......A........S.HQ.....Q........V.......V...F..Y.V..LP...GPRPGEG..S..M......NV.STTY.A.DRT.....TSTGC...Y..H. 327
Ola_COG1    D....R..A.......SS.HQ.....Q........V......FV...F..Y.V..LP...APRPGEG..G..M..TI..RV.SCSS.A..SK.....GSTGT.RF..P. 328
Tni_COG1    D.......A..........HQ.....Q........V..V....V...F.VY.V..VPR..SPRPGEGG.SY.M...I..NV.SPT..V.SRRL....TSTGG...Y..P. 328
Tru_COG1    D.......A........S.HQ.....Q........V..V....V...F..Y.V..SAR..GPRSGEGG.SY........NV.FTT..V..RRL....TSTGG...YV.P. 329

Hsa_COG1    IVEFQPTLRTLAHPISQEYLKDTLQKWIHMCNEDIKNGITNLLMYVKSMKGLAGIRDAMWELLTNESTNHSWDVLCRRLLEKPLLFWEDMMQQLFLDRLQTLTKEGFDSI 437
Ptr_COG1    .............................................................................................................. 437
Ppy_COG1    .............................................................................................................. 437
Mac_COG1    ......................................V............................A.......................................... 437
Cja_COG1    V...........Y..................H..........................................I..............I.................... 437
Mmu_COG1    .I..........................D..........G..................I.D..S...AS...E.V.Q............L.............R...E.. 436
Rno_COG1    VT....A..............G..R...D..S.......G..................I.D......AS...E.V.Q............L.............R...... 436
Ocu_COG1    V.....A.............R.S....V...K........S.........A..A....V.D...H.AAS.....I......Q..S..................R...... 239
Eca_COG1    V.....A.............R........V.............K......A.............NM..A.G.T.V..............L..R................. 437
Cfa_COG1    ..Q...A.....Q..............................L..............................I.Q............L.................... 437
Bta_COG1    V.....A......................V.....R...SS.....T.T.........V.G..A...IH.....V.....D........L.................... 437
Mdo_COG1    .M.............NE...........N..H....T......V....L.........V.....S..MSQ.E.V.....D..IS....LL............RD..... 436
Tgu_COG1    V....A..............RE...Q..N..SD..RT.VRS..V...........V....SS.ASS.N.EAV.....DR.VS...ELL.E................ 434
Gga_COG1    V..............D..R......A..S...RA.VS...V....L.........V.....S..ISQN.........D..AS....LLR.......EI......E.V 417
Aca_COG1    VT....A.........D...G......NV.....VS...S..V....L.........V.....S..MSQN.ETV.C...D...SL...LLR....N.......E....D. 415
Xtr_COG1    V.N...ER............CE...Q..S.......V..SA..V....L.........V.....S..MSQN..KV.H...DR.FR....IL......E...A......E.. 437
Dre_COG1    VMN...V.....Q..QR.Q.R....Q..DT.K...RS.VSS..V....L....A....V....SS..ISQH.STV.QS...Q..AL.D.LL.....R...VI.Q..TEG. 435
Gac_COG1    .T....A.....K..QR.Q.R....Q..DT.K...CR.VSG..V....L....A...SV.D..ST..ISQH.SAV.Q....R..AV.D.FL.....Q...AI...ETEA. 437
Ola_COG1    VT...........Q..QR.Q.Q....Q..DTVK...CR.VSS..V....L....A....V.D..STD.ISQH.SAV.Q....R..AV...FL.....Q...AI...ETEA. 438
Tni_COG1    .I...........Q..QT.Q.R....Q...T.K...CH.VGS..V....L....A....V.D..STD.IS.H.NTV.Q....R..AV...LL.....Q...AI...ETEA. 438
Tru_COG1    .T...........Q..QS.Q.R....Q...T.K...CH.VGS..V....L....A....V.D..STD.ISQH.NTV.Q....R..AV.D.FL.....Q...AI...ETEA. 439

Hsa_COG1    SSSSKELLVSALQELESSTSNSPSNKHIHFEYNMSLFLWSESPNDLPSDAAWVSVANRGQ-FASSGLSMKAQAISPCVQNFCSALDSKLKVKLDDLLAYLPSDDSSLPKD 546
Ptr_COG1    ............................................................-................................................. 546
Ppy_COG1    .....................................................N......-................................................. 546
Mac_COG1    ............................................................-................................................. 546
Cja_COG1    ....................G.S........H.....................T....V.-..N...............................I.............. 546
Mmu_COG1    .N..............T--N..T....V...Q...F......................A.-.........................................S.TP.L.. 543
Rno_COG1    ................A--N..T....V...Q....Y.....................A.-..N......................................S.TP.L.. 543
Ocu_COG1    A..CE...A........G.GGS.ACS..V.Y.Q..C........AG...P.....T....AP-A.G........R.V.....G.......A...............EE.----- 343
Eca_COG1    .A..R...............ATP.......H..A.......SS...P.....N......-L.G.........V...................E..M.....G....S.. 546
Cfa_COG1    .T.....I.......NT....T.......H...........H...P........H...-...........................................P.... 546
Bta_COG1    ....T...T........--.S.A.S..L...H..AV.......S.............SS.AP-.QG.............S.............E.........G.PLPRQ. 544
Mdo_COG1    .....K..........A.-.STSP..QVQ..H...........S.........N....S.-C..........L..Y..S..L..............S.....AP---.E 541
Tgu_COG1    .....Q..AG......VKAGSGALS.Q.QQ.H.VA........G...G.......GQ..P-..R...A.....LT....S...T...Q..AR.E.V.S....G...VPKEP 543
Gga_COG1    .....Q..IL......AKSNT.AFS...Q..H..AQ.......SS.........N....S.-..K.........LT..I.S...........AR.....S...AES.-PT.E 525
Aca_COG1    ....R....Q.I....AKFDA.AP..S.L..H..AS.....N.....L....I..G..S.-..K.........H..G..S...........A.......S...M.SP-ST.. 523
Xtr_COG1    .RNLVQ..M.S......---G.PETL..LQH.S.ICS.....G.S....P.....N.GS..L-QCR.....L....VT..I.SL.AT...Q.....E..CS...GESPTEKHN 543
Dre_COG1    ....RQ..S....RD.QGQA.AGSFSRGAQY.SDVGA.......QS..L...T.......Q.SP-QQK.......T..LT....T....S.......A..E..QH......TN-GKET 543
Gac_COG1    .T..IQ..T.TVRD..A--T.PG.ARGAQY.VDVAS........G..L...G....NQ...KHQR.S.A..T..VT.F.......S..A...AR....RY...FQ.PVSAPP 545
Ola_COG1    AA..VQ..S.....D.Q.Q.PSPSAERQAQ..VDVAS......AG..L..GG..T..Q..PQHQR...A..T..LT.........S.EA...TR....QC...PQ..VIVMS 548
Tni_COG1    ....VQ..T..IRD...--T..GPSRVAQY.TDVASY......G..S...G....SQ...QHQR.S.A..T..LT..I......M.A..NIR.E..QH....C.TASATS 546
Tru_COG1    .M..VQ..K..IRD...Q.T..GPSCVAQY.TDVGSY......G..S...G....SQ.A.QRQR.N.A..T..LT..I......M.A..HIR.E..QY....Q.TTSATS 549

Hsa_COG1    VSPT-QAKSSAFDRYADAGTVQEMLRTQSVACIKHIVDCIRAELQSIEEGVQGQQDALNSAKLHSVLFMARLCQSLGELCPHLKQCILGKSESSEKPARE-FRALRKQGK 654
Ptr_COG1    ....-...............................................................................................-......... 654
Ppy_COG1    ....-..........................S....................................................................-......... 654
Mac_COG1    ....-........................................................T......................................-......... 654
```

```
Cja_COG1    ....-.T............A..........V......................R..V.S.V.......................................-......... 654
Mmu_COG1    TT..H.P.N.............D...........SV.G..Q...CT...VTRE.K.V.H.T...A.....................VV.QCGG.......-A...K.... 652
Rno_COG1    TTH.H..T..............D...........SV.G..Q...RV...VT.D.K.V.H.T...A......................V..CGG.......-V...K.... 652
Ocu_COG1    --.PP..N.............RDL..A..AV..GR.TGR.Q...R....A.....AVRSGVQ..A......................V..PG.P......-P...K.AA. 450
Eca_COG1    ...S-..R.C............D..Q.H.....Q..T....V...N..DV......V.SGV...A...................E......G.......D-S...K.... 654
Cfa_COG1    .F.G-....C...............Q.H..V...Y.T...Q.......QV......V..GV...A........................R.G....T..D-P...K.... 654
Bta_COG1    T..A-A.E.C....F...E....T...H.T...RD.T...Q.......QAI.....V.DG.....A.......................R.G.P....QD-S...K.... 652
Mdo_COG1    A.GA-.T.N......S..S..EG...NHCA..TEQ..A.V.....GA.AAL..PTHM.SRP..S....................R......G.....I..-P..TK..A. 649
Tgu_COG1    AV.P----R.....F.......GL..ERC...VQ.LLG.VQEQ...AQ----S.L.PPGD.R.NA.........A.P......QR.V..QAGGA.SAP..-P.SAK.L.. 644
Gga_COG1    LT.PV.PR-.S......TSM.EGL..DHCI...H.VLS.V.E...GAQ------A..PSDTR..A...........S............R.G.V.TALK.-T.STK.L.. 627
Aca_COG1    IQQV-.SRN.....F......E.L..SHCIR.VD.VL..VQG..RHAQ------EMLCTIPR.TLSF......R.................GCPDNVT..-LWP.K.V.. 625
Xtr_COG1    EFV.TA..I......G..S......SHHCLS.MQQ.Q.SVNTT...VK..LVDHGV..F.P..TA...L.......C..............V.T.QSF..-P.STK.T.. 652
Dre_COG1    LEKYTVSS.TPIN.FM..EA.EDI..DHCL..VRDVLASV.S..ANAQ------AETSSHSQ.S...........M.....S........QGGVDVVS.GTP.QSK.L.. 647
Gac_COG1    SG.SES.SA.S.N.FT.SPA.EDA..EGCL..VRL.LSS..S..L.AP-------PD.SP.R.S...........M.....N..H.....QSG..AT.KGTP.QGK.L.. 648
Ola_COG1    TL.PGVPE..S.S.LT.TPA.EAA..DGCA..VQRLLAS..S..AAVS-------PDSAG.R.S...........VC....N..L.....HGE..LS..GIP.QAK.L.. 651
Tni_COG1    SC.AERFSA.S.N.FT.SPA.E.A..EGCL...H..LLS..S..A.AS-------PNPSPSQ.S...........MS....N..H.....RRGL.AALKSVP.QSK.L.. 649
Tru_COG1    SG.AECSLGTS.N.FT.SPA.E.A..EGCLT..H..LSS..S..VNAS-------PNPS.EQ.N..........FMS....N..H.....QRG..AMLQGTP.QSK.L.. 652

Hsa_COG1    VKT-QEIIPTQAKWQEVKEVLLQQSVMGYQVWSSAVVKVLIHGFTQSLLLDDAGSVLATATSWDELEIQEEAESGSSVTSKIRLPAQPSWYVQSFLFSLCQEINRVGGHA 763
Ptr_COG1    ...-.......................................................................................................... 763
Ppy_COG1    ...-.........................R.....................A.......................................................... 763
Mac_COG1    ...-.........................R...T....................................................................V....... 763
Cja_COG1    ...-.........................R...TV...................................................................M....... 763
Mmu_COG1    GRA-.DVL.A..Q..G...........A.R...T.L..F..C...R....R..........N.........T.............T................V....... 761
Rno_COG1    G.A-.DVL.V..Q..............A.R...A.L.....C........S....................T.............T................V....... 761
Ocu_COG1    G.G-.DAA....E....R.M......Q..R...A.L....TQR.A..........................T...G.I..T....T........................ 559
Eca_COG1    G..-...............L......V..R..........A...A..............................N.I.......V........................ 763
Cfa_COG1    G..-R.V..M.........L.........R..........A.......................................T....I........................ 763
Bta_COG1    G.A-..VL....R..D...L...R..T..R..G....R..A...A....V.....I.....................I.......V........................ 761
Mdo_COG1    G.A-..VN.VL........Q..E..LL..RL..M.ITN..VRD.......GN...I..M..N.........T.T...........V..................Q..... 758
Tgu_COG1    G.A-..VS.EL....G..AE.....LVA..L.....T.G.VQC..HT....T.............I.....S.............V....H..CL.......V......T 753
Gga_COG1    G.V-..VN.V........AE.....LAA..I.....T.A.VQC...T....T......A..N...I.........N.........M.......CL..N....V......T 736
Aca_COG1    G..-..VN.IKS....L..R...N.LAA..I..AT.A.I.VQC.A....K.GP..I.....N.E.I..E..T.T....K......V....C...L.......V......T 734
Xtr_COG1    G.K-VDSHAVSC...DL.DK.KK..LEA.SI...F...H.VTS..HT..INT...A.....H...I.....T.A...........G.C.....ML......AV.N..... 761
Dre_COG1    A.A-ADVS.A....SCL..E..SC.MDA.GI....LTR.RL--------------------------------------------------------------------- 687
Gac_COG1    ARATT.AS.S.T..AAL..DF.VC.MEA.RI....LS...LDK.ATA.HAES..AI.T...N.ED...........N........V....F...L..Q..VQV....... 758
Ola_COG1    L..AV.VS.A..E.AGL..E..GC.MEA.RI....LAQ..MKE.ARV.HAES.........N.EDV.....S...T.........V....FL..L..Q..V.V.K..... 761
Tni_COG1    SCAAT.VS.AE...IGL..D..TC.LEA.RI...T.S...LDK.STA.HAES..AF.M...G.ED..........N.........V....F...L..Q..V.V.K..A.. 759
Tru_COG1    SRAVT.VS.AE...IGL..N..SC.LEA.RI...TISNI.LDK.GTA.HTES..AF.T.....ED..........NN.....H..V....F...L..Q..V.V.K..A.. 762

Hsa_COG1    LPKVTLQEMLKSCMVQVVAAYEKLSEEKQIKKEGAFPVTQNRALQLLYDLRYLNIVLTAKGDEVKSGRSKPDSRIEKVTDHLEALIDPFDLDVFTPHLNSNLHRLVQRTS 873
Ptr_COG1    .............................................................................................................. 873
Ppy_COG1    ........................F...................................A................................................. 873
Mac_COG1    .............................S..............................A................................................. 873
Cja_COG1    .....V..................A.......D.T.........................T................................................. 873
Mmu_COG1    ...........T..A..I....Q.T..N.........M...............TM..SS..E........A...M..M.ER.....................N....... 871
Rno_COG1    ...I.......T..A..I....Q.T..N......T..M....................S..E....S...S......MA.R.....................N....... 871
Ocu_COG1    ..........R...AH......Q.AG.Q.R.......................SL......E.G................Y..G..................T....... 669
Eca_COG1    ................I.......P....M.......M.......................E........Q...F...A.Y..G..................N....... 873
Cfa_COG1    ........................A...HV.......M.....................RTE........Q.......A.Y.....................N....... 873
Bta_COG1    .............LGR.A....R...A..L.......M...............SV......E.M....CRQ...V...A.Y..T..............H...N....... 871
Mdo_COG1    ...A.......G.L...L.G......D.PA....T..M....................V.TE....A...Q.........F..G..................N....... 868
Tgu_COG1    ........L.R...AE.L...G..V.Q..E.RPDS..L..S...........VS.I.DS.SEDT.P..I.Q.C.....I.F..GH.................N....... 863
Gga_COG1    ........L..A..AE.L......MD...D..A.T..M.................I....SE.A.TS.I.H.........F..GH.................N....... 846
Aca_COG1    ........L..T..TE.L.G......V..E.VC-V.WMN----YYM.F.FS.S.SKNVFENQK.RNSEVTENEM...H..F..SY.................N....... 839
Xtr_COG1    ..R.....L.RH.LEE..G...S.CHKSLD..Q-.SSL..A......F....M.LI.SSRSEDL..SK..QE...Q..A.Q..TC..............A..N.YA.... 870
Dre_COG1    ------------------------------------------------------------------------------------------.KI.Q--------------- 692
```

```
Gac_COG1    ..RP....L.HT.LN..LHH.QT.TQQAPSRQ..V..M.........F.....HTI.GSRVE.G.GS..HQ.P.FHE.C.W..GY...........P..T..N..S....  868
Ola_COG1    .LQP....L.QA.LGRALEQ.NE.T.RQRHGEQDV..M.......M.F...F.HST.SSRLE.GR.S.PPQ.R.LIQIC.W..SF...........S..A..T..T....  871
Tni_COG1    ..RP....L.QA.LN.ALHQ.HSFLQQ-P.N.D....M.........F...F.HTT.SS.LE.S..T..QQ.P.FHE.CER..SF...........P..A..N..S....  868
Tru_COG1    ..RP....L.QA.LN.ALQK.QS.IQQ-PRD.DAV..M.........F...F.YNT.GSRLE.G..T..QQ.P.FHE.CEW..SF...........P..A..N..S....  871

Hsa_COG1    VLFGLVTGTENQLAPRSSTFNSQEPHNILPLASSQIRFGLLPLSMTSTRKAKST-RNIETKAQVVPPARSTAGDPT-VPGSLFRQLVSEEDNTSAPSLFKLGWLSSMTK  980
Ptr_COG1    ......................................................-.....................-................................  980
Ppy_COG1    ......................................................-.....................-.........I......................  980
Mac_COG1    ......................................................-..V..........H.......-................................  980
Cja_COG1    .................N................................P...-..........S..C.......-L........I.....S........A.......  980
Mmu_COG1    ...........F.S.......................................RA.S.SV..Q...G...L.RV....TH........A....DSP.......A.......  980
Rno_COG1    ...........F.S.......................................RA.G.G..SQT..G...L.RV....AH........A....GSP.......A.......  980
Ocu_COG1    ...........FTS..NAV....A......................N....L.-.SVQ....AD...L.R.E..MTHA...........D.................  777
Eca_COG1    ........P...FT...............................T...S.S......A....L.R.D..M-Q...........EDS.T...............  981
Cfa_COG1    ..........HFT...C............................................S.SM..........L.R.....-H...........EDA.T...............  981
Bta_COG1    ........P...FT..........A............................AGGSMD....AA...L.R.D..R-QA........AD.EDSA...............  979
Mdo_COG1    .....L...D..FSA.........................T.........SS.ST.S.D...L.TLPR.D.VMMR.......A.T..ED..T..S.T.S..AN...  977
Tgu_COG1    .....L......YTS.GG.LS...L......S..............S.S..S...A.ST.-RV..A.ARAPA--EEAAR........IADDED.A............G...  969
Gga_COG1    .....L......Y.S..GALG...L.....................S.S..T..AT..T.-RVE.P...LTR.E.EALR.........T..ED..............G...  954
Aca_COG1    .....LM.S...YSS....LT...L......T.T............S.S.TT..AL..S.H...TPTT.LMR-.EE.FR.....K..AA...D.AT...............  947
Xtr_COG1    .....L..A...FIT....VGL..T..V..................S.S..T.GR-AEED..I..TLSEVPPSEEEGFR........ATQDEEAPSQ.............  978
Dre_COG1    -------------------------------------------------------------------------------------------------------------  692
Gac_COG1    ..L..L..S.K.F.....SG.....Y.........T........TS.V..S..AP.SCDAAL.LAT.SSLAD.EDSFR........ADQDGD.ATQ.....S...G.A.  977
Ola_COG1    ..L..LM.P.K.FSA...PV..S..Y.........L.........SNE..P.AASAAPQKASPQARQSS.ATM.DAFR..N.....AEKDEDS.SS...R.....G.A.  980
Tni_COG1    ..L..L..S.K.FSS...SV.....Y...................SNV..P..SS.VSSH--LPT..SAKAEIADSLQ.........DQDEESAS......S...G...  975
Tru_COG1    ..L..L..S.K.FSS...SIH...NY...................SNLH.S..SS.ASSH--.LT..STKADNPDRFQ........ADQDEDSAS......S...G.S.  978
```

```
Hsa_COG2    MEKSRMNLPKGPDTLCFDKDEFMKEDFDVDHFVSDCRKRVQLEELRDDLELYYKLLKTAMVELINKDYADFVNLSTNLVGMDKALNQLSVPLGQLREEVLSLRSSVSEGI 110
Ptr_COG2    .............................................................................................................. 110
Ppy_COG2    .............................................................................................................. 110
Mac_COG2    .............................................................................................................. 110
Cja_COG2    .............................................................................................................. 110
Mmu_COG2    ...R.....T.........................................................................R........................... 110
Rno_COG2    ...R.....T.................................................................................................... 110
Ocu_COG2    ......................L....................................................................................... 110
Eca_COG2    ..Q......R................................................................................................I... 110
Cfa_COG2    ----------------------........................G............................................................... 88
Bta_COG2    ...........................................A.........................................S........................ 110
Mdo_COG2    ...K....................D..........................................................................M..K.T..D.. 110
Tgu_COG2    -------M.RE.---S........A.....................E....................................................M..K.C..... 100
Gga_COG2    ..ARK....R..EN..........P.........E...........E....................................................M....C..... 110
Aca_COG2    ..ARS....R..GH........L.PN........E................................................................M..K.C..... 110
Xtr_COG2    ..RVT.Q..Q.SPSM.........DG....V...E........T.........R...............................S.................KT..N.V. 110
Dre_COG2    ----M.T...A..S..........D.....K..A..........M.E...Q..R....................................I...................N.V. 106
Gac_COG2    LTEFN.........S......V...D.....Q...E...Q..M..M.E...................................................M....C...V. 110
Ola_COG2    ILQ.K.........S......V...D.....Q..AE...Q.....M.E...................................................M....C...V. 110
Tni_COG2    VLS.S.......ES......V.IRD.....Q..AE...Q.......E..........................................................C..GV. 110
Tru_COG2    ---.G.......ES......I.IRD.....Q..AE...Q.....M.E..........................................................C..GV. 107

Hsa_COG2    RAVDERMSKQEDIRKKKMCVLRLIQVIRSVEKIEKILNSQSSKETSALEASSPLLTGQILERIATEFNQLQFHAVQSKGMPLLDKVRPRIAGITAMLQQSLEGLLLEGLQ 220
Ptr_COG2    .............................................................................................................. 220
Ppy_COG2    .............................................................................................................. 220
Mac_COG2    ......V....................................................................................................... 220
Cja_COG2    .......C...................................................................................................... 220
Mmu_COG2    L.................S........................DV.SQ..........V................................................... 220
Rno_COG2    L..E......D.......S........................DA.SQ..........V................................................... 220
Ocu_COG2    ........................................N............................................I........................ 220
Eca_COG2    .............................................................................................................. 220
Cfa_COG2    Q......C................................N.....T.......W.......................................................R 198
Bta_COG2    ......L..........I......................H......................................L.............................. 220
Mdo_COG2    ..............R.........................N.....S............................................................... 220
Tgu_COG2    Q...D..T......R..I......H..Q..........H..GT..L.S..GN.......V................................................... 210
Gga_COG2    Q.I.D.L..........V......H..Q..........H..GTN.L.T..GN.......V................................................... 220
Aca_COG2    H.I.D.L.......R............K..........H..N...S.ST.V........V...V......................LK...V.................. 220
Xtr_COG2    Q...S.LA....V.R......TMME..H..........H..N....-...VK..H..............................N.........G............... 219
Dre_COG2    E.I.TQL...D..Q...L........V...........H-.NT.D.TS..T.....A..................................................IQ... 215
Gac_COG2    Q.I.NQLA....LQ...V........V...........H.......SNS..T....A.H....................................S.......V.I.... 220
Ola_COG2    Q.I.NEL...D..Q...D........V...........H..N...SNS..L....A.......................................S..........I.... 220
Tni_COG2    Q.I.DQLI.H..LQH..V..M.....V...........H......DSGS..-N....A...........H.....................................I...H 219
Tru_COG2    Q.I.DQLF.H..LQ...V..M.....V...........H.......SGSQ.-N.S..A.......................I.............S..........I.... 216

Hsa_COG2    TSDVDIIRHCLRTYATIDKTRDAEALVGQVLVKPYIDEVIIEQFVESHPNGLQVMYNKLLEFVPHHCRLLREVTGGAISSEKGNTVPGYDFLVNSVWPQIVQGLEEKLPS 330
Ptr_COG2    .............................................................................................................. 330
Ppy_COG2    .............................................................................................................. 330
Mac_COG2    .............................................................................................................. 330
Cja_COG2    .............................................................................................................. 330
Mmu_COG2    ......V.............Q..............VN...V........SS..L.........................V.....TI.............E..R........ 330
Rno_COG2    ..N................................VS...V.RV.D...S...L.........................V.....TI.............E..G........ 330
Ocu_COG2    ..N...V.................................V.H......S...M..D...........................I..........L..E........... 330
Eca_COG2    ...................................V.......I.........I.............C...............................E..R........ 330
Cfa_COG2    ..N................................M....V..I.........I.............................................E..R........ 308
Bta_COG2    ..S................................V....L..I.D..LSD..L..D..........................................E..R........ 330
Mdo_COG2    ..N.................................E...V....Q..K....T.............................................E..C........ 330
Tgu_COG2    ..N................................V...MV..Y.Q.......A...R.........................ADI.............E..R........ 320
```

```
Gga_COG2   ..N.....................................V..Y.QA......A............................ADI.............E..R........ 330
Aca_COG2   ..NI........................E......V....V..Y.Q.......T...R......L...........S.P...................EF.R...V.... 330
Xtr_COG2   ..N..TV............................V....N..Y.Q...G..HA..S....................V...NTD...........I..E.AR........ 329
Dre_COG2   ..NI..V............................M....V....K.S....K...A.......................D.ADI.............E.IR.V..RV.. 325
Gac_COG2   ..N..MV...............V............M.Q..T.EA.K.S.....L..SR......................D.AD..............EMIK.I..R.AY 330
Ola_COG2   ..N..MV............................M.Q....EA.K.S.....L..SR......L...........T...D.AD..............EMIK.I..R.AY 330
Tni_COG2   .CN.EMV............................M.Q..V.DA.QAS....EL..LR...................V..GRADA.............EM.RAI..R.SF 329
Tru_COG2   SCN...V............................M.Q..V.DV.T.S....EK..SR..................VVC.DRAD..............EMI.AI..R.SY 326

Hsa_COG2   LFNPGNPDAFHEKYTISMDFVRRLERQCGSQASVKRLRAHPAYHSFNKKWNLPVYFQIRFREIAGSLEAALTDVLEDAPAESPYCLLASHRTWSSLRRCWSDEMFLPLLV 440
Ptr_COG2   .............................................................................................................. 440
Ppy_COG2   .......................F.......................N.............................................................. 440
Mac_COG2   .......................F.......................N.....................T.......................N................ 440
Cja_COG2   .......................F.......................N.............................................................. 440
Mmu_COG2   .....D.....Q...V.....Q.F....................N.SN..............V..........G......G............I..GK...........A 440
Rno_COG2   .....D.....Q...V.......F......................SN..............V......S...G......G............I..SK...........A 440
Ocu_COG2   ....................L..F.E.....................N................................G................K.....L...... 440
Eca_COG2   ...............V......TF.......................N....H................S..........G.S.............Q......L...... 440
Cfa_COG2   ......................AF..........R............N................................G.SF............QK.........A.A 418
Bta_COG2   .....D.....Q..........TF.......................N.........................I......G...R...........Q......K.....A 440
Mdo_COG2   ........S..............F..............S........N.......................I.G..E...G.SF......MA.V..MK.....Q.....A 440
Tgu_COG2   ........V......T......KF.................S.....N.................A..E..S.R..E...G.SF....T.MV....VK.....Q.....A 430
Gga_COG2   ........V......T......KF..........R...S..S.....N.................A.....S.S..E..EG.S.....T.MV.M..LK.....Q.....A 440
Aca_COG2   ......................KF..............L..S.Q...D................EA..S..SEE.KE..DG...H...A.MV.E..QK...AQI...... 440
Xtr_COG2   ........V.FQ...T......KF..............S..S.....N.........L..K....N.....LHGH.E.....N...R.T.IL..C.LS...N.V...K.A 439
Dre_COG2   ........V.Y.R..V......KF.................S.Q..HN.........L..K.......S.IA.G..AT.VG.SFH.QVTEVL..CVC...T.QVY..P.A 435
Gac_COG2   ........I.Y.R.SA.VE....F....S.........V..S.T..HN.........L.YK.......N.IS.G..A...G.V.H.QV.EVL..C.M.....KIY.SP.A 440
Ola_COG2   I.......I.F.R.SAT.E....F....S.........V..S.T..HN.........L.YK....R..NSIS.G..A...G.T.H.QV.EVL..C.LK..A.KVY..P.T 440
Tni_COG2   ........V.Y-----AVGGRL.PD.ASAALS.TRP.SPP.PLS--------LLFWVY.YK.T..G..K.IS.G.QA...GGTFH.QV.EVL..C.V.....GVY.AP.A 426
Tru_COG2   .......EV.Y.R.S...E...KF....S...........VS.A..HNR........L.YKD...H..KTISEG.QA..VG.MFH.QV.EVL.FC.M.....RVY..P.A 436

Hsa_COG2   HRLWRLTLQILARYSVFVNELSLRPISNESPKEIKKPLVTGSKE-PSITQGN-TEDQGSGPS-ETKPVVSISRTQLVYVVADLDKLQEQLPELLEIIKPKLEMIGFKNFS 547
Ptr_COG2   ............................................-.......-.........-............................................... 547
Ppy_COG2   ..................S.........................-...A...-.......S.-................................T.............. 547
Mac_COG2   .....F............S.........................-.......-.........-.............L................................. 547
Cja_COG2   .......................K......L.............-.TV.P..-.......Q.-....M.C.......C................................ 547
Mmu_COG2   .............F....S...V..V....A..T....-....D-.-------S.....HA.-.A-SAA...S.......S..GR...W..D...TV.Q........... 539
Rno_COG2   .............F....S...V......TA..T....-A...D-.-------SD.H..H..-.A-GA.P..S.......S..GR...W..D...TVRL....V...... 539
Ocu_COG2   ..................S..........TA..S....I...RD-..TSP..-S..P..SAA-D........S...I......HT.....................S... 547
Eca_COG2   .H.............M.....L........A.DT......SN.D-.......FS........-.........SI.....................TV............. 548
Cfa_COG2   ...............M..S..L........T.D..........D-..V....-G........-.........S......................T.............. 525
Bta_COG2   ...............I..K..L........A.DV.........D-L......-C...A....-....I..V.S.......T..HR..........T.......VM..... 547
Mdo_COG2   ....K..........M.I...L...L...IA.DN..SSINTN..SS..S..T-N....N.L.-..QT.AF..N...I..........................V...... 548
Tgu_COG2   ....K.S..V.......IS.V.V....S.NT..S..TVPV.R..-S.LSLNP-S....NES.P.SL.LS...S...I..A.......DRI.DI.DM............I. 538
Gga_COG2   ....K.S....S.....I..V.V....SDNT..S..LMPA.R..-S.VNLSS-N....N.S.P.SQSLP...S......A........KI.DI..M............L. 548
Aca_COG2   ....K.S...V..........L........T.DHR.T.AGSN.D-SPLNL.S-V....I.SAPD.Q.LP...T....L.A....H..DW...........F.SV...DIA 548
Xtr_COG2   ..F.K....MIS..CS.IK.VA.HTC.-DASNNEM.NHPSNYRDSVTMSVAG-ND..D..STSD.RVT.K..TR..MLF...A..FKL.....R...IV..QQ.....I. 547
Dre_COG2   ..F.K....LIS...T.LT.VLTKTS.T.AS.DSVR..PSSASSTS.R.SQD-ADSETG..T-------VL.TK...FIA..V....GKI.DIS.M..A...N...Q..A 537
Gac_COG2   ..F.K....LYS..AK.LD.VLTKTP.P.AT..PTR..PSSASSTS.R.SMEEGGSESGS.A-------.L.TK....IA..IQ.....ITDVTDMVRQR..A...N.IA 543
Ola_COG2   ..F.K....LFS..AR.LD.VLTKSPPV.G...QAR..PSSASSTS.R.SMDDGGSESGS.A-------VL.TK....IA..VQ.....I.Q.S.MVRQR..A....... 543
Tni_COG2   ....K....LY...AR.ID.VLTK.STL.VT..PSR.PPSSASSTS.R.SVDEGGSE.G..V-------GL.TK...FIA..IQ....KGA....VVRRR.DA......A 529
Tru_COG2   ....K....L....AK.LD.VLTK.SVM.LT..PCR..PSSASSTS.R.SVDEGGSE.G..I-------.L.TK....IA..IQ....KMSD.S.VVRRR..A.A....A 539

Hsa_COG2   SISAALEDSQSSFSACVPSLSSKIIQDLSDSCFGFLKSALEVPRLYRRTNKEVPTTASSYVDSALKPLFQLQSGHKDKLKQAIIQQWLEGTLSESTHKYYETVSDVLNSV 657
Ptr_COG2   ............L................................................................................................. 657
Ppy_COG2   ............L....................S.......................................R................A................... 657
Mac_COG2   ..T.........L....................S......................................N.................A................... 657
```

Cja_COG2	............L....................S..P.........A...................	657
Mmu_COG2	...........AL..H..A...R.V....E...SY...................S.............Y......G..VQP.VM.S..QEA..D...R.F..........	649
Rno_COG2	...........AL..H..A...R.V....E....Y...................S.............Y......G..VQP.VM.R..QEA..D.....F.......S..	649
Ocu_COG2	............L...I.P..........E...RY....................A...........FH...R.......H.V.......A...................	657
Eca_COG2	..........L.L...L....NR......E..LSY..............................R.FY.............R.R.....A..A................	658
Cfa_COG2	..........T.L.....A..NR......E...SY................................FY.....................A................S..	635
Bta_COG2	..........L.L.....A..E..T....E...SH...................V.............Y....R........T.R....A....................	657
Mdo_COG2	...E.....RI.L.SSM....NR......E...SY.................A..K..P........FY....E.......S..H.....A................S..	658
Tgu_COG2	C.AG.....KT.L.....T.NNR......E.S.AY....................K..P........FYR..NDYR.S...PM.H.....A.....Q.ARDFLAKK.L.-	647
Gga_COG2	C.EG.....KT.L...I.T.NNR......E.S.TY......I.............K...........FYR..NEYRNI...PM.H.....A.....Q..........S..	658
Aca_COG2	C..G.....KD.LLG.W.T.NKN..........SC......I.............K.LP........FY..KNEYR.T..P...D.....A..G..Q..F..I..I....	658
Xtr_COG2	M.TG....THQLL.PSLLAIKN..VF...E...SN.................M..K......N.V.......NEYQNI.T.S.REE..TDV...C.P...D.I....S..	657
Dre_COG2	IV.E..Q..ST.L.S...T.N.RMT.H.TERSVR...N.S...............R..A.M.N..R..H..VTDS.NVV.DS...E..QV...DC..R.F..I....S..	647
Gac_COG2	IVED..T..KNCL.SS..T.NTRMT.H.TER.CR.....S...........DP.VR..A.M.N..R..H..LTDSTGLVAPSTA.E..RVA.TDY.QR....I.E..S..	653
Ola_COG2	VVED..A..K.CL.SSI...NTRMT.H.TER.CR.....S...........--SV.EHYNHQHRVNMVAR.TMQSCEWCCLSARRSK.QH.VCKP.ST....I.E..S..	651
Tni_COG2	VVEE..S..RARLDTS....N.RMS.H.TER.CR.....A..............VR..A.M.N..Q..H..L.DSAGLVSSSTV-...RVA...C..R.C..I.E..S..	638
Tru_COG2	VVD...F..RARL.SS....N.RMS.H.TER.CR.....A..............VL..A.M.N..R..H..LTDSMGLVTPSTTHE..HVA...C.QR....I.E..S..	649

Hsa_COG2	KKMEESLKRLKQARKTTPANPVGPSG-GMSDDDKIRLQLALDVEYLGEQIQKLGLQASDIKSFSALAELVAAAKDQATAEQP	738
Ptr_COG2	..........................-....................................P..............T...	738
Ppy_COG2	..........................-.................N..................P..................	738
Mac_COG2	..................T.....-.-....................................P..................	737
Cja_COG2	..........................-.............................T......P..TG..........T...	738
Mmu_COG2	..............RSPAT...SS..G.................H......RM...T......P..M...L..R...A....	731
Rno_COG2	R.............RSPAT...SS..-.................H.......M...T......P..T...L..R...A....	730
Ocu_COG2	R.................TT......-.........................M...T.S....P..S...V......A....	738
Eca_COG2	.......................S..S.........................M...TK.....V..T...V...........	740
Cfa_COG2	R..............S..T.......G.........................M..ATK.....P................--	715
Bta_COG2	R.............R....S.A.L..G.........R......A......E.M..RTK..R..P..................	739
Mdo_COG2	................ATT..I.TN.-.....N...........S....M..MD..T.N....T..T...I......M....	739
Tgu_COG2	--ATS.R.M..ET.S...........LT........PS	683
Gga_COG2	..............R.AAL....TN.-..................F....R.M..ETNN.......T...LT.........S	739
Aca_COG2	................VTS.S.S.N.-.....N............F.I.M......LNN.....G.S...Q..R..IS...T	739
Xtr_COG2	................VTSTNTTNA--.N...N............F.D.VE.....KH....LQT.T...YT..E..NT.--	735
Dre_COG2	R................TTST..VNA-.P...S.................ENM...P...TM..S.L...QE.R.L.ST..M	728
Gac_COG2	R..............GASTTTAAGANG.PT..S...................M...QR..PM..T.MD..KE.REL.EQG--	733
Ola_COG2	R..............GAA.TTTTGANG.PT..T...................M.F.PE..SM..T.MD..KE.REL.EQN--	731
Tni_COG2	R..............GAATAATAGANG.PT..G...................M...PA..SM..T.MD..RE.REL.EQN--	718
Tru_COG2	R..............GATTAATAGANG.PT..G...................M...PG..SM..T.MD.CSS..H.SGCQM.	731

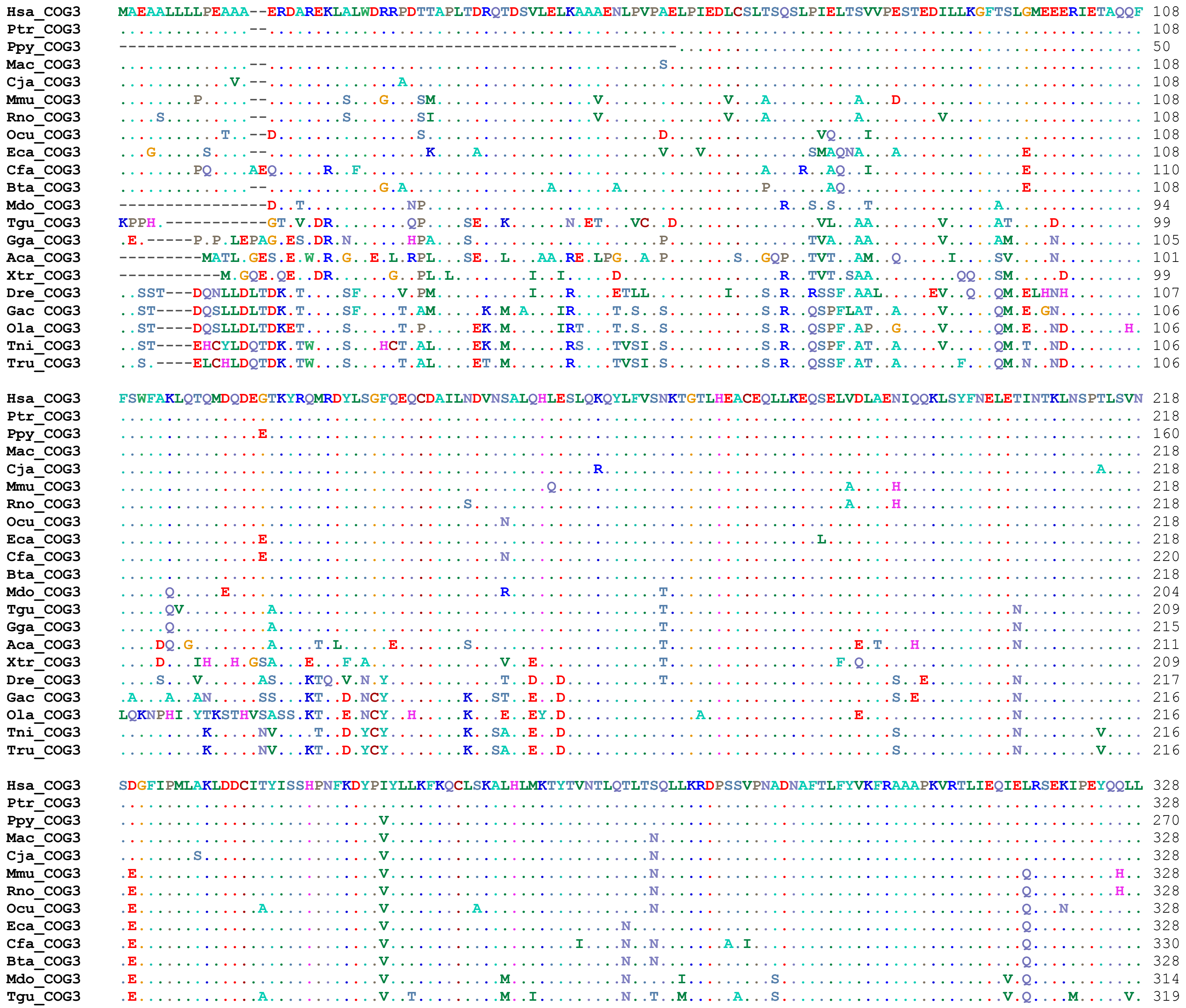
Hsa_COG3 108
Ptr_COG3 108
Ppy_COG3 50
Mac_COG3 108
Cja_COG3 108
Mmu_COG3 108
Rno_COG3 108
Ocu_COG3 108
Eca_COG3 108
Cfa_COG3 110
Bta_COG3 108
Mdo_COG3 94
Tgu_COG3 99
Gga_COG3 105
Aca_COG3 101
Xtr_COG3 99
Dre_COG3 107
Gac_COG3 106
Ola_COG3 106
Tni_COG3 106
Tru_COG3 106
Hsa_COG3 218
Ptr_COG3 218
Ppy_COG3 160
Mac_COG3 218
Cja_COG3 218
Mmu_COG3 218
Rno_COG3 218
Ocu_COG3 218
Eca_COG3 218
Cfa_COG3 220
Bta_COG3 218
Mdo_COG3 204
Tgu_COG3 209
Gga_COG3 215
Aca_COG3 211
Xtr_COG3 209
Dre_COG3 217
Gac_COG3 216
Ola_COG3 216
Tni_COG3 216
Tru_COG3 216
Hsa_COG3 328
Ptr_COG3 328
Ppy_COG3 270
Mac_COG3 328
Cja_COG3 328
Mmu_COG3 328
Rno_COG3 328
Ocu_COG3 328
Eca_COG3 328
Cfa_COG3 330
Bta_COG3 328
Mdo_COG3 314
Tgu_COG3 319

```
Gga_COG3     .E.............A............V..T......L..M..I.........N.....M.....A...S........................V.Q....M.....V. 325
Aca_COG3     ...............A......D.....V..A..R......M........H...H.....I.....VP..S..........................Q....M....... 321
Xtr_COG3     .E......T..........A...Y....V.MA......Y..M....N.....M.N.....I...S.NASTT........................V.Q............ 319
Dre_COG3     .E..V...S...E..E.V..........V..T.........MQ.I.SH......N..G..S....LGA.........Y............R....V.Q........H... 327
Gac_COG3     .E..V...S......E.V..........V..A.......R.M.F..IHI...M.H.....T....MGLT........Y...Y........S......Q.A..V...H... 326
Ola_COG3     .E......S......E.V....S.....V..A.........M....V.I...M.S.....T....MGLT........Y............S....V.Q.....T..S... 326
Tni_COG3     .E......S......E.V..........V..T.........M.F..VHI...M.N.....I....MNLTS.......Y...Y........S......E.A......HR.. 326
Tru_COG3     GE......S......E.V.A........V..A.........M.F..VHIT..M.N..T..I....MNLT........Y...Y........S......Q.A....Q.H... 326

Hsa_COG3     NDIHQCYLDQRELLLGPSIACTVAELTSQNNRDHCALVRSGCAFMVHVCQDEHQLYNEFFTKPTSKLDELLEKLCVSLYDVFRPLIIHVIHLETLSELCGILKNEVLEDH 438
Ptr_COG3     .............................................................................................................. 438
Ppy_COG3     .....................................................................-........................................ 379
Mac_COG3     .............................................................................................................. 438
Cja_COG3     S.........................S................................................................................... 438
Mmu_COG3     ....................Y..T...................................................................................... 438
Rno_COG3     ....................Y..T...................................................................................... 438
Ocu_COG3     S......................T...................................................................................... 438
Eca_COG3     ...................T...T.............I..........................P............................................. 438
Cfa_COG3     ...................T...T.............I........................................................................ 440
Bta_COG3     T..................T...T.............I........................................................................ 438
Mdo_COG3     .E.................TS..T...................................................................................... 424
Tgu_COG3     .E..................S..T........................................P..........L.....L..M....................M.... 429
Gga_COG3     .E.................SS..T........................................P..........L.....L..M....................M.... 435
Aca_COG3     .E..........H......TS..T...NR...........................D..................L.............................M.... 431
Xtr_COG3     SE..........S..S...TT.ITD............................H..........P...V......L.............V.............I.M.... 429
Dre_COG3     EE..........V..S...NS.ITD......K............................S..............L.....L.......V...............M.... 437
Gac_COG3     D..............S...TS.ITD..K..SK............................S...P..E.......L.....L.......V...............M.... 436
Ola_COG3     DE......T...Q..S.C.TS.ITD..N..SK.........................D..S..............L.....L......IV...............M.... 436
Tni_COG3     DE..........Q..R...TS.ITD..N..CK............................S.A............L.....L......I..........S.....M.... 436
Tru_COG3     DE..H.......Q..R...TS.ITD..N..SK........................H...S...P..........L.....L......I..........S.....M.... 436

Hsa_COG3     VQNNAEQLGAFAAGVKQMLEDVQERLVYRTHIYIQTDITGYKPAPGDLAYPDKLVMMEQIAQSLKDEQKKV-PSEASFSDVHLEEGESNSLTKSGSTESLNPRPQTTISP 547
Ptr_COG3     .......................................................................-...................................... 547
Ppy_COG3     ..........................................................----------------------------........................ 461
Mac_COG3     .......................................................................-...................................... 547
Cja_COG3     ......................................................A................-.........Q....G.................H..... 547
Mmu_COG3     ..H...................................................................A-.........R.....ASG.R.....D............ 547
Rno_COG3     ......................................................................T-.........R......S..R.....D............ 547
Ocu_COG3     ...................................................E...................-.........Q...A.A...................... 547
Eca_COG3     .......................................................................-....A....R...A...N......PD.P.......... 547
Cfa_COG3     .............................................................R.........-.........R...T...N.................... 549
Bta_COG3     ......................................................................LS.........R...A.P........S......Q...... 548
Mdo_COG3     ......................................A...............................L-........IRGHS..EQGIEVLD.R.P........... 533
Tgu_COG3     ......................................................E...K......E....L-.........R..DP..C..V....A......H.S.... 538
Gga_COG3     ......................................I...............E..........E....L-.........R..DP..CN.V....A......Q.S.... 544
Aca_COG3     ........A.....................Y.......A.....S......A..E............E..L-.AD.----MKVDDPKTYNVV.LDTW..S...L...... 536
Xtr_COG3     ...SV.H......V.....................S..L...............E...K....I.E..QRLHST.S........DPD..N.I...AS...SQK..N.V.. 539
Dre_COG3     ....VV..A..D.V........................I...............E...K......E..M.L-N.SN.....Q...S.--NKKLIS.----DT.L.SSV.. 540
Gac_COG3     .H..--.....DTV....................H...I..N.........E..E...R......E..M.QMSQ.SM....Q..NPD--GRRN.NAGNVEAS.L..S... 542
Ola_COG3     .R...S.....DTM........................I..N.........E..E...K.S....E..M.QMSQ.S.....Q..D.G--VRRNNSAGSLEVS.L..S... 544
Tni_COG3     ....TA.....D.V.....................................E..E...R......E..M.--SQ.ST...IR..DPT--GRRN.NPANVEASS..AS... 542
Tru_COG3     .....A.....D.V........................L............E..E...R......E..M.--SQ.ST...IQ..DPT--GRRS.NAANTEASSL.AS... 542

Hsa_COG3     ADLHGMWYPTVRRTLVCLSKLYRCIDRAVFQGLSQEALSACIQSLLGASESISKNKTQIDGQLFLIKHLLILREQIAPFHTEFTIKEISLDLKKTRDAAFKILNPMTVPR 657
Ptr_COG3     .............................................................................................................. 657
Ppy_COG3     .............................................................................................................. 571
Mac_COG3     .............................................................................................................. 657
```

```
Cja_COG3   .............................................................................................................. 657
Mmu_COG3   .............................................................................................................. 657
Rno_COG3   .............................................................................................................. 657
Ocu_COG3   ..........................................................V..................................R................ 657
Eca_COG3   ..........................................H................................................................... 657
Cfa_COG3   .............................................................................................................. 659
Bta_COG3   .-..................CFKFHQ.................................................................................... 657
Mdo_COG3   .................................................D..T.....V................................................... 643
Tgu_COG3   ................................................AD........V......................D.......................KA.S. 648
Gga_COG3   .........................................VH.....ADA.......V......................D.......................KA.S. 654
Aca_COG3   ..........................K...................A.A...T........................................................H 646
Xtr_COG3   ..................................................A.T..................................................H.R.... 649
Dre_COG3   ...........................T..................H..DI.Q..................M........AD.A.....................KA..N 650
Gac_COG3   ..............................................K..DI.L..................V.........D.S.....................KA..K 652
Ola_COG3   ..............................................K...I.I.S...V............M.........D.A.....................KA..K 654
Tni_COG3   ..............................................K..DI.L..................M.........D.A.....................KA..K 652
Tru_COG3   ........................M.....................K..DV.L..................M.........D.A.....................KA..K 652

Hsa_COG3   FFRLNSNNALIEFLLEGTPEIREHYLDSKKDVDRHLKSACEQFIQQQTKLFVEQLEEFMTKVSALKTMASQGGPKYTLSQQPWAQPAKVSDLAATAYKTIKTKLPVTLRS 767
Ptr_COG3   .........................................................................................N.................... 767
Ppy_COG3   .........................................................................................N...............L.... 681
Mac_COG3   ............................................E.......D..............................................L.......... 767
Cja_COG3   ..................................................................A...........................A..........A.... 767
Mmu_COG3   ................................................R........................................N..V............L.... 767
Rno_COG3   ................................................R....P...................................N..V............Q.... 767
Ocu_COG3   .........................................................................................G..V........A...L.... 767
Eca_COG3   .........................................................................................GE...G...KV.AQ..L.... 767
Cfa_COG3   ............................................................................................VTSV.....A...L.... 769
Bta_COG3   ................................................R..I.P....L.................................V.S......A...L.... 767
Mdo_COG3   ...................................................I.......S.........N................V..N..VSG..........L.... 753
Tgu_COG3   ...........Q.............I.......................Q..D.........A......T....N.S...........IN..VSST.........S.... 758
Gga_COG3   ...........Q.............I...........A...........Q.I..........A...A..T......S...........IN.MVSST........QS.... 764
Aca_COG3   ....S.S....Q.............I...........F...........QL..P....LA.......V.T.K....N...........I...VSST.........S..Q. 756
Xtr_COG3   ..........L....Q.....K..FI...........A.........S.I...P..D.....T.............S.........V.IN..VSST...M.........G 759
Dre_COG3   ......H..IL..........K...I...........LS..S......LM..GN.DQ.LSR.G......V....T.N...........IN.VVMST.RVL.N...S..Q. 760
Gac_COG3   ......H..IL..........K...I...........FS.........QI..GN....L...A......I....T.S.........-.IN.IVT.T.RVM.S...G..Q. 761
Ola_COG3   ......H..IL..........K...I...........FS.........QI..GN....L...A...K..TE...T.N...........IN.IVMAT.RVM.S...S..Q. 764
Tni_COG3   ......H..IL..........K...I...........FS.........QI..GN....L.R.A......IE...T.R...........IN..VTAT.RVM.S...S..Q. 762
Tru_COG3   ......H..IL..........K...I...........FS.........QI..GN....L.R.A......IE...T.R.A.........IN..VTAT.RVM.SR..S..Q. 762

Hsa_COG3   MSLYLSNKDTEFILFKPVRNNIQQVFQKFHALLKEEFSPEDIQIIACPSMEQLSLLLSVSK 828
Ptr_COG3   ...................VS.R-------------------------------------- 790
Ppy_COG3   ............................................................. 742
Mac_COG3   .....C....................................................... 828
Cja_COG3   .......R...............R......V..........V................... 828
Mmu_COG3   .A....................................S..............N....... 828
Rno_COG3   .................................................V...N....... 828
Ocu_COG3   .....................V......L............M...S..T....N...M... 828
Eca_COG3   ..............................V..........T...........NF...... 828
Cfa_COG3   ..............................V..........V...........N.....C. 830
Bta_COG3   ..............................V..........V...........N....... 828
Mdo_COG3   ........................A...L.........S.............VN....... 814
Tgu_COG3   ...........L............M...L.........N..L..........V-------- 811
Gga_COG3   ........................M...L.........N..L..........V....ANAT 825
Aca_COG3   .....A............K.........L....N..Y.-..L........----------- 805
Xtr_COG3   .....A.............S........I.M......AS..L..........VN.....T. 820
Dre_COG3   .T...A..................A...L....Q..Y.G..L..........IN.....K- 820
```

```
Gac COG3     .....A.R...................RL..S.Q..YGG..L..........IN.....N. 822
Ola_COG3     L....A.R...................RL....Q..Y.G..L..........IN.....N. 825
Tni_COG3     .....A.R...........----------------------------------------- 781
Tru_COG3     .....A.R..................RRL....V..Y.G..L.......T..IN.....N. 823


Hsa_COG4     MADLDSPPKLSGVQQPSEGVGG-GRCSEISAELIRSLTELQELEAVYERLCGEEKVVERELDALLEQQNTIESKMVTLHRMGPNLQLIEGDAKQLAGMITFTCNLAENVS 109
Ptr_COG4     ..............P.......-...........................................................---------------------------- 81
Ppy_COG4     ...F..........P.......-.....................T................................................................. 109
Mac_COG4     ..............P.......-..............................................S............RS.HM.---------------------- 87
Cja_COG4     ....E.L.......P.......-....................................................................................... 109
Mmu_COG4     ..EVE..L....APP.P.....-.H.....T........................A..K........................S...................S...... 109
Rno_COG4     ...VE.......APP.P.....-.H.....T...........................K................................................... 109
Ocu_COG4     ....E........P..C.V...-.C.....T.....................................S......................................... 109
Eca_COG4     .............PP.H.....-.......I............................................................................... 109
Cfa_COG4     .......Q.....SL.-..M..-.......T............................................................................... 108
Bta_COG4     .......Q.....PR.PD....-.S.....T.......D....................................................................... 109
Mdo_COG4     ...AN..I..T..AAAAAEGK.G.C...L......................S......E.........S......I..Q........................S...... 110
Tgu_COG4     ------------------------------M.R.Q...D.AD...A.S...E.....QE...S.....S...N...A...............Q................. 80
Gga_COG4     ..A--------------GSD..-AMGCGL.M.RV.A....S....A.S...E..NA.QQ.........GS......A.Q.............Q.........Y....... 95
Aca_COG4     ----------------VG.S..PSSS.SL.M.E..A.PG.P....A.S...A..IT..E..EM.....G...G..AS................................. 94
Xtr_COG4     --------------LGFQ.D..----.VL.M.HV....D..D.KEA.....A...D..S..LT..G..TIV.K..LA.Q.............Q..S.............. 92
Dre_COG4     .EE---------AAS.ARRR..PAGV.AVQTDT.EA....ED..R..AQ..A..AE.QV.....VG...N..T..LS.Q.............V..S...N...S...... 101
Gac_COG4     ...---------SGTIA.KRCDS.SL.SV.MDT.SA....ED..R..RQ..S...E.DS...R.VG.EG..HT..LA.Q.........A...S..S.......S...... 101
Ola_COG4     ...---------IAPLAVKRCESSSLCSV.MDT.S.....ED..K..QQ..E...G..A...R.VG.EAA.HK..QA.Q....S....GS..S..S.......S...... 101
Tni_COG4     ----------------TARRCDS.PS.SV.MGN.SA....ED..R..QQ..LQ..E..A...R.VG.EGG.HT..LA.Q....S....G...S..S.......S...... 94
Tru_COG4     ...---------SASLVAQRCD-SAP.SV.M.N.S.....ED..K..QQ..VQ..E..A...R.VG.EGG.HT..LA.Q....S....G...S..S.......S...... 100

Hsa_COG4     SKVRQLDLAKNRLYQAIQRADDILDLKFCMDGVQTALRSEDYEQAAAHTHRYLCLDKSVIELSRQGKEGSMIDANLKLLQEAEQRLKAIVAEKFAIATKEGDLPQVERFF 219
Ptr_COG4     ----------............................N.........I............................................................. 181
Ppy_COG4     ......................................N.........I..............................................V.............. 219
Mac_COG4     ------------------------------------------------------------------------...................................... 125
Cja_COG4     ......................................N.........I............................................................. 219
Mmu_COG4     ......................................N.........I............................................................. 219
Rno_COG4     ................................................I..............................................A.............. 219
Ocu_COG4     ......................................N.........I............................................................. 219
Eca_COG4     ......................................N.........I.........................................T................... 219
Cfa_COG4     ......................................N.........I.........................................T................... 218
Bta_COG4     ......................................N.........I.........................................T....V.............. 219
Mdo_COG4     ......................................N.........I....S..............................H...V.T...D............... 220
Tgu_COG4     ......................................N.........I....S...............GI...............TT..T...DT.M.Q.......... 190
Gga_COG4     ......................................N.........I....S...............GI.....N....S.....T..T...DT.M.Q.......... 205
Aca_COG4     ......................................G.........I....S..........K.Q...I................V..T...DV...QE......... 204
Xtr_COG4     ..........S.........................MKN.........I....................NI.E...HH......H..VV.S...DA...S.......... 202
Dre_COG4     ........T.K..................T........NQ........I....S..Q......K..G...AVE.S.A......RN..TL.TTRLEE.VAT.......... 211
Gac_COG4     R.........T...NV.............T.......HN.........I....S..Q.........E.S.AV..S.VM.....KK..V.....LDE.VAAV..S...... 211
Ola_COG4     Q.........T...NV.............T........N.........I....S..Q.........E.S.AV..S.V.......K..V...K.LDE.VAAV..A...... 211
Tni_COG4     R.........T...KV.E......N....T........N.........I....S..Q.........E.S.NV..S.L.......K.RV.....LDE.VAAV..A...... 204
Tru_COG4     C.........T...NV.E......N....T........N.........I....S..Q.........E.S.SV..S.LM......K.RV.....LDE.VAAV..A...... 210

Hsa_COG4     KIFPLLGLHEEGLRKFSEYLCKQVASKAEENLLMVLGTDMSDRRAAVIFADTLTLLFEGIARIVETHQPIVETYYGPGRLYTLIKYLQVECDRQVEKVVDKFIKQRDYHQ 329
Ptr_COG4     .............................................................................................................. 291
Ppy_COG4     .............................................................................................................. 329
Mac_COG4     ......R....................................................................................................... 235
Cja_COG4     .............S...................L............................................................................ 329
Mmu_COG4     ..........D..S...................L...S..........................................F...........T......N.......... 329
```

```
Rno_COG4   ..........D..S...................L...S..........................................F...........T......N.......... 329
Ocu_COG4   .............S...................L..............................................F............................. 329
Eca_COG4   .............S...................L...............................................N............................ 329
Cfa_COG4   .............S...................L............................................................................ 328
Bta_COG4   .............S...................L..........................................................................R. 329
Mdo_COG4   .............S...........N.......L...................................................H..L...Q................R 330
Tgu_COG4   .............S...........N......QL.M.................................................H...........F.QTALRNE...R 300
Gga_COG4   .............S...........K......QL.M..........................V......................H..G...Q......E..V.E....R 315
Aca_COG4   .............S.......R...N......QL.MK...T.....................V...................M..H..A.............VQ.....R 314
Xtr_COG4   ............IS...G.......K......QLA...EGGE....L.......................L..........M...H..A.............VQH....R 312
Dre_COG4   ..L.......Q..AR.AQ...S.L........ILAV.S.LGE...P..........L.......................H..LAH..K...K.AQ.I.....Q....NN 321
Gac_COG4   .........QQ..AR.GQ...S.L.........LAA.GELGEK..PLV........L.....V...............H.....TH..Q...Q.AQ.I.....Q..G..N 321
Ola_COG4   .........QQ..AR.GQ...S.L.........LAT.G.LGEK..LL.........L.....VI..............H.....TH..E.....AQ.I....MQ..E.LN 321
Tni_COG4   .........QQ..AR.GQ...S.I........QLAT.G.LGEK..PLV........L.....VI..............H.....TH..Q...Q.AK.I..R..Q..G..S 314
Tru_COG4   .........QQ..AR.GQ...T.I........HLAT.G.LGEK..PLV........L.....VI..............H.....TH..Q...Q.AN.I.....Q..G..N 320

Hsa_COG4   QFRHVQNNLMRNSTTEKIEP--RELDPILTEVTLMNARSELYLRFLKKRISSDFEVGDSMASEEVKQEHQKCLDKLLNNCLLSCTMQELIGLYVTMEEYFMRETVNKAVA 437
Ptr_COG4   ....................--........................................................................................ 399
Ppy_COG4   ....................--........................................................................................ 437
Mac_COG4   ....................--........................R............................................................... 343
Cja_COG4   ....................--........................R..............................................I................ 437
Mmu_COG4   ...L..S......A......--.....V..................R....A.......................................F.I................ 437
Rno_COG4   ...L..S......A......--.....V..................R....A.....................................V.F.I................ 437
Ocu_COG4   ...Q.........A......--........................R...........................R..................I................ 437
Eca_COG4   ........M....A......--........................R..............................................I................ 437
Cfa_COG4   .......S............--........................R.............V..........S...................F.I................ 436
Bta_COG4   ......S.......S.....--........................R..............................................I................ 437
Mdo_COG4   ..QL...SM..S.AA.....--........................................................H............Y.I................ 438
Tgu_COG4   E.QQ...SM..S.SA.....--.......................I.R..MA......A............Y...................Y.I................ 408
Gga_COG4   ..QQ...SM..S.SA.....--.......................I.R..VA...................Y...................Y.I...Q............ 423
Aca_COG4   ..QQ...SM..S.VA.....--.....V..................RR..TA.........P.........S.....HH.....A......Y.I................ 422
Xtr_COG4   K.QQ..SCM..G.S.D....ST.D.....S.....S..T......I.R.VV..........T.......LQN....IKH.....SV.....Y.I....FY...S...... 422
Dre_COG4   K.QV..S.M..GM..D....--.D...V.C......S.A...F...RR..VA....A.A..D.A.I....QS.EQ..K..Q..R.......Y.IP....Y...S...... 429
Gac_COG4   K.QV..SSM.K.APG.G.K.--K....V..........A.......RR.VLA......AQSVTQIFEQ..QNVE...KH....R.......Y..P....Y...S.....T 429
Ola_COG4   K.QV..SSM.KSMPG.S...RN.....V.A........A.......RR.VMA......PQSITQEPT...QSVE...KH.S..RA......Y.IP....Y...S.....S 431
Tni_COG4   K.QV..SSM..STPA..M..--.....V..........V.......RR..L.....D.V---QS.T....QNVE...KH....RN......Y.I-....Y...S...... 418
Tru_COG4   K.QI..SSM..SMPA..M..--.....V..........A.......RR..L.......V---QSIT....QNVE...KH....RK......Y.IP....Y...S...... 425

Hsa_COG4   LDTYEKGQLTSSMVDDVFYIVKKCIGRALSSSSIDCLCAMINLATTELESDFRDVLCNKLRMGFPATTFQDIQRGVTSAVNIMHSSLQQGKFDTKGIESTDEAKMSFLVT 547
Ptr_COG4   .............................................................................................................. 509
Ppy_COG4   .............................................................................................................. 547
Mac_COG4   ........................................................................................................L..... 453
Cja_COG4   .............................................................L..........................................L..... 547
Mmu_COG4   ................................N............R...A..................L...................................L..... 547
Rno_COG4   ................................N............R...A..................L...................................L..... 547
Ocu_COG4   ....................................................................L...................................LN.... 547
Eca_COG4   ....................................................................L...................................L..... 547
Cfa_COG4   ....................................................................L...................................L..... 546
Bta_COG4   .........................................................H..........L...........S.......................L..... 547
Mdo_COG4   ...........................................S.........E.................F................................L..... 548
Tgu_COG4   M.S.......................................HS.........E..Y...KQ.........F................................Q..... 518
Gga_COG4   M.S.............................N.........HS.........E......KQ.........F................................Q..... 533
Aca_COG4   M..C........V.............................HSI........E......KV.........F........SL......................Q..... 532
Xtr_COG4   M..C.R...I.................................S..M......E...........................L.Q...............N.........S 532
Dre_COG4   M..A.V...S..V............S...T...S..V.....H..SV..T...E..VC...A.Y.VSAL..L....S...SL.Q.......IQ.L....QEQ..SAY... 539
Gac_COG4   M........N......C........S................H.NSV......E..N....Q......L.......S...SL.Q........N.M....AEN..AA.... 539
Ola_COG4   M...............C........S......N.........H.NSV......E..Y....Q......L.......S...SL.Q........N.L.....EN..AA.... 541
```

```
Tni_COG4     M...............C........S......N.........H.NIA......E..L....Q......L.......S...SL.Q........N.L.....EN..AA....  528
Tru_COG4     M..F............C..............ANT........H.NSA......E..Y....Q......L.......S...SL.Q........N.L.....EN..AA....  535

Hsa_COG4     LNNVEVCSENISTLKKTLESDCTKLFSQGIGGEQAQAKFDSCLSDLAAVSNKFRDLLQEGLTELNSTAIKPQVQPWINSFFSVSHNIEEEEFNDYEANDPWVQQFILNLE  657
Ptr_COG4     ..............................................................................................................  619
Ppy_COG4     ..............................................................................................................  657
Mac_COG4     ..............................................................................................................  563
Cja_COG4     ...................................K..............S...........................................................  657
Mmu_COG4     .............................................................A....S.V.........T.L....S........................  657
Rno_COG4     .............................................................A....S.V.........T.L....S.....................S..  657
Ocu_COG4     .............................................................A......V.........T.L.............................  657
Eca_COG4     ..................................................S...........................T.L.............................  657
Cfa_COG4     ..............................................................................T.L.............................  656
Bta_COG4     ..................................................G...........................T.L...........S.................  657
Mdo_COG4     ...............T.........................................................K....I.L.............................  658
Tgu_COG4     ...........M..........S..L...F........IE.....M...............N...........K....L.L...........S.............V...  628
Gga_COG4     ...........M..........A..L...F........I......M.D............VND..N.......K....L.L.I..S......S.............VH..  643
Aca_COG4     ...........M..........A..L...F........I......MV...........D..N..TNS......K....L.L...........S.................  642
Xtr_COG4     ...........M....N..N..N.M....L.....K..I......M.S.....K.......G......V....K....L.L.........................V...  642
Dre_COG4     ................N.....A......ASSDH.KE.I.......VNT.S..K.......Q...N...R...K...S..L...........SE......F...L.VQ..  649
Gac_COG4     ...........T...RN..N..S...N..P.SGE-...IE......VNT.A..K...........T....S.IK...S..L.IT....................L.I...  648
Ola_COG4     ...........T...RN..N..S......A.SGE-...IE......VST.T..K...........T.......K...SG.L.......................L.V...  650
Tni_COG4     .....A.....T...RN..N..S......AAAGE-...IE......VNT.A..K.......S...T..V....K...S..L.I.....................L.V...  637
Tru_COG4     .....A.....T...RN.....S...I..SAAGE-...IE......VNT.A..K.......S...T.......K...S..L.I.....................L.....  644

Hsa_COG4     QQMAEFKASLSPVIYDSLTGLMTSLVAVELEKVVLKSTFNRLGGLQFDKELRSLIAYLTTVTTWTIRDKFARLSQMATILNLERVTEILDYWGPNSGPLTWRLTPAEVRQ  767
Ptr_COG4     ..............................................................................................................  729
Ppy_COG4     ..............................................................................................................  767
Mac_COG4     ..............................................................................................................  673
Cja_COG4     ..............................................................................................................  767
Mmu_COG4     .............................................................................................A................  767
Rno_COG4     .............................................................................................A................  767
Ocu_COG4     .............................................................................................A................  767
Eca_COG4     ........G..........S..................................V......................................A................  767
Cfa_COG4     ...............................R.............................................................A................  766
Bta_COG4     ........G..........S.........................................................................A................  767
Mdo_COG4     ........G..........S.....I.I.................................................................A................  768
Tgu_COG4     ...T....G.......T........I.I.....L.....S......................................................................  738
Gga_COG4     H..T....G...A...N........I.T.....L...S.....................................I.................................R  753
Aca_COG4     ...G..R.G..........S.....I.L..................................................................................  752
Xtr_COG4     .L......G..S...EN..S.L...I.M...........S.................................T....................................  752
Dre_COG4     .L.....VG.......T..S.....I.M.M..T.F.C..S............A.V...SS..S..........T...........S........................  759
Gac_COG4     .L.T...TA...I...T..S.....ISI.M..T...CS.S..............V..................T....................................  758
Ola_COG4     .L.G....A.......TM.S.....I.I.M..T...CS.S..............V..................T...................................R  760
Tni_COG4     .L.G...VA.......T........ISM.M..TA..CS.S..............V..................T..................-.................  746
Tru_COG4     .L.....VA.......T..S.....ISM.M..T...CS.S..............V..................T....................................  754

Hsa_COG4     VLALRIDFRSEDIKRLRL  785
Ptr_COG4     ..................  747
Ppy_COG4     ..................  785
Mac_COG4     ..................  691
Cja_COG4     ..................  785
Mmu_COG4     .........N........  785
Rno_COG4     .........N........  785
Ocu_COG4     .........G........  785
Eca_COG4     ..................  785
Cfa_COG4     ..................  784
```

```
Bta_COG4    ..................  785
Mdo_COG4    ..................  786
Tgu_COG4    ..................  756
Gga_COG4    .....K...D........  771
Aca_COG4    ..................  770
Xtr_COG4    ..................  770
Dre_COG4    .....V............  777
Gac_COG4    ..................  776
Ola_COG4    ..................  778
Tni_COG4    ..-...............  763
Tru_COG4    ..................  772
```

```
Hsa_COG5    RELLQDGCYSDFLNEDFDVKTYTSQSIHQAVIAEQLAKLAQGISQLDRELHLQVVARHEDLLAQATGIESLEGVLQMMQTRIGALQGAVDRIKAKIVEPYNKIVARTAQL 110
Ptr_COG5    ......E.........................................K.............................................................. 110
Ppy_COG5    ......E.........................................K.............................................................. 110
Mac_COG5    ......E.........................................K.............................................................. 110
Cja_COG5    ......E.........................................K...............................M.................D............ 110
Mmu_COG5    QAI...D...E.....................................K...........................................M.S................ 110
Rno_COG5    Q.....D...E..S..................................K...........................................M.S................ 110
Ocu_COG5    ......E..R..........A...........................K...........................................M.................. 110
Eca_COG5    ......E...A.....................................K............................L...........L..M.................. 110
Cfa_COG5    ......E.........................................K...........................................M.................. 110
Bta_COG5    ......E.........................................K...........................................M....I............. 110
Mdo_COG5    ......E..L...S..................................K........................................I.........D............ 110
Tgu_COG5    A..RA.E..A..FR......A...........................K.....................................ST....RV...D......S..... 110
Gga_COG5    A..QA.E..A..FR......A...........................K...V.................................ST....R....D......S..... 110
Aca_COG5    ------E..A...R......V...........................K.........................KHVLYLSPL.ERR.-INKVNITTTSSN.N..S-SESV 102
Xtr_COG5    ..MQR.E..T...RD.....I...........................K.....................................S.I...RT...D......S..... 110
Dre_COG5    NS..K.E......Q.........A.A..H........R..E......K...C..............................S...S.....RT...D............ 110
Gac_COG5    IT..KE...T...ADN.......A.V..H...................K...S..........S...................S...A.....RT...D........IT.. 110
Ola_COG5    NSI.K.D..TE.VGD.......AA.V.QH.......GG......R..K...G...............................S...A....M.T...D........IT.. 110
Tni_COG5    ------E.....VS.N.......A.A..H...................K...S..........S...................S...A.....RN...D........IT.. 104
Tru_COG5    NS..K.D..R..VPDN.......A.A..H...................K...S......D...S...................S...A.....RS.............IT.. 110

Hsa_COG5    ARLQVACDLLRRIIRILNLSKRLQGQLQGGSREITKAAQSLNELDYLSQGIDLSGIEVIENDLLFIARARLEVENQAKRLLEQGLETQNPTQVGTALQVFYNLGTLKDTI 220
Ptr_COG5    .............................................................................................................. 220
Ppy_COG5    .............................................................................................................. 220
Mac_COG5    .............................................................................................................. 220
Cja_COG5    .....................................................T..............................................H......... 220
Mmu_COG5    .................Y..................................................................V...............H......E.V 220
Rno_COG5    .................Y..................................................................V...............H........V 220
Ocu_COG5    .................Y............C..............................................R.............................H.V 220
Eca_COG5    .................Y................................R.................................V...............H......... 220
Cfa_COG5    .................Y.T................................................................V...............H......... 220
Bta_COG5    .................Y...............................................V..................V...............H......... 220
Mdo_COG5    .................Y...............................................V..................V...............H......... 220
Tgu_COG5    .K..A............Y..................................................................V...............H......... 220
Gga_COG5    .K..A............H..................................................................V..S....................... 220
Aca_COG5    FQI.A............Y..................................................................I......................V.. 212
Xtr_COG5    ....S............Y.......................S........M.................................V...................A...I. 220
```

```
Dre_COG5   .................Y................................V.....D.........S.................M.I...S.............N.RE..  220
Gac_COG5   ....M............Y................................V.............L.S.................M.I.......M.........S.SV..  220
Ola_COG5   .................Y.......................H........V.............L.S.................M.I...S.............S.GE..  220
Tni_COG5   D...G............Y.......................S........V.............L.S.................M.I.................I.RE..  214
Tru_COG5   D................Y................................V.....Q.......L.S.................M.I...N.............I.RE..  220

Hsa_COG5   TSVVDGYCATLEENINSALDIKVLTQPSQSAVRGGPGRSTMPTPGNTAALRASFWTNMEKLMDHIYAVCGQVQHLQKVLAKKRDPVSHICFIEEIVKDGQPEIFYTFWNS  330
Ptr_COG5   .....................................................L........................................................  330
Ppy_COG5   .....................................................L........................................................  330
Mac_COG5   ........TLIYHRVDGITH.ELIRI..K.WI.....................L........................................................  330
Cja_COG5   .N..................V..........L.....................L...........................................E............  330
Mmu_COG5   .........A..DS..N...V.................AA.....S..GF...L...........C.A...........T...............I.......L.M...A  330
Rno_COG5   .N.......A..DS..N...V..........A......A..........F...L...........C.A...........T.......................L.M...A  330
Ocu_COG5   .................................................F...L.......V.Y.C.............T.......................L.K....  330
Eca_COG5   .......R.A.....S.................................F...L.......V...CT..........I.........................L......  330
Cfa_COG5   .............H...................................F...L.......V...CT..........I.........................L......  330
Bta_COG5   .N.......AI......................................F...L.......V...FT..........I.T....................A..L......  330
Mdo_COG5   .N..............N.........S..T.IK.....A..........F..TL.........Q.C.A...........................L....S..L.....V  330
Tgu_COG5   AN......TV.....KN............TVT......AA.........F..AL.........Q.C.A................................SD.L.K..TA  330
Gga_COG5   AN......TV.....KN............TVT......AA.........F..AL.........Q.C.A.........L.......I..V...........SD.L.K..TA  330
Aca_COG5   .N.............SN.....I......TVA......A..........F..AL.........Q.C.A...........S......T.V...D.LA....SD.L....TA  322
Xtr_COG5   .......STA.N...S......I...T..AVQ..A...AA..S......F..AL.........Q...S.V.........T........V...D..A...HSD.L.K..S.  330
Dre_COG5   R......RTSVQ..VVN...........NT--..A...AV.........F..AL...L.....Q.C.A...........T......T.V...D..I.....D.LH...S.  328
Gac_COG5   G...G..RT.IHD..TR.....G....TNP--..A...AVL........F..AL...L.....Q.C.A.R.........M......T.V...D..I...K.D.LC....D  328
Ola_COG5   R...G..QTAIKDH.C......G..V.ANP--..A...AAL.A......C..AL...L.......C.AWT.........M........V............D.L....TD  328
Tni_COG5   SG..G..RSAIQD..SK.....G....ANP--K.A...AVL.....M..F..AL...L.....Q.C.A.R.........T......T.V...DD.I.....D.L....TD  322
Tru_COG5   NG..G..RS.IQDS.SK.....G....ANP--K.A...AML........F..AL...L.....Q.C.A.R.......I.M......T.V...DD.F....AD.L.....D  328

Hsa_COG5   VTQALSSQFHMATNSSMFLKQAFEGEYPKLLRLYNDLWKRLQQYSQHIQGNFNASGTTDLYVDLQHMEDDAQDIFIPKKPDYDPEKALKDSLQPYEAAYLSKSLSRLFDP  440
Ptr_COG5   ..............................................................................................................  440
Ppy_COG5   ..............................................N...............................................................  440
Mac_COG5   ..............................................N...............................M...............................  440
Cja_COG5   ............................................G.N.............C....L.........V..................................  440
Mmu_COG5   ..L....H..S................................S..NT..T.SP...P..C...P.....T..M.RL.R...............................  440
Rno_COG5   ..L....H..A..D................................NS..A.GTR..A...A..PDVD.....MSTL.R...............................  440
Ocu_COG5   ..........L.....V....S......................H.N.......G.S.....E..........V....R...............................  440
Eca_COG5   .........R...D................................N...K...................T.....T.................................  440
Cfa_COG5   .........R...D................................N...K..............Q.D..T.......................................  440
Bta_COG5   .........QT...................................N..........A...A....T...T..V....................................  440
Mdo_COG5   ..DT...R.QT.....V...........................N.N......S.......I...QI...V....MQ..Q..............................  440
Tgu_COG5   ...T.....QS..D................................N..R...T......FAE..QI.E......MQ.NE.............Q................  440
Gga_COG5   ...T.....QS..D................................N..R...T..SP..FAE..Q..E......MQ.TQ.............Q................  440
Aca_COG5   ...V.....QK..D...............................EN..R..ITN.SAEHFAE..QI...T.....Q.QQ.....I.......A................  432
Xtr_COG5   ...I.....EK...C...............................NL..S.SN..NS..VS..PT..........T..Q.V.A..M.......................  440
Dre_COG5   ...T..EELQK..AA.T.....L............E.........AS...ALVS..-AG.D.E.PAT.N.TE.L.THA........D.......................  437
Gac_COG5   ..NT..EEL.R..EA.S.....L........Q...E..R......ASL..ALTS..GI.TSL.ITAA.T.S..L.THGQQ..N..Q.......L................  438
Ola_COG5   ..ST.NDE..R.AEA.S.....L............E..R....H.TGP..A-PT.AAM.ASL.IGTT.T..SEL.MNI.Q..N..........D..S.............  437
Tni_COG5   ..ST.GEE..R..EA.S..................E..R......ASL..ALSGTAGS.SS..ISGT.T.S..L.THGQQ..N...........................  432
Tru_COG5   ..RT.GEE..T..EA.S..................E..R......TSL..ALP.TA-I.SAI.ISGA.L.S..L.THG.Q..N...S.....L..Q..............  437

Hsa_COG5   INLVFPPGGRNPPSSDELDGIIKTIASELNVAAVDTNLTLAVSKNVAKTIQLYSVKSEQLLSTQGDASQVIGPLTEGQRRNVAVVNSLYKLHQSVTKVVSSQSSFPLAAE  550
Ptr_COG5   ..............................................................................................................  550
Ppy_COG5   ...............E..........................................................I...................................  550
Mac_COG5   ..........................................................................................................P...  550
Cja_COG5   ..........................................................................................................P...  550
Mmu_COG5   .....................T...T.........A.................A........................K...G.....F..........A.....SAT..  550
Rno_COG5   ...................................A.................A........................K...G.....F..........A.....SA...  550
```

```
Ocu_COG5  ..............................I....A.................G........................K...........................A... 550
Eca_COG5  ..............A...E......T.........A.................G.......C.........................................A..P... 550
Cfa_COG5  ..............A...E................AD......R.........G........................................I...........P... 550
Bta_COG5  ..............A...E................S.................G....................................................P... 550
Mdo_COG5  ..............P....S...............S.................G...........E.............................I....N.V...A... 550
Tgu_COG5  ...................S...............PD.S...A..........G.......................................A.L..IT......A... 550
Gga_COG5  ...................S...............PD.S...A..........G.......................................A.L...TN.N...A... 550
Aca_COG5  ..............T....S...............P..S.............FG...................................R.....L.......A..A.S. 542
Xtr_COG5  ..............A....S........M......PS.SS.....I.......G...................................R....IQ..I.G...LSA... 550
Dre_COG5  ......Q.......N....S............S..SG..I..A..A...V..FC.......C...................I......FR.Q.A.A..I.GLG....... 547
Gac_COG5  ......M.......NE...S.....S......S..PK.S......A......FC.......C...E...............IT......R.Q.AI..II.GSG.C.PVTA 548
Ola_COG5  ......L.E.S...N..........S.........PH.STS.A..A...V..FC.......C....................G......R.Q.G.L.IL.GLV.C.PP.V 547
Tni_COG5  ......L..H....N..........S......S..P..A...A..T...V..FC.......C...........................R.Q.A...M..GSGTC.A..A 542
Tru_COG5  ......L..H.........A.....S......S..P......A..T...V..FC.......C..............A............R.Q.A...II.GLGTC.A.TA 547

Hsa_COG5  QTIISALKAIHALMENAVQPLLTSVGDAIEAIIITMHQEDFSGSLSSSGKPDVPCSLYMKELQGFIARVMSDYFKHFECLDFVFDNTEAIAQRAVELFIRHASLIRPLGE 660
Ptr_COG5  .............................................................................................................. 660
Ppy_COG5  ..V...........................................................................................I............... 660
Mac_COG5  ...........T..................................................................................I............... 660
Cja_COG5  .........................E...................P...R............................................I............... 660
Mmu_COG5  ...M....T..D..G..I.......A.................AS.........................N.......................I.....N......... 660
Rno_COG5  ...M....T..D..G..I.......A.........V.........T........................N.......................I.....N......... 660
Ocu_COG5  ........T..T..G.............................V.G................................S..............I.....N......... 660
Eca_COG5  ...T....T..V..G..............................TT...............................................I.....N......... 660
Cfa_COG5  ........T..V..GS............................I.................................................I.....N......... 660
Bta_COG5  ..........YV..G..............................PN...................V............S..............I.....N......... 660
Mdo_COG5  E.VAT...T..D..G............S.................P........................N...R....SE.............IK..V.N...L..... 660
Tgu_COG5  ..VTT....V.D..GS......N....SV.................N...........................R....F.........M....I.....N......... 660
Gga_COG5  E..TT....V.D..GS......N....SV.....................................G.......R....F.........M....I.....N......... 660
Aca_COG5  E..ST...T..D..G.......N....S.................PT.....T.............S...N...R....Y...YES........I.I...N...L..... 652
Xtr_COG5  .V.S....V.FN..G.......N..A.S.............A.N..N...........................R........Y..........I.V...N...L..... 660
Dre_COG5  .SLTAS.E.VQ...SS......N..T.SV....L........SP.PAG.R.........R..........N...RPLQ....LY.S..K.....IT........L..... 657
Gac_COG5  EPFS.S.E.VQV..SS......Q..S.S.......L.......P..CP.............M....S...A...R..Q.A..IYES...L....I......T..L..... 658
Ola_COG5  EALS.S.E.V.S..GS.LA...Q..S.S.....F.L.......PV..PDR................S...A...RP.Q.A..IYEK........IQ..V.....L..... 657
Tni_COG5  DVLS.S.EGLQ...SSS.....Q..S.S.......L.......PV..PD.................S.......R..Q.T..I.....R.........V..S.IL..... 652
Tru_COG5  DVLS.S.E.LQ...DSS.H...Q..S.S.......L.......PVC.PD.................S.......R..Q.M..I..S..R.........V..S.IL..... 657

Hsa_COG5  GGKMRLAADFAQMELAVGPFCRRVSDLGKSYRMLRSFRPLLFQASEHVASSPALGDVIPFSIIIQFLFTRAPAELKSPFQRAEWSHTRFSQWLDDHPSEKDRLLLIRGAL 770
Ptr_COG5  .................................................N............................................................ 770
Ppy_COG5  .................................................N....................................A..............K........ 770
Mac_COG5  .................................................N....................................A....................... 770
Cja_COG5  ...........................................T.....N....................................A....................... 770
Mmu_COG5  ...L...............L.......................T.....D...V..I.............................A..................L.... 770
Rno_COG5  ...L...............L.......................T.....D...V..I.............................A..................L.... 770
Ocu_COG5  ...........................................T.....N......I....VV.......................A....................... 770
Eca_COG5  S..........................................TN................TV.......................A....................... 770
Cfa_COG5  ...V.......................................T............I.............................A....................... 770
Bta_COG5  ...........................................T............I...............P.............A....................... 770
Mdo_COG5  .................A.L............L..........T.....N......I.......H.....................A.Y..................... 770
Tgu_COG5  .................A.L............Q..........T...I.......E.......L........P............IA.Y..............A...... 770
Gga_COG5  .................A.L............Q..........T...I.......E.......L........P............IA.Y..............A...... 770
Aca_COG5  .................A.L...........KL......M...T...I.....V.......TV.........P...........TIA.Y.R............I.V.... 762
Xtr_COG5  .................A.L....A.......L..........T...I...SSV..L..Y.V.L........PD....Y......IA.Y........L.....A..K... 770
Dre_COG5  .................A.L.........A..L..........TN..I...Q....L..Y.T.LH.M...........H......IS.Y.......S......T...... 767
Gac_COG5  .................A.L.........P.............N..LI.....V..L..Y.TLLH.F.C...S.....H......VA.Y..........R...M...... 768
Ola_COG5  ..............M..A.L...................F...T..LI.A...V..L....TLLH...S...P.....H......VA.Y..........R...S...... 767
Tni_COG5  ..............M..A.L.........P.........F...T..LIVNN..V.....Y.TLLH.......P..R..H......IA.Y..........R...S.L.... 762
```

```
Tru_COG5     .................A.M........RP.............T..LIVNNA.V.E...Y.TLLH.......P..R..H......IA.Y..........R...S.L.... 767

Hsa_COG5     EAYVQSVRSREGKEFAPVYPIMVQLLQKAMSALQ 804
Ptr_COG5     .................................. 804
Ppy_COG5     .................................. 804
Mac_COG5     .................................. 804
Cja_COG5     .................................. 804
Mmu_COG5     ..........D....................... 804
Rno_COG5     ........G............L............ 804
Ocu_COG5     ......................L.........H. 804
Eca_COG5     .................................. 804
Cfa_COG5     .............................T.... 804
Bta_COG5     .................................. 804
Mdo_COG5     .......K.......................T.. 804
Tgu_COG5     ........T......................... 804
Gga_COG5     ........A......................... 804
Aca_COG5     .......K..............I......L.T.. 796
Xtr_COG5     .......KA.D....................SMF 804
Dre_COG5     ........A.Q...........L.M....T..S- 800
Gac_COG5     ........A.Q......I....L....R.T.SS. 802
Ola_COG5     ........T.Q..N........L.V....T.GP- 800
Tni_COG5     ........A.Q......I....L....R.S.VS. 796
Tru_COG5     ........A.Q......I....L....R.SAGT. 801


Hsa_COG6     MAEGSGEVVAVSATGAANGLNNGAGGTSATTCNPLSRKLHKILETRLDNDKEMLEALKALSTFFVENSLRTRRNLRGDIERKSLAINEEFVSIFKEVKEELESISEDVQA 110
Ptr_COG6     ...............................S.............................................................................. 110
Ppy_COG6     .......................V.......S.............................................................................. 110
Mac_COG6     ...............................N.............................................................................. 110
Cja_COG6     ...............................N...................................T.......................................... 110
Mmu_COG6     ..DA....A..P.S.....FS....A.P.QPN...............E.............A...................R.........N...D........N..... 110
Rno_COG6     ..DT....A..P.S......S....A.P.QPN...............E.............A...................R.............D........N..... 110
Ocu_COG6     ...A.......P........S.....A.SQSN.................................................R.........R...D........N..... 110
Eca_COG6     .......SSSAPIR.LPTASAM........SS...V.....T......I............S...................R......................N..... 110
Cfa_COG6     ..D.GA..A..PPS......S.....AL.Q.G.......N.........................................R......................N..... 110
Bta_COG6     .........V.P.................Q.S.......R...D.......D.............................R......................H..... 110
Mdo_COG6     ........A.LPVS.PPSS.G...S.AATQSN.................................................R......................N..... 110
Tgu_COG6     ..D------TAAPPPPPAAPAP.LPAAPG.--.......N.........................................R.........H...Q........N..... 102
Gga_COG6     ..D------TAPLPP..APAGP.I--------.......N.........................................R.........H...Q........N..... 96
Aca_COG6     --------------------------------.......R.V.....EA............A..................HR.........Y...Q......G.N....F 78
Xtr_COG6     ..D------VELNG.GPT---ISTTPAAVQST.......N...........D.........A..T.....S..........R..S......G...K.R....N.N..... 101
Dre_COG6     ...------TKADLT.DAS-.SSNNNV.SQPN..........................S..V..T................R..S......R...D.......VH..... 103
Gac_COG6     ..D------PKLEMSSD.STALQNPNAHTQPN.......N.....................V..TD...............R..S...D.AR...D...A...VN..... 104
Ola_COG6     VCS..----G.ASIPGDSSTVTSNSNV.SQPN.......N.....................V..T................R..T...D.AR..Q........VH....D 106
Tni_COG6     ..D------.KVE.S.DSSTITQNQNAQSQ.N.......N.....................V..T................R........AQ...........VN..... 104
Tru_COG6     ..D------.KVDIS.DSSTITQNQNAQSQ.N.......N.....................V..T................R........AQ...A.......VN..... 104

Hsa_COG6     MSNCCQDMTSRLQAAKEQTQDLIVKTTKLQSESQKLEIRAQVADAFLSKFQLTSDEMSLLRGTREGPITEDFFKALGRVKQIHNDVKVLLRTNQQTAGLEIMEQMALLQE 220
Ptr_COG6     .............................................................................................................. 220
Ppy_COG6     .............................................................................................................. 220
Mac_COG6     ......................................................................E....................................... 220
Cja_COG6     ..S......................................................I.................................................... 220
Mmu_COG6     ..S...........................A.N.R.......V....A................G..V.......................................... 220
Rno_COG6     ..S...........................A.N.R......................T......G..V.......................................... 220
```

```
Ocu_COG6   ..S......N....................A...R......................G.........V..........................................  220
Eca_COG6   ..............................A...R...........................................................................  220
Cfa_COG6   ..S....................I......A...R......................G.....................R..............................  220
Bta_COG6   ..S...........................A...R...........................................................................  220
Mdo_COG6   ............K.................A...R....................................................I......................  220
Tgu_COG6   ..S..E..S...K.................A.N.R..MK.......IA.....P...N.....KDE.................D...I.......R..............  212
Gga_COG6   ..S..E..S...K.................A.N.R..MK.......IA.....P...N.....KDE.................D...I.......R..............  206
Aca_COG6   .N....E..NH.K.T...............A.N.R..LK..I....ITE...S.E..NI...A.D..V..N...V............I.......RS.............  188
Xtr_COG6   ..S.........K......H............N.R............A.........NI........L......V............L......................  211
Dre_COG6   ..S..EE..N..K.............N...G.NHR..V.....Q........SAA..AT..SA.D........R..N...N..E......................V...  213
Gac_COG6   ..A.SEE..N..K.............N...G.NHR..L.....Q...A....SNE..AT...S.DA.........SEP-----EMS.SS.................V...  209
Ola_COG6   ..T..EE.....K....K........N...G.N.R..V.....Q...A....S.E..AV..NA.DA.V......V.S......E...I..................V...  216
Tni_COG6   ..E..EE..N..K.............N...G.NHR..V....VQ...A....SHE..AT...A.DA.LS.E.....KQ..H..E...I..................V...  214
Tru_COG6   ..E..DE..N..K.............N.F.G.NHR..V....VQ...G....ATE..AT...A.DA..........R...H..E...I..................V...  214

Hsa_COG6   TAYERLYRWAQSECRTLTQESCDVSPVLTQAMEALQDRPVLYKYTLDEFGTARRSTVVRGFIDALTRGGPGGTPRPIEMHSHDPLRYVGDMLAWLHQATASEKEHLEALL  330
Ptr_COG6   ..............................................................................................................  330
Ppy_COG6   ..............................................................................................................  330
Mac_COG6   .......................I......................................................................................  330
Cja_COG6   .S.............A.........A....................................................................................  330
Mmu_COG6   ...............A.........A..S.................................................................................  330
Rno_COG6   ...............A.........A....................................................................................  330
Ocu_COG6   ..................H....I...............................................S......................................  330
Eca_COG6   ...........N...........I.....K................................................................................  330
Cfa_COG6   .......................I......................................................................................  330
Bta_COG6   .S.....................I......................................................................................  330
Mdo_COG6   .S.....................I......................................................................................  330
Tgu_COG6   .S.......T.N...........I....A..........................A.............L......................................M.  322
Gga_COG6   .S.......T.N...........I....A..........................A.............L......................................I.  316
Aca_COG6   .S..Q......G...A.......I....A....................A.....A......................................................  298
Xtr_COG6   .S..Q......N...........I..I.A...D.........N............A...................................................S..  321
Dre_COG6   ....Q..........G....T..IP...S....S.....................A............................M.........................  323
Gac_COG6   .S..Q......ND..G......................................CA.............Q..............M.........................  319
Ola_COG6   .S..Q......N...G....T..I..............................CA......................................................  326
Tni_COG6   .S..L......N...G....T..I.....E.........DIFR............A............................M.........................  324
Tru_COG6   .S..L......N...G....N..IG....E.........................A............................M.................R.......  324

Hsa_COG6   KHVTTQGVEENIQEVVGHITEGVCRPLKVRIEQVIVAEPGAVLLYKISNLLKFYHHTISGIVGNSATALLTTIEEMHLLSKKIFFNSLSLHASKLMDKVELPPPDLGPSS  440
Ptr_COG6   ..............................................................................................................  440
Ppy_COG6   ...................................................................T....V.........................I...........  440
Mac_COG6   .......I...........................................................T..........................................  440
Cja_COG6   ....A......V..........................................P.P..........T..................N.......................  440
Mmu_COG6   ....A...K..........................L..............................AT.................T......N.................  440
Rno_COG6   ....A...K..........................L..............................AT.................S......N.....I...........  440
Ocu_COG6   ...P...............................L...............................T..........................................  440
Eca_COG6   ...................................................................T..........................................  440
Cfa_COG6   ....I..I...........................................................T..........................................  440
Bta_COG6   ....A........................................................I.....T..........................................  440
Mdo_COG6   .M..I...D.....I....................................................T..........................................  440
Tgu_COG6   .L..I.............................................................AT..........................................  432
Gga_COG6   .L..I.............................................................AT..........................................  426
Aca_COG6   .FI..E.....M...............................................N......AV.........Q....................I....A......  408
Xtr_COG6   .L.NI....D.........................I........F..............A...T..SS...........................L.......A......  431
Dre_COG6   .Q..V......M.......................I..........L..............M.T.VSS..M.MD..M....M..........R..............TA  433
Gac_COG6   .Q..LP.....L....A.............................L............S.I.T.VAS..I......I....M..........R.........V....TA  429
Ola_COG6   .Q..L.DL.D.M..................................L............S.I.T.VAS..M...........M..........R.........A....TA  436
Tni_COG6   ....Q..A.D.M.........................D........L............S.I.T..VS..I......V....M..........R.........A....TG  434
```

```
Tru_COG6    ....HH...D.M......V..................D......H.L............S.I.T..VS..V......I....M..........R.........A....TG 434

Hsa_COG6    ALNQTLMLLREVLASHDSSVVPLDARQADFVQVLSCVLDPLLQMCTVSASNLGTADMATFMVNSLYMMKTTLALFEFTDRRLEMLQFQIEAHLDTLINEQASYVLTRVGL 550
Ptr_COG6    .............................................................................................................. 550
Ppy_COG6    .............................................................................................................. 550
Mac_COG6    ......I....................................................................................................... 550
Cja_COG6    .............................................................................................................. 550
Mmu_COG6    ..S...T...DI........I...............A......................................................................... 550
Rno_COG6    ..S...T...D................................................................................................... 550
Ocu_COG6    .............................................................................................................. 550
Eca_COG6    ...........................................................................................................T.. 550
Cfa_COG6    ...........................................................................................................A.. 550
Bta_COG6    ......V...........................................H........................................................A.. 550
Mdo_COG6    ..S...A........................................................................K...........................A.. 550
Tgu_COG6    ......N.............I.........................M............Y........R..........K...........................A.. 542
Gga_COG6    ......N.......................................M................................K...........................A.. 536
Aca_COG6    ......A...........C.I...T............................S...........H.............K......Y....................... 518
Xtr_COG6    ......T......V.............................................Y...C...............K................V..........A.. 541
Dre_COG6    S.T...S.............L.........A.....I......L...............Y...................K.....E................F....AA. 543
Gac_COG6    S.T...S.......................A.....I......L...............Y......V............K.....E.....................A.. 539
Ola_COG6    SIT...S......T................A.....I......L..............SY......V............K.....E.....................A.. 546
Tni_COG6    S.T...S.......................A.....I......L..............CY......V............K.....E.....................A.. 544
Tru_COG6    S.T...S.......................A.....I......L..............CY......V............K.....E.....................A.. 544

Hsa_COG6    SYIYNTVQQHKPEQGSLANMPNLDSVTLKAAMVQFDRYLSAPDNLLIPQLNFLLSATVKEQIVKQSTELVCRAYGEVYAAVMNPINEYKDPENILHRSPQQVQTLLS 657
Ptr_COG6    ........................................................................................................... 657
Ppy_COG6    ........................................................................................................... 657
Mac_COG6    .C...A..............A...............................................................V...................... 657
Cja_COG6    .N...........H................................M...............I............................................ 657
Mmu_COG6    ......I...R.D...........A.A.....A..........H..M....S..........I...........A..H......D.A.....S......E..K.... 657
Rno_COG6    ......I...R.D.....S.......A.....A..........H..M....S..........I..............H......V.A.....S......E..K.... 657
Ocu_COG6    ...............C...L......A.....L.............M...............I.............................S.............. 657
Eca_COG6    ...............P...L......A...................M...............I...........D.........G.......SV............. 657
Cfa_COG6    ...................L......A................S..M...............I.....................V.......S.............. 657
Bta_COG6    .....M.....V...P...L......A...................M...............I.....................V......GS.............. 657
Mdo_COG6    ...............P.V.L.S....S...................M...............I...........S.L.T.....T.......T.............. 657
Tgu_COG6    .....AL........P.S.LSSM...S..V..A..........S..MS..............I...........S.L.....D.S.......T......H...A... 649
Gga_COG6    .....S.........P.S.L.S...MS..V..A..........S..MS..............I...........S.L.....D.S.K.....T......H...A... 643
Aca_COG6    .....VL........P...I.SM.AMS................G..M..I.....TA..Q..I.L.........S.........D.K.....TL.....H...A... 625
Xtr_COG6    .Q..SC.........P.S.L......S............A.....QM....YF.........I........K..S.L.E.....V.G.....TG............. 648
Dre_COG6    .H..SC....TAD..P...L.GM..TS..T.........AS..T.VM.........AI....FR.......G..A...K..T..T.T...A.SV.........A... 650
Gac_COG6    ....SC....SA...P.SLL.SME.SSV............S..T.VM.........AI....FR.............H..LSS.A.S......L.P........... 646
Ola_COG6    .....C....ST...P.SLL.SM.GSSV....L.......S..S.VM.........AI....FR..........A...S.LN..N.G.....TL.P.T......... 653
Tni_COG6    ....SC....TA...P.SLL.SM..SSV.T..........S..T.VM.........AIR...FR.............HT.LTS.P.G.....TLVP...K....... 651
Tru_COG6    ....SC....CA...P.SLL.S..GSSV.T..........S..T.VM.........AI....FR..............T.LTS.S.G.....TLVP...K......C 651
```

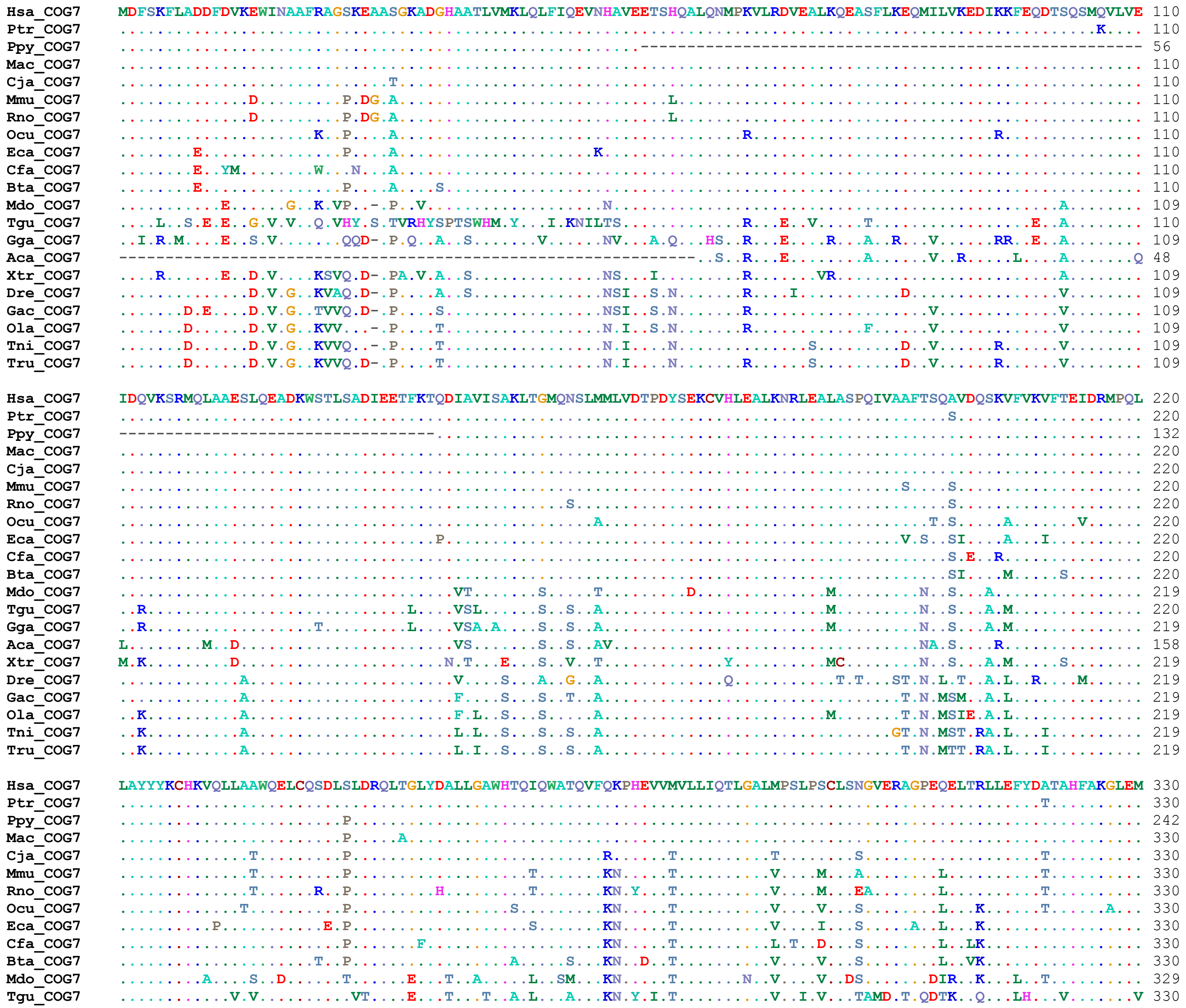

```
Gga_COG7    ............V.V..D........N....E...T...T..S.L.......K......T..........V..I.V...TAM..T.Q.TK.SK...L....V........ 329
Aca_COG7    ............VS...D......P..........L..ET....L...M...KS..Q..T...L.A....A.PI.A...LSM......VK.GL..SL.ET.TF..R...A 268
Xtr_COG7    ............VTV........MP......EF..T..S.....L..VS...K..Q...T..M....AMV...I.V...TAM...S..DK.ST...L.N..G....SM.T 329
Dre_COG7    .........A..TV..ESI....AP.KQ..SEF..T..ST....L..SS...RN.C..LT..M.....MV...I.D.IAETLK.CSGDCR.DV...LHQTS.N.SRS..A 329
Gac_COG7    .........G..VSM..D.S..E...NQ..SEF..T..SS..S.L..SS...KN.Y...T........MV...I.V...SA...AQ...R.DT...LHQT.SA.GQS..G 329
Ola_COG7    .........G..VSV..D.S..E...NQ..SEF.ET..ST..V.L..C....RN.Y...T........MV...ISV...TAI..PQ...R.EA...LHHT.STCGHH..A 329
Tni_COG7    .........G..VSI..D.S.G....NQ..SEF..T..ST..C.L..TS...KN.Y...T........MV...V.V..NTA...AQ...R.DT...LHHV.FT.GHS..T 329
Tru_COG7    .........G..VSM..D.S..E...NQ..SE...T..S...C.L..TH...KN.Y...T........MV...I.V..NTAM..AQ...R.DT...LHSI.ST.GHS..A 329

Hsa_COG7    ALLPHLHEHNLVKVTELVDAVYDPYKPYQLKYGDMEESNLLIQMSAVPLEHGEVIDCVQELSHSVNKLFGLASAAVDRCVRFTNGLGTCGLLSALKSLFAKYVSDFTSTL 440
Ptr_COG7    .............................................................................................................. 440
Ppy_COG7    .................................N.........I...............................I.........................W.T------ 346
Mac_COG7    .................................N.............................................I.............................. 440
Cja_COG7    ......Y............V.......................I..M............................I...I...................T.......... 440
Mmu_COG7    ......QD......V.......G....F.........N.....I.....................H.............AK...........T...........H..NA. 440
Rno_COG7    ......QD......V.......G...........L........I..................Q..H.............AK...........T......T....H..NA. 440
Ocu_COG7    .....P....M.......Q...G..............NH....L...................................A............T.............G... 440
Eca_COG7    .....P.D......M.......G..............K.....I...................................I............T......T.......... 440
Cfa_COG7    .Q...P..Y.........E...G...........L..K.V...I...................................I............A................. 440
Bta_COG7    .....AY.Q.....M.......G..............KY....F.E.........................S.......I...S........T................. 440
Mdo_COG7    ...S.SR.......M...E..FG.......Q..EL.D.H....I.......W.....................G.....IK..D...............T.......N.. 439
Tgu_COG7    .M..N.K.Q.....M...EV.A........E...L..E.....I.......W.........N.......T..AG.I.S..KL.D...V....K...A..S..T....N.. 440
Gga_COG7    .M..N.K.Q.....M...E...H....F......L..E.....I.......W.........N...S...I...G.I.N.IKL.D...V....K...A.....T....N.. 439
Aca_COG7    .MM.C.KDC.....G...E.T.G....F.MQ.RAL..E...............IL..........S...........H.LQL.D...VG...K..RA.H...S...SNA. 378
Xtr_COG7    .I..NMG.Y....LI......FG.......Q...L........I..M...R...L..........SR..S...G.M...IK..D...L....Q..Q...K...T...L.. 439
Dre_COG7    .MQ.Q.G.C..L..A...SC..S...T..IQ..GL..T.....L......R...L...L..T...Q.V....A.......K..D..AV...IK..RA..N......SV.. 439
Gac_COG7    .MS...G.NT.L..N...C.L.........L...L..RH....I..............E......G......G.......KL.D..AV....K...A..T......ST.. 439
Ola_COG7    .M....A.N..L..N...C.M.....A...Q..EL..AH...............L...E..N...G..............TL.D...V....K...A..T......ST.. 439
Tni_COG7    .M....G.N..L..N...C.L.....S...R..ELQ.AH....I..............E.M....G.......-......KL.D..AV....K...A..T.QCNY.ALF. 438
Tru_COG7    .M....G.N..L..N...C.L.....S...Q..EL..AH....I..I...........E.M....G.......S......KL.D..AI....K...A..T......ST.. 439

Hsa_COG7    QSIRKKCKLDHIPPNSLFQEDWTAFQNSIRIIATCGELLRHCGDFEQQLANRILSTAGKYLSDSCSPRSLAGFQESILTDKKNSAKNPWQEYNYLQKDNPAEYASLMEIL 550
Ptr_COG7    .............................................................................................................. 550
Ppy_COG7    -----------------------------..........................................A..........S........................... 427
Mac_COG7    ..V.......D...D.....................................----------------------------------........................ 516
Cja_COG7    R.........D.......................................................................S........................... 550
Mmu_COG7    ..........D.................V...........Q.......................Y.........D.......SP.......................... 550
Rno_COG7    ..........D.................V...........Q.......................Y.........D.......SP....................N..... 550
Ocu_COG7    ..........D..A.F............V...........Q.......................Y.............A...S........................... 550
Eca_COG7    H.........D.............................Q.......................Y...N.T...D.......S........................... 550
Cfa_COG7    ..........D.............................Q.......................Y.........D.......S........................... 550
Bta_COG7    H.....YR..D..L..........................Q.......................F.....T...D.......S...............S....G...... 550
Mdo_COG7    ..........D..SS.............V................................T..Y.....T...D.S..E..SLV............ES.V.....L.V. 549
Tgu_COG7    ..........DVLADP............V...SI.....HQ.......................Y..C.FS...DTSSAE..S.I..........L.E..S.......T. 550
Gga_COG7    ....M.....D..SD.............V...T.......Q.......................Y..C.FS...DTSA.E..SNV..........L.E..S.......T. 549
Aca_COG7    L......Q..E..SD.......G...TAV...G......CQ..............R........Y..C..S.L..PS-PE.HSPF......H....AES.....A.L.T. 487
Xtr_COG7    ..........N..AD...L...S.....V...GA......Q.........S.F..I...H...TY.....T.I..TSYSR..S.-R...HD.....GD...Q..N...T. 548
Dre_COG7    ......Y..EDT.TSEV.T.........V.V.........Q..A.....S.K..GR..RF.WEGFN....T.L..GV-AERRSQ-...........QS.T...NN.L.T. 547
Gac_COG7    .........EET.SA.A...........V...........Q..A.....S.K..G.......E.Y.......I..ASA.ER.STTR..........RG.MT..N....V. 549
Ola_COG7    ..V......EDA.SSAA...........V...........Q..A.....S.K..........E.Y.....T.I..ASS.ERR.AT...........RG.V...NN...V. 549
Tni_COG7    --------------------.LFCVAFC-...S.......Q..A.....SE....R......E.Y.......I..ASS.ER.STT.-.........RG.M...N....V. 526
Tru_COG7    .........EES.GSFV..........CV...S.......Q..A.....S............E.Y.......I..ASSIER.STT...........RG.M...N....V. 549

Hsa_COG7    YTLKEKGSSNHNLLAAPRAALTRLNQQAHQLAFDSVFLRIKQQLLLISKMDSWNTAGIGETLTDELPAFSLTPLEYISNIGQYIMSLPLNLEPFV-TQEDSALELALHAG 659
Ptr_COG7    ...............................................................................................-.............. 659
Ppy_COG7    ......................................................M.........D..............................-.............. 536
Mac_COG7    ....................................................R...........D..............................-.............. 625
```

```
Cja_COG7    ................S...............................................D..............................-.............. 659
Mmu_COG7    ..............S.S.T...........................V.R...............D..............................-.............. 659
Rno_COG7    ..............SVS.T...........................V.R...............D..............................-.............. 659
Ocu_COG7    ..................T............................P................D..............................-.............. 659
Eca_COG7    ..............S.S...............................................D..T..........H................-.............. 659
Cfa_COG7    ..............S.S.I...........................V............D....D..T...........................-.............. 659
Bta_COG7    ...........H..S.S.S............................P................D..T...........................-.............. 659
Mdo_COG7    ..............STS.M...............................E.............D..T......-....................-.............. 657
Tgu_COG7    F......T.Q.S..SSS....A....LS............R.....LPR.EG.SSG........D..N.....................H.....-.............. 659
Gga_COG7    .......T......SSS.S..S....L..H................V...E..TSG.V.....ED..N.....................H.....-.............. 658
Aca_COG7    .......T.-....SSS.S..A....S......E............VP..EA.S.G.S..M...D..N...............L.....H.....T.............. 596
Xtr_COG7    .M.....AVSTS....S.S.F..Q..V...........H.......VP..E..SCE.S..M..ED..T...............L.....H.....-.P...T........ 657
Dre_COG7    HS.....TG.SS...ET...........N.........Q.....F.LN.PEARGS.SL......D......S.Q...T...........H.....-....P.......T. 656
Gac_COG7    .S.....TG.SS..TE............N.........Q..H..C.V...ERREAP.F..SY..D..T...S.Q...T...E.L.....H.....-....P...M..... 658
Ola_COG7    .S.....TG.S....E..T.........N.........Q..H..G.V...EIQEAS....NF.ED..T...S.Q...T.....L.....H.....-....P...M..... 658
Tni_COG7    .S.....TG.SS...E..S.........N.........Q..H..C.V...EKHEAP.L..SY.ED..T...S.Q...T.....L.....H.....-....P...M..... 635
Tru_COG7    .S.....TG.S...TE..S.........N.........Q..H..C.V...ETHEPP.F..NY.ED..T...S.Q...T.....L.....H.....-....P...M..... 658

Hsa_COG7    KLPFPPEQGDELPELDNMADNWLGSIARATMQTYCDAILQIPELSPHSAKQLATDIDYLINVMDALGLQPSRTLQHIVTLLKTRPEDYRQVSKGLPRRLATTVATMRSVN 769
Ptr_COG7    .............................................................................................................. 769
Ppy_COG7    .............................................................................A..........................A..... 646
Mac_COG7    .............................................................................................................. 735
Cja_COG7    .........ERG.--------.VDLM------------------------------------------------------------------------------------ 677
Mmu_COG7    ....................................V......VT...T..........................N.AA...AK..E.............A......G.. 769
Rno_COG7    .........E..........................G.....AVT...T..........................N.A....AK..E.............A......G.. 769
Ocu_COG7    ..........D....................E............T...T......M...................N......SK......A.........A...A..G.T 769
Eca_COG7    ....................................V.......T...T..........................N......AK................A...A....D 769
Cfa_COG7    ....................................V.......T...T..........................N.......K....................A..N.D 769
Bta_COG7    ............................................T...T..........................N..M...AK.........................D 769
Mdo_COG7    ....................Y...............V.......T...T..........................NM......K.......A........SK..S..N.. 767
Tgu_COG7    ...Y.....E.......V..Y........-.....EV.....Q.T...T..........................N......AK..EF..AA.S....M.AAI.A..GLE 768
Gga_COG7    ...Y................Y..............EV.......TV..T...S......................N......AK......AA.SV...MVSSI.A..GLE 768
Aca_COG7    ....................Y....L.........E.......................A...........KA..NT......K....K..A..F.....S...S...LD 706
Xtr_COG7    .........E.T........Y....L..........V....SL.T..TT.....................T...........AK..E..P.A.S...K.SSSI.A...LE 767
Dre_COG7    ...Y......DV...E.T..Y...............V...L....A.HT..........S.........T.....N.....RVK.DE...TA.T.....SA.I.NI.GLE 766
Gac_COG7    R.........D......T..Y..................L..Q..AR.T..........S............G....AA..RAK......TA.L.....VA.I.AV.GID 768
Ola_COG7    ..........D......T..Y..................L..Q.T...T..........S.....................RAK.A....TT.L.....SS.I.AL.GID 768
Tni_COG7    .................V..Y...-..............L..Q..TR.T..........S.....................RAK......AA.L......S.I.GL..ID 744
Tru_COG7    ..........D......V..Y..................L..Q..TR.T..........S............N........RAK......TA.L......S.I.AL..ID 768

Hsa_COG7    Y 770
Ptr_COG7    . 770
Ppy_COG7    . 647
Mac_COG7    . 736
Cja_COG7    - 677
Mmu_COG7    . 770
Rno_COG7    . 770
Ocu_COG7    . 770
Eca_COG7    . 770
Cfa_COG7    . 770
Bta_COG7    . 770
Mdo_COG7    . 768
Tgu_COG7    G 769
Gga_COG7    C 769
Aca_COG7    . 707
Xtr_COG7    . 768
Dre_COG7    . 767
```

```
Gac_COG7    . 769
Ola_COG7    . 769
Tni_COG7    . 745
Tru_COG7    . 769

Hsa_COG8    AALGEVEDEGLLASLFRDRFPEAQWRERPDVGRYLRELSGSGLERLRREPERLAEERAQLLQQTRDLAFANYKTFIRGAECTERIHRLFGDVEASLGRLLDRLPSFQQSC 110
Ptr_COG8    .............................................................................................................. 110
Ppy_COG8    ........................................................................................................G..... 110
Mac_COG8    -------------------------------------------------------------------------------------------------------------- 1
Cja_COG8    ......................................................................................C.........S............. 110
Mmu_COG8    V..........................K...............D...............R............................................R..... 110
Rno_COG8    ...........................K...............D.....................................................H............ 110
Ocu_COG8    ..V.....................................................................................................G..... 110
Eca_COG8    .......................H.................E.................................................................... 110
Cfa_COG8    .............................................................................................S......G......... 110
Bta_COG8    .............................................................................................E...........L.... 110
Mdo_COG8    ......................G.......................................................................A..H............ 110
Tgu_COG8    ------------------------------------PIAPG.---------------------------------------------G..GI.SR.....E...AL.DA. 29
Gga_COG8    ---MAA.E.R...W.---.G.A.AGPGGAELAA.VA..AAL..AE.G...A...A..ERVGAE..R...EH.RA...S....G.AG.G..GI.SR..S..E...AL.DA. 104
Aca_COG8    ---------------------------------..GR...L...A.....G.........GA...A....H......S....AQTR.G.SGT..G.D...A...P.AH.. 77
Xtr_COG8    M.TMD.........V..AS...S-..DS..FAS..L...SF..DK........SD..S.I.........S.......T.....L.Y.D..Q..G.VSH..NK.....E.. 109
Dre_COG8    M.AVD....SI...I.K.S...N-...N..FAA..SQ..AC.VDE.T......S.....I.....E...T.......T.D..QH.YTD.ST..NC.SK..HK....TEK. 109
Gac_COG8    M.AVD....SI...I.K.S..DS-..DN..FSF..S...SF.V.K.S............I.....E...S..Q....T.D...H.Y.D..R..S.VSL...K..G.RER. 109
Ola_COG8    M.AVD....S....M...S..DT-..DN..FAA..S...AC.V.E.S............I.E...E...S..Q....T.D...L.Y.D..R...RVSS..EK..R.GER. 109
Tni_COG8    M.SVD....SI...I.K.T..DS-..DN..FAA..S...SF.V.K.N............I.....E...S..Q....T.....L.Y.D..R..G.VS....K..G.GER. 109
Tru_COG8    M.AVD....SI...I.K.T..DS-..DN..FAA..S...SF.V.K.N............I.....E...S..Q....T.....L.Y.D..R..S.VS....K..G.GER. 109

Hsa_COG8    RNFVKEAEEISSNRRMNSLTLNRHTEILEILEIPQLMDTCVRNSYYEEALELAAYVRRLERKYSSIPVIQGIVNEVRQSMQLMLSQLIQQLRTNIQLPACLRVIGYLRRM 220
Ptr_COG8    .............................................................................................................. 220
Ppy_COG8    ..................................................................................................T........... 220
Mac_COG8    M............................................................................................................. 110
Cja_COG8    .............................................................................................S.............W.. 220
Mmu_COG8    ............S....T...........................H................................................................ 220
Rno_COG8    .................T...........................................................................S................ 220
Ocu_COG8    ...LQ.....GCS....T............................................H..........S..................SG................ 220
Eca_COG8    .................T...................................S..............................T....................F.... 220
Cfa_COG8    .................T.......................................................................................F..C. 220
Bta_COG8    .................T.......................................................................................F..Q. 220
Mdo_COG8    .....D....G...H..T..............................................T............................................. 220
Tgu_COG8    .T.MRD..A.ACS..............................G........T.........H..........Q.....A...............P......L..F.... 139
Gga_COG8    ...MRD..A.ACS..............................R................K............A.....T....NE.........P..T........... 214
Aca_COG8    ...MR...Q.AQS....N.........................G........V.......K.HA.........A.....S....T.........LP............C. 187
Xtr_COG8    .....Q......S....T.........................G................K.H..V.............A....N..L....S................. 219
Dre_COG8    .G.I...D...VS..............................G............K...K.H..L.......H...L.A....N..L....S.S...V........... 219
Gac_COG8    .G.M....Q.GAS..............................G.........G.AK...K.H..L....A..G.....A....N..L....S.S............... 219
Ola_COG8    .S.M......AAS................V.............A............K.....HA.LH......R.....T.......L....S.A...V........... 219
Tni_COG8    .G.M......GAS..............................G............K...K.HTAL.......G.....A....N..L....S.S...I........... 219
Tru_COG8    .G.M......GAS..............................G............K...K.HT.L.......S.....T....N..L....S.S...V........... 219

Hsa_COG8    DVFTEAELRVKFLQARDAWLRSILTAIPNDDPYFHITKTIEASRVHLFDIITQYRAIFSDEDPLLPPAMGEHTVNESAIFHGWVLQKVSQFLQVLETDLYRGIGGHLDSL 330
Ptr_COG8    .........................................................................................................R.... 330
Ppy_COG8    ..........................................C..............................................................R.... 330
```

```
Mac_COG8     ..........................................C.........................V....................................R....  220
Cja_COG8     ..........................................C........................V.........................L.....N....SR....  330
Mmu_COG8     ..........................................C............................Y....G..........I.................R....  330
Rno_COG8     ..........................................C...........................QY....G..........I.................R....  330
Ocu_COG8     ................E...H.......D.............C......V..................A..R........................A..A..V..R....  330
Eca_COG8     .............................E............C........................................................H.....R....  330
Cfa_COG8     .I........................................C........................VSS....D.....Y.....R............H.....R....  330
Bta_COG8     ..........................................C.............................M..........................N....SR....  330
Mdo_COG8     .I.......I...............S..SE......N.....C.........................A..PS.......................S..Q..G.SR....  330
Tgu_COG8     ..LS..................VQAS..DH...V.L......C......VL..........E..A----------.G....S.......E..R..RR..E..V..R....  239
Gga_COG8     .........I.............QAS..E......L...V..C..................E...--.PEQPSL..G............E......R..Q..V.SR....  322
Aca_COG8     .........IT...........V.A...EE...V.V...V..C......VV.......A..E..V.VPGQQEALG.G.L.............LL..R..Q..A..R....  297
Xtr_COG8     .........I.......S....LQNS.........L.....SC..N...V.................PT.TLPIS.G...........TE.....QN..Q..V..R....  329
Dre_COG8     ................GS..........DE...........TC........................--.GQVL..........V.Q.A...ET.NR..H..V.SR....  327
Gac_COG8     ................GS..Q...A.V.E.............C.....................P...D.QAPL..A.......V...AE...T..R..Q..V..R....  329
Ola_COG8     ................ST....V.....D....V.V......C.....................A..PA.QTA...A.......V...AE..ET..R..Q..V..R....  329
Tni_COG8     E...............SS......S.V.E.............C.................D------------.H.A.......V...AE..ET.RR..E..V..R....  317
Tru_COG8     .A..............ST..H...A...E.............C.................D...V...D.QVW...A.......V...AE..ET.....E..V..R....  329

Hsa_COG8     LGQCMYFGLSFSRVGADFRGQLAPVFQRVAISTFQKAIQETVEKFQEEMNSYMLISAPAILGTSNMPAAVPATQPGTLQPPMVLLDFPPLACFLNNILVAFNDLRLCCPV  440
Ptr_COG8     ..............................................................................................................  440
Ppy_COG8     ............P.....P.....................................................Q.......................YSSP....A.....  440
Mac_COG8     ............................M.......................T..............TV.........................................  330
Cja_COG8     ...............................R.......Q......D.....T.M.......S....T..........................................  440
Mmu_COG8     .....................................VE.A.....D..T..T...TA....S..T..T.........................................  440
Rno_COG8     ...............................N.....VE.A.....D..T..T....A....S...............................................  440
Ocu_COG8     ........................................A.....D.....T.VL......S.KL......A.......................V.............  440
Eca_COG8     ........................................A.....D.....T..L......S..L.....V......................................  440
Cfa_COG8     ...............V........................A.....D.....T.........S..L.....V......................................  440
Bta_COG8     ......................V....Q............A.....D.....T...T..V..S.AL...A.V.......................S..............  440
Mdo_COG8     ...........................Q..LN..K..V.DG.....D.....T......A.RSAI-..V..V.E........G...........................  439
Tgu_COG8     ...............V........L.....ADA.GR.VE.A....R......T......V..GTA-AVP...A......................GL............I  348
Gga_COG8     ........................I.....AT..S..VE.A...........T......V..SAA-AVP..SA......................GL............I  431
Aca_COG8     ......................V.I...A.LLA....T..AASR.R...DC.T.V....V..SALPTPP.GQ..........G............G..A...........  407
Xtr_COG8     ...............V......P.I.....AE..A..VH.A.........M.T.....TV..STIT.VPT.TA.....................................  439
Dre_COG8     ........................I..Q..MD..R.....A.D....D..L.T...L.S...STI-.P.I.S..........A.............V.T..........I  436
Gac_COG8     ........................M..Q..GE..GR.V..AAD....D..L.T...L.SV..GTG-.HVA..S..EA..........Q........V.T...........  438
Ola_COG8     ........................M.....AD..RC.V..A.D....D..M.T..AL.SV..GAI-.PTA.GV......................S..S..........L  438
Tni_COG8     ........................L.....EE..CR.V..A.D....D.DM.T.MAL.SV..GTV-.PMA.GA.........S....Q........V.S..........L  426
Tru_COG8     ........................M.....AQ..SR.V..AAD....D..M.T..TL.SV..GTV-.P.A.TS.........S....Q..........S...........  438

Hsa_COG8     ALAQDVTGALEDALAKVTKIILAFHRAEEAAFSSGEQELFVQFCTVFLEDLVPYLNRCLQVLFPPAQIAQTLGIPPTQLSKYGNLGHVNIGAIQEPLAFILPKRETLFTL  550
Ptr_COG8     .........................................................................KRMKI.-------------------------------  519
Ppy_COG8     ..GRH.........VQ..............V..........................................-------------------------------------  513
Mac_COG8     .............................T...R......I.....................................................................  440
Cja_COG8     ........S.........Q................................I................................I........E.............LA.  550
Mmu_COG8     ........T..N..T....T..........V.....H.I......A............................S......H........S...............V.C.  550
Rno_COG8     ..............M....T..........V.....H.I......A............................S......H........S..............AV.C.  550
Ocu_COG8     ....E..R......VQ...T................R........A.....................L...............D....DVS..............MV.S.  550
Eca_COG8     ........T................................................................V..............DVSVV.............V.C.  550
Cfa_COG8     .......RV..........V.............T.......................................V..A............LA.V......L.....LVLC.  550
Bta_COG8     ....E..R......D....V.....................................................................VSVV............PV.C.  550
Mdo_COG8     .......AS.....G...................Q...................................I..V.......F........QVV............AV.L.  549
Tgu_COG8     .......AC.DA...ETATHLVL----------------------------------------.SQREPEA.AL..A.G.PW.---------------------------  391
Gga_COG8     ......AAC.K...G...................GR........AA.....L..................A.....A..QR..H..C.DVD.LR.L..PV..AQ.AELPP  541
Aca_COG8     ..LRR.WEW.PES.TF.A.V........SM....GR.........A.............PL...A..L.....V.....H...S..C.DERPVRDL.SS...EK..----  513
Xtr_COG8     ..TEA..TT.Q...E..V.T..T.Q........MQ.R...I..........T.F.............R..I..VA.AH..R....CSIDVTVL..A.EG...QKD.----  545
```

Dre_COG8 G...E.SRCI.E..I....L..V......S....R.R.......SA.A..M..F.............L.LI..V....V....S..CI.MNTVL...Q.V..QK.PVTPV 546
Gac_COG8 G..HH.ASC.Q...NT.SRQ.V.......S....R.KQ...L..SSYA.Q.L.F.....KL....E.L.LV..VS....HRC.S..SIDLA.VLL..D.L..Q..PEIHV 548
Ola_COG8 G..H...LC.Q..AHM..HQ..G.....A..I..R.A...SR..SAYAD..L.F......L......L.LV..V.AS..VRF.G..CIDVA.VLQ..D.L.SQK.----- 543
Tni_COG8 G...G.ATC.Q.G.S..SRQ.V.......S.L.DR.R...AH..SAYAD..L.F.R...........L.LV..V.....HR.AH..GIDVA.VL...H.L..Q..----- 531
Tru_COG8 G...G..TC..AG.N...HQ.I..Y....S...DR.K...FH..SSYAD..L.F.R...........L.LV..V.....H..SS..GIDVA..L...N.L..Q...SPPE 548

Hsa_COG8 DD 552
Ptr_COG8 -- 519
Ppy_COG8 -- 513
Mac_COG8 .. 442
Cja_COG8 .. 552
Mmu_COG8 .E 552
Rno_COG8 .. 552
Ocu_COG8 .G 552
Eca_COG8 .E 552
Cfa_COG8 E. 552
Bta_COG8 .K 552
Mdo_COG8 .E 551
Tgu_COG8 -- 391
Gga_COG8 GE 543
Aca_COG8 -- 513
Xtr_COG8 -- 545
Dre_COG8 L. 548
Gac_COG8 TS 550
Ola_COG8 -- 543
Tni_COG8 -- 531
Tru_COG8 L. 550
