# Supplementary material for: Comparative analyses of the Conserved Oligomeric Golgi (COG) complex in vertebrates
Source: BMC Evol Biol. 2010 Jul 15;10:212. doi: 10.1186/1471-2148-10-212 (PMC2927914; doi:10.1186/1471-2148-10-212)
Supplement: Additional file 3 — Protein-coding sequences alignments used in the different analyses performed with PAML. [file 1471-2148-10-212-S3.PDF]

COG1

```
Hsa  ATGGCCACCGCGGCAACCTCACCCGCGCTGAAGCGGCTGGATCTGCGCGACCCTGCGGCTCTTTTCGAGACGCATGGAGCGGAGGAGATCCGCGGGCTGGAGCGCCAGGT 110
Ptr  ........................T.................................................................................G... 110
Ppy  ..........G................................................................................................... 110
Mac  .....GG....T..C.........................................................................................T..... 110
Cja  .....GG....A........T..A.....A.......................C..........................A..........................A.. 110
Mmu  .....GG.....A.CG....CT.......C....................T..CAAC..G..C........T...................................... 110
Rno  .....GG.G..TA.CG....CT....................T.......T..CAAC..A..C..T.....T..............A....................... 110
Ocu  -------------------------------------------------------------------------------------------------------------- 1
Eca  .....GG.......CG....C................................C.....G...........A.......................A.............. 110
Cfa  TCCCGACAG.GCTGT.AGGGG...CT.GAA...ATCT..CCA.ACA.A.GTGA..TCC.CAAA.G.AGA.AAGGCATTC.AC..AG.C.GG..A.C..AAC..CAACACA 110
Bta  .....GG.......CG....CT.G..T......A.......C..C........C.....G..C........C..C..C................................ 110
Mdo  GC..TAG.AAT...CGG...G.AA..T........T..A..G.....G...G.C.AA..G..A..T...G.A...........A.................A..G..... 110
Tgu  CCC.GTG...GT..CGGTGTGG.G...GC..T.GC.GC.CGCGGCGC...GG.C.A...G..G......G....CACG....CC...C.G..G.C.G.......G.G.C. 110
Gga  ------------------------------.T.GC.GC.CGGGGCGCG..GG.C.A......C........T..CAC.....C....C.G....A.GC......G.G.C. 80

Hsa  TCGGGCCGAGATCGAGCACAAGAAGGAGGAGCTGCGGCAGATGGTGGGCGAACGGTACCGCGACCTGATCGAGGCGGCCGACACCATCGGCCAGATGCGCCGCTGCGCCG 220
Ptr  .............................................................................................................. 220
Ppy  .........................................................................................................T..T. 220
Mac  .............................................................................................................. 220
Cja  G..................................................G..........................T.............................G. 220
Mmu  C..A...........A...................................G..........................G............................... 220
Rno  ...A...............................................G..............A...........G........T....................G. 220
Ocu  ----------------------------------------------------------------------------------------------...G.GGAG..G..GC 16
Eca  ...................................................G.......................................................... 220
Cfa  A..TCTGAC..........................T...............G......................................G................... 220
Bta  G..C...............................................G...........T.............................................. 220
Mdo  A..A..G.......................A....................G..C...................................A........TT......... 220
Tgu  G..C....GC........G..CG...........................G..C.....G.................G..........CGG.......G..GA....T. 220
Gga  G.......GC........G..CG...........................G..C..................................C.G.......G.TGA...... 190

Hsa  TGGGGCTAGTGGACGCCGTGAAGGCCACCGACCAGTACTGCGCCCGCCTCCGCCAGTCGGCCGCGCCCCGGCCACCGCGGGCCCAGCAGCCACAGCAGCCATCCGAGAAG 330
Ptr  .............................................................................................................. 330
Ppy  ...............................T.......................A...................................................... 330
Mac  .......G..............A.....G...................................A...................C......................... 330
Cja  .......G..T........C........G.........................................C...........T.C..................T...... 330
Mmu  A...C..G............C..........T..A.......................C.TG.........GTC..A.......C...........C...G......... 330
Rno  A......G............C..........T..A.....T.................C..G...T.....GTC..A.......C.........T.CC..G......... 330
Ocu  A.------------------------------------------------------------------------..A..TCAG.T.TC...C---------------... 39
Eca  A......G...................................G..T..........................G.......A..C......G.................. 330
Cfa  A......G....................G.........................T.C................G.......A..C...............G......... 330
Bta  C......G.............G...................................................G.......A..C......C.................. 330
Mdo  A....T.GC............G...T..G.......G......G..T.........AGCCA...CA.T.CTGTG.TC.CAAG.ACATCAAGCA.AA.A..C......... 330
Tgu  A.C.C..GC...G.......CG..GGCTGC.G.GCGG.G.T...GC..GG.C.GGCC.C....GCA.GGT.AGGGGC..C.GG....GC..CTC.G.A.....TC.AGGT 330
Gga  A.C....GC...G.T.....CG..G.CTGC.G.GCGG.G.G.TGACG.GG.C.GGCC.C..TC....GGCT..G.......TG...G..AAG.T.T.T..GGG..GC.CA 300

Hsa  TTCTACAGCATGGCTGCCCAGATCAAGCTACTCTTAGAAATTCCGGAGAAGATCTGGAGCTCGATGGAAGCCTCTCAGTGTCTCCACGCCACACAGCTCTACCTGCTCTG 440
Ptr  .............................................................................................................. 440
Ppy  .............................G..............A.........................................T....................... 440
Mac  ............................................T..................................A......T...................G.. 440
Cja  .............................C.....G........C..................................A......T....................... 440
Mmu  ..............C........T.....G..GC.G..G..C..................G.C...........G...CAC.....G....................... 440
Rno  ..............C........T.....G..GC.G..G..C..A................GC...............CAC.....G....................G.. 440
Ocu  ..............C..G...........G...C.G..G..C..C........G......G.C.....G.....G.G..AC...........G................. 149
Eca  .............................C..............T.......................G.....G....A.............................. 440
Cfa  .............................G..............C................................A.A...T..................T....T.. 440
```

```
Bta   .............................T...C.G.....C..T.................C.....G.....G.G..A............C................. 440
Mdo   .....T.............................G..G.....T.....A...........C................AC..A..T.....T................. 440
Tgu   ..T...G.GGC...G..G...C.G.....G..GC.G..GG....C...C..G.G...G.GG..G....G.G.GGC.GC.AC..G.C...GG.C.G...GC....A...G. 440
Gga   GCTG.AGCTGCT....GA..TCC.CGAGCGGG.G.G..GCGC------------------------C.T.GAGGC.G.GCGA.A.CT...CGC.GC..ACCTGCTG.GCC 386

Hsa   CTGCCACCTCCACAGCCTGCTCCAGCTGGATTCTTCTAGTTCCCGATACAGTCCCGTCCTCTCCCGGTTTCCTATACTCATCCGGCAGGTGGCAGCCGCCAGCCACTTCC 550
Ptr   ................................................................................................T............. 550
Ppy   .............................................................................................................. 550
Mac   .........T....A.................................................................................T............. 550
Cja   T........T........T...........................C.......T.........................................T............. 550
Mmu   .........G..T........G........C..C....AC...A.......C...A.......GA.A.....A..C........C...........A............. 550
Rno   .........G..T........G........C..C.....C...A.......C...A.......GA.A.....C......G....C...........A............. 259
Ocu   .C.......G...........G........GG.GG.GGCC.....CAG...C..G......G.G........C..C...G................A............. 259
Eca   .........T....AT.................C...G......A..G...C..............A..C..C.......................T............. 550
Cfa   .........T....A.......................C.........T..C..T........A..A..C..............T.....A.....A........T.... 550
Bta   ....T.........AG.....G........G.....C.........C....C..T..............C..C.......................T............. 550
Mdo   ...T.....T..TG.........G......C..C..C...GGAT.T..T..C..T..T..TG....T..C..G.....GG.T..T...........A.....T....... 550
Tgu   GGCT.....G.G.C...A...G........CA.CC.CC.AG....CGC...C...A.....G....C..C..C..C..GC.G..C..A.....G..T.........C..A 550
Gga   GCCA.CTGCA.GGCCTGCTGCAGCT.GACGCG.CC.GC.CC.G---CTACAG...CGT.CTCG..C.C....C..C..GC.G..C........C...............A 493

Hsa   GGTCAACTATTCTGCATGAAAGCAAGATGTTGCTCAAATGCCAAGGTGTGTCTGACCAAGCTGTGGCCGAGGCCCTGTGCTCTATAATGCTCTTAGAAGAGAGTTCTCCT 660
Ptr   .............................................C................................................................ 660
Ppy   .............................................CC............................................................... 660
Mac   .............................................CC............................................................... 660
Cja   .............C...............................CCA...................T........A................................. 660
Mmu   ...........T....C............C.............G.C......G.....G...........A.....C.....C...........G..G.....C.....G 660
Rno   ...........T....C............C.......G.....G.C......G.....G........T.....T..C.....C...........G..G.....C.....C 660
Ocu   ..------------------------------------....CT..A.ACGTCAG.ACG.TCACC..G..---------------------------------------- 294
Eca   .......C.....................C.......G.......CC.................C..T...............G.G........................ 660
Cfa   ......................T......C...............CC.................A..T...........TG............................A 660
Bta   .......C..C........G.........C...............C............G..........................C........G..............G 660
Mdo   .A.....C..C..........C.......A.A..T.G........C............G.....T..A......T...C...AG.C......C.......T......... 660
Tgu   .A..C..C..C.....G..G......GCCC....GCGC....CCACG.GC..G.....G..G.....A..A........TG.C..C.....GC.G..G..C..C..G..A 660
Gga   ....C..C..CT....G..G......TCTC....G..G.C...GACA.....A.....G..G..A..A..A...T....TG.C..C.....TC.G..G..T..C..C..A 603

Hsa   CGCCAAGCCCTCACAGACTTCCTGCTGGCCAGAAAGGCAACTATTCAGAAACTTCTCAACCAGCCACACCATGGTGCTGGTATCAAGGCTCAGATTTGCTCATTAGTGGA 770
Ptr   .............................................................................................................. 770
Ppy   ...............................................................................................C.............. 770
Mac   .............................................................................................................. 770
Cja   .....G....................A..........................A.........................................C.............. 770
Mmu   ......................................G..........CG..C..G......T.T..T..C........C........C...........T..G..... 770
Rno   ..G.....................T........................C...C..G........T..T..C........C........C.....A.....T..G..... 770
Ocu   ---------------------------------------------------------G.G..AAGC..GGC...CCGA-------------------------------- 315
Eca   .....G......G.C.........T..............G..................G........T..................................C....... 770
Cfa   ...........A...........AT..............G....A.......................T........CA.C........C.................... 770
Bta   ...........A...........................G.A..C....................T.....C........G.....................C.G..... 770
Mdo   .....G...........T...T..T.A..T.....A...G.C......C.T........T.......................T..A...........T........... 770
Tgu   .....G.....GG.T..T..............G..ACTGG.C..C...C.G..G.................C..C..A..A.....A......G.G..T..CC.GA.... 770
Gga   .....G......G..........T........G..ACTGG.A..C..AC.G....................C.....A.....A..A......G.G..T...C.GA.... 713

Hsa   GTTGCTGGCCACCACTCTGAAGCAAGCTCATGCCCTTTTCTACACTTTGCCAGAAGGACTGCTGCCAGATCCAGCCCTGCCATGTGGCTTGCTCTTCTCTACTCTGGAGA 880
Ptr   .............................................................................................................. 880
Ppy   .............................................................................................................. 880
Mac   ........................................................................G........G............................ 880
Cja   .C..............G.....................................................................................C.....A. 880
Mmu   .C.......T...........C..G..A.................CC.A........CG.......C..C...T.......C..............T.....C....... 880
Rno   .C.......T...........C.....A.................GC..........TG....A..C..C..CT.......C....................C....... 880
```

```
Ocu  -------------------------------------------------------------------------------------------------------------- 315
Eca  ...A........T...T....C.............................G.....C...............T..............C..........G.......... 880
Cfa  .C..T................C...........T..............A........T.A.............T.........................A.......... 880
Bta  .C...................C.....A.....T..C.........C.A.....G..G..........C..CTTG.....G......C..........C..G.....C. 880
Mdo  .C.................T.C.....C........C........C..A..C.....TG.......T........A..............A.....T..C..C.....TT 880
Tgu  .C....CAG......G...T.C..G..C..C..T..C........CG....C..G..GA..GCC..G..G...........C.....A...........C..C....... 880
Gga  .C....AA....T..C...T.C..G........A..C.......TGA....T.....GG...CA...............A..C........A........C..C....... 823

Hsa  CCATCACAGGCCAGCATCCTGCCGGAAAGGGCGGTGTCCTGCAGGAAGAGATGAAACTCTGCAGCTGGTTTAAACACCTGCCAGCATCCATCGTCGAGTTCCAGCCAACA 990
Ptr  .............................................................................................................. 990
Ppy  .T............................................................................................................ 990
Mac  ..................................................................................G........................... 990
Cja  .......G............A.T.....A...................G........................................G.................... 990
Mmu  .AG.....A.G........CA.T.............C...C..A.GG........G..A...........C.G.........CA.C.....TA.............C... 990
Rno  .GG.....A.G........CA.T.........A...C...A..A.GG........G..G...........C.G............C...G.TAC.............G.. 990
Ocu  --------------------.AAC.G........C........A.GG........G..G...........C.GG........G..G...G....G............G.. 405
Eca  ....................A...........A....T.....A..G........G......G.......C..............G...G.G..G...........GG.G 990
Cfa  ....................A.............C..T.....A..G........G..............C...T....................C...........G.T 990
Bta  G......CA.......C...AG......A..TT.G..T........C........G..G...........C...........C..G...G................GG.T 990
Mdo  .TG.T...A.......G.T...A.....A...ATCAATG.CTTAC..........GT.G...........C........C.....C......A.G............... 990
Tgu  G..G...G......A.C..C..A..G.GA..A..G..G...G....G..TC....G..GA..........C..GT.......T.AG...G.G..G..A.........G.C 990
Gga  G..CT...........G..A..A..G..A..T..G..T...G....T...G....G..GA..........C.GGT....C....AG..TG.A..G..............T 933

Hsa  CTCCGAACCCTTGCACATCCCATCAGTCAGGAATACCTGAAAGACACGCTGCAGAAATGGATCCACATGTGTAATGAAGACATTAAAAATGGGATCACCAACCTGCTCAT 1100
Ptr  .............................................................................................................. 1100
Ppy  ..........................C................................................................................... 1100
Mac  ........T..C..............C....................A..........................C..................G................ 1100
Cja  ...............T..........C..............G.....A........................C...............................A..... 1100
Mmu  ........T..C..............C.....G..............................G.......C.........................GG..T.....T.. 1100
Rno  ..G........C..............C.....G........G.G........G...G......G.......C.G.......................GG..T.....T.. 1100
Ocu  ..G..C.....C..G...........C.....G.......G....T.C........G...G.............G..............C..........G......... 515
Eca  .....C.....C..G..C........C.....G......CGG..............G........TG......................C.................T.A 1100
Cfa  ........A..C..G..G........C....................C...........................................................TT. 1100
Bta  .....G..G..G..G...........C.....G.......................G.........G................C.G...C.......G..G......G.. 1100
Mdo  ..T.....T........C.......A.G....G...T..........T..A..A.........A.T......C.......T..C....C............TT....GG. 1100
Tgu  ..G..G.....C..G...........C.....G.....C.GG..G..C......C.G......A.......C.G...T.....C.GG.CG...G...GG.G......GG. 1100
Gga  ..G..G.....G.....C........C.....C..T..C.G......A..............TGC......C.G...G..T..C.GGGC...TG...G.........GG. 1043

Hsa  GTACGTGAAGAGCATGAAGGGTCTCGCGGGAATCCGGGACGCCATGTGGGAGTTACTTACCAATGAGTCCACCAATCACAGCTGGGATGTGCTATGTCGGCGGCTTCTGG 1210
Ptr  .............................................................................................................. 1210
Ppy  .............................................................................................................. 1210
Mac  .....................C....................T..............C............G......................G................ 1210
Cja  ...........................A..G............................................................A.................. 1210
Mmu  ........................G..A..C...A.A........A.....T..G....G...C.....TG...GC...........G...G.G....A........... 1210
Rno  ..................A.....G..A..C...A.A........A.....T..G........C.....TG...GC...........G...G.G....A........... 1210
Ocu  ......C.............CG..G..T.CC............G.......CC.G..C...C.C...G..G...GC...........C...A.C..C........G.... 625
Eca  ....................C......A..C.....................C.T....A..TG.........GCC...G.....ACC...G.G..C........C.... 1210
Cfa  ...T..............A.....T..A..T.....A..T..T........A..............A........................A......A......G..A. 1210
Bta  ........C.....C......G..G.....G.....A......G......G......CG....C.....G.T.C.................G.C..C........C.... 1210
Mdo  T.....C..A...C....A..C..T..A..G..T........TG.A......C.G..C....G...A..A.TG.GC..A........G...G.G..C.....C....... 1210
Tgu  .....................G..G..A..G.....T..T...G........C.G..GT...GC...G...G..GC....A......G.CCG.G..C........G.... 1210
Gga  C..T.........C....A..C.....A..G..T..T..T..AG........C.G..C..A.G........TA.G...G.A..........T.G..C..T..C...T... 1153

Hsa  AGAAGCCGCTCTTGTTCTGGGAAGATATGATGCAGCAACTGTTCCTTGACCGATTACAGACTCTGACAAAAGAAGGCTTTGACTCCATCTCCAGTAGCTCCAAGGAGCTC 1320
Ptr  .............................................................................................................. 1320
Ppy  .............................................................................................................. 1320
Mac  ..........T...........................................................G..............T........................ 1320
```

```
Cja  .A..........................A................................................................................. 1320
Mmu  ......................G..CC..........G..C.....G..T..G.........A.....C.G............A..........AC........A.....T 1320
Rno  ......................G..CC..........G..C.....G..T..G.........G.....C.G........................C........A...... 1320
Ocu  ..C....A....C.........G..C...........G........G.....GC.G.....G.....G.G.........C.........G....C....G.G.A...... 735
Eca  .......C...C..........G..CC.........GC..C..............G...........G...........C............GC...T....GA...... 1320
Cfa  ......................G...T..........G.......................................................C............A... 1320
Bta  .C....................G..CC..........G..............GC.G...........C...........C..............C..T....CA...... 1320
Mdo  .T.....CA...CC...........CT..C....A..G.......................A.....G.G...T.................T..C........AA....T 1320
Tgu  .C.G...TG.G.CC........G..GC..C.....G.G........G...A.GC.G...........C...........C...........A..C.........C....G 1320
Gga  .C.....TGCT.CC........G..CC..C...G...G..C.....G...A.GC.GG...TA.....T........A.....A...G....A..C........AC....T 1263

Hsa  TTGGTTTCAGCTTTGCAGGAACTTGAAAGCAGCACCAGCCCTTCAAATAAGCACATCCACTTTGAGTACAACATGTCGCTCTTCCTCTGGTCTGAGAGTCCTAATGACCT 1430
Ptr  .............................................................................................................. 1430
Ppy  C......................C...................................................................................... 1430
Mac  C...........................................................................................C................. 1430
Cja  C......................G...............T.......C..................C........................................... 1430
Mmu  C.A..C......C.......G..G..G.C..A..A.TC.A.A..G..C.....TG...........C.G........TT.............C.....C..C..C..... 1430
Rno  C.A..C.................G..GGC..A..A.TC.A.A..G........TG...........C.G........T....A.........C.....C..C..C..... 1430
Ocu  C...C...............G..G..G..TG...G.G..G...GC.G.......G......AC...C.G.......GC..G.....G.....C.....CG.CGGC..... 845
Eca  C.C.........C..........G...............A.CC.......................C.T......G.T..............C......T.C.G...... 1430
Cfa  C.CA.......CC.......G.....G.A..C.......A..........................C..........T..............C.....C..CC.C..... 1430
Bta  C.CACC.....C........G.............G.TCGG......GC......C.......C...C........G.CG.............C.....C..C.GC..... 1430
Mdo  C.......T..................---GC..GTTC.T.CC.C..C.....GG....G......C..........T.....T........A..........TC...T. 1427
Tgu  C...C.GG...C........GT.G...GTG.AGG..G..G.CCTG.GC.....G.....GCAG..AC.....G..G.C..G.....G.....G.....C..CGGG..... 1430
Gga  C.CA.C.T...CC..............GC..AAT...A.G.C.TT.GC...........G.....AC........G.C.AG.....G.....A.....CT.C.GC..T.. 1373

Hsa  GCCTTCCGATGCGGCCTGGGTCAGCGTGGCAAACCGGGGTCAGTTTGCCAGTAGCGGCCTCTCCATGAAAGCACAAGCCATCAGCCCTTGTGTACAGAACTTCTGTTCTG 1540
Ptr  .............................................................................................................. 1540
Ppy  .......................A...................................................................................... 1540
Mac  .........C.......................................................................T............................ 1540
Cja  A...........T..........CT.............T...........A..............................T.....A.................C.... 1540
Mmu  ......T.....T..............A..........C...............T.................C........T.....G.....C................ 1540
Rno  ......T.....T..............A.....T....C...........AC..T.................C.....A..............C..............G. 1540
Ocu  ...CC....C..C........G.C......C.......C..C.GC....G.C.................G..C.GG...G.G.....C..C..G...GG......C..C. 955
Eca  ....C.T.....C..........A......G............C.G..TG....T..G..............T..G..TG.G.....G.....C................ 1540
Cfa  ....C.T.....T........T.....A..GC.......G...........C..T..G.................G.....T...........C...........C.... 1540
Bta  ...C..T..C..C...............T.C.G.....C..CA...CAGG.C..T..G...........G.....G.....T........C..G....G......C.... 1540
Mdo  ......A.....T..A.......A......G..T...A.C....G......C..T.................C..G...C.......C.A........GT..T..CCT.. 1537
Tgu  ...CGGG..C.....G.............GGC.G..C..G.CC..C.....G.....G..GG.......G.....G..GC...CG..G..C..G....G......C..CA 1540
Gga  ......A.....T..T.......A............CA.C..........AG........G........G..C..G..AC...CA..A..CA......G......C..A. 1483

Hsa  CCCTGGATTCTAAGCTGAAGGTTAAACTAGATGACCTCCTGGCTTACCTCCCCTCTGATGACTCATCACTGCCCAAGGACGTTTCTCCCACACAGGCCAAGAGTTCTGCC 1650
Ptr  .............................................................................................................. 1650
Ppy  ................................................................G............................................. 1650
Mac  ................................................................G......................................C.....T 1650
Cja  ...................................A...........................................T...............A.......C...... 1650
Mmu  ............................G..C...........C.....T.....CAG....A..C..T...T......TACCA.C.....G...C.T....AC...... 1650
Rno  ..........G.................G..C........C..C.....T.....CAG....A.CC..T...T.......ACCA.C.A.........A....CC..C..G 1650
Ocu  ....A...G.C..............G..G..............C...........C..G..GAGCC.C.C...G------------------..........AC...... 1047
Eca  ....T........A............T.G..G......A..........T.......G......G.....TT.T.....T..C..C...T.G..A..G.G...C.G.... 1650
Cfa  ....C........A........G.....G..C.................T...............C.C........A..T..C.TC...GGG...........C.G.... 1650
Bta  ..........A..A........G.....G..G...........C.............G....C.G.TG.CC.G.C.....ACC..C..TG.GGCA...G....C.GC... 1650
Mdo  .T.....C..C..A.....A..C.....G........TT..T.G.....T.....A....CAC.CAAGGA.G..TC..GT------------...A......AC...... 1635
Tgu  .G.....C..CC.........CC.GG..G..G..TG.....T.C.........GGG..C...---..TG.C........G------...G.G.CAC.GCGCTCC---... 1638
Gga  .T.....C..A........A.CC.GGT.G.............T.C.....T..TG.A..GTCT---....CCA.......GC.CA.....C.C...C.TCGCTCA---T.. 1587

Hsa  TTTGACAGATACGCAGATGCGGGGACCGTGCAGGAGATGCTGCGGACTCAGTCCGTGGCATGCATCAAGCACATCGTGGACTGCATCCGGGCAGAGCTACAGAGCATTGA 1760
```

```
Ptr        .............................................................................................................. 1760
Ppy        .............................................................C................................................ 1760
Mac        .................C..A.....T.................C.....................................................G........... 1760
Cja        ..............G.....T...G.G..........................T....TC......................................G..A.....C.. 1760
Mmu        .....TC.............T..............C........T..G...........C.........TC.G......G........A.........GTGC.C...... 1760
Rno        .....T..G..T.....C..T.....T........C........C..A...........C.........TC.G.T....G........A.........T.GAGT...... 1760
Ocu        ........G.....G..C.....C.......GC..CC........G.G.......C..TG......GGC.G....AC..G.C......A...G.....G.GA........ 1157
Eca        ........G.....T.....T..............C.......A...C..C..G.....G......C........AC..............TG.....G....A...... 1760
Cfa        ........G...........C......................A......C.......TG.........T.....ACA..........A.........G..A.....C.. 1760
Bta        ..C.......TT........A.A...G..........CA........G..C...AC...C......CG.G.....AC...........A...G.....G........C.. 1760
Mdo        .....T..G...T.......CA.C..T...G...GA.....C....AC..C.GT.C...T....CTG....G..T....CG...G.G..T........G..AG.GGC... 1745
Tgu        ..C...C.C.T...C..C..C..C..........G.C....C..CGAG.GC.G...C..C...G..C.....C..C...G....G...A..AGC....C......GCCC. 1748
Gga        ..C...C.C..T..T..CA.CA.C.TG...G.A.G.C....C..TGA...C.G.A.T..C......C.C...G.GC..AG...TG.G..T.A......C...G.TGC.C. 1697

Hsa        AGAGGGTGTGCAAGGGCAACAGGATGCCCTCAACAGTGCCAAGCTGCACTCAGTTCTTTTCATGGCCAGACTCTGCCAGTCCCTGGGAGAGCTGTGCCCCCATCTGAAGC 1870
Ptr        ...A.......................................................................................................... 1870
Ppy        ...A.......................................................................................................... 1870
Mac        ...A...................................T.C.................................................................... 1870
Cja        ...A.............G........TT....G.....T.........................................................T............. 1870
Mmu        ...A.TCACA.GG.AA...A......T...TC....CA...........G....C..C.......................T.................T..C..A.... 1870
Rno        ...A.TCACA....AC...A......T...TC....CA..........TG.......C................................A........T..C..A.... 1870
Ocu        G....CC...........G....C..T..G..G.G.G.T.C........G.C..C..G...........G....................A...........C..C.... 1267
Eca        ...C.T............G.....C.T.....GTG...T......T...G....C..G................................A.....T............G 1870
Cfa        .C...T......G.....G.......T.......G...T....T.....G.G..C..........................A........A.....T............. 1870
Bta        .C...C.A.T........G.....C.T...TG.G..G..............C.CC..C.........C...................G........T.....C....... 1870
Mdo        G.CA.CAC....G..A.CGACCC.CATG....GTC..C.......CTC......C..G...........G..............T.....A.....T............. 1855
Tgu        GAGC------------..G.T...CC...CTGGGGAC....G...CA..G.C..G..C...........G..G......G.G...CCC...........T..C...C... 1846
Gga        G------------------GC...C..T.C..GTGA.A...G...C..TG.T..C..C..T........G...............A.C.....T..T..T..C..C.... 1789

Hsa        AGTGCATCCTGGGAAAATCAGAGAGCTCAGAGAAACCAGCAAGGGAGTTTAGGGCTCTGAGAAAACAGGGAAAGGTGAAAACTCAGGAAATCATTCCTACACAGGCCAAG 1980
Ptr        .............................................................................................................. 1980
Ppy        ....T......................................................................................................... 1980
Mac        ..........A.......................................C........................................................... 1980
Cja        ...................G....................................................................G..................... 1980
Mmu        ....TG.TG.....C.G.GC.G.G.T..T........T..C......GCG..........A...G.....G....G..G.G.......TG..C.C..CG.G......C.. 1980
Rno        ....T..TG.......G.GT.G.G.T..C..A..G..T.........G.G..........A...G..........G....G....A..TG..C.C..CGTG......C.. 1980
Ocu        ....T...G........C.T.G....C.C...........C......CCCC.........AG...GCA.CC....G...GGG......CGC.GC...C..G......G.. 1377
Eca        ........T..........G.G......C.................T.C...........A..............G...............A........G......... 1980
Cfa        ........T......GG....G......T......A..........TCC...........A..............G........G....G.A.......TG......... 1980
Bta        ...............G...G.G....C.C.....G..C..CCA...C.CCC.........AG........G....G...GG.A.....GG..C.C..C..........G. 1980
Mdo        GA..T..TT....C.....G.GT..T...........CATC......CC.C....CACA.AG.......C...A.GA...G........G.G.A....GTT.TA..A..A 1965
Tgu        GC..TG.G.....CC.GG.G.GCG..G.G...TCGG.GC.C.....ACCGC.CT..GCC.AG..G.T...C....GC...G.C.....GG.GTCC..CGAG.T....... 1956
Gga        ....T........CCGG....GC..TGTG....CGG.CTG..A...AACCC..T.CACC.AG..G.T...G....G....GTC..A...G.G.A....GTG..A...... 1899

Hsa        TGGCAAGAGGTTAAAGAAGTACTCCTCCAGCAGAGCGTGATGGGCTACCAGGTCTGGAGCAGTGCAGTTGTGAAAGTTTTGATTCATGGATTCACCCAGTCATTACTTCT 2090
Ptr        .............................................................................................................. 2090
Ppy        .................................................G............................................................ 2090
Mac        .................................................G...........C................................................ 2090
Cja        .................................................G...........C..TG............................................ 2090
Mmu        .....G.GA..G..G.....C..G.....A........C....C.....G...........CA...C........T..C.....TG.........T.GA...C.G..... 2090
Rno        .....G..A..G..G.....C..G...................C.....G..........GCA...C.C.........C.C...TG............A.....G...T. 2090
Ocu        .....G..A..G.G...GA.G..GT.A........T..CA.........G...G......GCC..GC.C.....G..C....C...GC.G...G..........G..... 1487
Eca        .....G..A..A..G...C.C..............T...G.........G..........TCA...........G......GC.........TG..............T. 2090
Cfa        .....G..A.....G..GC.C..T.........................GC.........TCA...........G......GC...C..............G......T. 2090
Bta        .....G..C..G..G..GC.C.....G....G........C........G.......G..TCG..G..C....GG...C..GC..........G.............CG. 2090
Mdo        .....G..A..C.....GCAG..T..GG.......TC..C....A....G.C.G.......TG..CA.CACA..T...CC.G...G..AT.................... 2075
Tgu        .....G.G...G..G.C..AG.....G.........C..G...C...T...C........TCG..T..CACC....GCC..G.G..GT.C........CA.G..G..G.. 2066
```

```
Gga      .....G..A..G..G.C..A......G.........C..GCT.CT......A........TC...T..CACT....C.C..G....GT.C..T..T..AA....G..G.. 2009

Hsa      AGATGATGCTGGCTCAGTTCTGGCCACAGCCACCAGCTGGGATGAGCTAGAAATTCAGGAGGAGGCAGAGTCTGGCAGCAGTGTCACATCCAAGATCCGACTCCCTGCAC 2200
Ptr      .............................................A................................................................ 2200
Ppy      .....C................................................................................................G....... 2200
Mac      ..................C..............................................................C............................ 2200
Cja      ..................C..............................................................C............................ 2200
Mmu      CAGG.....C........C........T.......AT........A...........A......A......................G..T........C......A.T. 2200
Rno      .AG.........T.....C.....T..T........T........A...........A......A..........T..............A........C.....CA.G. 2200
Ocu      ......C........C..C........G.................A.....G............A.C.........G.T..CA.........C......G..G...A... 1597
Eca      ..................G.....T..G.................A.................A.............A...CA........................TC. 2200
Cfa      ..................C..........................A...........A.....A..G..............C..........CA............AT.. 2200
Bta      ...C...........CA.C..........................A..G.....C........A..G..............CA....G.....A.............T.. 2200
Mdo      ..GGA..........TA.C..A....TG.......A.........A..G.....C.........A....AA....G........A...........T.....G....T.. 2185
Tgu      C..CACA..C..T..C..C........G..................A.T.....C.........AGC........G.....C..G..G...........G..G....TG. 2176
Gga      ...CACA.....T..T..CT....TG.........A..........A.T.................T........A.A......A.....T........G..G...ATG. 2119

Hsa      AGCCGTCCTGGTATGTACAGTCCTTCCTGTTTAGTTTATGCCAGGAAATTAATCGGGTTGGAGGCCATGCCTTGCCAAAGGTGACATTACAGGAGATGCTGAAAAGCTGT 2310
Ptr      .............................................................................................................. 2310
Ppy      ....A......................................................................................A.................. 2310
Mac      ....A..........................................G...................C.......................................... 2310
Cja      .................................................G................................A...G....................... 2310
Mmu      ....C.............................C............G......................T...........C..CC................G.C...C 2310
Rno      .............C.................C..C............G................................A.T..CC................G.C...C 2310
Ocu      .............C..G..............C..CC.C........G.....C.....C..G..............G........GC.G.............GG...... 1707
Eca      ....A..T.....C..G.................C...................................................C.G..................... 2310
Cfa      ....A........C..C.................CC.T..........................T.....T........A......C.G..A.................. 2310
Bta      ................G.................C..G...........C.................C..................C.G....................C 2310
Mdo      ....A..............A........C..C...C.G..T..A.....A....A................C.........CC...C.............A..GG....C 2295
Tgu      ....C..G...C.C..G....G.C.......C..CC.G........GG.G..CA.......T..G..CA..C....C.....C..GC.G......C......GG.....C 2286
Gga      ....A........C..C....G.C....C..C.ACC.G.....A..GG.G..C..A..C..C..T..CA.TC..........C..C..G......C.......GGC...C 2229

Hsa      ATGGTTCAAGTAGTAGCTGCCTATGAGAAACTCTCCGAAGAAAAACAGATTAAGAAAGAAGGTGCATTTCCAGTCACCCAGAACCGGGCGCTGCAGCTGCTTTATGATCT 2420
Ptr      .............................................................................................................. 2420
Ppy      ................................T.TT.......................................................................... 2420
Mac      ................................T..T.............G............................................................ 2420
Cja      ................................TG.........................C...A.............................................. 2420
Mmu      ....CC.....CA..............C.....A.A..G.....C.....A...........G.........A.G...........A..C........C..C..C..C.. 2420
Rno      ....CC.....CA.C..C.........C.G...A.A..G.....C.....A...........GA........A.G...........A..C........C..C.....C.. 2420
Ocu      ....CC..C.....G........C...C.G...G.G.G...GC.G....GG.....G........G..C.....G........T.....A...........G..C..C.. 1817
Eca      .........A......................TC.A..............G.....G........G......A.G........T.....A.................... 2420
Cfa      .....C.....G..G.................TG.A...........TG.G..............C..C..GA.G........T.....C.....A.............. 2420
Bta      C...GC.G.....CT..C.....C....GG.....G....CC..G...C.C...........C..C......A.G........T.....A...........C........ 2420
Mdo      C...........T....G.G......A..G..T..T.....T..G.CAGC.............A....C...A.G..T......A....A.....A..A........... 2405
Tgu      ....CAG.G..GC.C..........GA..G..GGTG..GC.G..G...GAG....GGCCG.ACAGC..C..CC.G.......G.A....T......T.A..C..C..C.. 2396
Gga      ....CAG....GC.T...........A..G...ATG..C..G..G...GAC.......C...CA.C..C..CA.G..A......A.A..T........A..C..C..... 2339

Hsa      GCGTTACCTCAACATTGTTCTGACAGCCAAGGGTGACGAGGTGAAGAGTGGCCGGAGCAAGCCAGACTCCAGAATTGAGAAAGTGACTGACCACCTGGAAGCCCTCATTG 2530
Ptr      ........................G..................................................................................... 2530
Ppy      ................................C............................................................................. 2530
Mac      ................................C............................................................................. 2530
Cja      ...............................AC......................................................G...................... 2530
Mmu      A..C.......C...G.......GCAG......G..G........................G.............G.....GA....C..GAGG.....G.......... 2530
Rno      A..C....................CAG......G..G...........CA...........T.C...........A.....GA..G.....AGG.....G.......... 2530
Ocu      ...C.......G.C.C..C.................A....G............A.....A..G........G........G..C......T.........GA....... 1927
Eca      ......T........C........C........C..A...........C.....C.......AG.........T...........G.....T.........G........ 2530
Cfa      ......T......................G.AC...G........A........C.....A.AG.....................G.....T.T.....G..A....... 2530
```

```
Bta          ...........GTG.A..C................A..A...A..C.....CT...GA.AG.........G...........G.....T........A.G.....C. 2530
Mdo          ......T..........G.......T....AC...A.....A..A....CT..A..T..A.AG.............A.....A..A...TT.T.A....GT.....A. 2515
Tgu          .A.G...G...G...CA.C..TGACAGT...A....G..CACA..ACCC...A....T.....AG....G...G........G..C.TC...TT......G.GA.A...C. 2506
Gga          ...G.....G..T..CA.CT.......G...A....G..A.CA..A.CCA..A....T.....AC........G........G.........TT......G.GA.A...C. 2449

Hsa          ATCCATTTGACCTGGACGTTTTCACGCCACACCTCAACAGCAACCTTCATCGCCTGGTGCAGCGAACTTCTGTTCTGTTTGGATTGGTGACTGGTACAGAGAATCAGCTC 2640
Ptr          .............................................................................................................. 2640
Ppy          ....G......................................................................................................... 2640
Mac          ........................A........T..........................A.............T.................................. 2640
Cja          ..........T....T..........G.......................T................C......................................... 2640
Mmu          .C..T...........T..C.....A..G..T..............CA.C..............G.....A.....A.....C.....T..A..C............T.T 2640
Rno          .C..T...........T.......A..G..T..............CA.....................A.....A.....G..........................T.. 2640
Ocu          .C..G...........T..G.........................ACC................C.....C.........C....T........G.........T.. 2037
Eca          .C..G...........T.......A.....T.....T.........A............A...................C....T.....CC...........T.. 2640
Cfa          .C..G...........T.......A.....................A.......A.....A..........C..A...........C.....G..........TT.. 2640
Bta          .C......................C..C.....TC.........CA.C.....C........G..C..C.....C.......C.......A...C.G.......C...T.. 2640
Mdo          ............A..T..C..T..A........A..T.........A............A.....C..A................C.A.....G.....T......T.T 2625
Tgu          .C..C..C........T..G.....C..T.....G...........GA.C............A.....C..G.....C.....CC..C.......G........C...TA. 2616
Gga          ..........T....T.....T..A........A............A..........T........C........C.....C...C.......G........C...TA. 2559

Hsa          GCCCCCCGGAGCAGTACGTTCAACTCCCAAGAACCCCATAACATCCTGCCACTGGCATCCAGTCAGATCAGGTTTGGACTTCTCCCACTGAGCATGACAAGCACTCGAAA 2750
Ptr          .............................................................................................................. 2750
Ppy          .......................................................................................C...................... 2750
Mac          ..................................................A.........................................................G..... 2750
Cja          ............A...A..........................A.........C........................................................ 2750
Mmu          ...T.........C..T..........G..G..........A.....G....C.......................C..T...................AA.... 2750
Rno          ...T.........C..............G............A..........C.......................C..T...................AA.... 2750
Ocu          A..T.GA......A.G.AG............GG....C..........C.....................C..G..G..T..C...............AC..C.. 2147
Eca          A....TA..........A....................................G..............C.......T...................C..... 2750
Cfa          A.T...A.....T.C..A.....................T..A.....C.....G..G.....................T.................T..C..... 2750
Bta          A.....A.......................G....C..........G.....G..G..C.............G.....T..G................A..... 2750
Mdo          T.TG............T.....T.....................A..A.........................................T.....T....C...T.....A..... 2735
Tgu          A.GAG.A..G..G.C...C.G.G...G..G..G.T...C...........CT.AT.......C...................G............T.G...T.A...G.. 2726
Gga          ..AAG.A....TG..G.TC..GG......G..G.T......T...T....CT.A........C.........A...........G..G..C...T...T....TT.A..... 2669

Hsa          GGCTAAATCAACCAGAAACATCGAAACAGCTCAGGTTGTCCCCCCGGCACGCTCCACAGCTGGTGACCCGACAGTTCCTGGCTCCTTGTTCAGACAGCTTGTCAGTGAAG 2860
Ptr          .............................................................................................................. 2860
Ppy          ....................................................................................................CA......... 2860
Mac          ..................G..........C..................A...............................................C.....C.... 2860
Cja          .C...........................C.........T........T.....................C.....................CA......... 2860
Mmu          ...G.GGG.........G.G.T.....C..C.......G...........T.....G..TA...........GCA............A.....G.......CA..C..G. 2860
Rno          ...C.GGG.....C.GGG........GCA.C.......G...........T.....G..TA........A..GCA.........TC.A..............CA.....G. 2860
Ocu          ......GCTG.......G.G..C....C..C....C..A......A..G.T....CG...G.AG.......TGCACG.C.......C.............C..G..C..G. 2257
Eca          .A.C.............G.........C..C....CG............T.....CG...A.A.......A.TGCAG...............................G. 2860
Cfa          ...C.............GT..G........C.................T....CG...A.........A..GCA...............................G.......G. 2860
Bta          ...C..G...G.TG...G...G..C..C..C....CG.C...G.....G.T....CGG..A.AC.....C.GGCAGG.C.......C.......G.....G...GC...C. 2860
Mdo          ......G..CT.TC...G..CA...T.C.AC.........A.TT..AA...T.C....G...A.A...TGTC.TGCG......T................GC.....CA.... 2845
Tgu          .AGC..G..C..T..G.G..CT....G..TC.......CT..TG.AAGGGCAC.A------.C...GGA.G.GCGG..G.......C.....C.G.....CA..GCC..C. 2830
Gga          .A.......TG.T..G....CA....G..T.G.....CCA..T..T....T.A.A.G...A.AA...GA.G.CCGC.................G.........A.C...G. 2779

Hsa          AAGACAACACGTCTGCACCTTCATTATTCAAACTTGGCTGGCTCTCTAGTATGACTAAG 2919
Ptr          ........................................................... 2919
Ppy          ..................................................C... 2919
Mac          ..................................G..................... 2919
Cja          ........T.........................C..................... 2919
Mmu          .....G..T.AC.................G....C..................C... 2919
Rno          .....GG.T.AC............G.....G..C.C..................C... 2919
```

```
Ocu     .....G..........G..G..G........G...........G..C............ 2316
Eca     ....AG..T.....A...........................................A 2919
Cfa     ....AG..G.A...A.G..............G..A..T....................A 2919
Bta     .G..AG..T..G.C.....C..GC.G........C..G........C..C.....C... 2919
Mdo     .G..AG....A...A.C..A..TCGT....C....A.T......G...AC........A 2904
Tgu     .C..GG.....G.G.....G..CC.C.....G..G...........GG.C.....C... 2889
Gga     .G..AG....A..C........TC.C.....G..G..A.....T...G.C.....C... 2838
```

COG2

```
Hsa     ATGGAGAAAAGTAGGATGAACCTGCCCAAGGGGCCGGACACGCTCTGCTTCGACAAGGACGAGTTCATGAAGGAAGATTTCGATGTCGATCATTTTGTGTCTGACTGTAG 110
Ptr     .............................................................................................................. 110
Ppy     .............................................................................................................. 110
Mac     .............................................................................................................. 110
Cja     .......................A...........A.......................................................................... 110
Mmu     .........C.C..............T.C...............G........T....................G...........G..C..C..............C.. 110
Rno     .........C.C................CC..............G........T.....T..A...........G..C........G..C.................C.. 110
Ocu     ....................TT...........................................TC.......G...........T....................... 110
Eca     ......C.....................G....................................T....................T....................... 110
Cfa     ------------------------------------------------------------------............................................ 44
Bta     ...................................A........G................................C..T...........C...............C. 110
Mdo     ........G.AG.................A........T..A..G.....T.......................T..C........T....................... 110
Tgu     ---------------------A.....CGA.AA..T---T.A------...........T.............CG.....T.....T.....C........A.....C.. 80
Gga     ......GCC..G.A.........C..GCG...C..C..G.AC..............................CCG.....T..C........C..C..A..C..G..... 110

Hsa     GAAGCGGGTCCAGCTGGAAGAACTGAGAGATGACCTGGAGCTCTACTATAAACTTCTTAAAACAGCCATGGTCGAACTCATCAACAAGGATTATGCAGATTTTGTCAATC 220
Ptr     .............................................................................................................. 220
Ppy     .........................................................G.................................................... 220
Mac     .............................................................................................................. 220
Cja     .................................T..........................................................................C. 220
Mmu     .........G........G..G........C...........G..T..C..G..G..G..G..G........G..G..............C..C..C..C........C. 220
Rno     .........G........G..G........C.........T.G..T..C..G..G..C..G...........G..G..............C.....C..C..C.....C. 220
Ocu     .........T..AT.........................A........C............................................C.....C........C. 220
Eca     ......T...............T................A................................T...................................C. 220
Cfa     ......T...........G..........GA..T.....A...................................G........T.......................C. 154
Bta     ......A.............C...C.......................C..G..G.................T..G..............C..C.....C........C. 220
Mdo     ...A....................T........T..T..A.....T.....G....................G..G.....T..T..............C.......... 220
Tgu     ...A..T..G...T.......G..C.....G..T..A........T..C.....C........T..T.....A........A..T.....C..............T.... 190
Gga     ......C..G..AT....G..G..C..G..G..T...........T..C..G..C........G........A..G.....A..T.....C.....G..C.....T.... 220

Hsa     TTTCAACAAACTTGGTTGGCATGGACAAAGCCCTCAACCAGCTTTCTGTGCCTTTGGGACAATTACGAGAAGAGGTTCTGAGCCTTAGATCGTCTGTCAGTGAAGGAATT 330
Ptr     .............................................................................................................. 330
Ppy     ..................................T.....................................................G..................... 330
Mac     ...........C...............................................................................A.................. 330
Cja     ........................................A..................................................A.................. 330
Mmu     .C.........C...............G............A....................G....................T.....G..C.................. 330
Rno     .C.........C....C.............................................C....G..............T..G..G..A..A............... 330
Ocu     ...............................T..A.......................G..G.....G..............T........A.................. 330
Eca     ...................T..............................................................T.....G..A.......T.......... 330
Cfa     ...................G..............T..T..............C........G...........A........T..C..G..A.................C 264
Bta     .G.....T..TC.......T........G.......GT...............C.......G..............G.....T...C.G..A.................. 330
Mdo     ..........T..A.............................C.....A...............A...........A.........AG..AA..........T...... 330
Tgu     .C........TC.A.....T........G..T.....T........A..A..C........G...A.......A..GA....T....AG..A.G................ 300
Gga     .C.........C................G........T........A..A..C........G...A....G..A..AA....T..A..G....G................ 330
```

```
Hsa         CGGGCAGTTGATGAACGAATGTCTAAACAAGAGGACATTAGGAAAAAAAAGATGTGTGTATTGAGGCTTATACAAGTTATTCGGTCAGTTGAGAAAATTGAAAAAATCTT 440
Ptr         ...................................................................................A.......................... 440
Ppy         ................................A............................................................................. 440
Mac         ..................G......................A.........................................A.......................... 440
Cja         ......................G....................................G.................................................. 440
Mmu         .T......A.................G.....A...........G.........A....G.....A.................A..G..............G........ 440
Rno         .T......A..G.....G........G.....T.....C.....G..G......A.C..GC....A...........C.....A..G....................... 440
Ocu         ........A..................................................G.......................A.......................... 440
Eca         ........A..................................................G.......................A.......................... 440
Cfa         .A......A.............G...............A.....G..............G.......................A........A................. 374
Bta         ........A.........C..................................A.....G...C..........G........A.......................... 440
Mdo         ..............G....................T..C..A.G...............T.....A.....T...........C.......................A.. 440
Tgu         .AA.....A.....C..G...A..........A........A.G.........A.....CC....A.....T..T.......A...G..............G..G..TC. 410
Gga         .AA...A.A.....T...C....C........A..T..C..A.........G.......CC..........T..T.......A..................G.....TC. 440

Hsa         AAACTCTCAAAGTTCTAAAGAAACCTCTGCACTAGAAGCAAGCAGCCCCCTTTTGACTGGACAAATTTTGGAGAGAATTGCCACAGAATTTAATCAGTTACAGTTTCATG 550
Ptr         .............................................................................................................. 550
Ppy         .............................................................................................................. 550
Mac         ........................A................................C.................................................... 550
Cja         ........................A.......................A..............G.................T...........C........A....... 550
Mmu         .....................TGTA...T...A...............A..CC....C.....GG......................G.....C...C.T.......... 550
Rno         G.....C...........G..TG.G..CT...A...............A..C...........GG......................G.....C...C.T........C. 550
Ocu         ...........A............A.......................A.....A..A.....G.............................C...........C.... 550
Eca         ...T....................G.......................A................................T...........C................ 550
Cfa         ...T.......A............A...A................T..A....G.........G..............................C................ 484
Bta         ...T......CA............A.......................G..............G.............................................. 550
Mdo         ...T..C....A......G.....A...T..T................A..G........G..G.....A.......................C........A....... 550
Tgu         .C.T..C...G.CA........TTG..CT..T....G.GG.A...T..A...C.A.........G....A..........................AC....A....... 520
Gga         .C.T..C...G.CA....C..GTTA...A.TT......G..A......G.....A........GG....A..........................AC....A....... 550

Hsa         CTGTTCAAAGCAAAGGCATGCCTCTTTTGGACAAAGTAAGACCGCGTATAGCTGGCATTACAGCCATGTTACAGCAGTCACTGGAAGGTCTCCTATTAGAAGGCCTTCAG 660
Ptr         .............................................................................................................. 660
Ppy         ........................................G..................................................................... 660
Mac         ........................................................................................C..................... 660
Cja         .C............................................C............................................................... 660
Mmu         ....G..G.....G........C..............G.....T..............C...........G........T........C.....GC.G.....TT.G... 660
Rno         ....G........G........C..............G.....C..C........G..C.........C.G.....A..T........C......C....G...T.G... 660
Ocu         ...................................A.......A...........T...................................G..G............... 660
Eca         ...........................................A.................G....................C.....C.....G........T...... 660
Cfa         ..........................C...............................C..G.................T........C..G..G........T....G. 594
Bta         .................T.........................A...........T................................C..............T...... 660
Mdo         ................A..............T.....G.....A..A........T..A...........G..A........A.....A..G...........T...... 660
Tgu         .A..A...........A.....C.....A........G.....A...........A..A.........C.G........T........G..G..C..G............ 630
Gga         ....A...........A.....C...C..........G.....A...........A..A.....T.....G........T........A..G..C..G.....A...... 660

Hsa         ACGTCTGACGTCGATATAATACGGCACTGCTTGCGGACTTACGCCACGATTGACAAGACACGGGACGCGGAGGCCTTAGTTGGCCAAGTACTAGTGAAACCATACATAGA 770
Ptr         .............................................................................G................................ 770
Ppy         ........T................................T.......................T............................................ 770
Mac         ........T.....C............................................................................................... 770
Cja         ..T..............C..............A........T.................T.....T...........G................................ 770
Mmu         ..C..A..T..G..C..CG.C.........C....C..C..T.....C.....T.......A...T..A..A..TC.G..G........C..G.....G.....TG.CA. 770
Rno         ..C..AA.T..G..C..C..C..C..T...C.......C........C....................A..A..TC.G..C........C..G.....G.....TG.CAG 770
Ocu         ..T..CA....G..C...G.G..........................C..C........C.....T..........................G................. 770
Eca         ..T.....T..T...........A........A........T..............A........T..A..A..G........T.....................G.G.. 770
Cfa         ..T..CA.T..T........T...........A.....C..T.....A.................T..A.....A..........................G.....G.. 704
Bta         ..T..CAGT..T.....T..T...........A..C...........A...........C.....T..A.....G........T.....C..C............G.... 770
```

```
Mdo      ..T...A.T..T..C........T..T..............T..T..A............A.A..T..A.....A................................T.. 770
Tgu      ..A..AA.T..T..C........C.....TC.A..C.....T..A..A.....T..A.....A..T..A.....A........T.....G..T..A.....T..TG.... 740
Gga      ..T..AA.T..T..C........T.....TC.T..C.....T..T..A........A.....A..T..A.....A........T.....G..T..A.....T........ 770

Hsa      CGAGGTGATTATAGAGCAGTTTGTTGAATCTCATCCCAATGGCCTTCAGGTCATGTATAATAAACTCCTGGAGTTTGTTCCTCACCATTGCCGCCTTCTTCGAGAAGTCA 880
Ptr      .................................C............................................................................ 880
Ppy      .............................................................................................................. 880
Mac      .............................................................................................................. 880
Cja      ..................................................................T...........G.............................T. 880
Mmu      T.....A..CG.C........C.....G.....C....G.A........C.......C..C..G..T.....A.....C........C...........C..G....... 880
Rno      T.........G.T....G.G.C.....T..........G......G...C.......C..C...........A..C..C........C...........C..G....... 880
Ocu      T.........G.G.....C........G..........G..........A.G......G................C...........C...................... 880
Eca      T..............A...A.......G........A............A.........................................T.............G..T. 880
Cfa      T........CG........A................A............A.............G.......................C...................... 814
Bta      .........CC........A.......TAG.....TG.G..AT..C...C........G........T.......C.....C..T..C.................G.... 880
Mdo      A.........G....A..A......C.G.....C..A..A.........AC............GT.GT.........................T................ 880
Tgu      T........GG.G.......A...GC.......C..T........C....CT.....C....GG..GT................T........T..C..G..G.....T. 850
Gga      T.........G.G.......A....C..G.......T.............C......C........A....................C.....T..CT.G..T.....T. 880

Hsa      CAGGAGGTGCCATCTCCAGTGAAAAAGGCAATACTGTTCCTGGATATGACTTTTTGGTGAATTCTGTTTGGCCACAAATAGTACAAGGATTAGAAGAAAAGTTACCCTCG 990
Ptr      ...................................................................C.......................................... 990
Ppy      ...................................................................C.......................................... 990
Mac      .G.................................................................C................................A......... 990
Cja      ....C..A...............................................A...........C...........C.......................G.....A 990
Mmu      .......A..TG.G..A.....G.......C..TC........C.....T..C..............G......G.......T.G..CC.....G......C........ 990
Rno      .......A..TG.G..A.....G.......C..TC........C.....T..C..............G.....GG.......TGG....C....G......C.......A 990
Ocu      .G.....A........T................T.........G.....................C.C......G............................G.....A 990
Eca      .......A........A................................T.................C......G.........G....C..........A........A 990
Cfa      .......A........A..........................G.....T...........C.....C......G.........G..................G..T..T 924
Bta      .......A........A........G..............C..C.....T.................C......G.........G...............A.....G... 990
Mdo      .......A........A...........T....................T...C.T...........A......G........TGT..T........G...C.C..T..A 990
Tgu      ....G..A..A..T.............CAG...T...............T..C..A........G..G......G.........GT..T..G.....G..A.....A..A 960
Gga      ....G..A..T..T..A..........CAG...T...............T..C..A.....C..A..G......G.........GT..T...........A..G..A..A 990

Hsa      CTTTTTAATCCTGGGAATCCCGATGCATTTCATGAGAAATATACCATAAGTATGGATTTTGTCAGAAGATTGGAACGGCAGTGTGGATCACAGGCTAGTGTAAAGAGATT 1100
Ptr      .............................................................................................................. 1100
Ppy      .......................................................................T........A.......................A..... 1100
Mac      .......................................................................T................................A..... 1100
Cja      .......................C...............................................T........A..............C........A..... 1100
Mmu      ..C..C..C......G.C..A..C..C.....CC.......C..TG.G..C.....C.....GCAG..G..T..G..............C.....C..C......C..C. 1100
Rno      .....C..C......G.C..T..C..C.....CC.......C..GG.G..C.....C..C..G..G..G..T..G..............C.....C..C......C..C. 1100
Ocu      ....................T...........C...........................T.A........T...GAA..A..............C........A..... 1100
Eca      ....................T....................C...G................A....C...T........A..............C........A..... 1100
Cfa      ....................T....................C....................A...GC...T.......................C.....T.GA..... 1034
Bta      ..G............G....T...........CC............................A....C...T........A..............C........A..... 1100
Mdo      ..A.................A...T....C........G.....T.....C...........G........T.....A..A..C...........C.....G..A..... 1100
Tgu      ........C.....A..C..A....TT..............C....CC..............AC.G.A...T..............C..C.....C.....G...C.G.. 1070
Gga      ..G.....C.....A..C..A....TG...........G..C..T.CC..............AC.G.AG..T..............C........C.....G.GAC.G.. 1100

Hsa      AAGAGCCCATCCTGCCTATCACAGCTTCAATAAGAAGTGGAACTTGCCTGTTTATTTTCAAATAAGATTTAGAGAAATAGCGGGATCCTTAGAAGCAGCACTTACAGATG 1210
Ptr      .............................................................................................................. 1210
Ppy      ......A..............T...........T............................................................................ 1210
Mac      ......A..............T...........T...............................................A...............A............ 1210
Cja      ......A..............T...........T...............................................T............................ 1210
Mmu      TC....A..C.............A.....GC..T.........C..........C.....G.....G..CC.....G.G..A......C.G........C..C.....C. 1210
Rno      GC................C..........GC..C.................C..C..C..G.....G..C......G.G..A...............T.C..C....... 1210
Ocu      ......A....................T.....T............................................................................ 1210
```

```
Eca        ......G..........................T.............A................................................TT............ 1210
Cfa        ......A..........................T...........A....................G...........C..A............................ 1144
Bta        ......A..........................T....................................................................C.....CA 1210
Mdo        ....T.A...........C.....T........C...........A...................................A......................T..... 1210
Tgu        ......T.....CT.T..C........T..C..C..A.....T........A.................C...........A..GG.........A.......T.....A 1180
Gga        G...T.T.....CT....C........T..C..C..A............................................A..GG..........T......T.....T 1210

Hsa        TCCTGGAAGATGCCCCAGCTGAAAGTCCGTATTGCCTTTTGGCTTCTCATAGAACTTGGAGCAGCCTTAGGAGGTGTTGGTCAGATGAGATGTTCTTGCCATTACTGGTG 1320
Ptr        ............................A................................................................................. 1320
Ppy        ............................A................................................................................. 1320
Mac        ............................A...............................A......C...................................G...... 1320
Cja        ...................C........A....................C.....A...............................................GT..... 1320
Mmu        GG..C..G..C..........G...........................C.....A....T......GG.C.AA..C.....C............C.A..T..GT...CA 1320
Rno        GG..A.....C..........G................C..........C.....A....T...T..G..C.AA..C.....C............C....T..GT...CA 1320
Ocu        .T........C..........G...........................C.....A........T.......A................C....T........G...... 1320
Eca        .A...........T.......G....T............................A............CA...................C.....C.A..G..G.....A 1320
Cfa        .A.....G.....T.....C.GC...T...T..................CC.G..A...........GCA..A............................GCG....C. 1254
Bta        ..........C..A.......GG..C......C...................G..G............CA......C.....T..CA.............GC.G....C. 1320
Mdo        GG........A..T.......GG..CT.A.T............C.......TGG.A...GT...T....T..A.............C.......T..A.....G..A.CA 1320
Tgu        GA..A..G..A..A.......GG..CT.C.TC..T..C.....CA....C.TGGTC........T..CGT..A...C.........C.......T..A..GC.G..A.C. 1290
Gga        CT..A..G..A..A....AG.GC..CT.A.....T.........A....C.TGGTC....TG..T...TT..A...C.....G...C.A........A.....GT.A.CA 1320

Hsa        CATCGCCTGTGGAGACTCACTCTGCAGATTTTGGCACGATACTCTGTGTTTGTCAATGAGCTTTCACTCAGGCCCATTTCTAATGAAAGTCCCAAGGAGATCAAGAAACC 1430
Ptr        .............................................................................................................. 1430
Ppy        .......................................................G...................................................... 1430
Mac        ...............T.......................................G...................................................... 1430
Cja        ..............................C.......................................A....................T...A........A..... 1430
Mmu        ..C..T........G.....G.........C....CA...T..............G.........TG........G..........G...G.........CT..A..G.. 1430
Rno        .....T........G.....G.........C....C....T..............G.........TG..........C..........C.G.........CT..A..G.. 1430
Ocu        .....T.............................C.................T.G.....................A..........C.G.........G...A..... 1430
Eca        ....AT..............A...........A..C..G.....CA..................T............C............G.......T.CT..A..G.. 1430
Cfa        .....T..C.....G.....G..............T..G.....CA.........G........T............C............A.......T..T..A..G.. 1364
Bta        .....T..........G..G.........C....G..G.....CA.A........G.......T............C............G.T.....TG.T..A..G.. 1430
Mdo        ..C..T..C....A.......T.A........A.....G.....CA.....A.T..C...T.A.T.........TC.C..C.......T.G.......T.A...A...T. 1430
Tgu        ..C..........AG....GC......G.C........C...........CA...GC...G....TG.A..A........C.G...G.A.A.A.....A.G...A...A. 1400
Gga        ..C..T.......A...T.G......A......T....T...........CA........G....CG.A...........C.G...T.A.A.A..A..A.G...A...TT 1430

Hsa        TTTGGTAGGTAGCAAAGAACCTACTGAAGACCAAGGAAGTGGTCCTTCGGAAACAAAGGTGGTTTCCATTTCCCGCACTCAGCTCGTGTATGTGGTTGCAGACCTGGACA 1540
Ptr        .............................................................................................................. 1540
Ppy        ...........................................T...........................................A...................... 1540
Mac        ................................................A....................................C........................ 1540
Cja        ............................................AG..A.........A......G.......................G.................... 1540
Mmu        ....ACT..C........C...T.G..............CCA.G....A...G...GC.CC.CG........TA..........G...........CT.........G.. 1540
Rno        .....C...C........C...T.G..C.....T.....CCA......A...G..GGC.CT..GC....C...A..........G...........CT.........G.. 1540
Ocu        ....A.........G...C....G........C......CA..G.GG.A..C.....................A...........A.C..C.....C.........C... 1540
Eca        .......A...A......C....G........................T...........A............A.T.T...A..T..A...................... 1540
Cfa        ..................C...GG..................C....................C.........A.T..G..A..T...........A............. 1474
Bta        .........C........T.T.TGC..........C......C.....T.........A.C..C...G.....A.......A..T..C........CA........C... 1540
Mdo        C.CCA..AC..A...G..GT...A...G..........A...C.T...T......C....A.C..T.......AAT..A.....TA.A..C..............T.... 1540
Tgu        .G..CC......A..G...T...G............G.A..AAT....T....GCCT.T..TC...T..A..TA.T........GA.A.....T.C...T..T....... 1510
Gga        AA..CC......A..G...T...A...G........G.A...AT....T....GTC..T.ACC......A...A.T......T.G........A.C......T.....T. 1540

Hsa        AGCTTCAGGAGCAGCTTCCAGAACTCTTGGAAATAATCAAGCCAAAACTTGAAATGATTGGCTTTAAGAATTTTTCTTCTATCTCAGCAGCCCTGGAGGACTCCCAGAGC 1650
Ptr        .............................................................................................................. 1650
Ppy        .................................C............................................................................ 1650
Mac        ...................................................................................A.......................... 1650
Cja        ...............................................................................C.............................. 1650
```

```
Mmu    G...G.....ATG.........C...C....G.CGG......AG.....C..........................G...........G........A...........T 1650
Rno    G...G.....ATG.........C...C....G.C.G...G..TG........G...G.C..............C..A...........G........A...........T 1650
Ocu    C...G.....................C..................................A....GC....................G.....T............... 1650
Eca    .................................C.G...........................................C...........................CTG 1650
Cfa    .................................C.....................................................................T....CG 1584
Bta    G...........................A....C...................G....G.................C..C...........................CTG 1650
Mdo    .A.....A..A..A.............................C.........G.A.....T..C...........C..........A...T..T.....T..T.GA.TT 1650
Tgu    .A..G.....T.G.A....T..CA....A..C..G..T..A.....G.................C......A.A....GC..TG...G.........A..T..AA...CT 1620
Gga    .A..C.....AA..A....T..CA....A.....G..T..A...........G...........C........G....G....GA..G....T....A.....AA...CT 1650

Hsa    TCTTTTTCAGCCTGTGTGCCCTCCTTGAGTAGCAAGATCATCCAGGATTTAAGTGACTCTTGCTTCGGTTTCCTAAAAAGCGCCCTGGAGGTTCCCAGGCTTTACCGAAG 1760
Ptr    .....G........................................................................................................ 1760
Ppy    .....G............................................................A........................................... 1760
Mac    .....G............................................................A................G.......................... 1760
Cja    ...C.G............................................................A........................................... 1760
Mmu    G.C..G..T...CA......GG.TC....C....G...TG.......CC.G.....G.........A...AT..C..G..T...........C..............C.. 1760
Rno    G.C..G..T...CA......AG.TC.........G...TG.G.....CC.G..C..G.............AT..G..G..T...........G..............C.. 1760
Ocu    ..C..A.........A.A..TC..C....C.................CC.......G..C......A.G.A...G.....T..............T.....G........ 1760
Eca    ...C.G.........C....T........C.A..G............C..G.....G........GA...A...G.....T..............TC............. 1760
Cfa    ...C.G..T........T..TG....A....A..G.........A..C........G.....T...A...A...G.....T............................. 1694
Bta    .....G..T.....C..A..TG........GAG..A....C......C........G........TA..CA...G.....T....................G........ 1760
Mdo    .....A..CT...CCA....T...C......A..G.........A..C..G.....G..C......A...AT..G.....T..T.....A.....T..A........... 1760
Tgu    ...C.A..............TA......A..A..G...T.........C.C.....G..C.C...T.CA.A...G..G..T..G.....A.....A..AT.A..TA.G.. 1730
Gga    .....A........CA.A..TA......A..A..G...T.........C.C.....G..C.CT...AC..A...G..G..T..A.....AA....A..AT.A..TA.G.. 1760

Hsa    AACCAATAAGGAGGTCCCAACCACAGCTTCCTCCTATGTGGACAGTGCTCTGAAGCCCTTATTCCAGCTTCAGAGCGGACACAAGGATAAGCTCAAACAAGCAATAATTC 1870
Ptr    .............................................................................................................. 1870
Ppy    ............................................................................A................................. 1870
Mac    ..........................................................................A................................... 1870
Cja    ..........................................T................................T......................C........... 1870
Mmu    G.................C.G......C..T..A.....A........A........AC.G.A............T.....TGG...C...G.GC.G.C....G.G..G. 1870
Rno    G.................C.G......C..T..A..............C........AC.G.A............T.....TGG...C...G.GC.G.C....G.G..G. 1870
Ocu    ......C..............TG.........................CT.......A..CCA..........C.T....................G..T...G...... 1870
Eca    ...............G.....T...........T...................G...A..T.A............T..G.........................G..... 1870
Cfa    G..............G.....A.....C.....T.......................A..T.A...A........T..G....................G.......... 1804
Bta    ...............G..CGTG.....C.....T..C........C...........A..G.A.............A.G........C........G.....C.CG.... 1870
Mdo    ..............C........A......AC.T..C...........A........A..C.A......C.....T.AG........C..A.....G..GT......... 1870
Tgu    ......C........G.....T.A......AC.T.....T...........T........C.AT.GA..G....AT.ACT...GA...TCCT.G..G..GC.C..G.... 1840
Gga    ............A..G.....T.A......A..T...........C.....T........C.A..GA..G....AT.AGT...GAA...TA..G.....GC.C..G..C. 1870

Hsa    AGCAGTGGCTAGAAGGCACTCTCAGTGAAAGCACTCATAAGTACTATGAAACCGTGTCAGATGTATTAAACTCTGTGAAGAAGATGGAAGAGAGCCTGAAAAGGCTGAAA 1980
Ptr    .............................................................................................................. 1980
Ppy    .................G.................................................G.......................................... 1980
Mac    .................G............................C....................G..............A........................... 1980
Cja    ........T........G.C........G..........................A.........C.G..............A........................... 1980
Mmu    ..AGC.....GC.G.AAG....GTCG..C.....A..C.G.....T...G..T.........C..GC.G.....A..............G..A.........C.C..C... 1980
Rno    ...GC......C.G.AAG....GTC...C.....A..C.......T...G..T.........C..GC.G.GT..A....G.........G..A........GC.C..C... 1980
Ocu    ..........G.....AG....GTCA..G.....G........T........T.....T.....CC.G..........G...A........................... 1980
Eca    GT......T.......TG.G...TC..CG.......................T.....G.....CC.G..............A.....G.................A... 1980
Cfa    .................G.A..TTC...G.................C.....T........C..TC.G.G...G....G.........G..................... 1914
Bta    G...A...T......C...C...TC...G.....G..............G..T.....G.....CC.G.........CG.........G...........G.....C... 1980
Mdo    .T..A...T.......TG....TTCA.....T.....C.....T........T..A..T.....TC.G.G.........................T.......A..A... 1980
Tgu    .T......T.G.....TG.....TC.........A..G..AGCAAGA..TTT.C..G.TA.GAA....CTT..A.CA.CC------------------------------ 1920
Gga    .T......T.G......G.....TCC........A..A.....T........T..A........G..G.GT..C..T.....A............T.A...........G 1980

Hsa    CAAGCCAGAAAAACCACTCCCGCCAACCCCGTCGGTCCCAGTGGTGGCATGAGCGACGACGACAAAATCAGGCTGCAGTTGGCCCTAGATGTTGAGTACTTGGGAGAGCA 2090
Ptr    .............................................................................................................. 2090
```

```
Ppy   .....................................................T..........................................A.............  2090
Mac   .....................A................A---...........T..T.............................G..............A........  2087
Cja   ...........G....................T...........C...........T..T..................C.......G........A.....A........  2090
Mmu   ........G.G.T..C.AG..A.......T...A.CT....C.....T.....T..T........G...C.A......C.......G..C..G...C..C.......A..  2090
Rno   ........G.G.T..C.GG..A.......T...A.CT.......C---.....T..T........G...C.A......C....T..G..C..G...C..C.......A..  2087
Ocu   .....................A...C...T...........C..............T........G............C.....T.G.....G......C..........  2090
Eca   ....................T........T..T...T.....A.C........T.....T..........................G........A.....A........  2090
Cfa   .............G.......A....T...........T..............T..T..T........................T.......G.................  2024
Bta   ........G.G.........T....G...G.CT..G.T......C...........T..T.....G...C.A.G....C.....T.G..C....C...T..A........  2090
Mdo   ..G...........TG..A.AA....T...A.T..CA.T.A...C...........T...A..........A..C..AC.....T.G..........C...A.....A..  2090
Tgu   ------------------------------------------------------------------------------------------------------------TC  1922
Gga   ..G.......GG..TG.AG.TTTG........T...A.A.A...G..T.....T..T..T..T......C.A......C....T.....C..C.....T..T........  2090

Hsa   GATACAAAAGTTGGGACTACAAGCAAGTGACATAAAAAGCTTCTCAGCTCTCGCAGAGCTTGTTGCTGCTGCCAAGGACCAGGCAACAGCAGAGCAGCCT  2190
Ptr   ..........................................TC......................C....................CA...........  2190
Ppy   ...........................................C........................................................  2190
Mac   ..........C.......G........................C........................................................  2187
Cja   ..................G...A....C...............C........A...G...............................A.G.........  2190
Mmu   ........G.A....CT.G..GA.C..C.....C..G......C....C...ATG.....C..CCT........G.........GG.T.....A.....C  2190
Rno   ...C......A....CT.G..GA.C........C..G......C.G..C...A.G........CCT...C....G..........G.T...........C  2187
Ocu   ..........A....C..G...A....CAG.............C.......GT.C..........T...................G.............G  2190
Eca   ..........A....C..G...A...AG..............TGT...A...A............TC..........................A......  2190
Cfa   ..........A....C..GGC.A...AG........G.....TC.G..C..G........G.....C..C.................T.....A------  2118
Bta   ....G.....A....C..G.G.A...AG.......G......TC.......T........C........C.................G..G.........  2190
Mdo   A..G.....AA...AT..G...A....CA.............TA........A...........ATA...................TG.....A.....A  2190
Tgu   T....G....A.......GG..A....CAG............T.....C..T........G...CTAA..........T.....T.....G..A.CAT.C  2022
Gga   A....G....A.......GG..A...ACA.............T.....C..TA...........CTGA..........T.....T........A..AT.C  2190
```

COG3

```
Hsa   ATGGCGGAGGCGGCGCTGTTGCTGCTGCCTGAGGCGGCGGCGGAGCGGGACGCTAGGGAAAAGCTGGCTCTCTGGGATCGGAGACCGGACACGACGGCGCCGCTGACCGA  110
Ptr   ..............................................................................................................  110
Ppy   --------------------------------------------------------------------------------------------------------------  1
Mac   ..................C..........C................................................................................  110
Cja   ..................C..........C.......T................C.............................G.........................  110
Mmu   ..................C......C...C...........C...........CC.C..G......T...........G.......C...T...T............A..  110
Rno   ............T.....C..........C...........C...........CC.C..G......T...................C...T...TA...........A..  110
Ocu   ..................C..........A...A..........C........CC....G..........................A...T...................  110
Eca   ..........G.......C........T.C....................T...C....G..................................A..............C  110
Cfa   .........................C..AG.............C..........C....G.G.......T................C.......................  110
Bta   ..............T...C.....TCT.TGCCA.A...A....C..........C....G...........G......G.....G.........................  110
Mdo   ---------------------------------------------......A..C.C..G.....C.....T.....C.....G..CA..C.C.....T..A..T..T..  65
Tgu   ---------------------AA..C...A------------C.CAC.....TGC....CCGC..C...........C..CC.....C.GC.C..C........C.G...  77
Gga   ---------------A..GA.GA..C...ACC...CCTC.A.CC.A....GT.GC....CCGC...AAC........C..CC.C...C..C.CG.T..........G...  95

Hsa   CAGGCAGACGGACTCGGTATTGGAGCTGAAGGCGGCGGCAGAGAACTTGCCGGTGCCAGCTGAGCTTCCAATTGAAGACTTGTGCAGTTTAACATCCCAGTCACTGCCCA  220
Ptr   ..............................................C...............................................................  220
Ppy   ----------------------------------------------------------------..............................................  46
Mac   ..................G...........................C...........T.C.................................................  220
Cja   .C................CC..........................C.............C.....C..G.......................G................  220
Mmu   ..................GC........C.......C.TG......C..........C..C.....C...........TG.A.....C...G..........G.......  220
Rno   ..................GC........A.......A.TG......C..........G..C.....C...........TG.A.........G..........G.......  220
Ocu   .........C........GC...................G......C..........G.AC.....C..........................C........G.....TG  220
Eca   ..................G....................G......C..........G.TC.....C...G...............................G...T...  220
```

```
Cfa   ..................GC...................G......C..........G..C.................T.....T......G..........G.G..... 220
Bta   .C................GC...C............T..C...GC.C....C.....G..C.....C..T........T..A.........C.C................ 220
Mdo   .C.C..A...........GC...................G......C.A........G..C.....C..T........T..............T....GA...T..T... 175
Tgu   GC.C....AA........GC..........T....AAA.C......C.CGTTTGT..T.....C..G..C.....G...........CC.G..G........G.....TG 187
Gga   .C.C.....C........GC...................C..A...C..........GC.C........C........T..A.....CC.............GT..A.TG 205

Hsa   TTGAACTGACTTCAGTAGTGCCTGAATCTACAGAAGACATTCTCTTGAAGGGCTTCACTTCCTTAGGAATGGAAGAAGAAAGAATTGAAACCGCACAGCAGTTTTTCTCA 330
Ptr   .............................................................................................................. 330
Ppy   ......................G....................................................................................... 156
Mac   .............................................................................................................. 330
Cja   ................C...........C.............................C.............................G..................... 330
Mmu   ....G......G.T...........C..............C..G..A...................................G.....G..T..G............... 330
Rno   ....G......G.T........................G.C.....A...........A..T..........................G..T.................. 330
Ocu   ..C...........A.....................................T......................................................... 330
Eca   .G.C..A..A.G.......A....C...........................T.............A..............................A............ 330
Cfa   ...C..A.......A....A................................G.............A........................T.................C 330
Bta   ...C..A.........G.....C.............................A.....G.......A..................C.....T.................. 285
Mdo   .CTCTT.A.....CACG.....A...........G..T.....T........A..TG.C..T.....C.......................T...........C...... 285
Tgu   ..CTG.....AG.T.CC.....A..G..C.....G..TG.C..TC.......A..TG.AA.AC....G.....G..T..............A...........C.....C 297
Gga   ...C......AG.T.C......A.....C........TG.....C.......A..TG.CATGC.G........GA.T..............A..G........C.....C 315

Hsa   TGGTTTGCAAAGCTGCAAACTCAGATGGATCAAGATGAAGGAACTAAATATAGACAGATGAGGGATTACTTGTCTGGGTTTCAGGAGCAGTGTGATGCTATATTGAATGA 440
Ptr   .............................................................................................................. 440
Ppy   ........................................A..................................................................... 266
Mac   ......................................................................................................C....... 440
Cja   .................G..........................C.........................................................C....... 440
Mmu   ...........A....................G.....................................................A...............C....... 440
Rno   .......................A........G.....................................................A...............C...G... 440
Ocu   .......................A.................G........C...............................................C........... 440
Eca   .....C........A.................G.......A...G..G.........................................A.................... 440
Cfa   ..............A.................G.......A.........C........................................................... 440
Bta   ..............A.................G..C.......................................................................... 440
Mdo   .........C....A.....C........G..............G.................A.......................A.....C.....C...C....... 395
Tgu   ........TC.AG.......A....................TG.C..G.............................C.......................C........ 407
Gga   ........TC.......G..A....................TG.C..G.....G....................C..C..............C................. 425

Hsa   TGTAAACAGTGCTCTTCAGCATCTGGAGTCTTTGCAGAAACAGTATCTTTTTGTGTCCAATAAGACAGGAACCCTACATGAAGCCTGTGAACAGCTCCTAAAAGAACAGT 550
Ptr   .............................................................................................................. 550
Ppy   ..........................................................................................G................... 376
Mac   .........................................................................................................G.... 550
Cja   ......................................G............................................................T.......... 550
Mmu   C..G...........G.......A......G.........................................T..T.................................. 550
Rno   C..G.................C........CC................G...........C...........A..T..C..........................G.... 550
Ocu   ........A.....................................................................C............................... 550
Eca   ...........................A..A...............................................C............................... 550
Cfa   ........A..................A..A............................................................................... 550
Bta   ...........................A..A...................................................................G.....G.... 550
Mdo   ...T.....A..............C..A...............................CA........T..T..G.......................G.....G.... 505
Tgu   ...G..T.....C........C........AC....A......................CA...........G..G.....G..............TT.G.....G.... 517
Gga   ...C..T.....C..............A..AC....A......................CG........G..G..G.....G..............T..G.....G.... 535

Hsa   CGGAACTTGTTGATCTGGCTGAAAACATTCAACAAAAGCTTTCCTATTTTAACGAATTGGAAACTATTAACACAAAATTGAATTCCCCTACATTGTCGGTGAATAGTGAC 660
Ptr   .............................................................................................................. 660
Ppy   .................................................................................................A............ 486
Mac   .............................................................................................................. 660
Cja   .........................................................................................G.G.................T 660
Mmu   ....G....C......T.....GC....C.....G.....G..T........T..G.....G..G..........................G.....T...........A 660
```

```
Rno      .........C......T.....GC....C.....G.....G..T........T..G.....G..C.......................A..G.....T...........A 660
Ocu      ..............T.....................................T.................T..............T..A........T...........A 660
Eca      T......G..........................G.................T.................T..T...........T...........T...........A 660
Cfa      ....................................................T................................T.....G.....T..T........A 660
Bta      .....................................A..............T................................T..C..G.....T...........A 660
Mdo      .A.....G.....C...........T..............C..T........T.................T..G..............A..G.....T.....C.....A 615
Tgu      .A...........CT.............C..G..G..A.....T..C.....T..GC......A...C.....T...C.............GC....C..A..C.....A 627
Gga      .A...........CT.............C..G..G.....G..T.....C..T..GC....G.AC..C.........C....C........G.....T.....C.....A 645

Hsa      GGATTTATACCTATGCTGGCCAAGTTAGATGATTGTATAACATATATCTCATCTCATCCTAATTTTAAAGATTATCCCATATATTTGCTGAAGTTTAAACAGTGTCTTTC 770
Ptr      .................A............................................................................................ 770
Ppy      ..............................................................................G............................... 596
Mac      ........................C.....................................................G............................... 770
Cja      ..................T.......G.....C............................................TG.C...........A................. 770
Mmu      ........C......T...............................T.....G.......................TG....C..A..............A........ 770
Rno      ........C......................................T.............................TG....C..A..............A........ 770
Ocu      .......................................G..........C..........................TG......................A......G. 770
Eca      ................................C..........................G.................TG.......A...........G..A........ 770
Cfa      .....................................................A.....A.................TG...................G..A........ 770
Bta      .........................................G..C..A...........G.................TG.......A...........G........... 770
Mdo      ...........A........T.......................C..T..T..............C.....C.....TG.T.....A..C........G..A..C..... 725
Tgu      .....C..T..C........T...C.T...........TG.......T.....A.....A........G..C.....TG........ACA...........A..C..... 737
Gga      .....C..T..C........T...C.G...........TG.G.....T.....A.....A...........C..C..TG........ACT..A........A..C...CT 755

Hsa      TAAAGCTTTGCACCTCATGAAGACATATACTGTGAACACACTACAGACCCTCACAAGTCAGTTACTGAAAAGGGATCCTTCATCTGTACCTAATGCAGACAATGCCTTCA 880
Ptr      .............................................................................................................. 880
Ppy      ........................................................................A..................................... 706
Mac      ........................................................A..................................................... 880
Cja      ......C....................C............................A..................................................... 880
Mmu      ..........................................G.....T.......A.........A........C..C.....A..C...................... 880
Rno      ..........................................G.....T.......A.........A........C..C.....A..C...................... 880
Ocu      ........................G...............T.G...........C.A....C...................C............................ 880
Eca      ............T...........G..C...................A................T............................................. 880
Cfa      ............T..T........C..C...A.A...........A.A........A.......T.A............G.....A......................T. 880
Bta      ...........................C...................A........A.......T.A.........................................T. 880
Mdo      .......A.......T.....A..T..C..............G..A.A............A...A.C...C................G..A...T............... 835
Tgu      .......A.......T..C........C........T..........A........CA......A.................G....G..A...T.T............. 847
Gga      G......A.......T..C........C...........G..G..A.A.........C......A.................G.A..G..A...T.T............. 865

Hsa      CATTATTTTATGTGAAATTTCGAGCTGCTGCCCCCAAAGTCAGAACTCTTATTGAACAAATAGAACTGCGGTCTGAAAAAATACCTGAATACCAACAACTGCTAAATGAT 990
Ptr      .............................................................................................................. 990
Ppy      .............................................................................................................. 816
Mac      .............................................................................................................. 990
Cja      ............................C...................................G.....A.......................G........C.G.... 990
Mmu      ..C......................C........T..............A........G.....G.AA..A........G.....C........G..C..T..C.....C 990
Rno      ..C...............................T..............C..............G.AA..T.....G..G.....C........G..C.....C...... 990
Ocu      ..C...............................T.............................G.AA..A........C.................G.......G.... 990
Eca      ..C...............................................................AA..A......................................C 990
Cfa      ...............................T..................................A...A......................................C 990
Bta      ..C..............................................C........G.......AA..A..................................C...C 990
Mdo      .TC..........A.................T.................C.........G....G.AA..A..............A...........G..TT.......A 945
Tgu      ..C.G..............CA....A.....T...........................G....G.AAA.A...........G..A..G..T..G...G.T..C.....A 957
Gga      ....G........A.....CA....A.....T.................G.....G...G....G.AAA.A........G..G..A..G..T......G.T..C.....A 975

Hsa      ATCCACCAGTGTTACCTTGATCAGCGGGAGCTCCTTTTGGGCCCTAGTATTGCTTGCACTGTTGCAGAGTTAACCAGCCAAAATAATAGAGATCACTGTGCCTTGGTTCG 1100
Ptr      .............................................................................................................. 1100
Ppy      .............................................................................................................. 926
```

```
Mac   ..A.......................................T.....................................................T.............. 1100
Cja   ....................................C.............A..................C...G............C.....C................. 1100
Mmu   ............................................A........C.A......CA................G..C..C.....C..T.............. 1100
Rno   ............................................A........C.A......CA.G...C..........G..C..C.....C..T.............. 1100
Ocu   ................................G.......................T.....CA.....C.............C..C........T.............. 1100
Eca   .......................A.................T.........A....T.....AA.......G........G.....C..G.....T.........A.... 1100
Cfa   .....T..............C.............................CA....T..C..AA..........A.....G..............T.........A.... 1100
Bta   .................G...........A..............C......A..........AA.......G........G.....C.....C.....C......A.... 1100
Mdo   .....T.....C............A....A..T..GC....T.........A..A.T.....AA.T........A..T..G..C..C........T.............. 1055
Tgu   .....T..A........C..C...........TT.GC.T..T..A.....C...A........A....A........T..G.....C........T.....TC....A.. 1067
Gga   .....T..A...........C...A....A..TT.GC.T..T..A.....CT.CA....C...A.............T..G..C..C.....C..T.....TC....G.. 1085

Hsa   TAGTGGCTGTGCCTTCATGGTTCATGTCTGCCAGGATGAACACCAACTTTACAATGAATTTTTCACAAAACCAACATCAAAATTAGATGAGCTTTTGGAGAAACTGTGTG 1210
Ptr   .................................A............................................................................ 1210
Ppy   ..............................................................................................---............. 1033
Mac   .............................................................................................................. 1210
Cja   .............................................................................................................. 1210
Mmu   C..............T.....C...........A.....G.............................G.......................A.........T....C. 1210
Rno   C..............T.....C...........A.....G.......................T.....G.................C..A..G..............C. 1210
Ocu   C..............T.....C.....A..............T..G.................T.....G....................A................... 1210
Eca   C..............T.....C.....A...........G...................................GC.............A................... 1210
Cfa   C..............T.....C.....A...........G..................................................A..............C.... 1210
Bta   C..............T...........G...........G.....G...........G.................G..............A................... 1210
Mdo   ........................C..G..T..A.........................................G..............A................... 1165
Tgu   A.....T........T.....C.....A.................G...........G..C...............C.....C.G........C........GT.....C 1177
Gga   G.....T..............C..C..T...........G..T..G...........G..C.....G..G......C.....C.G.....A..C........G......C 1195

Hsa   TGTCATTGTATGATGTCTTCAGGCCATTGATCATTCATGTTATTCACTTAGAGACTCTGTCGGAACTTTGTGGGATTCTTAAAAATGAAGTGCTTGAAGATCATGTGCAG 1320
Ptr   ........................................................................................G..................... 1320
Ppy   ........................................................................................G..................... 1143
Mac   ........................................................................................G..................... 1320
Cja   ........................................................................................G.....C............... 1320
Mmu   ....G...........G...C....C........C.....C...............T.A.....G..C.................C..............C..C..A... 1320
Rno   .....C.......C..G...C....G........C..C..C......C.............T..G..C....................G...........C.....A... 1320
Ocu   ...............................................C.............A.................G........G..C...........C..A... 1320
Eca   .........................G..............C.................A..A..........................G.................A... 1320
Cfa   .........................................................................A..............G.................A... 1320
Bta   ....G...............................................A.....C.............................G.................A... 1320
Mdo   ....C.....................C......................G.....C.....T...........A..............G........G........A... 1275
Tgu   .T...............C.G......A.......C.....C........G.....A..T..T..G..C...........C........GA.......G........A... 1287
Gga   .T.....A.........C.GC.....A.......C..C...........G...........T.....C...........C.....C..GA................A... 1305

Hsa   AACAATGCTGAGCAACTGGGGGCATTTGCAGCTGGAGTCAAGCAGATGTTAGAAGATGTACAGGAGCGGCTCGTCTACCGAACCCACATCTATATTCAGACGGACATCAC 1430
Ptr   .............................................................................................................. 1430
Ppy   .............................................................................................................. 1253
Mac   ........C..............................................................T...................................... 1430
Cja   ..............G........G.....C..................C.G.....C..T...........T....................C................. 1430
Mmu   C....C.....A..........................A...........G...........A...A....T..G..T........T.....C................. 1430
Rno   .....C.....A..G........C..............A...........G...........A...A....T..G..T..C.....T.....C................. 1430
Ocu   ...........A..G.....A...........C.....A...........G...................................T.....C........A........ 1430
Eca   ........C..A...........C..............G...........G...........A.............................C........A........ 1430
Cfa   ...........A........A.................A..A.................C..A.....C.........A....T..T.....C........A........ 1430
Bta   ..T........A..G....................C..A.........C......................T........G...........C................. 1430
Mdo   ...........A..G..T....................A..A........G...........A..A..A..G.....T..G..T.....T...........A.....TG. 1385
Tgu   ...........A........T..............T..............G...........A.....C..T.....TA....G.....T...........T........ 1397
Gga   ...........A.....T..T........T.....T..........................A..A..T..T......A.G..A..T..T..C........T..T....T 1415
```

```
Hsa   GGGCTATAAACCAGCTCCTGGAGATCTGGCATATCCCGATAAGTTAGTCATGATGGAGCAAATTGCACAGAGTTTGAAAGATGAACAGAAGAAGGTACCTTCAGAAGCTT 1540
Ptr   .............................................................................................................. 1540
Ppy   ....................................T.....................---------------------------------------------------- 1311
Mac   ................................................A............................................................. 1540
Cja   ............G.................C................C....................................G.................G....... 1540
Mmu   A.....C........G....................T......C...................C...........A..G.....G..........CG............. 1540
Rno   A.......................C.....G..C..T......C.T.............................A..G...............AC.............. 1540
Ocu   T.....C........C........C.....C........G.............................A.....A.................A.....C..T....... 1540
Eca   ......C.......................G.....T...........A..........................A.................................G 1540
Cfa   A..G..C.....T.................T.....T..C.....G..G..................AG......A........G......................... 1540
Bta   ......C....................A..G.....T...........T.................T........A..................T............... 1540
Mdo   T..............C..G.....C.....T.....T......................................A...........A......T....C........C. 1495
Tgu   C.....C..G..C..G..A..C.....C..G.....T..C...C.G.AA.........A......................G............C.G..A..T....... 1507
Gga   A.....C...........G........T........T..C...C.G.AA.......................C..A.....G............T.G..A..T....... 1525

Hsa   CATTTTCAGATGTTCACTTAGAAGAAGGAGAGTCTAACAGTCTGACAAAATCTGGTTCAACAGAATCCCTCAATCCTAGACCACAGACCACAATTTCTCCAGCAGATCTT 1650
Ptr   .............................................................................................................. 1650
Ppy   -----------------------------..................................................................................... 1392
Mac   .......G..............G......................................G........T....................................... 1650
Cja   ................G.............G......T..............C...........G....................C.................G...... 1650
Mmu   .C...........A.GG..............AG.C.GTG..T.A.G...G..............C.......................G..G..C............... 1650
Rno   .C...........A.GG.................C.GT...T.A.G...G..............C..........................G.................. 1650
Ocu   ................G..........C...AG.......C..C.......................T.................A.................G...... 1650
Eca   ...............GG..........C.........T.A...A...............C....C..G.C............T....................T...... 1650
Cfa   ...............GG.....G...ACG........T.A...A..............................................................C... 1650
Bta   .G.............GG..........C....C....T.....A...............T.T.....T.............AG........G............---... 1647
Mdo   ...........A..AGAGGGC.TTCT..G..AGTGCTGG.GA.TGA.GTCCTG.A...G.G....C.T..T........G.................A............ 1605
Tgu   ..........C..C.GGC.......TCCC.....CTG...CT.AGTT...........GG.T........T.....C..G.AC..A.G...G.....A..T........G 1617
Gga   .............C.GGC.......TCCC.....CTG..A.T.AGTT...........GG.G........T.....C....AG...T....G..C..A...........G 1635

Hsa        CATGGAATGTGGTATCCTACGGTTCGAAGAACTCTTGTCTGTCTCTCCAAATTATACAGATGCATAGATAGGGCAGTGTTCCAAGGATTATCACAGGAAGCATTGTCTGC 1760
Ptr   .............................................................................................................. 1760
Ppy   ....................A......................................................................................... 1502
Mac   ..C........................................................................................................... 1760
Cja   .........................................................................................G..C........C........ 1760
Mmu   ..C..G...........C..A........G..A.......................T.................G..........................C........ 1760
Rno   ..C..G...........C..A........G..A.......................T.................G..........................C........ 1760
Ocu   ....................A.............................G..G...............................................G........ 1760
Eca   ....................A...........C.....T..............G..T...........C...........T....................C........ 1760
Cfa   ....................A.................G..............G........T......................................C........ 1760
Bta   ..............C.....A......C........................GT.TT.A..TTCACC.G..................C.............C.....G.. 1757
Mdo   .................C..T.................A...................................................C..............G.. 1715
Tgu   ....................T..CA....G.....A..G............C.G........T.................T..G...........A..G..C..A..... 1727
Gga   ..C..C........C.....T..CA....G..C.....G............C.G........T.....C...........T..G...........A.....C..A..... 1745

Hsa   CTGCATTCAGTCCTTACTTGGAGCGTCAGAGTCTATCAGCAAAAACAAGACTCAGATTGATGGACAACTTTTCTTAATTAAGCACCTTTTGATACTTCGTGAACAAATTG 1870
Ptr   .........................................................................................................G.... 1870
Ppy   ..................G........................................................................................... 1612
Mac   ........................A..................................................................................... 1870
Cja   ..................G..............A....................A....................................................... 1870
Mmu   ......A........G..A.....A........................................................A...........T.....A.......... 1870
Rno   T.....A........G..A.....A....................................................................T.....A.......... 1870
Ocu   ............T...........A........C.....................G..........G....................C.....T.....G.....G..C. 1870
Eca   .........C..G...........A................................C....................A..............T................ 1870
Cfa   ............A..G........A........C...........................................................T................ 1870
Bta   ............AC.G........A........C................................G...........A..............T..............C. 1867
Mdo   T.................A.....T.....T..G....C......T.....C...G..........G.................T........T................ 1825
```

```
Tgu      ......C.....GC.G..G.....TG.T..T..C.................C...G.C........G...........A..A......C....T.....G.......... 1837
Gga      ...TG.C..C..GC.G..C.....TG.T..TG...................C...G..........G...........A..A...........T................ 1855

Hsa      CTCCATTTCACACTGAATTCACCATTAAGGAAATTTCCCTGGACCTCAAGAAAACTAGAGATGCAGCATTTAAAATCCTGAACCCTATGACTGTCCCAAGATTTTTTAGG 1980
Ptr      ...........................................................................................A..T............... 1980
Ppy      ...........................................................................................A..T............... 1722
Mac      ..............................................................................................T............... 1980
Cja      ................................................................G.............................T............... 1980
Mmu      ..............................................................................................T...C........... 1980
Rno      ........................................................C.....................................T..G............ 1980
Ocu      ....G..............................................G.................................C.....G..A............... 1980
Eca      ..............................................................................................T............... 1980
Cfa      ..........T.....................................................T.............................T..G............ 1980
Bta      ..................................................................................T...........T............... 1977
Mdo      .......C......................................T............................................C..T..............A 1935
Tgu      ....T..C........T.............................T...................................T....A.G.A..TT.............A 1947
Gga      ....T..C........T.............................T........................................A.G.A..TT.............A 1965

Hsa      CTGAATAGCAACAATGCCTTGATAGAGTTCTTGTTGGAGGGTACTCCTGAGATAAGAGAACATTATCTTGACTCTAAAAAAGACGTAGACCGTCATCTGAAATCGGCCTG 2090
Ptr      .........................................C.................................................................... 2090
Ppy      .............................................................................................................. 1832
Mac      .....................................................T........................................................ 2090
Cja      .........................................A..C...........................................................A..... 2090
Mmu      .........................................G...........C.......................G..G...........G..C.....G..A..... 2090
Rno      .........................................A...........C.....G.................G..G.....G..T..G..C.....G..A..... 2090
Ocu      .....C.........................................C.................C..........................C........G........ 2090
Eca      ...................................................................................T.......................... 2090
Cfa      ...................................................................................T.......................... 2090
Bta      .................................................................C..C.......................C..C.....G..A..... 2087
Mdo      ....................A.........................................C....................T........A........G..A..T.. 2045
Tgu      T....C..................C.......AC.............A..A..C............A.......C..G.....T.....T..............A..T.. 2057
Gga      ..A..C........C.........C.......AC.............A..A..C........C...A.......G..G..G..T..C..T..C.........G.A..T.. 2075

Hsa      TGAGCAGTTTATTCAGCAGCAGACCAAGCTGTTTGTAGAACAGCTGGAGGAGTTCATGACAAAGGTTTCAGCGTTAAAAACAATGGCCAGTCAGGGAGGCCCCAAGTATA 2200
Ptr      .............................................................................................................. 2200
Ppy      .............................................................................................................. 1942
Mac      .............G.........................C.....................................................A................ 2200
Cja      ....................................G..G....................G..................G.............................. 2200
Mmu      .........C................G............G..........................A.....A..G.....C........C.................C. 2200
Rno      .........C........A.......G...A........G.CA.......................A.....A........C..........................C. 2200
Ocu      .......................................G..........................C.....A.................C................... 2200
Eca      .......................................G................................A..........................A.....A.... 2200
Cfa      ..................A....................G.............................G..A.....G..G..............T..A.......... 2200
Bta      ..........................G......CA....G.C.............C................A..........................A..T..A.... 2197
Mdo      ..................................A....G...........A......T.............A................A.........T.......... 2155
Tgu      .....................A.....A.A.........C..........................AG.T..T................C...A.....T.....T..C. 2167
Gga      .....................A.....A.A....A....G..........................GG.T..T......G.......T.C...A.....T.....A..C. 2185

Hsa      CTCTCTCACAGCAGCCTTGGGCACAACCAGCAAAGGTCAGTGACCTTGCGGCAACTGCATATAAGACAATAAAAACAAAGCTGCCTGTGACATTGAGAAGTATGTCCTTG 2310
Ptr      .......................................A..................G........G.................G........................ 2310
Ppy      .......................................A..................G...........................C....................... 2310
Mac      ..........................................................G.........C......................G.................. 2310
Cja      ....G....................G....................C......G....G.................G..........C...GC.......C......... 2310
Mmu      .C..............G........G.............A......C.T...C.....C..C...........G..G........GC.....C.......C...G.T... 2310
Rno      .C.............................G.......AC.......T...C.....T..C..............G........GCA............C.....TC.. 2310
Ocu      .....................................TG.C.......T...T.....C...............G.....T.....T....G..............A... 2310
Eca      .......T..............................G....G.........GG...C.......A.G.C...G..C.......GC....G..............AC.. 2310
```

```
Cfa          .C..T....................G......................TAA...G..TC..C............G...........C....T..............G... 2310
Bta          .G..G..T........................................T.....G...C..C.....G......G..........CC....................... 2307
Mdo          ....G..T........C.............TC..A....A......A.T.T.TGG...C..C..A.................A..AT....G..............AC.. 2265
Tgu          GC..T.....A..A.....................A...AC..T..G.TCT.TT.CA.T..C..................T....ATCA..T..AC....C........A 2277
Gga          GC..T..T...........................A...AC..TA.G.TCT.TT.CA.T..C......................AGTCA..G..AC....C.....AC.. 2295

Hsa          TACCTATCCAATAAAGATACCGAGTTCATCTTGTTTAAACCTGTGAGGAATAATATTCAGCAAGTCTTCCAGAAGTTCCACGCTCTGTTAAAGGAAGAGTTCAGCCCTGA 2420
Ptr          .................C..............................GTG.G...CAGA-------------------------------------------------- 2370
Ppy          ................................................................................T............................. 2162
Mac          .......G........................................................................T............................. 2420
Cja          ...T.G.......G...C....................G..A...................G....................TC...C.G.................... 2420
Mmu          ..TT.G........G..C..G.................G.....C........C.................................C................TT.... 2420
Rno          ...T.G........G..C..G.................G.....C........C..........................T......C................T..... 2420
Ocu          ........T...................................T.........G....................C....T..........................C.. 2420
Eca          .....G..............T...........A...........C........C.....A........T...........T.T.T......................... 2420
Cfa          .................C..A....................C..T...................................T.T.T................T........ 2420
Bta          ...........C..G.....G.........C..........A..C.....................................TCT................T........ 2417
Mdo          ...T................A..A......C..........G......................CA........AC....T...T........A.....A.....AG... 2375
Tgu          ...T.G..............A..A..A..TC.......G..A..C........C.........A.G........AC....T..................A....TAA... 2387
Gga          ...T.G..............A........T........G..A..T........C..C......A.G........AC....T...T................T..TAA... 2405

Hsa          AGACATCCAGATCATTGCCTGTCCATCTATGGAACAGCTGAGCCTTCTGCTGTCAGTTTCTAAA 2484
Ptr          ---------------------------------------------------------------- 2370
Ppy          .................................................T.............. 2226
Mac          ................................................................ 2484
Cja          G...G...................G..C.....G...........C.................. 2484
Mmu          .........A........T.....T..C.............A...C..............A..G 2484
Rno          ...T........T.....T.....T...G..........A.A...C...........C.....G 2484
Ocu          ......G.........T....C..TA.......G.......A...G......ATG.....C..G 2484
Eca          ...T.C............T.....T................A.T.C..A.....G......... 2484
Cfa          ...TG.............T.....T................A...C.............G.... 2484
Bta          ...TG.T..A........T.....T..A.............A...C..A.....C......... 2481
Mdo          ...T...........A..T..C..T..C.........G...ATT.A........G..G..C... 2439
Tgu          ....C..........A..T.....T..A.........G.A------------------------ 2427
Gga          G..TC.......T..A..T.....T..A.........G.CTC.T.G..TT..G..AA.G.G.C. 2469
```

COG4

```
Hsa          ATGGCGGACCTTGATTCGCCTCCGAAGCTGTCAGGGGTGCAGCAGCCGTCTGAGGGGGTGGGAGGTGGCCGCTGCTCCGAAATCTCCGCTGAGCTCATTCGCTCCCTGAC 110
Ptr          ...........................................C.................................................................. 110
Ppy          .........T.................................C.................................................................. 110
Mac          ...........................T.A.............C.............................................................T.... 110
Cja          ...........C..G....T.......T...............C.............................................A.................... 110
Mmu          ........GG.G..G..T..G.T...AT.......A.CT.CT.C...AC.G..............A..A.A................A.C..............T..... 110
Rno          ........TG.G..A..C.........T.......A.C..CT.CA..AC................A..T.A................A.C..............T..... 110
Ocu          ........T..C..G..T.........T............CT.....C.G.....T.........A...T.................A.C..............T..... 110
Eca          ...........C.....C..A.................T.CT.C...CA....A...........A...........G.........ATC.................... 110
Cfa          ........T..C.....C....A...............TTCT.T...T---......A.......A...........G.........A.C.................A.. 107
Bta          .....A.....C.....C....A............C....CT.G....C....C...........A...A.......A.........A...................... 110
Mdo          .........GCCA.C..C..AATC.....AA.C..A.CCGCTGCAG..G.C..A...AA......C..TT....T..G..GC....T..C........C........... 110
Tgu          ---------------------------------------------------------------------------------------ATG....G...C.AG..G..... 23
Gga          ------------------------------------------AT.G..G.G.GCA.C.AT..C..C.CGATGG...G..GGC.G...ATG....G.G.C...G....C.. 68

Hsa          AGAGCTGCAGGAGCTGGAGGCTGTATACGAACGGCTCTGCGGCGAGGAGAAAGTGGTGGAGAGAGAGCTGGATGCTCTTTTGGAACAGCAAAACACCATTGAAAGTAAGA 220
Ptr          .............................................................................................................. 220
Ppy          ...................A.......................................................................................... 220
```

```
Mac   ...............................................................................................G.............. 220
Cja   .........A..................................................A........A........................................ 220
Mmu   ..................A.....G........A.....T.............C........A...A.......................G................... 220
Rno   ..................A.....G........A...........A................A............................................... 220
Ocu   ........................G...........G....................A..................................G...T............. 220
Eca   C.......................G...................................................................................A. 220
Cfa   C.................................................................A..........................T..............A. 217
Bta   C..T....................G...........................................................G........T..............A. 220
Mdo   C..A.................A..G.....G..T..T...A.T...........T.....AGAG...........C........G.......G...A.....G..C.... 220
Tgu   C..T...GC...C........C.CC...AGC.....G..T.AG...........C...C..GAG.........T.C..CC....G.......G...T..A....G.A..... 133
Gga   C......TCC...........C.CC...AGC.....G....AG........C.CC...C..CAG........C..C..CC....G.....GGG..G...C..G..C..A. 178

Hsa   TGGTCACTCTCCACCGAATGGGTCCTAATCTGCAGCTGATTGAGGGAGATGCAAAGCAGCTGGCTGGAATGATCACCTTTACCTGCAACCTGGCTGAGAATGTGTCCAGC 330
Ptr   .......................--------------------------------------------------------------------------------------- 243
Ppy   ............................C................................................................................. 330
Mac   ......................G.GG.GCT....C--------------------------------------------------------------------------- 255
Cja   .........................C..C........A....................A.......................T........................... 330
Mmu   ..............A.G........C.GC......T.............C........A............................G...A.....A..C.....G... 330
Rno   ..............A.G........C..C......T......................A........G.......................A.....A............ 330
Ocu   .........................C..C......T........................................A..............A..C.....C......... 330
Eca   .........................C..C..A...T.......A...........A.....A.............................A.................. 330
Cfa   ....T....................C..C......T.......A.................A.....G.......................A..C............... 327
Bta   .......C........G........A.....A...T.......A...............................................A..............A... 330
Mdo   ..A....C..G..A..G.....C..C..C..A....................C.............................T....G......A..A.....C...... 330
Tgu   ....TG.C..T..T..C.....C.....C.................G.....CC..........A..G...........C..............C.....C..CAG.... 243
Gga   ....GG.C..G..G..T.....C..C..C........C..C.....G.....CC..........A..............C..A.AT.................CAG.... 288

Hsa   AAAGTTCGTCAGCTTGACCTGGCCAAGAACCGCCTCTATCAGGCCATTCAGAGAGCTGATGACATCTTGGACCTGAAGTTCTGCATGGATGGAGTTCAGACTGCTTTGAG 440
Ptr   ---------------------------................................................................................... 326
Ppy   .............................................................................................................. 440
Mac   -------------------------------------------------------------------------------------------------------------- 255
Cja   .................T............................................................................................ 440
Mmu   .....C..................................................C..................................................... 440
Rno   .....C..................................................A.........................................A........... 440
Ocu   .....C..............A...............................................A......................................... 440
Eca   .....C..C................................A.................................................................... 440
Cfa   .....C............T..........T...........A.................C..T..............A................................ 437
Bta   .....C.......................T...........A....................T....................T.......................... 440
Mdo   .....C.......................T........C..A.....C........................T.......T..T........G........G..CC.A.. 440
Tgu   .....G........C.....T...........A.................................C.T................................A..CC.... 353
Gga   .....C..G.....G..T...........T..A..........................C.....AC.A.....A.......................A..A..CC..C. 398

Hsa   GAGTGAAGATTATGAGCAGGCTGCAGCACATACTCATCGCTACTTGTGCCTGGACAAGTCGGTCATTGAGCTCAGCCGACAGGGCAAAGAGGGGAGCATGATTGATGCCA 550
Ptr   ..A.............................T............................................................................. 436
Ppy   ..A........................C....T...........................A................................................. 550
Mac   -------------------------------------------------------------------------------------------------............. 268
Cja   ..A...............A........C..C.T..................C........A................................................. 550
Mmu   ..A...G....................C..C.T......T....................A....................A........A..A................ 550
Rno   ...........................C..C.T......T....................T.....A........T.....A........A..A................ 550
Ocu   ..A............A...........C..C.T............A..............A...........G.....C.....T.....A................... 550
Eca   ..A........................C..C.T...........................A.............................A..A................ 550
Cfa   ..A........................C..C.T.........T.................A.............................A..C..............T. 547
Bta   A.A........................C..C.T...........................A.....C.......................A..C................ 550
Mdo   ..A......C........A..A..T..T..C.TC..C......C.A.CT..A.....A..A...........T.....G..A........A..A..T............. 550
Tgu   A.A......C........A..A.....T....TC.........C...CT...........A..G...........T..T........G..A...G.A..A........T. 463
Gga   A.A............A..A..A.....T....TC........TC...CT...........A..G...........T..T..A.....G..A...G....A.......... 508
```

```
Hsa  ACCTGAAATTGCTGCAGGAAGCTGAGCAACGTCTCAAAGCCATTGTGGCAGAGAAGTTTGCCATTGCCACCAAGGAAGGTGATCTGCCCCAGGTGGAGCGCTTCTTCAAG 660
Ptr  .............................................................................................................. 546
Ppy  ..............................................................G............................................... 660
Mac  .............................................................................................................. 378
Cja  ..............................................A............................................................... 660
Mmu  ..........A.......................T..G.....A..C..............T..C..T.....A.........T................A......... 660
Rno  ..........A.......................T........A..A..............TGC...T.....A.....G...T................A......... 660
Ocu  ............................G.................A..............T.................G.............................. 660
Eca  ............................G.................AA......................T..A.....G.....A...........A............ 660
Cfa  .............A..............G..............C..AA.............A..C..T..T..A.....G.................A............ 657
Bta  ........C...................G.....T...........CA..............G.......A........G.............................. 660
Mdo  .T.....G.....T..............G.A......G..TG.G...A....A.......A..................G...........A........A......... 660
Tgu  ........C.C...........A.....G.....G.C.A.A.....TA.G.....A....A..CA..T.TG...C.G..A..C..............A..G......... 573
Gga  .......CC.C.........T.A..A..G..C..G..GA.A.....CA.......A....A..CA..T.TG...C.G..A..C..............A..G......... 618

Hsa  ATCTTCCCACTGCTGGGTTTGCATGAGGAGGGATTAAGAAAGTTCTCGGAGTACCTTTGCAAGCAGGTGGCCAGTAAAGCTGAGGAGAATCTGCTCATGGTGCTGGGGAC 770
Ptr  .............................................................................................................. 656
Ppy  ..T............................................A.............................................................. 770
Mac  ..A............C..C............................A..A........................................................... 488
Cja  ..A.................A.................C........A..A.............................................T............. 770
Mmu  ....................A........T..CC.G..C...........A.....G.....A.....C........G...............T.GT..........CT. 770
Rno  ....................A........T...C.G..C...........A.....G.....A..............G.....A............T..........AT. 770
Ocu  ..........................A...........C........A..A................................A.....C......T............. 770
Eca  ......................................C........A..A.........................................A..TT.......A..... 770
Cfa  ..................C...................C........A..A............................................GT............. 767
Bta  .................C....................C........A..A..T...................................C..C..GT....TT.A..C.. 770
Mdo  .........T.AT....G..A.....A..A..G.....C..A..T..A..A...T.A..T...........A.AC.....A..A..A..C.....TC.T...T....A.. 770
Tgu  .....T..T.....A..C..A.....A.....GC.G..C........T........C................AC.....A..A.....C....AGC.....A....... 683
Gga  .....T..C.....A..CC.A.....A.....GC.G..C........A........C................AG.....A........C....AGC.....A....... 728

Hsa  AGACATGAGTGATCGGAGAGCTGCAGTCATCTTTGCAGATACACTTACTCTTCTGTTTGAAGGGATTGCCCGCATTGTGGAGACCCACCAGCCAATAGTGGAGACCTATT 880
Ptr  .............................................................................................................. 766
Ppy  ...............................................................C.............................................. 880
Mac  ....................................................................................A.....A................... 598
Cja  .....................................................................T....................A................... 880
Mmu  ........................G...........G.....T..C..A..C..............C.....A........A..T.....A..C..C........T..C. 880
Rno  ...............A....................G..C..T..C.....C..C.................A........A........A..C...........T..C. 880
Ocu  ...............A........G..............C.....C.....CT......................C........T..T..A.....C.....A....... 880
Eca  ...............A.............................C............................................A........A.......... 880
Cfa  G............................................C........T........T..............A...........A................... 877
Bta  ...............A.............................G.................A........A..............T..............A..A..C. 880
Mdo  ............CA.A..G..A..T.....T..............A..A..............A........A.....T...........A..T.....A.......... 880
Tgu  ............C..TC.......T..............C..C..G..A..C..C..............T.......................C..C...........C. 793
Gga  ............C..TC.......T.................CT.G..A..C..C..............T..TG...................C..T.....A..T..C. 838

Hsa  ATGGGCCAGGGAGACTCTATACCCTGATCAAATATCTGCAGGTGGAATGTGACAGACAGGTGGAGAAGGTGGTAGACAAGTTCATCAAGCAAAGGGACTACCACCAGCAG 990
Ptr  .............................................................................................................. 876
Ppy  .............................................................................................................. 990
Mac  ................G............................................................................................. 708
Cja  ........................................................................................A..............T...... 990
Mmu  ....A......C......T..................T..A..A..........C...............C...A................................... 990
Rno  ...........C......T..................T..A..A..........CG.....A............A................................... 990
Ocu  ................T.T....................................G.....A................................................ 990
Eca  .......G........T....A......A..............................................................G.................. 990
Cfa  ....A...........T..C........A...................................A........G..............A..................... 987
Bta  ..........C.....T...........A.....C..........................A...........C.................G..........GA...... 990
Mdo  .......C..CC....G...............C.C......T....G......CA..................G.................G........T....GC... 990
```

```
Tgu     .C...........G.....C.....C.....GC.C...........G..C...C.G............T.T..G.TAG..AA..CG.GTG.....AG..GGG.GGCAG.A 903
Gga     .............G..G..C.....C.....GC.C.....A.GA..G.....TCAG.................G..G.....TG....AG.G........T...AGA... 948

Hsa     TTCCGGCATGTTCAGAACAACCTGATGAGAAATTCTACAACAGAAAAAATCGAACCAAGAGAACTGGACCCCATCCTGACTGAGGTCACCCTGATGAATGCCCGCAGTGA 1100
Ptr     .....................T........................................................................................ 986
Ppy     .....................T........................................................................................ 1100
Mac     .....................T.....................................................................................C.. 818
Cja     .....................T........................................................................................ 1100
Mmu     ..T....TC..C....GT..TT..............G.............T........G..G........TG..T..........T..............G..A..... 1100
Rno     .......T...C....GT...T..............G.............T........G..G.........G..T..........T..............T..A..... 1100
Ocu     .....A..G..C........T..............CG.............T........G......................................C........... 1100
Eca     ..T.....C..C.........A..............G.............A........G...........T...........A.......................C.. 1100
Cfa     ...........C.......G.T............................A........G...........T....................T...........T..... 1097
Bta     ..T........C....G....T.................T..........A..G.....G........T..T...................................... 1100
Mdo     ....A..T...C.....T.G.A.......G.G...AG..G.T..G.....T........G........T.....T.....A....................T........ 1100
Tgu     ....A...A..C.....T.G.A.........G....T.TG....G..G..T........G..G..T.....T..TT.A..A..A.....T.................... 1013
Gga     ....A...A..........G.A.........G....T.TG....G..G..T........G.....T.....A..TT.A..A.......................G..... 1058

Hsa     GCTATACTTACGCTTCCTCAAGAAGAGGATTAGCTCTGATTTTGAGGTGGGAGACTCCATGGCCTCAGAGGAAGTAAAGCAAGAGCACCAGAAGTGTCTGGACAAACTCC 1210
Ptr     ...........................................................................G.................................. 1096
Ppy     .....................................................................A........................................ 1210
Mac     ...C................G..............................G..............G..A.....G.................................. 928
Cja     ...G........T.......G..............................G.................A........................................ 1210
Mmu     A..G................G.........C...G.G..............G...........T.....A..........................C............. 1210
Rno     AT.G..............T.G.........C..TG.A..............G...........T.....A..........................C............. 1210
Ocu     ...G........A..T....G.........C.....A..............G...........T.....A........A.........................G..... 1210
Eca     ...G................GA........C....................G.................A..............A....................G.... 1210
Cfa     ....................G.........C........C...........G..........T......A.........................C.............. 1207
Bta     ...G...C............G...A.....C.....A..............G.................A.....C..A.................C............. 1210
Mdo     ...G...C.............A..A..A..C....................G....................G.....A........T..............T..G.... 1210
Tgu     ...C.....GA.A...A......GAC.A..A.TGG...................TG................G..............T..A..A.AC........G.... 1123
Gga     ...C.....GA.G...A......GAC.A..AGTAG......................A........C.......................A..A.AC............. 1168

Hsa     TCAATAACTGCCTTTTGAGCTGTACCATGCAGGAGCTAATTGGCTTATATGTTACCATGGAGGAGTACTTCATGAGGGAGACTGTCAATAAGGCTGTGGCTCTGGACACC 1320
Ptr     .............................................................................................................. 1206
Ppy     .............................................................................................................. 1320
Mac     .............................................................................................................. 1038
Cja     .....................................G............A........................................................... 1320
Mmu     .............A................................C...A.........................A.....G.......................T... 1320
Rno     .............A........................G.......C...A.........................A.............................T... 1320
Ocu     ...........T.AC......................C........G..CA...............................C.......................T... 1320
Eca     .............A........C...........................A....T...................................................... 1320
Cfa     .T...........A........C.......................C...A........................................................... 1317
Bta     .............AC.......C..............C.....TC.....A.C...................................C...........C......... 1320
Mdo     .....C.T.....C..A..............A.............AT..CA.......................C.A...........C........A........T... 1320
Tgu     ....C.....T..GC.......C........A.....C.......AC..CA.C...........A.......................C........T..CA......G. 1233
Gga     ....C.....T..GC.......C........A..A..C.......AC..CA.C.........C.A..T....................C........T..CA......G. 1278

Hsa     TATGAGAAGGGCCAGCTGACATCCAGCATGGTGGATGATGTCTTCTACATTGTTAAGAAGTGCATTGGGCGGGCTCTGTCCAGCTCCAGCATTGACTGTCTCTGTGCCAT 1430
Ptr     ...............T.............................................................................................. 1316
Ppy     ...............T.............................................................................................. 1430
Mac     ...............T....................................................A......................................... 1148
Cja     ...............T......................................................................T.....C................. 1430
Mmu     ........A......T.....................................C..A.............................T.A...C................. 1430
Rno     ...............T............................T...........A..............T..............T.A...C................. 1430
Ocu     ...............T.A........T........C..C....................A...........A.....A.....T........C................. 1430
Eca     ..C............T............................................................................C................. 1430
```

```
Cfa   ........A......T........................................A...........A...........T...........C................. 1427
Bta   ...............T......................C..........................C........C.....T...........C.....C.....C..... 1430
Mdo   ....................T...........A........G...........C..A.....T...........CT..........T.....C.....C........... 1430
Tgu   ..C..............C..C....................G.....T..A..G.................T....................A..............T.. 1343
Gga   ..C..............C..C.................C..G..T..T..A..G.................T...T.A.....T....A...A................. 1388

Hsa   GATCAACCTCGCCACCACAGAGCTGGAGTCTGACTTCAGGGATGTTCTGTGTAATAAGCTGCGGATGGGCTTTCCTGCCACCACCTTCCAGGACATCCAGCGCGGGGTGA 1540
Ptr   .............................................................................................................. 1426
Ppy   .........T..................................................T..............G..........................T....... 1540
Mac   ......................................................C.................C..G.....T............................ 1258
Cja   ...................................................C..C.........T.......C..A..G.......................T....... 1540
Mmu   .........T.......G.........AG...................A...........AA.A........C..A...........A..............T....... 1540
Rno   .........T..A....G.........AG...................A...........AA.A........C..A...........A..............T....... 1540
Ocu   ...A.....T.......................T....................C...T.............C..G...........A..............T....... 1540
Eca   .......................................A..C..C........C.....A...........C..G...........A..............T....... 1540
Cfa   .........T..T..T......................................C......A..........C..A...........A...................... 1537
Bta   ..........................................C..C......C.C.................C..A.........C.G..............T..A.... 1540
Mdo   ..........T.............A.....G..T........G...........C..A...........T..C.....T.....T.....A...T.......T....... 1540
Tgu   ........A.T.......C.......................G.......AC..C......AA.CA......C..A.....G............T.....A.A....... 1453
Gga   ........A.T.......C..A....................G........C..C......AA.CA.........A..A..A............T.....A.A..T.... 1498

Hsa   CAAGTGCCGTGAACATCATGCACAGCAGCCTCCAGCAAGGCAAATTTGACACAAAAGGCATCGAGAGTACTGACGAGGCGAAGATGTCCTTCCTGGTGACTCTGAACAAC 1650
Ptr   .............................................................................................................. 1536
Ppy   .............................................................................................................. 1650
Mac   ...................................................................................C.......................... 1368
Cja   .......T.............................G...................................T..A......C.............A...........T 1650
Mmu   .....................................G.....G........C..............C.....T..A..C...C.........................T 1650
Rno   .G.........................................G........C..............C.....T..A..C...C......................T..T 1650
Ocu   ....C................................G.....G.......................C.....T..A..C...C..AA............G......... 1650
Eca   .C........C..........................G.............................C...............C.A........................ 1650
Cfa   .G...........................................................T.....C...........C...C.A..............C......... 1647
Bta   .C..........G........................G.....G..C.....C..............C..C........C...C.......T........G......... 1650
Mdo   .G.....T.............................G...........T..C..............C..A..T.....C...C................CT........ 1650
Tgu   .C.....T.......................G.....G.....G..C.....C........T..A..C..A........C...CA......T........CT.A.....T 1563
Gga   .C.............................G.....G.....G.....T.....G........A..C...........C...CA......T.....A..CT.A.....T 1608

Hsa   GTGGAAGTCTGCAGTGAAAACATCTCCACTCTGAAGAAGACACTGGAGAGTGACTGCACCAAGCTCTTCAGCCAGGGCATTGGAGGGGAGCAGGCCCAGGCCAAGTTTGA 1760
Ptr   .............................................................................................................. 1646
Ppy   .............................................................................................................. 1760
Mac   .....................................................T.........................................T.............. 1478
Cja   ............................................A...................................................A............. 1760
Mmu   ..........................A..............T.............................T.................A.................... 1760
Rno   .................G........A..............T.............................T.................A.................... 1760
Ocu   .........................................G..T....................G...........T..........................A..... 1760
Eca   ..........................T..............G..............T........G............................................ 1760
Cfa   ........G....................G...................................G............................................ 1757
Bta   ..............C...............T....A.............................G............................................ 1760
Mdo   ...........T..................T......CA..C..............T..T.....A........A..T..C...........A................. 1760
Tgu   ........................ATG..C..A........TT...............G...A..AC.......A.AT..C..G..C........A.........A.... 1673
Gga   ........................ATG..C...........TT.A.....C.....TG....A..GC.T.........T.......T..A..A..A..A......A.... 1718

Hsa   CAGCTGCCTTTCTGACTTGGCCGCCGTGTCCAACAAATTCCGAGACCTCTTGCAGGAAGGGCTGACGGAGCTCAACAGCACAGCCATCAAGCCACAGGTGCAGCCTTGGA 1870
Ptr   .............................................................................................................. 1756
Ppy   .............................................T..................................................A............. 1870
Mac   .............................................................................................................. 1588
Cja   ......T.........................G..............................C........T..................................... 1870
Mmu   ..................A..T..T..............T.....................T..G.............TT....AG.......G........A....... 1870
```

```
Rno   ..................A..T..T..............T.....................T..G.........TT........AG.......G........A.......  1870
Ocu   .....................T..........................................G.A.................AG.......G................  1870
Eca   .....................T..........G................C......................A.................A..G................  1870
Cfa   ................C....T............................................A.....G....................G..A.............  1867
Bta   .....................T.........GG..............................C..A.....G....................G................  1870
Mdo   .........C.....T.....T..T..A...........T.....T...C.A........C.....T.....G..T.....T........A.........A.........  1870
Tgu   G........C..A...A....T..T..C..............T.....GC..........T..C.AT.......................A..C......A....G....  1783
Gga   T........C.....TA....T.AT..C..A.....G.....T......C..........TG.C.AT..C..G....A............A..C......A....A....  1828

Hsa   TCAACAGCTTTTTCTCCGTCTCCCACAACATCGAGGAGGAAGAATTCAATGACTATGAGGCCAACGACCCTTGGGTACAACAGTTCATCCTTAACCTGGAGCAGCAAATG  1980
Ptr   ..............................................................................................................  1866
Ppy   ...........................................................................................C..................  1980
Mac   ..........................................................A.....T.......................T.....................  1698
Cja   .T....................T..T....................................................................................  1980
Mmu   ......CT...C....A.....G....G.....................C..............T.....C........G..A........C........A.....G...  1980
Rno   ......CT...C....A.....A....G.....................C..............T.....C........G...........C.G...A..A.....G...  1980
Ocu   ......C....C.G..T..........................G........T.....A.....T...........C..........................A......  1980
Eca   ......C....C....T...............................................T.............................................  1980
Cfa   ......C...C..A..T..T...........T.................C..............T................................A.....A......  1977
Bta   ......C...CC....T...........T...........G.......G..................T........T.................T...............  1980
Mdo   ......T...CC....T..............T..A.......................A.....T.....A.....G....................T........G...  1980
Tgu   .....TTA..CC....T.......................G..G....G...............T..T..C.....C..G.........G.C..T.....A.....G...  1893
Gga   .....CTA..CC.....A.........G...A................G.........A..T..T..T..C.....G..G........TG.CC.......A..T..G...  1938

Hsa   GCAGAGTTCAAGGCCAGCCTGTCCCCGGTCATCTACGACAGCCTAACCGGCCTCATGACTAGCCTTGTTGCCGTCGAGTTGGAGAAAGTGGTGCTGAAATCCACCTTTAA  2090
Ptr   ..............................................................................................................  1976
Ppy   ...............................................T..G........C..................................................  2090
Mac   ..G........................................................C..................................................  1808
Cja   ..........................A................................C..............G...................................  2090
Mmu   ..........................A........T.......................C...........T..T..AC....A........C..............C..  2090
Rno   ...................................T...........T...........C...........T..T..AC....A........T..............C..  2090
Ocu   ..........................A........T.....T..G..T...........C...........T..T................................C..  2090
Eca   ..G............G..........A.................G...A....T.....C...........A..........................G........C..  2090
Cfa   ..........................A........T........G..............C...........A.............G........................  2087
Bta   ..C............G..........A.................G...A..........C........C..A..T.......................G........C..  2090
Mdo   ..T...........TG.G..C..T..C........T.....T..C...A....T.....C.....CA....GA.T.................T..............C..  2090
Tgu   A.............TG.A.....T..A..G..T..T..T.CT..C..T..T..T.....C..T..CA.A...A.T..AC...........C....C...........C.G  2003
Gga   A.............TG.A.....A..A.CG..T..T..T.A...T..T...........C.....CA.A...ACG..AC...........C.T..C.......G......  2048

Hsa   CCGGCTGGGTGGTCTGCAGTTTGACAAGGAGCTGAGGTCGCTCATTGCCTACCTTACCACGGTGACCACCTGGACCATCCGAGACAAGTTTGCCCGGCTCTCCCAGATGG  2200
Ptr   .......................................A......................................................................  2086
Ppy   .........C.................A...........A......................................................................  2200
Mac   ...........................A...........A......................................................................  1918
Cja   .........C........A....................A...............................................A..............T.......  2200
Mmu   .A.......C....................A..CC....A........T...........T.....A.......................C.....C.....T.......  2200
Rno   .A....A.......................A..CC....A........T...........T.............................C...................  2200
Ocu   T........A.....A.......................A.....C..............A.....T.............................A.............  2200
Eca   .......................................C...G..................................................................  2200
Cfa   .........C..............T..............A..A........T..................................................T.......  2197
Bta   .........C.....A.......................A..............C.......................................................  2200
Mdo   .A..........C..........................C........T...........A................................TA.A..T..........  2200
Tgu   .A....C................................C.....A.....T..C.....A..C....A.....G......T..............T..T.....A....  2113
Gga   .A....T..C.....A.......................T.....A........C.....A..C..........T......T...........A..T..T.....A..A.  2158

Hsa   CCACCATCCTCAATCTGGAGCGGGTGACCGAGATCCTCGATTACTGGGGACCCAATTCCGGCCCATTGACGTGGCGCCTCACCCCTGCTGAAGTGCGCCAGGTGCTGGCC  2310
Ptr   ................................................................G.............................................  2196
Ppy   ................................................................G..........................G..................  2310
```

```
Mac    .......................................................C...................................................... 2028
Cja    ............................G........T.................C....................T................................. 2310
Mmu    .T..A.....T..C..A...........T.....A..A...........TG.T..C..T.....T.....A..........................A...........T 2310
Rno    .T..A.....T.....A.................A..A...........TG.T..C..T.....T.....A..........................A.....A.....T 2310
Ocu    .T..A........C..............A.....A..A...........TG....C.........C....C.............................A........T 2310
Eca    ....A................................A...........TG....C..............C.....T.................A........A..A..T 2310
Cfa    ....A..............A.....C..G........A...........CG....C..T.............................C.....A..............T 2307
Bta    ....G................................G...........CG....C..G.....GC................G........................... 2310
Mdo    .T...........C..A..AA.......A..A.....G.....T.....CG....C..A.....TC.C...........G..T..A.....G............T....T 2310
Tgu    .............C.....AA.......G.....T..G...........C..A..C..G..T...C.C..C...........T..G..C..G..C..G...........T 2223
Gga    ...................AA.......A........G...........C.....C........GC.C..C...........T..G..A..G..C..G.G....T....T 2268

Hsa    CTGCGGATAGACTTCCGCAGTGAAGATATCAAGAGGCTGCGCCTG 2355
Ptr    ............................................. 2241
Ppy    ............................................. 2355
Mac    .....C....................................... 2073
Cja    .....C....................C.................. 2355
Mmu    .....C.............A...G....................A 2355
Rno    .....C.............A...G..C.................. 2355
Ocu    .....C...........TG.C..G..C.................. 2355
Eca    .....C........T.....C..G..C.................. 2355
Cfa    .....C..............C..G..C.................A 2352
Bta    .....C..............C..G..C.................. 2355
Mdo    ..A..A..T..T..........................T...T.. 2355
Tgu    ..C..C..C...........C..G..C......C........... 2268
Gga    ..C..A.AG..T.....TGA...G..C.................. 2313
```

COG5

```
Hsa    TCTGCTGCTGACGACATCAACCCGGCACCTGCCAACATGGAAGGTGGCGGCGGCAGCGTCGCTGTAGCTGGCCTCGGAGCTCGAGGCTCTGGAGCGGCTGCAGCTACAGT 110
Ptr    ........................................................................................................G..... 110
Ppy    .....................G...........................A........................T.............................G..... 110
Mac    .....C...........................................A...........T...G...........G........T........T........G..... 110
Cja    ..G.T............................................A...T.A.........G...........G...........C....T.........G..... 110
Mmu    ------------------------------------...........A.A.A...CAA.TT.C.....A....GT..G...TCG..T..C.C..TA.TG..G..G..C.. 74
Rno    CGAT.GCAA....T...........T..T..................A.A.A...CA...T.G..G..A....GT..G...TCG..T..C.C...A.TG..G..G..C.. 110
Ocu    GGG..CA......C...................................A............C...T.......T..G...TG.......C.......A.G..G..... 110
Eca    C.C..........C.....C...................A.........A....G....T.....G.......AT..G...TG.........AGC........G..C.. 110
Cfa    G.GT.C.......T.....G...........................T.A....G..........G.......A..G...TG....G...G.....G.....G..... 110
Bta    ---------------....CT..C.T...A.................T.A....G.....T..A.G.......GT..G...CG..T..C.....T........C..T.. 95
Mdo    GGGCTACT....AT.....GT..T.....................------ATTG.A..T....GGT....GGCT.AG....TT.TT............C..ATCT.G.. 104
Tgu    ------------------------------------------------------------------------A.G.AG.AGGCG..G---.CC..CC.C..CC.GG.GC. 35
Gga    ------------------------------------------------------------------------A.G.AG.AAGCG...GG......CC.G..TC.GG.GC. 38

Hsa    CCGGGAACTTCTGCAGGACGGGTGTTATAGTGACTTTTTAAACGAAGACTTTGATGTAAAGACTTATACTTCTCAATCTATTCATCAAGCTGTAATTGCTGAACAACTAG 220
Ptr    ....................A......................................................................................... 220
Ppy    ....................A......C.................................................................................. 220
Mac    ....................A.............................................C........................................... 220
Cja    ...A................A......................................................................................... 220
Mmu    G.A..CGA.C..........AC...........A........T.................A.....C.....C.............................G....... 184
Rno    G.A...G..CT.........AC..C..C.....A.....G.GT.................A..G..C.....A.............................G.....G. 220
Ocu    ......G..C..........A.........A......C....T..G...........G...AG....C.....A.....A..........................G.... 220
Eca    ......G..C..........A...........CT......................................C..G.................................. 220
Cfa    ......G..C..........A......C.....T........T..............C..............C..G.....C............................ 220
Bta    ......G..C..........A............T........T........C.......................................................... 205
Mdo    .........G..........AA.....CTTG..T..C....GT........C................................C..G.....G..A........G.... 214
```

```
Tgu          .GCC..G..G.G.GC.....A.......GC......C..C.GA..............G..AG....C.....C..G..C..C.....G.....G..A.....G.....G. 145
Gga          GGCA..G..G.A.GC.....A.......GC......C..C.GA..G...........G..AG....C..G..A..G..G..C.....G..G..G..A..C..G..G..C. 148

Hsa          CAAAACTTGCCCAAGGAATCAGTCAGTTGGACAGAGAACTACACTTACAGGTTGTTGCAAGACATGAAGATTTACTGGCACAAGCAACTGGGATTGAGTCGTTGGAAGGT 330
Ptr          .................................A............................................................................ 330
Ppy          .................................A............................................................................ 330
Mac          .................................A............................................................................ 330
Cja          .................................A.........T.................................................................. 330
Mmu          ................G.........C....T.A..........C.............................T.........................T......... 294
Rno          ....G...........G.........C....T.A...G......C....................................................A..T......... 330
Ocu          ...............................T.A......G...C.......................................................T......... 330
Eca          ...............................T.A..........C.......C..............G...........G....................T......... 330
Cfa          ...............................T.A...G......C............................G..A.......................T......... 330
Bta          ...............................T.A..................C.....................T................A........T......... 315
Mdo          ..........A........A...........T.A..........C.......A......C.G............T....G..G.....A........A..T..A...... 324
Tgu          .T........T.....T..T.............A...G..G..T.....A.....................C.GT....C...........A.....A..CC........ 255
Gga          .G..G...........T.....C...C....T.A...G..T...G....A..A.................CC.GT....T...........A.....A..CC.A...... 258

Hsa          GTTCTTCAGATGATGCAGACGAGAATTGGGGCTTTACAGGGAGCTGTTGATAGGATAAAAGCAAAAATTGTTGAACCATACAATAAGATAGTTGCCCGGACTGCACAACT 440
Ptr          .............................................................................................................. 440
Ppy          ..................................................................................................T........... 440
Mac          ..................................................................................................T........... 440
Cja          ...................T......................................................T.......................T........... 440
Mmu          .............................A...C......................G...T....G....................A.....A.....T........... 404
Rno          .............................A...........T..............G...T.............G...........A.....A.....T........G.. 440
Ocu          ....................A........A...........G..............G....................G.....C..A.....A..A..C........G.. 440
Eca          .........C................C..............G...C..........G.................G..G........A.....A.....T..C.....G.. 440
Cfa          ....................A...................................G..............G..............A.....A................. 440
Bta          ..C.................A.......................C...........G............A....G..G........A.....A.....T.....T..... 425
Mdo          ..C.....A...........C...........CC.C.....T...A.C..C.....T.....T...........T..T.....C..............A..A..C..G......T. 434
Tgu          ..C..C..A........A..T........T...C....AA.CA..........A..T.G..T............C..C.....C..A.....GT.T..C..A..T..G.. 365
Gga          ..C..C..A...........C..G.....T...C....AA.CA..........A..T.G...C...........T..C.....C..A.....GT.T..C..G..T..... 368

Hsa          AGCAAGACTTCAGGTTGCCTGTGATTTGCTTCGGAGGATTATTCGTATCTTGAATCTCAGTAAGAGACTCCAAGGACAACTGCAAGGGGGAAGTAGAGAGATAACAAAAG 550
Ptr          .............................................................................................................. 550
Ppy          .............................................................................................................. 550
Mac          ....................................A......................................................................... 550
Cja          C...................................A............C............................................................ 550
Mmu          G...................................A..A.....C.....AT.............G.....G..............A..G................... 514
Rno          G.....G.............................A..A.....C.....AT...................G..............A........G............. 550
Ocu          .............................................C...C..T.....................................TT.................. 550
Eca          G...................................A..A.....C.....AT.....................................G................... 550
Cfa          ....................................A..A.....C......T......C................................................G. 550
Bta          ....................................A..C.....C.....AT...............................G......................... 535
Mdo          G.....G........A....................A..A..A..C......T.........................G.....G..A...................... 544
Tgu          .....A......A.C.........C.....G........A..A..C......T................G........G..T.....A.....C................ 475
Gga          .....AG.......C.........C.....G........A..A..C......C................T...........A.....A.....C..G............. 478

Hsa          CTGCTCAGAGTCTCAATGAACTTGATTATCTTTCTCAAGGAATAGATCTTTCTGGAATAGAAGTGATAGAAAATGATCTACTTTTTATTGCAAGAGCCCGACTTGAAGTG 660
Ptr          .............................................................................................................. 660
Ppy          .............................................................................................................. 660
Mac          ............................................................................C................................. 660
Cja          ..............................................C...A.......C................................................... 660
Mmu          ................................................................A.....G...........C..............T............ 624
Rno          ..................................G.............................A.....G......T....C..............T...........A 660
Ocu          .A..............................................................A................................T............ 660
Eca          .............G............................G......C..............A................................T............ 660
```

```
Cfa       .............T..................................................A................................T............ 660
Bta       ..............................................C.......................................G..........T............ 645
Mdo       .A...........A.............................C.....C..............A............T....C...G....C.....T...........T 654
Tgu       ....C.....C.............................G........G.................T........C..G.................T..T.....G... 585
Gga       ....C.......................C...........G........G..............A..T........C..G.................T..G..G..G... 588

Hsa       GAAAATCAAGCTAAGCGCCTACTAGAGCAGGGTTTGGAGACTCAGAATCCAACTCAAGTCGGAACAGCTCTTCAGGTTTTCTATAATCTTGGAACTTTGAAGGATACTAT 770
Ptr       .............................................................................................................. 770
Ppy       .............................................................................................................. 770
Mac       .............................................................................................................. 770
Cja       ..........................A......................................................C............................ 770
Mmu       .....C...........T..G..G.....A...G.......C..............G..T.....................C....C..C..G...........G...G. 734
Rno       .................T..G..G.....A...G.......C..............G..T.....................C.C.....G..G...............G. 770
Ocu       ...........C.G......GT.G..A..A...C.A....................G..T..........................................C.....G. 770
Eca       .......................G.........G...................C.....T..G..................C.........................C.. 770
Cfa       .......................G..A.....GG.A.................C.................C.........C............................ 770
Bta       .......................G.........G...................C..G........T...............C......................C..... 755
Mdo       ..............A...T..T.G.....A..AG.........................G.....................C...................A........ 764
Tgu       .....C..G..C..AA.GT.G..T.....A..CG................T........G.....T...............C..........G..............C.. 695
Gga       ........G.....AA.GT....T.....A...G............G...T........G..G..T..............T.....C.........C..........C.. 698

Hsa       TACCAGTGTTGTGGATGGATATTGTGCTACTTTAGAAGAAAATATCAACAGTGCATTAGACATAAAAGTTTTGACTCAGCCTTCCCAGTCAGCTGTGAGAGGGGGACCTG 880
Ptr       .............................................................................................................. 880
Ppy       .............................................................................................................. 880
Mac       ......C.................CA..CTGA.CT.TC.C.GGG.TG.TG.AAT.ACTC.T..TG..T.AA.A.GAATA..CAGTA..AGTTGGA.A............. 880
Cja       .....A...........................G...........................G......................T.........T..........T.... 880
Mmu       .........C........C.........G....G.....C.GC.......A......G...G.C.................................C....A..C.... 844
Rno       .....A............C.........G.C..G.....C.G........A......G...G.C...............................C......A..T..C. 880
Ocu       .........C...........C...........G........C....................C.................A..A....................T..C. 880
Eca       .....................CC.....G..........G..C....G......G........C.............................................. 880
Cfa       ........................................C.C....................C......C.............G......................... 880
Bta       .....A......................G.AA............................T..C.............................................. 865
Mdo       .....A...C........G............C..........C..A....A............C...............T.......AA.....A.A.A.---..T.... 871
Tgu       .G.T.A...G...........C..CA.AGTGC.G.....G..C..A..A.A...T..G..T..C.....G.....C..A..A..T..AA...TCACA...---..C.... 802
Gga       .G.T.A...G...........C..CA.AGTCC.G..G..G..C..A..A.A...T..G..T..C.....AC....C..A..A..T..AA.T.T.ACA...---..C.... 805

Hsa       GACGATCTACCATGCCAACCCCAGGAAATACTGCAGCTTTGCGTGCCTCATTCTGGACCAATATGGAGAAACTTATGGATCATATTTATGCTGTTTGTGGACAGGTACAA 990
Ptr       ..................................................C........................................................... 990
Ppy       ..............................................T...C........................................C.................. 990
Mac       ..............................................T...C.......G................................................... 990
Cja       ..............................................T...C........................................................... 990
Mmu       .G...G.CG..........T.......GC..A....G...T..G..T..CC................A..............C..C.G.....C................ 954
Rno       .G...G.C...........T........C..G........T..A..G..CC..........C.....A.................C.G.....C.........A...... 990
Ocu       ..A.....................................C.....T..C..G.....................G.....T......G..................G... 990
Eca       ....C.......................C...........T.....T..CC.......................G............G.A.................... 990
Cfa       ..........T.................C...........C.....T..CC.......................G............G.A.................... 990
Bta       ...................T.....G..C...........T.....T..CC..........C............G............T.A..........T.....G... 975
Mdo       .....G.C..T........T.....G..C.....T..C..C.....TA.TC.......A............T.G........A....G.....CA...........T... 981
Tgu       .TA..G..G.T........T..T..G..C..A........T..A..AG.TC.A.....T..C...........C........G..C.G.....CC..C...........G 912
Gga       .TA..G..G.T........A..T..G..C..A........T..G..AG.TC.......A..C...........C........G..C.G.....C............G..G 915

Hsa       CATCTACAAAAAGTATTGGCCAAGAAGAGAGATCCTGTTTCTCACATTTGTTTCATTGAAGAAATAGTTAAGGATGGACAACCGGAAATTTTCTACACATTTTGGAATTC 1100
Ptr       ...................................................................................T.......................... 1100
Ppy       ...................................................................................T.......................... 1100
Mac       ............................................T......................................T.......................... 1100
Cja       ...............C..........................................................G........T.......................... 1100
Mmu       .................AA...............................................A.............G..T........A....TG.........G. 1064
```

```
Rno        .................AA.............C...............................................G..T........A....TG.........G. 1100
Ocu        ........G........AA.............................................................G..T........A..T.AG........... 1100
Eca        ........G...A....A.................G...............................................T..G.....A.....G........... 1100
Cfa        ............A....A..........................T......................................T........G.....T........... 1100
Bta        ............A....AA..........G........C.........................................GG.T........A..T.............. 1085
Mdo        ...T.G...........A..............C........A..T.....................C.A........C...T.T........A..T..C.........GT 1091
Tgu        ...T.G..G..........A......A...........G..A..T..C..C.....A..G..G..............C..GT.T..T.....G....A........C.G. 1022
Gga        ..CT.G..G...C......A......A.........A....A..TG.C..C........G....................GT.T..T.....G....A........C.G. 1025

Hsa        AGTTACTCAGGCACTTTCTTCTCAATTTCATATGGCAACAAACTCTTCGATGTTTTTGAAGCAGGCATTTGAAGGAGAATACCCTAAATTATTACGTCTTTATAATGACT 1210
Ptr        ................................................T............................................................. 1210
Ppy        ................................................T...................................C......................... 1210
Mac        ....................................G...........T...................................C....................C.... 1210
Cja        .....................C..G.......................T.......................G...........C......................... 1210
Mmu        ...C..C.T...............C......TCA..............T..............A...........T............C.C................... 1174
Rno        ...C....T......C..C.....C......GCA..G...G.......T..............A...........T............C..........C.......... 1210
Ocu        .........A..C..............C..CC.......C........TG.......A..A...T.........................G..G................ 1210
Eca        .........A...................G..........G.T.....C...........A................................G................ 1210
Cfa        .........A...................GC.....T...G.T.....T...........A..A......................................C....... 1210
Bta        .........A....................G.CA........T.....T...........A.......................A........G..G............. 1195
Mdo        ...G...G.TA.C.....C....G......A.CA..G.....T.....TG.............A...........T.....T...........G.....C.......... 1201
Tgu        ...A..C...A.......A.....G.....GTCA......G....C..T..............A...........................C.GA.G............. 1132
Gga        ...A......A.T.....C.....G.....GTCT......G.......T...........A..A..T........................C.GA.A............. 1135

Hsa        TATGGAAGCGTCTTCAACAATACAGTCAGCATATCCAAGGGAATTTTAATGCAAGTGGAACTACAGACCTCTATGTTGACCTACAACACATGGAAGATGATGCACAAGAT 1320
Ptr        .............................................................................................................. 1320
Ppy        .............................A................................................................................ 1320
Mac        .............................A..........A..................................................................... 1320
Cja        ......................TG.....A......................C...................G..............T...................... 1320
Mmu        ................G..G.C...C...A...C...G....C...C.G.C.......G...C.T.......G...G.....C.C............C..CA.......C 1284
Rno        ................G..G.....C...A...G......AGC...CGG.A..C....G..AG.C..........C......C.CTG..G....C..C...........C 1320
Ocu        .......................CA....A..........A............G......GC.....T...........G.....G...........C............ 1320
Eca        .........................C...A....T.....A..G......................................C..................A.......C 1320
Cfa        .........................C..AA....T.....A..G..........................T.................G.....C......A.......C 1320
Bta        .........................C..AA....T.....A.....................G............C....T.........C..........A........ 1305
Mdo        .......A..............T.A...AA....T.....T.........T..........C...........CA....T........G..A..........TG.....C 1311
Tgu        .G........C..C........T.....AA....T..GA..........CA.............T..T..T.T..C...G........A..A.....A........G..C 1242
Gga        .G........C..G..............AA....T..GA..........CA.........G.C.T..T....T..C...A..G.....G.....G..A........G..C 1245

Hsa        ATATTCATACCAAAAAAGCCAGATTATGATCCAGAAAAGGCTTTGAAAGACTCACTACAACCCTATGAGGCTGCTTATCTATCAAAATCCTTATCTCGACTCTTCGATCC 1430
Ptr        ............................................................................................G................. 1430
Ppy        .............................................................................................................. 1430
Mac        ................T............................................................................................. 1430
Cja        ......G.............G......................................................................................... 1430
Mmu        ..G....G..TG....G.................................T.....G.....A..................C.........C...A..C........... 1394
Rno        ..G.C..C..TG..G.G...G....................G........T..C..G.....T..............C...C.........CG................. 1430
Ocu        G.............G.G........................C........T.....G..G..T..............C...G......................T..... 1430
Eca        .........A..........G.....................C.............G.................C......G.........................C.. 1430
Cfa        ..............G...........C..C..........................G...........A.........T..........T..............T..... 1430
Bta        G..........G..............................................................A...T..........A..............T..... 1415
Mdo        ........G.AG.......AG.......................A.........T.G.....A.....A..A.....C...T.......A..............T..... 1421
Tgu        ........G.A......TGA......C..............C........T.....T....AG.....A.....C...T..G...................G..T..C.. 1352
Gga        ........G.A.....CC.A.........C...........C........T..G..G....AA...............T..G......................T..... 1355

Hsa        TATCAACTTGGTTTTTCCCCCGGGTGGTCGTAATCCTCCTTCCTCTGATGAACTTGATGGTATTATTAAAACTATAGCAAGTGAACTAAATGTTGCTGCTGTTGATACAA 1540
Ptr        .............................................................................................................. 1540
Ppy        ................................................A............................................................. 1540
```

```
Mac     .....................T........................................................................................ 1540
Cja     .....................A.........................................C...................................G.......... 1540
Mmu     C.................T.....A..A.......................G..C.....A..C.C...G......A..........C.....A............G... 1504
Rno     C.................T..T.....A.....C.................G........G..C.......................C.....A............G... 1540
Ocu     .....................T.....G..............A.................C..C.....G..............G......A.A.....C......G... 1540
Eca     .....................T....................TG.G...........A.....C........C..CA................A............G.C. 1540
Cfa     .....................T........C...........TG.A...........A.....C..........................C..A........A...G..G 1540
Bta     .....................T....................TG.............A.....C..............G...........C..A............T... 1525
Mdo     ......TC....C..............C..G.....A.....TC..........C..CA.C..C........C.....C........T.....G.....A....CT.... 1531
Tgu     C.....TC.T..C.....T..C..A.....G........C...........G.....CA.C..C..............C.....G........G.....C......C..G 1462
Gga     C.....TC.C..C..C..T..T..A.....G........C..T........G.....CA.C..C........G.....C.....G........G........C...C..G 1465

Hsa     ACCTCACATTAGCTGTGTCAAAAAATGTGGCAAAGACCATCCAGTTATACAGTGTAAAATCAGAGCAGCTTCTCTCCACACAAGGAGATGCAAGTCAGGTGATTGGGCCT 1650
Ptr     ..............................................G............................................................... 1650
Ppy     .........................C.................................................................................... 1650
Mac     ..........................................................G................................................... 1650
Cja     .............................................................................................................. 1650
Mmu     .................................................TGC...G........A...........T..T..............C...........A... 1614
Rno     ...............................G.................TGC...G..............G.....T..T..............C............... 1650
Ocu     ...............................C.................TG........................................G..............A... 1650
Eca     ......................G...........................G..........C........G....G...G.............................. 1650
Cfa     .....................G...C........................G....T.....G...........A................................A... 1650
Bta     ..............................................G..TG.G.................G..............G....................A... 1635
Mdo     ....T..T........A.......................A........TG....G.....T.....A.....T..T...........A........A........C... 1641
Tgu     ..T.G.GT.........G.C...........G........T...C.T...G......................G..T..T...........C..C..............A 1572
Gga     ......GT.........G.C........A...........T...C.C...G....G..G..T..A........G..T..T...........C..C...........A... 1575

Hsa     CTTACTGAAGGACAGAGAAGAAATGTGGCAGTAGTGAATTCATTGTATAAGTTGCACCAATCAGTAACAAAGGTTGTTTCCAGTCAGAGCTCATTCCCACTGGCAGCTGA 1760
Ptr     ..........................................C................................................................... 1760
Ppy     ....T.....................T................................................................................... 1760
Mac     ....................................................................................................C......... 1760
Cja     ..........................................C................G..............C..............T..........C......... 1760
Mmu     ..C..G..........AG..........GT..G.....C..CC...T....C.......G..................G.................T..GCAA....C.. 1724
Rno     ..C.............AG..........GT..G.....C..CC...T....C.......G..............G...G.................T..GCA........ 1760
Ocu     ................A......C........G..C..C...C.A........A....................G........C........C......GCA........ 1760
Eca     .....C.....G.....G...........G............C........C..........C...........G........C......G....T..T.CA........ 1760
Cfa     .....A.....G..............A...............C........C....T..G..TA....G.....G..............T..........CA........ 1760
Bta     .....C.............................A......C.A.............................G.........................CA........ 1745
Mdo     .....A.....T........G.....A........T..............A..A...........T.T......A.....T.AC...GT..........GCA.....A.. 1751
Tgu     ..G..A..G..C.....G..G..C.....T..G..T.....TC....C..AC.......GG....TCTG......A..A................T...GCA..T..... 1682
Gga     ..C..A...........G..G........T.....T......C.A..C..AC.A......G....TCTG.........A...A.....A..........GCA..T..... 1685

Hsa     GCAAACTATAATTTCAGCTCTAAAGGCTATTCATGCTCTTATGGAAAATGCTGTGCAACCCTTACTCACTTCTGTGGGAGATGCTATAGAGGCCATAATCATCACCATGC 1870
Ptr     .............................................................................................................. 1870
Ppy     .......G...................................................................................................... 1870
Mac     ..................................A........................................................................... 1870
Cja     ...................T.........................................................A................................ 1870
Mmu     ......C.....G.....C......A.A.....C.A........GG.....CA.A..G.....G..G..G..A....C......G..C...................... 1834
Rno     ............G.....C......A.A.......A........G......CA.C..G.....G..G..G..C....C......A..C........C.........G... 1870
Ocu     ...G..............C..C...A.G.....CA....G....G............G........G..C..C.....G........C.....T..C............. 1870
Eca     ......G....C......C......A.C..C..C.T........G............G.....G..G.....A..................................... 1870
Cfa     ......C.....C.....C..T...A.A.......T........G..G..................A.....A...........C....................T.... 1870
Bta     ..................C........G..CT...T........G..................G..G...........T..C..C..T.....T..C........T.... 1855
Mdo     .G.....G..GC.A...........A.A..C....A........G...............T..G..G.....A..A......T.A.................T..T.... 1861
Tgu     ......AG.C.CAA.T..CT.......AG.C....A...A....GT.G......T..G..GC....G.AC.....A......T.AG.G..A..T..T........T.... 1792
Gga     .G....G..C.CAA.T..CT.G.....AG.C....A...A....GT.G......T.....A.....G.AC.....A......T.AG....A..T..T........A.... 1795
```

```
Hsa    ATCAAGAAGACTTTTCTGGGTCATTATCCAGCTCAGGAAAACCTGATGTTCCTTGTTCTCTGTACATGAAGGAGCTACAAGGTTTCATTGCCAGAGTTATGAGTGACTAT 1980
Ptr    .............................................................................................................. 1980
Ppy    .............................................................................................................. 1980
Mac    ..................................................................................C........................... 1980
Cja    ..........T...............C............G..........................................A........................... 1980
Mmu    ....G.....T.........G...C.AG............................C...........A..................................A......C 1944
Rno    ....G.....T...........C...A.T...........G..G..............C...........A...........C...................A......C 1980
Ocu    ....G.....T..C........GG.G...G..........G..C..C.............................G...............C................C 1980
Eca    ..........T..C..............GA...C........G..................................G.............................C 1980
Cfa    ..........T..............A................G..................................T.............................C 1980
Bta    ..........T........A......C...AT........G...........C........C...............T.............T...............C 1965
Mdo    ....G.....T........A......C.T..............A.....C..C..C.....A.......................T...........G....A......C 1971
Tgu    .C..G.....T........A..........A............A.....C...........C........A.....T..G.....T.....A.....C...........C 1902
Gga    .C..G.....T........A.......................A...........................A..A.....G.....T..A.GA.....C...........C 1905

Hsa    TTTAAACACTTTGAATGCTTGGATTTTGTCTTTGACAACACTGAGGCTATTGCCCAAAGAGCTGTTGAACTTTTTATCCGCCATGCCAGTCTCATAAGACCTCTTGGTGA 2090
Ptr    .............................................................................................................. 2090
Ppy    ..............................................................CA.............................................. 2090
Mac    ...........C.............................C....................AA.............................................. 2090
Cja    ..............................................................AA.............................................. 2090
Mmu    .............................T..............A.................CA...............A............................ 2054
Rno    ..C..G.......................T..............A.................CA...............A...................C........ 2090
Ocu    ..C........C.......CA....................G..A............C....AA...............A....T....................... 2090
Eca    ...........C..G.......................T.....A.................AA.C.............A............................ 2090
Cfa    .............................T..............A.................AA...........T...A............................ 2090
Bta    ...................C........................A.................CA...............A.C...........T.............. 2090
Mdo    ....G............T.CA..G.................A..A..C........G.....AA.CA........G.T..TA...........T.......A........ 2081
Tgu    ..C.G...T.....G..T..T..C...........T........A..C..G..T........AA....G.....C..T...A.......C..A..CC.G..C..C..... 2012
Gga    ..C.G...T.....G.....T..C...........T........A..C..G..A........AA..........C..T...A..........A.....G..C........ 2015

Hsa    AGGTGGGAAAATGCGACTTGCTGCTGATTTTGCACAGATGGAGTTGGCTGTGGGTCCATTCTGTAGACGAGTATCTGATTTAGGAAAGTCCTATCGGATGCTGAGATCAT 2200
Ptr    ............................................................................................................G. 2200
Ppy    ............................................................................................................G. 2200
Mac    ................................................................................................A............. 2200
Cja    ...............................................................C.............................................. 2200
Mmu    ..........C...........................................C..CC.T..C..G.....G..G...C.G.......................G.... 2164
Rno    ..........C.............C..................C..........C...C.T..C........G..G.....G...........C................ 2200
Ocu    ...........................................C.............C.....C........G..C..C.....G....................G.... 2200
Eca    .A.............................................................C...A............................A........G.... 2200
Cfa    ......C...C.T....................T.............................C...A............................A........G.... 2200
Bta    ...............................................................C...A.............G..............A........G.... 2185
Mdo    .................................C........A..........C....C.T........G.....C..CC....G........C..CT.A..C.....T. 2191
Tgu    .........G.....C..G..A..A....................A..A..A.CA..GC.G...C.G.....C.....C..G.....A..T..CA.ACA......G.... 2122
Gga    .........G.....G.....A..A....................A..A.....CA..GC....CC.C.....C.....CC....C..A.....CA..CA......G..G. 2125

Hsa    TCAGACCTCTGCTCTTCCAGGCAAGTGAACATGTAGCCAGTAGTCCTGCATTGGGGGATGTGATTCCGTTCAGCATCATTATTCAGTTTTTGTTCACGAGAGCACCCGCT 2310
Ptr    ....G..................................A...................................................................... 2310
Ppy    .......................................A...................................................................... 2310
Mac    .......................................A..........C........................................................... 2310
Cja    ....................A..................A..........C................A......................................T... 2310
Mmu    .T..G..C............A.............G...GAC..C..C...G....A..CA.T..A..A...........C.........C.......A........A... 2274
Rno    .......C............A....C........G..AGAC..C..G...G....A..CA.T..C..A........T..C.........C.......T........G..G 2310
Ocu    .......A.....G......A.C...........G....AC..C..A...C.......CA.C.....C......G..G.C..C.....C.........C.C.....T... 2310
Eca    .......G............A.C.A......C..G.....C.....A..CC.......C........C.......C.G.C.........C.............G..T... 2310
Cfa    .......C............A.......G..C..G...........C...C....A..CA.T...................................C............ 2310
Bta    .......A............A..........C..G.....C..C......C.......CA.A.....A.............................A.........C.. 2295
Mdo    ..C.............T...A....C.............A...C..A...C........A.T.....A........T........T..C..A..T..A.....T..A..C 2301
```

```
Tgu      .......A.....G......A.C.....G...A.T........C..A..TC....A..G..T.....A........T...C.......C..A..T..A........AC.A 2232
Gga      .......G.....G......A.C.....G...A.T.....C..C..A..CC....A..G..T.....A........T...C.......C..A..T..A........AC.G 2235

Hsa      GAACTGAAATCTCCTTTCCAGAGGGCAGAGTGGTCCCACACACGCTTCTCTCAGTGGCTGGATGACCATCCATCTGAAAAGGACAGGCTCCTCCTCATCAGGGGAGCCCT 2420
Ptr      .............................................................................................................. 2420
Ppy      ..G....................................G.............................................A........................ 2420
Mac      ..G....................................G.............A...............................................A........ 2420
Cja      ..G....................................G..............................................A....................... 2420
Mmu      ..G........C..C..............A.....T..TG.T........C..............T..C....................G......C............. 2384
Rno      ..G........C..C..............A........TG..........C..............T..C..........................TC...........T. 2420
Ocu      ..G.....G..C..C...........G............G.G..T.....C...........C........C.....G...........G.................... 2420
Eca      ..G.....G..C..C.......................TG....T.....C....................G............C......................... 2420
Cfa      ..G........G.....T.....A...............G..........C........A............................................C..T.. 2420
Bta      ..G........C..C......CA...............TG..........C..............T..C..T................................T..T.. 2405
Mdo      ..GT...................A..............TG..A.A.A...C..............T........A.................T..T.....A........ 2411
Tgu      ..G..A.....A..C...........T.........ATTG.C....A...C........C.....T........................GCT.....AC.T..T..A.. 2342
Gga      ..GT.A.....A..C...........T.........ATTG.C....A............C.....T.....T.....G..A........TGCA.....AC.A..C..G.. 2345

Hsa      GGAAGCTTATGTTCAATCAGTGAGAAGTAGAGAAGGCAAAGAATTTGCACCAGTTTATCCCATAATGGTTCAGCTGCTTCAAAAGGCTATGTCTGCTCTTCAG 2523
Ptr      .........C............................................................................................. 2523
Ppy      .........C............................................................................................. 2523
Mac      .........C.......................................................................G..................... 2523
Cja      .........C..........................A..............C................................A.................. 2523
Mmu      ...............G...........C..G..C..T..........................T....................................... 2487
Rno      .........................G.C..G..............C...........C.....TC................G.....C............... 2523
Ocu      ...............G..T............................................C...C.C...........G..A..C........C.A.... 2523
Eca      ........................................................................A...........A.................. 2523
Cfa      .........C........T..A.........................................C.................G..A..C.CA.....C..C... 2523
Bta      .....................A.....C.......................G................................A.................. 2508
Mdo      ......C................A........................G...........A...........A........G..A..A.....CA........ 2514
Tgu      ..........................CA.....G..A..G..G...........C........T..............G.....A..C.....A..C...... 2445
Gga      .........C.....G..G..C...GCAC.......A..G..G.....T..C..C.......................G..G..A..C.....G..C...... 2448
```

COG6

```
Hsa      ATGGCAGAGGGCAGCGGGGAAGTGGTCGCAGTGTCTGCGACCGGGGCTGCCAACGGCCTCAACAATGGGGCAGGCGGGACCTCGGCGACGACCTGCAACCCGCTGTCGCG 110
Ptr      .............................................................................................A................ 110
Ppy      .....C................................................................T......................A................ 110
Mac      .....C.......................................................................................AA............... 110
Cja      ..............T.....G..............G.....T........G..........................................AA............... 110
Mmu      .....T..C.C.........G....CT..G..CC.C..CT.......C.....T...T...GT...........T.C....C.C...CA.C..AA...T........... 110
Rno      .....T..CAC.........G....CG..G..CC.C..CT.......C.....T.......G.........T....C....C.A...CA.C..AA............... 110
Ocu      .....C....C...T..............G...C.G.................T.......G.........G.....CG.....T..CA.T..AA............... 110
Eca      .....C...............AGTCG.AGT.CCC.CATCCGG...CTGC.A.CG.C.TCAGCA.TG.....G..T..C...........A.G.A..........TGTC.. 110
Cfa      .....C..C...G...C........CG..G...C.CC..T.....................G...C............G..CT....CA....G................ 110
Bta      .....C.................A....T....C.C.....T....................T........C...............CA....A................ 110
Mdo      .....C..........CC.GG..A.CT..TT..C.G.T.T.G...C.GC...G.A.T..TGG.........TA....AG..G.AA.TCA..G.AA............T.. 110
Tgu      .....C..CAC.GCGCC.CCGCC.CC...C.CCC.C..CC.G...CTCC.G------------------------------G.T..TC.CGG.AC............C.. 80
Gga      .....C..CAC.GCTCCCCTGCC..CA..C.CCC.G..GG.----------------------------------------------C..GGGAT............C.. 65

Hsa      CAAGCTGCATAAGATCCTGGAGACGCGGCTGGACAACGACAAGGAGATGTTAGAAGCTCTCAAGGCACTTTCAACCTTTTTTGTTGAAAATAGTCTGCGGACTCGAAGAA 220
Ptr      .............................................................................................................. 220
Ppy      ...........................A.................................................................................. 220
Mac      .................................................................................................A.A.......... 220
Cja      .................................T..........................................................C................. 220
Mmu      T........C..............A.....A..A..T..............G..............C......G....................T............... 220
```

```
Rno    .........C...........A........T..A..T..............G..............C..C...G....................T...........C.G. 220
Ocu    ......A....................T.................A.....G......................................C...T............... 220
Eca    G.............CT..T..A.....C.......T...............G......................G...................T............... 220
Cfa    G......A...........................................G.................C....................C...T............... 220
Bta    G..A....G............C..C....................C.....G........T........G....................C...T............... 220
Mdo    G........C.....A........AA.......T..T............C............................C.....G........CT.A..A..CA...... 220
Tgu    ......CA.C.................C........T........A...C.G.....A........T..C..G...........G..G..C...T....C..C.....G. 190
Gga    .......A.C.................T.................A...C.G.....A........T..C..G..G........C.....C..C.....C..C.....G. 175

Hsa    ATTTACGTGGAGATATTGAACGTAAAAGTTTAGCCATCAATGAAGAATTTGTAAGCATTTTCAAGGAAGTGAAGGAGGAACTTGAAAGCATAAGCGAAGATGTTCAAGCA 330
Ptr    .............................................................................................................. 330
Ppy    .............................................................................................................. 330
Mac    ..............................................................................................T............... 330
Cja    ...............................................................................G..............T............... 330
Mmu    ..........T...........C.G...C..............G..G.......A...C.....A..C...........G...........C.AT........A..G..C 330
Rno    ..C....C..T.............G...C..............G....................A..T..A...........C........C.AT.....C..A...... 330
Ocu    ................A.....C.G......G.......................G...........C.....A...................AT............... 330
Eca    ......................C.G...................................................A................AT............... 330
Cfa    ......................C.G...........................C.......................A................AT............... 330
Bta    ................C.....C.G.....................G..........................A..A...............CAT............... 330
Mdo    ......................C.G..................G........G.....A...........A.................T....AT........A...... 330
Tgu    .CC.G........C........ACG...C..G.....A..............GCA...A......C.G..T..A...........G..T....AT........G...... 300
Gga    .C..G........C.....G...CGG..C.......................CCA...A......C....T..A.....G.....G..T....AT..............G 285

Hsa    ATGAGCAACTGTTGTCAAGATATGACAAGTCGCCTACAGGCAGCAAAGGAACAGACTCAAGATTTAATAGTAAAAACCACTAAGCTTCAATCTGAAAGCCAAAAATTAGA 440
Ptr    .............................................................................................................. 440
Ppy    .............................................................................................................. 440
Mac    ................................A...............................................A............................. 440
Cja    .......G...........................G.......................................................................... 440
Mmu    .......G.........G...............T.............A..........................G...........G...G.G....A.....G.C.... 440
Rno    .......G...C.....G..C........C...T........................................G...............G.G....A.....G.C.... 440
Ocu    .......G....................A..................A..........................................G.......T....G...G.. 440
Eca    ...............................................A..........................................G.......T....GG..... 440
Cfa    .......G.............................................A..A............A....................G.......T....G...... 440
Bta    .......G...................................................G..............................G.......T....GG..... 440
Mdo    .....T..............C..............GA............................G..T........T.....A......G.A.....T....GG..G.. 440
Tgu    .......G...C...G....C...T.C.....TT.GA................A.....G.....G..T...........A..A..C..GG.A....AT..G.G...... 410
Gga    .......G...C...G........T.C.....TT.GA..........A.....A..C..G..C..G..............A.....C...G.A....AT..G.G.C.... 395

Hsa    GATAAGAGCTCAAGTTGCAGATGCCTTCTTATCCAAGTTCCAACTGACTTCTGATGAAATGAGTCTTCTCCGAGGTACAAGAGAAGGACCCATTACTGAGGATTTTTTCA 550
Ptr    .............................................................................................................. 550
Ppy    .........A................................G................................................................... 550
Mac    .........A.....C......................................................................................G....... 550
Cja    A........A....................................................T............................................... 550
Mmu    ...T.....A..G....TG........T..GG..........G........G..............C.....C..........G...C...G..........C....... 550
Rno    A..T.....A.................T..............GT..................C...C................G...C...G..........C....... 550
Ocu    A..CC....A..G.....T...........G...........G..................G..........T.................TG..........C....... 550
Eca    A........A........T.......................................................................T................... 550
Cfa    A........A.....C.............................................G....C...........T...........T................... 550
Bta    A........A.....G..........................G...........C..........................G...........C................ 550
Mdo    A................................T..A.....G........A..................A....C..............T.....A............. 550
Tgu    A..G.A...A..............A...A..G.A..A.....G.....AC.G..........A...G..T.....C....A...T.AG..A...........C....... 520
Gga    A..G.A...A..............A...A..G.A..A.....G.....AC.A..........AC..C..T.....C..G.A...T.AG..A.....C.....C....... 505

Hsa    AGGCACTGGGAAGAGTAAAACAGATTCATAATGATGTCAAAGTTCTCTTGCGTACAAATCAACAAACGGCAGGTTTAGAAATTATGGAACAGATGGCCTTACTTCAAGAA 660
Ptr    .............................................................................................................. 660
Ppy    .............................................................................................................. 660
```

```
Mac     .......T...................................................................................................... 660
Cja     ...............................C.............................................................................. 660
Mmu     .....T..........G.................C.....G.....T.....A..C..C..G..G...........G..G....................G......... 660
Rno     ....TT..........G.....A...........C...........T...A.A..C..C..G..G...........G..G....................G......... 660
Ocu     .A...T.A..........................C............C.A.....C..C..G.....A.....G..........................G......... 660
Eca     .A...T....................................................C........A.......................A........G......... 660
Cfa     ....TT..........G....G..............................A.....C.....G...................................G......... 660
Bta     .....................................T....................C........A..G.................G..A........G......... 660
Mdo     .....T.A..............A..................A....T.....A.....C........T..G...C.......A........................... 660
Tgu     ....TT.....C..........A......G..........GA.....C.C..A........G....G......C..G....................T...........G 630
Gga     ....TT.............G..A......G..........GA.....C.C..G........G....G......C..G....................T.....G.....G 615

Hsa     ACGGCTTATGAAAGACTTTACCGATGGGCTCAAAGTGAATGCAGAACATTGACACAAGAATCATGTGACGTATCTCCAGTATTGACACAGGCAATGGAAGCCCTGCAGGA 770
Ptr     ....................................................................T......................................... 770
Ppy     ....................................................................T......................................... 770
Mac     .....................................................................A........................................ 770
Cja     ...T.C...................................T...G.............................G.................................. 770
Mmu     ..T....................G...........C..G......G..C....T.....G..C.....T..G...G.G..G..AT.C.....C................. 770
Rno     ..T....................G...........C..G......G..C....T..G..G..C.....T..G...G.G..G..A..T.....C................. 770
Ocu     ..A........G............................................T............A..........C..A..T....................... 770
Eca     ..A.................T.............A.......C..........................A.............A..TA...................... 770
Cfa     ..A...........G..G..T..G........................C....................A..........T..A..T....................... 770
Bta     ..AT....C..............G........................C....................A..........GC.A..T....................... 770
Mdo     ..TT.C......C........A......................G..T.....................A...........C.T..C..A..T................. 770
Tgu     ..AT........C........A.....A......A......T..G........G...............A..........TC.TG.T..A..C.........T.A..A.. 740
Gga     ..AT.......GC........A.....A......A...G.....G..T..............G......A.T........TC.CG.T..A..T.........T.A..A.. 725

Hsa     CAGACCTGTCTTATATAAATATACCTTAGATGAATTTGGAACAGCCAGAAGAAGTACAGTTGTTCGTGGATTTATTGATGCGCTCACAAGAGGGGGCCCCGGAGGTACAC 880
Ptr     .............................................................................................................. 880
Ppy     .............................................................................................................. 880
Mac     .............................................................................................................. 880
Cja     ..................G.............................G...........A..G.......................G...........T.......... 880
Mmu     ...G...........C..G.......................G...C..C....C..T...........G...........T..T........A..G..A.......... 880
Rno     ...G........G..C..G.......................G.....GC....C..T..G........G...........T..T........A..G..A.......... 880
Ocu     T.................G..C...........................................................C..T........A.....A...A...... 880
Eca     T.................G..............G...............................................T..T..............A.......... 880
Cfa     T.................G..........................T........C..T.......................T..T..............A.....C.... 880
Bta     ..................G..............G..........................G...........C........T..T..............A........C. 880
Mdo     ......A..A........G....................G..C..T.......................G...........T..T........T.....A.....G..T. 880
Tgu     T.........C.G..C.....C..G..G.................T.....G...G.T..G..C.....C.....A.....T..T..T.....A..T.TG........T. 850
Gga     T........TC.G..C..G..C..TC.G.....G..C........TC....G...G.T..A..C.....C.....A.....T.....C.....A..T.TG.....C..T. 835

Hsa     CTAGACCAATTGAAATGCATTCTCATGACCCTTTGAGGTATGTAGGAGATATGTTGGCTTGGCTCCATCAAGCTACTGCTTCTGAAAAGGAACACCTTGAAGCTCTCTTA 990
Ptr     .............................................................................................................. 990
Ppy     .............................................................................................................. 990
Mac     ...................C...........A...........G.................................................................. 990
Cja     .C..G..............C.......................G.....................................................C............ 990
Mmu     ....G..C...........C..C........GC..........G.....C...C....A....................C.....G........T..G............ 990
Rno     .......C...........C..C........GC..........G.....C...C....C....................C.....G........T..G............ 990
Ocu     .......C.....G.....C..C....................G.........C....C......................................G............ 990
Eca     .......T...........C..C....................................................................A.................. 990
Cfa     ....G..C...........C..C........G..A..A.....................................................A..T............... 990
Bta     .......C...........C..C..............A..C............................................G.....A.....C........TC.G 990
Mdo     ..C....T...................................G.....C............................................T.....G..AT.A... 990
Tgu     .CC.C..T...........C........T...C................C...C....C...T.G...........A..............A...T.A......A.G... 960
Gga     .CC.C..T...........C..C.....T...C................C...C....C.....G...........A..............A..T..A......A.A... 945
```

```
Hsa   AAGCATGTAACTACACAAGGTGTTGAAGAAAATATTCAAGAAGTTGTTGGGCATATCACTGAAGGTGTGTGCAGGCCTCTAAAGGTTCGAATTGAGCAAGTAATAGTTGC 1100
Ptr   .............................................................................................................. 1100
Ppy   ..................................................C........................................................... 1100
Mac   .....................A........................................................................................ 1100
Cja   ........C...G................G...G....G....................................................................... 1100
Mmu   ............G.T..G......A..........C........C..............C..G........TC.......................A.........C.... 1100
Rno   ............G....G......A..................................C..G........TC.......................A.........C.... 1100
Ocu   ..A..C..TC....C...............................................G...........A.....G..............A.........C.... 1100
Eca   ........G...........C.........................................G..G.............................A.............. 1100
Cfa   .............T.......A................G.................T.....G................................A.............. 1100
Bta   ............G.....................................C...........G...........A....................A.............. 1100
Mdo   ..AATG.....C.T............T.....C.........A.A....................A..T..T..............C........A.............. 1070
Tgu   ....T...G....TT........G....................A..C.....C.....A........C.....A.....G......A..........G..G..T..... 1070
Gga   ....T...G....TT........G....................G........C.....A..G.....T..................A.G........G..G..T..... 1055

Hsa   TGAACCTGGGGCAGTTTTATTATATAAAATTTCTAATCTCCTCAAATTTTATCACCATACAATCAGTGGTATTGTTGGAAATAGTGCAACTGCATTATTGACTACCATTG 1210
Ptr   .............................................................................................................. 1210
Ppy   .................................G.........................................................A..............G... 1210
Mac   .....................................................................C.....................A....G............. 1210
Cja   .....................G...............................C....C..........C.....................A..........C....... 1210
Mmu   ...............G..G.................C........G.....C.....C..C........C..C...............G..A.TC..C....A..A.... 1210
Rno   ...............G....................C........G.....C........C........C..................G..A.TC..C....A..A.... 1210
Ocu   ..........................................T........C.................C...........C.........A.................. 1210
Eca   ................C...................C.....T........C..T..............C.....................A....G........A.... 1210
Cfa   ..................G.................C..............C..T..............C.....................A.................. 1210
Bta   ....................................C..............C.....C...........C...A..........C......A....G............. 1210
Mdo   ...G................................C.....T.....C..........................G........C.....CA....G.....C....... 1210
Tgu   ...G..A..A.........C.G..C..G.........T.G.....G..C..C.......................A.....C......G.CA..C.G.....A..A.... 1180
Gga   ...G.....A.........C.G..C..G...........T.....G..C..C.......................G.....C......G.CA..C..C....A..A..A. 1165

Hsa   AAGAAATGCATTTGCTAAGCAAAAAAATATTCTTCAATAGCTTGAGTCTTCATGCAAGTAAATTAATGGACAAGGTTGAACTCCCACCACCTGATCTTGGACCAAGTTCT 1320
Ptr   .............................................................................................................. 1320
Ppy   ..........................................................................A................................... 1320
Mac   .............................................................................................................. 1320
Cja   .......................................A...................................................................... 1320
Mmu   .G........CC................C.......C............C.....G.A....C.G..............G..............C..C.....C..C... 1320
Rno   ..........C.........................G............C.....G.A....C.G.........A.C..G..............C..C.....C...... 1320
Ocu   .............A........G................................C...................................................... 1320
Eca   .............................................................................................................. 1320
Cfa   ............................C..................................................G.......................G..C... 1320
Bta   ................G....................C.....A...........C.....G................................................ 1320
Mdo   .................................................C............C.......T...................................C... 1320
Tgu   ..............T.G.....G...........T......C....................C................G........T...........G.....C... 1290
Gga   ..............T....T..G..G........T..C...C.............G......C.G.......................T........C..C.....C... 1275

Hsa   GCACTAAATCAGACACTCATGTTGCTGCGTGAAGTTTTAGCATCTCACGATTCTTCAGTTGTACCATTAGATGCTCGTCAAGCTGATTTTGTGCAGGTTTTATCATGTGT 1430
Ptr   .............................................................................................................. 1430
Ppy   .............................................................................................................. 1430
Mac   .................A..A..........................T.............................................................. 1430
Cja   ..G...................................G...................G........G.....A................................... 1430
Mmu   ..C..G.G......C....CT........G..TA..C.G..G..G..T...........CA.C..C..G........G..G.....C..............G..C....C 1430
Rno   ..C..G.G......C....CT........G..C...C.G..C..A..T...........G..C..C..G........G.....C..............G........ 1430
Ocu   ...T..................................G........T..............C..T.............................A........G..... 1430
Eca   ..CT....................T.............G........T...........C......C.G.....C................................... 1430
Cfa   ...T....................T...........C.G...................................C...........C.....A..A.............. 1430
Bta   ..CT..........G...G.................C....G.....T..............G..G........C.............................G..... 1430
Mdo   .......GC.........GCA..AT.............G........T..C...........G...C................A........A................. 1430
```

```
Tgu         ..T..............T.AC.....A..G..G...C.G........T..C..A..T...A.T...C.G.....C.....G.....C............C.G..T..... 1400
Gga         .................T.AT.....A..G..G...C.G...........C..A..T.....T...C.G.....C.....G.....C............C.G..T..... 1385

Hsa         CTTGGATCCTCTCCTACAGATGTGTACTGTATCAGCCAGCAATTTAGGCACAGCTGACATGGCCACTTTCATGGTCAATTCACTATATATGATGAAGACAACATTAGCTC 1540
Ptr         .............................................................................................................. 1540
Ppy         ....................................................................................G......................... 1540
Mac         ..........................................C................................................................... 1540
Cja         ..........................................C................................................................... 1540
Mmu         .C.............C...........A..G...................................G..........C......G..C..............CC.G.... 1540
Rno         .C.............C........C..A..G....................C..............G.................G..C...........G...C.G.... 1540
Ocu         ...............C.........................................T.......................T..G......................... 1540
Eca         ...............C.............................................................................................. 1540
Cfa         ...............G..A..............T.....................................................................C...... 1540
Bta         ...............C........................C....G...........T.........................................G.......... 1540
Mdo         T.........T.A..C.................T..T..T.......................T.............C...TT.G..................C....A. 1540
Tgu         GC.A.....AT.GT....A.........A.G..T..T..T.....G...........T.....A..C.A......A........T..C.......G...T..TC.G.... 1510
Gga         GC.A.....AT.GT..............A.G..T..T..T.....G.................A..C........A........T..............T..T..G.... 1495

Hsa         TATTTGAATTCACTGACAGACGTCTGGAAATGCTACAGTTTCAGATCGAAGCACATTTGGACACACTTATAAATGAGCAAGCCTCTTATGTTTTAACTAGGGTAGGCTTG 1650
Ptr         .............................................................................................................. 1650
Ppy         ..................................C........................................................................... 1650
Mac         ..............................................T............................................................... 1650
Cja         .............G.......................A..................................................C..............G...... 1650
Mmu         .G..C........A.....G..C..A........T...........T.....C...C.......T.....C.............................A..T...... 1650
Rno         .G...........A.....G..C...........T.................C...C.......T.....C.................C...........A..G...... 1650
Ocu         .......G.............................A........T..................................T.................A......... 1650
Eca         .G............................................T.............................A.....T.................AAC....... 1650
Cfa         .G.....G.................A........G...........T...................................T.....C..C........A.C....... 1650
Bta         .G................................G......C....T...................................T..............C..A.C....... 1650
Mdo         .C........T.......A...C..A......T....A........T..............T....................A........G.....C..A.C...TC.. 1650
Tgu         .C................A......A......T.G...........T.....T.....A..T.....C..............T..C..C..GC....G..A.CT..TC.. 1620
Gga         .C................A.............T.............T.....T.....A..T.....C........A.....T..C..C..CC.......A.CT..TC.. 1605

Hsa         AGTTACATCTATAACACTGTACAGCAACATAAACCTGAACAGGGCTCTTTAGCTAATATGCCCAACCTAGATTCTGTGACACTGAAGGCTGCAATGGTTCAGTTTGATCG 1760
Ptr         .............................................................................................................. 1760
Ppy         .............................................................................................................. 1760
Mac         ....G..........G............................................G................................................. 1760
Cja         ...A................G........C.....A.....C....................A............................................... 1760
Mmu         ..C........C......A............G......T.....T........C..............G...G.....G.G................C.........C.. 1760
Rno         ...........C.....GA.......G....GG.....T........C.....C.G......................G.G................C......C..C.. 1760
Ocu         .....T........................................G..........T.............C......G.............G...C............. 1760
Eca         .....T.......................................C...........T.......T............G..T............................ 1760
Cfa         ...................................A.....................T....................G............................... 1760
Bta         .....T..T..C....TG...............GT..........C...........T....................G............................... 1760
Mdo         .....T.............................A........TC.C....T...CT......G...G.....A..CT..........A.................... 1760
Tgu         ........T.....TG..C.G........C.....A..G.....TC..C.TT.A...T..T.A.GTA.G.........T.C.......T........C............ 1730
Gga         ........A......T....G........C.....A........TC..C.TT.A...T....A.GT..G.....CA..T.C.......TA.......C............ 1715

Hsa         TTATCTGTCAGCCCCAGACAACCTATTGATACCACAGCTGAACTTTCTTCTAAGTGCCACAGTGAAAGAGCAGATCGTAAAACAATCTACAGAATTAGTCTGCAGAGCCT 1870
Ptr         .............................................................................................................. 1870
Ppy         .............................................................................................................. 1870
Mac         ...........................A.................................................................................. 1870
Cja         ...C...........G...........A..G.............................................A.......G..C...................... 1870
Mmu         C..C..C............C....CC....G...........T.CC.....C..............G........TA....G..G........GC.C............. 1870
Rno         C..C..C...........TC....GC....G.............CC..C..C........C.....G........TA....G..G.....G..GC.C............. 1870
Ocu         ...C........T...........G..A..G..T.............................A............A.......G..C...................... 1870
Eca         ........................G..A..G..C........T.................................A....G..G......................... 1870
```

```
Cfa          ...CT.............T.G...GC.A..G.........................................A...A.......G......................... 1870
Bta          C...........T..T........G..A..G.............................................A.......G......................... 1870
Mdo          C.....T..T........T..TT.G..A..G...........T..C.................A.....A......A..........C...........T.......... 1870
Tgu          C........T..T.....T.GT..G..A..GT.C........T..C.....G........T..............AA.T.....G..A......C.C...........T. 1840
Gga          C.....A..T..T.....T.GT..G.....GT.C........T..C.....C........T..............AA..........G......C.G...........T. 1825

Hsa          ATGGTGAAGTGTATGCAGCCGTGATGAATCCAATCAATGAATACAAAGATCCAGAGAACATTCTTCACCGATCGCCGCAGCAAGTGCAGACGCTTCTTTCC 1971
Ptr          ..................................................................................................... 1971
Ppy          ...................T................................................................................. 1971
Mac          ...................T............G...........................................A........................ 1971
Cja          ...................T................................................A......................A......... 1971
Mmu          .C.CG......C....................GA...C.C.................G...C.....TA.G..A..TG....G...A....A..G..C..T 1971
Rno          .C..G..G...C....................G....C.CG........C..G....G...C......A.G..A..CG....G...A.......G..C..T 1971
Ocu          ...............................C.........................G......A...........A..............A......... 1971
Eca          ...A...............T............GG.......................G.G....C..........................A.....C... 1971
Cfa          ...................T............G.G......................G...C..A...........T..............A..C..C... 1971
Bta          ....G..............T...........GG.......G...........T.G..G......A...........A..............A.....C... 1971
Mdo          ..A.....C.....A....T.............C..................T....C......G........T..T........C.....A.....C... 1971
Tgu          .CA....GT.A........T......G....TTCA........T...........A.C...C..A...A....T..T..T.....T...G.A..C.....A 1941
Gga          .CA....GC..........G......G....TTCA...A....T..G........A.CG..A..A...A....T..T..C.....T...G.A.....C..A 1926
```

COG7

```
Hsa          ATGGACTTCTCCAAGTTCCTGGCAGACGACTTCGACGTGAAGGAGTGGATCAATGCGGCCTTCAGGGCCGGCTCCAAGGAGGCGGCGTCCGGGAAGGCGGATGGCCACGC 110
Ptr          .............................................................................................................. 110
Ppy          .........................................................................................T.................... 110
Mac          .............................................................................................................. 110
Cja          .......................C........T.............................T........................A.............C........ 110
Mmu          .......................T....................C.....T..............A......C....A..C.G....G.G...........C..T..... 110
Rno          .......................C..T.................C....................A......C.T..A..C.G....G.G...........C........ 110
Ocu          .......................C........................................A...T...C..........C...G.............C........ 110
Eca          ..................T....C..A..........................C..............T...C..............G.............C........ 110
Cfa          .......................G..A......T..A................C.........T..........A..T.........G.G...........C........ 110
Bta          .......................G..A..........................C..............T...C..............G.............CA....... 110
Mdo          .....T..............A..G..T........G...................G........A...G.T.C.......A..CC.T---.......T...C..G..T.. 107
Tgu          ...........GCT...T...T.G.....G.....A.......GC...G.G..C.TT..T...CAA....TGCATT.T...T.T..AA.A.T..G.CACT.CTCT.C.A. 110
Gga          .....TA.....CGC...A....C..T..T.....G......AGC...G.G..C.................GCAGC....C---...C.....C.......C.C...... 107

Hsa          AGCCACCCTGGTGATGAAGCTGCAGCTGTTCATCCAAGAGGTGAACCACGCCGTGGAGGAAACAAGTCACCAAGCTCTCCAGAACATGCCCAAAGTGCTCCGTGATGTTG 220
Ptr          .............................................................................................................. 220
Ppy          ..........................................................---------------------------------------------------- 168
Mac          .............................................................................................................. 220
Cja          ...........................................................................A................................C. 220
Mmu          ............A.......................G...............................T...G..C................................G. 220
Rno          .........................T..........G...............................T......C..T.............................G. 220
Ocu          G.....G.............................G......................................C..T.............G............C..C. 220
Eca          G...................................G........A..........................G..C..T..............G..............G. 220
Cfa          G.....G.............................G...................................G.....T............................... 220
Bta          ........................................................................G..C................................C. 220
Mdo          C......T...................T........G..A..C...A....T...........C........G..C........T...........T..T..A.....G. 217
Tgu          CT..TGG.ACA.....T.C.....AT.AA.A..TA..A.CA.CTTAACAAG...T..A........C..T..G..A........T.......G...C.....G..A..G. 220
Gga          CT.......................G..........G.....C...A...TG.....A...G.C..C..G..G..C..G..C.G........GG.....G..C..G..G. 217

Hsa          AAGCCCTAAAACAGGAGGCATCTTTCCTGAAAGAACAGATGATTCTTGTCAAGGAGGACATTAAAAAATTTGAACAGGACACATCTCAATCCATGCAGGTGTTGGTAGAA 330
Ptr          ...............................................................................................A.............. 330
Ppy          -------------------------------------------------------------------------------------------------------------- 168
```

```
Mac   ...............................G...............................................T.............................. 330
Cja   ....T..........................................................................T..........................C... 330
Mmu   .......G..G........G..............G..A..............A..............G.....G........G..A..G............C.......G 330
Rno   .......G..G........G.................A..............A....................G.....T..T..A..G...........AC.......G 330
Ocu   .G.....T...........G.................A.........................GG........G...........A..G.................G... 330
Eca   .......T...........G.....T...........A......................................A.....G.....G..G........AC........ 330
Cfa   .......T...........G........A........A.....C...................................T...........G........AC....G... 330
Bta   .......T...........G.....T...........A.............................G....................G..G.................. 330
Mdo   .......T.......................G.....A.................A..T........................G....G..A........AC....G..G 327
Tgu   ...T.T.G............A.A........G..G........C..C..T..A.....T..A..G..G.....GG.A......G....G...........CC....G..G 330
Gga   .G.....GCGG........GG.C......CGG..G......G.G..G..............C.GG.GG.....GG.......GG.G..G..G........CC....G..G 327

Hsa   ATTGACCAAGTGAAGTCCAGAATGCAACTTGCTGCCGAATCTCTTCAGGAAGCAGATAAGTGGAGCACGTTGAGCGCCGATATTGAGGAGACATTTAAGACTCAGGACAT 440
Ptr   .............................................................................................................. 440
Ppy   ------------------------------------------------------------------------------------------------------........ 176
Mac   ........G....................................................................T..............G................. 440
Cja   ........G.................................................................T..T................................ 440
Mmu   ........G...........G.....G........G.................T..C............C....T..T..............C..C..A.....A..... 440
Rno   ........G...........G.....G........A.................T..C............C....T..T..............C..C..A..G..A..... 440
Ocu   ..C..T..G..C..............G..G..C.....G.....G...........C...........CC..........C..C........C..C.............. 440
Eca   ..C.....G..C..............G..G..C..A.....G.....A........C.................T..T.........................CA..... 440
Cfa   ........G..A..............G.....C.....G........A........C...........AC.A..T........C....................A..... 440
Bta   ..C.....G..C..............G.....C..A...........A.....T..C..A........A.....T..T.....C........T...........A..... 440
Mdo   ........G.....A....................A...........A....................C.....T..A........A..A...........A..A..TG. 437
Tgu   .......GG...........G.....GT.G........G..A........G.....C..A........AC....T..A........A.....TC.C..A..A......G. 440
Gga   ..C....G......A..T........GT.G.....T..G.................C..A....CT..A..A.....A.....C..A.....CC....A..A.....TG. 437

Hsa   AGCTGTGATTTCTGCCAAGCTAACAGGTATGCAGAACAGCTTAATGATGCTTGTTGATACACCAGACTACTCAGAAAAGTGTGTGCACTTGGAGGCACTGAAGAACAGGC 550
Ptr   .............................................................................................................. 550
Ppy   .............................................................................................................. 286
Mac   .............................................................................................................. 550
Cja   ....................................T.....G.............................T.....A.............................A. 550
Mmu   ...............A.....G..C..C..............G...........G.....T..G........T...............C.......T...........A. 550
Rno   .........C.....A.....G..T..........G......G...........G.....C....................C......C.......T...........A. 550
Ocu   .....................C..G..C..............GGC.........G..C..C...........C.....A.................G............. 550
Eca   ...........................................................................G.................................. 550
Cfa   .....................G........................................................A..................T............ 550
Bta   ............A........C..................C..........C..........................A.....A...........GT.......C... 550
Mdo   GA.............A..AT....CA.............T..G.C.........A....................T..A.........C.T.................A. 547
Tgu   GT.CC....C..A........G..CA.C.......G....C.GGCC.....G..G..C..G.....T........G............C.................C.C. 550
Gga   .T...CA...G.A............A.........G......GGCT........G.....G..G..T.....T..G..........TC.T.....T.........C.C. 547

Hsa   TGGAGGCCCTAGCCAGTCCACAGATTGTAGCGGCATTCACCTCTCAGGCTGTAGATCAGTCCAAAGTGTTTGTGAAGGTGTTTACTGAAATTGACCGGATGCCCCAGCTC 660
Ptr   ...................G...........................T.............................................................. 660
Ppy   ...................G..............G................................A.......................................... 396
Mac   ...................G...........A.............................................................................. 660
Cja   ...................G........C...........T..................................................................... 660
Mmu   ..........G.....C..C.....C.....AT.G............T....G........T.............................C.................. 660
Rno   ..........G.....C..C...........A..G............T.C..G........T.....A..C..........C.........C.................. 660
Ocu   ..........G........C.....C..G..C..G......A.G...T....G.............C............T.C...G...G.G.................. 660
Eca   .A......T.G.....C..C...........A.TG....G...C...T..A...............C.........AA.T.............................T 660
Cfa   ....A.....G...TC...C........G.....G............T.......G......G.......C........................A...........T.G 660
Bta   ........T.G.....C..C.....C.....A........A......T..A..............A.............T....G........................T 660
Mdo   .T..A...A.G.....C........A..G..T.......AT......T.C........AG.......A........A..T.....A........T..A.....T..A..T 657
Tgu   ........A.G.....C..T........T..T..T....A...C...T.C.........G.A...A.......T..A..C.C............T.............T 660
Gga   ........A.G.....C...........G..T..T..T.A.......T...........G.A...A.............C.C............T..A.....T.....T 657
```

```
Hsa     CTGGCCTACTACTACAAGTGTCACAAGGTGCAGCTTTTAGCAGCCTGGCAAGAGCTGTGTCAAAGTGACCTATCCCTGGACCGGCAGCTTACCGGACTCTATGATGCCTT 770
Ptr     ........................................................................................................C..... 770
Ppy     ........................................................................C...................T...........C..... 506
Mac     ........................................................................C.................G.T...........C..... 770
Cja     ..........................................A.............................C...................T...........C..... 770
Mmu     ..T..A.......................A......C.....A...................G........TC.A..............C..T..C............C. 770
Rno     ..T..A....................................A...................G..G.....TC.A..............C..T..C.....CC....... 770
Ocu     ..C..G.............................C...A..........G........C..G........CC................C..............C..... 770
Eca     ..T..A..T....................C.C..............................G.....G..CC................C..T..G.....C..C..... 770
Cfa     ..C..............A...................................A........G.....T..CC.T..............C..T...T............. 770
Bta     ..T..A..............C.............................G........C..G.CC.....GC................C..T........C..C..... 770
Mdo     ..T........T.....A..C..T....CA........G...T.......G..T..T.....G.........A.TT.A........A..A..T.A.........CA..C. 767
Tgu     ..T.....T..........................GG.G..C.TG.....G.....T..C..G........GAG.G..ACG........G....AG..G..C..CA..C. 770
Gga     ..T..T..T..T..T...........A........GG.G....TG.....G..T..T..C..G......T..AG.T..A.T..C.....G..T.A...G.....CA.AC. 767

Hsa     GCTTGGTGCTTGGCACACACAAATCCAGTGGGCTACACAGGTTTTCCAGAAGCCCCACGAGGTGGTAATGGTGCTGCTGATTCAGACCCTGGGGGCCCTCATGCCCTCGC 880
Ptr     .............................................................................................................. 880
Ppy     .........................................................T.................................................... 616
Mac     ......G....................................................................................................... 880
Cja     ......C........................................G.........T........G.C................................C......A. 880
Mmu     ......C..G.............C.........C............A....C..............G.C............................T.GG.......C. 880
Rno     ......G..A........G....C.........C............A....C...T.T........G.CA...........................T.GG.......C. 880
Ocu     .........C......T................C..G.........A....C..............G.C............C...............T.GG.......C. 880
Eca     ...C..............G....G...A........G.........A....C..............G.CC...........................T.GG.......T. 880
Cfa     ...C..G........T..G..G...........A..G........TA....C..............G.CT.................T.........T.GC.....A.T. 880
Bta     ...............TG.................T.G.....C...A....C........C..C....CT..T..........................GG.......T. 880
Mdo     .....C...C........C...C........T...TG.....C...A....C................CT.....T...........GT..AAT..TT.GG.A.....CT 877
Tgu     C..G..GA.C......G.C..GC.G........AG...........A....C...T.T..AA.C..G.CT......T.......A........A..T..GG....T..CA 880
Gga     C.....GA.C......T.G..GC.T........A............A....A........A..T..G.CT...T..........A..T..A..A..AT.GG....A..CA 877

Hsa     TGCCCTCCTGCCTCAGCAACGGCGTGGAGAGGGCAGGGCCCGAGCAGGAGCTCACCAGGCTGCTGGAGTTCTACGACGCCACCGCCCACTTCGCCAAGGGCTTGGAGATG 990
Ptr     .............................................................................A................................ 990
Ppy     ............................................................................T................................. 726
Mac     ......................T....................................................................................... 990
Cja     ..................G...T.................G.................A..................A....T........T.................. 990
Mmu     .....ATG........TGCA..T........A..C..A..G....T............A...........T......A.............T........GC....A... 990
Rno     ....TATG........TG.G.CT........A..G..A..A....T............A..A........T..T...A.T..T........T........GC....A... 990
Ocu     .....GTG.........TCT..............C..........T...........AA..................A....G........T.......CGC........ 990
Eca     .....AT..........TC...T......C....G.CA..T....T...........AA.................T.....T........T........G......... 990
Cfa     .A..TGA..........TC.....................T....T.......CT..A......A........T.................T........G......... 990
Bta     .....GTG........TTC..........C.......A..T....T.......GT..A..................T.......................G......... 990
Mdo     ....AGT......GGA..GT..A..A.....A........T..CATAAG......A.A...A......C....T...A....T........T.....A...C........ 987
Tgu     .C...GTG..T.......CT.C.A....C...A....C.AG..CACCA....G...CA..........C..C....T.......TG..............GC.....G.A 990
Gga     .C..TGT...........CT.C.A.......AA.G..C.AG..AACCA....G.G..A..........C....T..T.......T......T..A..A..GC....A... 987

Hsa     GCACTGCTCCCCCACCTACATGAACACAATCTGGTAAAAGTCACGGAGCTGGTGGATGCTGTGTATGATCCATACAAACCCTACCAGCTGAAGTATGGCGACATGGAAGA 1100
Ptr     .............................................................................................................. 1100
Ppy     ...................................................................................................A.......... 836
Mac     ...........................................................C.......................................A.......... 1100
Cja     ..G...............T.......................................T...A........G..........................T........... 1100
Mmu     ..G...........T..C..G..C.....C.....G.....TGT.......................G...C..T.......T............C..T........G.. 1100
Rno     ..G...........T..C..G..C.....C.....G.....TGTT.................A....G...G..T....................C..T..TC....G.. 1100
Ocu     ..T.............C......G.....CA....G........C.........C.G..A.......G...C...................................... 1100
Eca     ........T.......C......C.....CT....G.......T...A...................G...G...................................... 1100
Cfa     ....A...........C......GT....C.....G........A..A........G..........G..............................T...C....... 1100
Bta     ..C............GC.T....G..A..C.....G.......T...A...................G...G.....G.....................A........... 1100
Mdo     ..G..T...T.A..TTCC.GA........CT....G..G....T...A........A.......T..GC...........A......T.AC.A.....A..AC....... 1097
```

```
Tgu         ..CA....G..AA....CA.G..G..G..C.....G.....G.T......C.....A.TG.......C...C.....G.....T..A..CG.......A...C....... 1100
Gga         ..CA....G..AA.T..GA.G..G..G..CT..........G.T...A.....A..A..G......C..........G....TT..A..A........A...C.C..... 1097

Hsa         GAGCAACCTCCTCATCCAGATGAGTGCTGTGCCTCTGGAGCATGGGGAAGTGATTGACTGTGTGCAGGAGCTGAGCCACTCCGTGAACAAGCTGTTTGGTCTGGCGTCTG 1210
Ptr         .............................................................................................................. 1210
Ppy         .........A...........A..C..C......T..............................................T............................ 946
Mac         ........................C.........T..............................................T.......................A.... 1210
Cja         .....................C..C..CA.....T........................................................................... 1210
Mmu         ..A......T.....A.....C..C..............A..C.................C..T.................T...C.......C...CT......A.... 1210
Rno         .........T.....A..A..C..C..G...........A..C.................C..T..............A..T...C.......C...CT......A.... 1210
Ocu         A.A.C..............C.C..C.................C........C.............................G...........C..C..G........C. 1210
Eca         A.AA.................C..C..C......T.......C..A..G..C.........................................C...CT........... 1210
Cfa         ..AG...G..........A..C..C..G......T.......C........C........C....................T.......................T.... 1210
Bta         ..AGT....T.........T.C..C.AG...........A..C........C.............................T...........C.....C...T.T.... 1210
Mdo         T..TC.T..T.....T.....C........C...T....A...T.......T..C........C..A..............A........A.........T....C..A. 1207
Tgu         AGAG..T..............C.....A.....CT.......CT....G........T.....C.....A....A......A..C.....A..C..CACG......G... 1210
Gga         AGAA..T..G.....T.....C.....A......T....A...T.......A...........C.....A....AT..T..A..C.G...A..C..CAT......T.... 1207

Hsa         CAGCCGTTGACAGATGCGTCAGATTCACCAATGGCCTGGGGACCTGCGGCCTGTTGTCAGCCCTGAAATCCCTCTTTGCCAAGTATGTGTCTGATTTCACCAGCACTCTC 1320
Ptr         .............................................................................................................. 1320
Ppy         .....A...............................................C............................A.GG..CA.A------------------ 1038
Mac         .................A...................................C........................................................ 1320
Cja         .....A...........A......................C.....T..T...C................T......A................................ 1320
Mmu         ....T...........T.C..A........................T..A...C..A...................................C.C.......A.G..... 1320
Rno         ....T...........T.C..A...............T........T..A...C..A.....T....G.........A..............C.C.......ATG..... 1320
Ocu         ....T...........T.C...G....................G.........C..A.....T....G.................C............GG...T..G... 1320
Eca         .................A...................................C..A.....T..........T...A.........................T.....G 1320
Cfa         .................A............................T...T..C..G....AT........................................T...... 1320
Bta         .................A............GC..............T......C..A..........G..T.....C.................C.....T..T.....T 1320
Mdo         G...T..G..T......A...A......AG....A........T..T..G..TC................A..T..CA.A........A..C..........AT..C... 1317
Tgu         GG...A.C.....C..T....AGC....GG...........GTG..T..G...C..AAG........GG.T.....CT........ACA.....C.....A.A...A..G 1320
Gga         G....A.C....AC...A.T.A.C....TG....A.....AGTA..T..G..CC.TAAG.........G.T..A.....A......ACA.....C..T..A.AT..A..G 1317

Hsa         CAGTCCATACGAAAGAAGTGCAAACTGGACCACATTCCTCCCAACTCCCTCTTCCAGGAAGATTGGACGGCTTTTCAGAACTCCATTAGGATAATAGCCACCTGTGGAGA 1430
Ptr         .........................................T.................................................................G.. 1430
Ppy         ---------------------------------------------------------------------------------------....................... 1061
Mac         ......G.......................G...........G................................................................... 1430
Cja         .G............................G............................................................................... 1430
Mmu         ........T..............G.....TG...................G..T........C.....C...........T..GG.C.....C................. 1430
Rno         .....G..C..............G.....TG.T.................G........G........C..............TG.C.....C................. 1430
Ocu         .....A.................G.....TG........G......T......T........C...........C........TG......................... 1430
Eca         ..C..A.................G.....TG.......G..............T..A..................................................... 1430
Cfa         .....A.................G.....TG.T..............G..T..T..A...........A.................C....................C.. 1430
Bta         ..C..A.............A..G......TG.T.......T......T..T..T..A.......................T............................. 1430
Mdo         ..A..A..T.................A..TG.T......T...G......T..T..A...........A.....C........TG.AC...................... 1427
Tgu         .....T..............T........TG.TG.C.TGG.AG.TC....G........G..C.....A..A..C..A.....TG.CC.......TT.A.TT.....T.. 1430
Gga         .....G.......T...A..T........TG.T.....AT.AG.......T........G..C.....T..A..C..A.....TG..C.A.....TA.T..T.....T.. 1427

Hsa         GCTTTTGCGGCATTGTGGGGACTTCGAGCAGCAGCTAGCCAACAGGATTTTGTCCACAGCTGGGAAGTATCTATCTGATTCCTGCAGCCCCCGGAGCCTGGCTGGTTTTC 1540
Ptr         .............................................................................................................. 1540
Ppy         ........................................................................G...............................C..... 1171
Mac         ..............................A..............A---------------------------------------------------------------- 1476
Cja         ...C.............................................C..........C................................................. 1540
Mmu         ...C.....C..G...........T...........G..A........CC..........G...........G.....C....A......T...........A....... 1540
Rno         ...C.....C..G...........T...........G..A........CC..........A.........T.G.....C....A......T...........A....... 1540
Ocu         ...C........G...........T..............A.........C....G..T..............C.....C....A......T...........C..C.... 1540
Eca         ...C...A....A..........................A................................G.....C....A......T....A....A.C....... 1540
```

```
Cfa     ...C..A.....A.............................A.......................C..A.....C.....C....A...T..T..A................ 1540
Bta     ...C..AA....A.......................G..A................................C.....C....T......T.........A.C....... 1540
Mdo     ........................T..A...........A................................TA.G..C..T.A......T..T..T..CA....C.... 1537
Tgu     A..CC.T.AT..G.....A.................G.................A..T.....A........C..A..C....A......TT.C..TT.TT....C.... 1540
Gga     ...CC.T..T..G.....A.....T........AT.G.................A..T..............C..G..C....A.......T.T...T.TT....C.... 1537

Hsa     AGGAGAGCATCTTGACAGACAAGAAGAACTCTGCCAAGAACCCATGGCAAGAATATAATTACCTCCAGAAAGATAACCCTGCTGAATATGCCAGTTTAATGGAAATACTT 1650
Ptr     .............................................................................................................. 1650
Ppy     ...........................G.................................................................................A 1281
Mac     --------------------------------------........................................................................ 1548
Cja     ...........C...............G...............C...........................................................G...... 1650
Mmu     ....C......................G.C...................G............................................................ 1650
Rno     ....C......................G.C...................G...........................................A................ 1650
Ocu     ...........C..G.G..........G...............C....................G........C..............C.....C..G.....G...... 1650
Eca     ....T......................G...............G.................................................................. 1650
Cfa     ....C..T...................G.................................T.................G..C........................... 1650
Bta     .A..T......................G...............................................G......C.......G................... 1650
Mdo     ....T....G.C....G..G.......G..TG.TT..............G...........T..T.....G..G.GT..A.TA...............T.....G.T... 1647
Tgu     ....C.C..GT.CTG....A.......G...CATA..A..T..C.....G.....C.....T...TT...G..G..T..AT.C..G........CC.T.......C.... 1650
Gga     ....T.C..GTGCT.....A.......G.AA..TG.....T..C.....G.....C........TTT...G..G..T..GT....G........CC.T.......C...G 1647

Hsa     TATACCCTTAAGGAAAAAGGGTCAAGCAACCACAACCTGCTGGCTGCACCTCGAGCAGCGCTGACTCGGCTTAACCAGCAGGCCCACCAGCTGGCTTTCGATTCCGTGTT 1760
Ptr     .............................................................................................................. 1760
Ppy     ........C..................................................A..A............................................... 1391
Mac     ........C..................................................A..........................T....................... 1658
Cja     ........C.......................................T..........C......................................T.....T..... 1760
Mmu     ........C.....G..G..C..C..T...........A...T....GT.A..CA....C.....C.....G.............................C..G..C.. 1760
Rno     ........C.....G..G.....C..T.....T.........T...TGT....CA....C.....C.....G.............................C..A..C.. 1760
Ocu     .....T..C..............C.....T..............C..C.....CA....C.....C.....C...........G.......................T.. 1760
Eca     ..C.....C...........A.....T...............T.....T....G..G..C........T.............................T.....G..T.. 1760
Cfa     ..C....................T..T...............T....GT....CAT...................................................T.. 1760
Bta     ........C..............G..T......C........T.C..GT.G...T.G..T......A................G....................G..C.. 1760
Mdo     .....T..C...........C.....T..T.....T.....AT..A.CT.....ATG..T..A.....C.....T...............T....C..T.....T..T.. 1757
Tgu     .TC.....G.....G..G..CA.C..TC.G....G.......T.GT.GT.G.....G......G.G..C..G.......T.T.............C.....C........ 1760
Gga     ..C.....G.....G.....TA.G..T.........T.....T..T..T.....T.T..A..A.GC..C..C.......T...A.....CT.......T.....T..... 1757

Hsa     CCTGCGCATCAAACAACAGCTGTTGCTTATTTCGAAGATGGACAGCTGGAATACGGCTGGCATCGGAGAAACCCTCACAGATGAACTGCCCGCCTTTAGTCTCACCCCTC 1870
Ptr     .................................C........................................................T................... 1870
Ppy     ...........................C.....C.................C.T..............................C.....T................... 1501
Mac     .....................C.....C.....C............C....C..........................G.....C.....T................... 1768
Cja     ...........................C.....C.................C..A.......................G.....C.......................C. 1870
Mmu     .T.............G......C....CG....C.G......T........C..A..C.....T..T........G.....C..T.....T.....C..C.....A.... 1870
Rno     ...............G......C....CG.C..C.G......T........C..A........T..C........G.....C..T.....T.....C..C.....A.... 1870
Ocu     ...............G......C....C...C.C.................C..A.......................G..C..C...........C..C.....G.... 1870
Eca     ...............G.....CC.T..C.....C.................C..A..C.....T.....G.....G..C..C..C......A.G..C............. 1870
Cfa     ...............G......C.T..CG....C.................C..A..A.....T..C..T.....G..C..C..C.....TA....C........T..C. 1870
Bta     ...............G......C.C..C...C.C........T........C..T..C.....T...........G..C.....C......A....C...........G. 1870
Mdo     ......T..............T..AT.A.....C........G..T.....C..A.....A..T..G..G..TT.G........T..T...A.A.....C...T.T.... 1867
Tgu     ...........GG..G......C....GC..C.CCGC.....GG.A....G.T.T.G......T..T..G.....G........C.....AAA...C..C..G....... 1870
Gga     T..T........G........C..A...G....A........G..T....CCT.T.G...TG.T..T..G.....G.....G..C.....AAA...C..C..G..T.... 1867

Hsa     TCGAGTACATCAGCAACATCGGGCAGTACATCATGTCCCTCCCCCTGAATCTTGAGCCATTTGTGACTCAGGAGGACTCTGCCTTAGAGTTGGCATTGCACGCTGGAAAG 1980
Ptr     .............................................................................................................. 1980
Ppy     ................T............................................................................................. 1611
Mac     .............................................................................................................. 1878
Cja     .......T.............................G.....T.............................A...............C..........T......... 1980
Mmu     ...................T....................G.............................A...........A...........C.....T.....G... 1980
```

```
Rno   .T.................T....................G.............................A...........A...........C..A..T.....G... 1980
Ocu   .....................................T..G........C........G..C.....C.................G...C....CC.......C...... 1980
Eca   .............TC.T...........T.......................C.....G........G.....................C.A........T..C.....A 1980
Cfa   .G..............T..T........T.............................T..........................G...C....TC..........G... 1980
Bta   ............................T....................C..G........C.................A.........C....C.....T..C..G... 1980
Mdo   .T---..T..T........T........T..............T..A.....G...........T........A..T.....T..G..A..A.....A..T......... 1974
Tgu   .G..A..T........T..T...........T.....T..T..T...C.C........G.....C.....A.....T.....T..G..AC.......A..T........A 1980
Gga   .G..A..T........T..T.....A..T..T.....G..T..T..CC.C.....A........C..G........T.....T..G..AC.......A..T........A 1977

Hsa   CTGCCATTTCCTCCTGAGCAGGGGGATGAATTGCCCGAGCTGGACAACATGGCTGACAACTGGCTGGGCTCGATCGCCAGAGCCACAATGCAGACCTACTGTGATGCGAT 2090
Ptr   .............................................................................................................. 2090
Ppy   .............................................................................................................. 1721
Mac   ......................................................................................G..............C........ 1988
Cja   ..............C........T..GCGGGGA..GTG.G.....TTG...----------------------------------------------------------- 2031
Mmu   ........C...........A.........C.T......T...................T...........C..T...........T.................C.T... 2090
Rno   ........C...........A.....A...C.T..T...T...............................T..A..T........C...........T.......G... 2090
Ocu   ..T..G.....C..C.....A..C.....CC......................C.................C........G.....C...G.............C..C.. 2090
Eca   .....G........G.....A.................................................................G.................C.T... 2090
Cfa   .....C..............A.....C............T...............................C..T..T..............A.............T... 2090
Bta   .....G..............A.....C..GC......................C.................C..............C.................C..C.. 2090
Mdo   ....................A........G.....T........T............T.....T....T..A..T..T........C........A..T.....C.T... 2084
Tgu   T......AC..C..A.....A..A..G..GC....T............G........T.............C........G...---........G.....C..G.TC.. 2087
Gga   T....T.A...C.....A..A..A......C.A........................T.....T.......C..T........T..G..............C..A.TC.. 2087

Hsa   CCTACAGATCCCTGAGCTGAGCCCACACTCTGCCAAGCAGCTGGCCACTGACATCGACTATCTGATCAACGTGATGGATGCCCTGGGCCTGCAGCCGTCCCGCACCCTCC 2200
Ptr   ............C........................................................T........................................ 2200
Ppy   ...G........C.....T.....G.............................................................................T....... 1831
Mac   ...G........C...........G.......................C.............................C............................... 2098
Cja   -------------------------------------------------------------------------------------------------------------- 2031
Mmu   ...C........G...G...C...G.....CA.......A........C.............................C..A..............A..A..A....... 2200
Rno   ...C........A.C.G...C...G..T..CA.............T..A....................T........C..A..............A..A.......... 2200
Ocu   ...G........G.......C...G.....CA................C.....G.....C.................C.................C...........G. 2200
Eca   ...C........C.......CG........CA......................T.....C.................C.................C............. 2200
Cfa   ...G........C.......C...G.....CA................C....................T..................T.......A........A.... 2200
Bta   ...G.....T........C.C...G.....CA................C...........C.................C............................... 2200
Mdo   ...T.....T..........C.........CA.............T..A..T..T.....C.....A..T...........AT.......T.....C.....G..T.... 2194
Tgu   ...G........GC....C.CA........CA................G.....A..T.....C..A.................A...........C..A..G..G..G. 2197
Gga   ...G.....A..A.....C.CAGTG..T..AA.A........TT....A.....T..T.....A..A..T..............T.....T.....A..AA.A..A..G. 2197

Hsa   AGCACATCGTGACGCTACTGAAGACCAGGCCTGAGGACTATAGACAGGTCAGCAAAGGCCTGCCCCGTCGCCTGGCCACCACCGTGGCCACCATGCGGAGTGTGAATTAC 2310
Ptr   ................C............................................................................................. 2310
Ppy   .........C......C........................................................................G.................... 1941
Mac   .............A..C.................................................................T........................... 2208
Cja   -------------------------------------------------------------------------------------------------------------- 2031
Mmu   ..A....T.C.G....CT.....G...A.........A..........................A..C.....T..AG....G.............TG...........T 2310
Rno   ..A....T.C...A..CT.....G...A...C.....G........A..............T..A..C.....C..AG....A.............TG...........T 2310
Ocu   ..A.............G..C....G..A...C........C..G....C......G...........C.....C...G....T......G........G.C....CC... 2310
Eca   ..A.............C......G...AA..G.....T.....G.......................C.....C..TG....G......G..........C...G.C... 2310
Cfa   ..A.............CT.........AA........T..C..G.............................T........G.....TG.........A....G..... 2310
Bta   ..A.........T...CT.....G...A...C........C................................C.....A..G.....................G.C... 2310
Mdo   ..A....G.....T...T....A....AA..A.....T...........AGC......T..C........G..A....G..AA......T.....A...A.......... 2304
Tgu   ..A....T.....T..G......G...A...A.....G.T.C.C....CTGC...GA..........C..GA....AG.TG..A.T..GG.....A..G.CC..G.GGGT 2307
Gga   .AA.T..T..A..T..GT.....G.A.A...A........C..G..A.CTGCA...A.TG....T..CA.GA...T..G..G.A.T..TG.A...A..G.CC..G.G.GT 2307

Hsa   2310
Ptr   2310
Ppy   1941
```

Mac	2208
Cja	2031
Mmu	2310
Rno	2310
Ocu	2310
Eca	2310
Cfa	2310
Bta	2310
Mdo	2304
Tgu	2307
Gga	2307

COG8

Hsa	ATGGCGACCGCGGCGACTATCCCATCGGTAGCCACGGCCACAGCAGCGGCTCTCGGCGAGGTGGAGGATGAAGGGCTCCTGGCGTCGCTGTTCCGGGACCGCTTCCCCGA	110
Ptr	......................................T..G..	110
Ppy	.CC...G.GA.TATCC.ATCGT.GG.CACG...........G..	110
Mac	--	1
Cja	......G.GA......T..G.T.....TC....T.......G..G...........T.......................A..............A...........G..	110
Mmu	C.CCA.C..T.CA....CGCTT.GG.CACGA.G..A.....G..C.TC..G..G..G........A.....G..C..............A....................	110
Rno	C.CCA.C..T.CA....AGCTT.TG.CACGA.A..A...........T..G..G..G........A........A..............A.....C...........T..	110
Ocu	.C....G.GA.T.TCC.GTCGT..G.CACT.....AT.G..G..T..C...G....A........A..	110
Eca	G.CC.AT.GT....T..G.C.T.GG.CACGA.G..A.....T..............G.................A................................G..	110
Cfa	GC.C..G..A...TCG..TCG..CG.CACG...T.....G.C..............G..............G......................................	110
Bta	..CC.AT.TT.AA.T..GGC.T.GG.CACGA.G..A.....C..G..T........G..........................A....................T..G..	110
Mdo	.....C..G..T.TAG..GGTGGT..T.CG..TG.A.....GA.G..A..A..G.................G...........C...........A..T..G.....G..	110
Tgu	---..GAT	5
Gga	--ATG.C..C......G..GC....G..T..C.G....CG.G.C---------..G.C	47
Hsa	GGCCCAGTGGCGCGAGCGGCCCGATGTGGGCCGCTACCTCCGGGAGTTGAGCGGCTCGGGGCTGGAGCGGCTGCGGCGCGAGCCCGAGCGCCTGGCGGAGGAGCGGGCGC	220
Ptr	....	220
Ppy	....A...................A......................	220
Mac	--	1
Cja	A...................................T...T.	220
Mmu	A..T........T..AA.......C.....................C..........C........C..............A.........T.......A........C.	220
Rno	......A.....T..AAA......C.....................C..........C........C........T.....A..G.......................C.	220
Ocu	............G........G..C...	220
Eca	......C.....T....................T.........................A..	220
Cfa	............G.....C.....C..A...............................A....	220
Bta	............T........T..C.....A.....T...A..........T.....A..A.................G.................C........A....	220
Mdo	..GT.................T..C.....G........G..C...C......................A.....A..G...........A...........A.......	220
Tgu	C..G.C.G.C--	15
Gga	...GGC.G...C..GCG.CG.T..GC.C.C.GCT..TG.GGCC...C..GCG.CGCT........C.GA....G.T..G.....G.C...G.......C........A..	157
Hsa	AGCTGCTGCAGCAGACGCGCGACTTGGCCTTCGCTAACTACAAGACCTTCATCCGCGGCGCCGAGTGCACCGAGCGCATCCACCGCCTGTTTGGCGACGTGGAGGCGTCG	330
Ptr	.............A..	330
Ppy	............................T..T..C...	330
Mac	--	1
Cja	.....T.............T........T.....C....................T...........................T....C.....A...............	330
Mmu	...G...................C....T.....C...........G.....T.....G..........................G..C..C.................C	330
Rno	...................G...C..........C..T........G.....T.....T..G..A..............A.....T..C.....A..............C	330
Ocu	.......................C..........C...................................G.................C.....................	330
Eca	..................................C..T..C..C..................	330
Cfa	.......................C..........C...C..C............T....C	330
Bta	..................................C...C.....G..T.......A....	330
Mdo	...................G..T...........C....................T..T...........G.....T...........C..C..G...........CG.C	330

```
Tgu     -----------------------------------------------------------------------------------G..GGA..C....G.A.C...AGCCG. 42
Gga     G.G.CGG.GCCG.......GCG...........AGC.....CGCG.......T...TC...G..........G....GC.GG....GGC..C..T.G.A.C...AGCCGT 267

Hsa     CTCGGCCGCCTGCTCGACCGTTTGCCCAGCTTCCAGCAGAGCTGCAGGAACTTTGTGAAGGAAGCCGAGGAGATCAGCTCCAACCGCCGGATGAATAGCCTGACCCTAAA 440
Ptr     ....................C......................................................................................... 440
Ppy     ....................C......G....................................................T............................. 440
Mac     ---------------------------------------------.T.......................................T...........T........... 65
Cja     ...A.T..............C............................................T..A..........................C...T....T..... 440
Mmu     .....G...............C.....C.....................................T................G......C.....C.CG.....T..... 440
Rno     .....G.A...............A.............................C...........T...............................C......T..... 440
Ocu     ....................CC.....G.........................CC..C....G............G...G..G...G........C.CG.....T..G.. 440
Eca     ....................C......................................A..G..T..............A..T..A........C.CG.....T..... 440
Cfa     ................G...CC.........................A...........A..G..T.............................C.C......T..... 440
Bta     ....................C...........A..A..............T........A..G..T...........T.....T...........C.C......T..... 440
Mdo     ..G....A......G..T..CC...................T..TC.............A..T..A..A.....TG.T........A.AT.......CT........G.. 440
Tgu     ..G...A.G.....G..G..GC.....GCGC.G...G.CGC....C.C.C...CA..CGC..T.......CC...GC..G..G...G..C.....C........G..G.. 152
Gga     ..G...A.......G..G..CC.....GC.C.G...G.CGC....C.......CA..CGT..T.......CC..TGCT.G..G...G........C........A..G.. 377

Hsa     CCGGCACACAGAAATTTTGGAAATACTGGAGATTCCTCAGCTCATGGACACCTGTGTCCGGAACAGTTATTATGAAGAGGCCCTGGAGCTTGCAGCCTACGTACGCCGAC 550
Ptr     .............................................................................................................. 550
Ppy     ...................................................................................................T.......... 550
Mac     ..................A................................................................................T.......... 550
Cja     ...............CC..........A..............................................................C........T.......... 550
Mmu     ...............CC....G..TT.A.....A..C.....T........G........A.....C...C..........G...........C.....T.....G.... 550
Rno     T........G.....CC....G..TT.A........C.....T.................A.....C..............A.................T.....G.... 550
Ocu     .........G..G..CC....G..T........C....................C..G..C.....C..C..C..G..............C..C.....T..G.....C. 550
Eca     ......T........C........C................................T........C...........................T.......C....... 550
Cfa     ...............CC.......C.................A.....T.................C.......................C..G.....T.......... 550
Bta     ...............CC.......C........C................................C.......................G........T.......... 550
Mdo     ............G..C........CT..............T.A..............T..A..T..C..C.....G.....T..T..A........A..T..G...A... 550
Tgu     .........C..G..CC....G..C........C..G.................C.........G.C..C..C..G.....G........GA..........G.....G. 262
Gga     ............G..CC.C..G..C........C..G....................G..T...C.C..C.....G.....A........G..G........G.....G. 487

Hsa     TGGAGAGGAAATACTCTTCCATCCCTGTCATCCAGGGCATCGTGAACGAAGTGCGCCAGTCCATGCAGCTGATGCTGAGCCAGCTGATCCAGCAACTGAGGACCAACATC 660
Ptr     .............................................................................................................. 660
Ppy     .............................................................................................................. 660
Mac     .............................................................................................................. 285
Cja     ...........................................A..T.......................A..................................G.... 660
Mmu     ....A.....G.....C........C..T...........T.....T.............................A..............A...........T...... 660
Rno     ....A...........C...........T...........T.....T.....T.......................A..............A..G..........G.... 660
Ocu     ..........GC....C........G...................G...G.....G............T.......C........C........G.......G.GG.... 660
Eca     ....A...................................T.................................T...C............................... 660
Cfa     ....A.....G.....C.......................T........................................................A............ 660
Bta     .A..A...........C.......................T..................................................A.................. 660
Mdo     ....A........T...A....T.....T..A........T..T..T.....C..G..A..T......T.......C.....AT.A..T........TC.A.....T..T 660
Tgu     ..........GC..AGCAG......C..T...............C.G..G............GC..............................G...C.C......... 372
Gga     ......A...G...AGCAG......C...........A..T...GCT..G.....G.......CC.............A.G....C........G..CC.C......... 597

Hsa     CAGCTTCCTGCCTGCCTCCGTGTCATTGGCTACCTGCGGCGCATGGACGTCTTCACTGAGGCTGAGTTGAGGGTGAAGTTTCTTCAGGCCCGAGATGCTTGGCTCCGGTC 770
Ptr     ........C..................................................................................................... 770
Ppy     ........CA............................N........T.............................................................. 770
Mac     .....C..C......................................T.............................................................. 395
Cja     .....C..............................T..........T..............C...C.........................G................. 770
Mmu     .....C..A.................A.....T..T...........T.............................A...........T..G..............C.. 770
Rno     .....C..A........G..............T..T..A........T.............................A..............G..C........T..C.. 770
Ocu     .....G..C........G..C.....C.............................C.........C.............C..C...........G..C.......AC.. 770
Eca     .....C.........................T...............T........C..................................................T.. 770
```

```
Cfa     .....C.........................T.......T.......TA.......A..................................................T..  770
Bta     .....C.........................TT..A....AA.....T........C.........C.......C................................T..  770
Mdo     .....C........T.....A..T...........T..T..A......A.T.....A............C.AA.T.....CT.G..............A.....G..T..  770
Tgu     .C...G..C........G..CC....C....T......C...........GC...GC.....C...C..C.C..C.....C..G.....G..G..C..C.....G..C..  482
Gga     .CT..G..CA.T.....G..G.....C.....T..............T........A.....C...C..C.CA.C.....C..G.....G..G.....C.....G..C..  707

Hsa     CATCCTGACTGCCATTCCTAATGATGATCCCTATTTCCATATTACAAAAACCATCGAGGCCTCCCGTGTCCATCTCTTTGATATCATCACCCAGTACCGTGCCATCTTCT  880
Ptr     ..............................................................................................................  880
Ppy     ..........................................C...................G...............................................  880
Mac     ..........................................C...................G...............................................  505
Cja     ..........................................C..G................G...............................................  880
Mmu     ...............C..............T..C.....C..C...........T.....T.G...G...................................T..T....  880
Rno     ...............C..G...........T..C........C.....G..T..T.....T.G...C.......................A...................  880
Ocu     ......C........C...G....C..C..............C..G................G...C........G..C...G.......T........C..........  880
Eca     ............G..C..C.....G..............C..C...................G............A..C...............................  880
Cfa     ...............C..C....................C..C...........T.......G............G..C...........T..............A....  880
Bta     ...............C..C....................C..C...................G.....................T.........................  880
Mdo     ..........T.......C.G...G.....T.....T..C..C.AC..G.....T.....A.GT..A.....C..T..............A..............A....  880
Tgu     .G.G.A.G.CT....C..CG.CC.C..C.....CG.G..CC.C..C..G...........G.G...C..G..C..G..C..CG.GC....G........C..........  592
Gga     .....A.G.CT....C...G.G.....C.....C.....CC.C..C..G..AG.........G...C.....C.................A...................  817

Hsa     CAGACGAGGACCCACTGCTGCCCCCTGCCATGGGTGAGCACACTGTGAATGAGAGTGCCATCTTCCATGGCTGGGTGCTACAGAAGGTCTCACAATTCCTGCAGGTGCTG  990
Ptr     ..............................................................................................................  990
Ppy     .....................................................................................A........G...............  990
Mac     ....T........................G.............C.........................................A........G...............  615
Cja     ................A..........T.....................................T.....C.................A........G.........T.....  990
Mmu     ....T........C........A...........C...T..............G................A........G.....AA.......G..............A  990
Rno     ....T........C........A...........CC..T..............G................A......T.......AA.......G...............  990
Ocu     .G...........G...........C..GGCA..G....G...A...........C...........C...........G...........G..G............T..  990
Eca     ....T........TT...................G............................................G.....A........G...............  990
Cfa     ..............T..T.........T.TCAA.C............G.................T..................GA..G.....................  990
Bta     ....T........T........T..............A....TG...................................G.....A........G...............  990
Mdo     .T..T.........T.......A..A..TGCC.......C.T.A.................A...............T.G...........T..G............T..  990
Tgu     .G........G..G------------------------------C..GCC...G.G...........CA..........G...........CG.G.......GT......  672
Gga     .T..T.....G.....C...G....C.AGCA.------.C.T.CC.C......G.G..............T........G...........TG.G...............  921

Hsa     GAGACCGACCTTTACCGGGGCATAGGCGGCCACCTGGACTCTCTGCTGGGCCAGTGCATGTACTTTGGGCTGTCCTTCAGCCGGGTGGGAGCTGATTTCCGGGGTCAGTT  1100
Ptr     ...............................G..............................................................................  1100
Ppy     ...............................G............A.....................................C.................C.........  1100
Mac     ...............................G.....................................................................C........  725
Cja     ............A..............A...G........................................................................C.....  1100
Mmu     .....T..............T.....A....G.T.............T.............................T...........G...........C..C.....  1100
Rno     ....................T.....T....G...............T..A..........................T...........G...........T..C.....  1100
Ocu     ...G.G......GCG.....GG.C.......G.........C.......................C.......................G..............C.....  1100
Eca     ..A..T......C.......G..........G......................................................A..G..............C.....  1100
Cfa     ............C..A....G.....T..T.GT..A..............T......................................G.T............C.....  1100
Bta     ............A....A..G..T...A...GT...............................................T..A..A..G....................  1100
Mdo     ....GT......C.G..T..TGGG..TA...G.........AT....A..A....................C..............A..G..............C...C.  1100
Tgu     CG.CG......GG.G..C...G.G.......GG........G........G..............C...............A.......C.TG........C..G...C.  782
Gga     ...CGT.....GC.GA.A..AG.G...A...G.T.......A........G..A...........C..C..C.....T...A.......G.....C.....T..G...C.  1031

Hsa     GGCTCCTGTTTTCCAGCGGGTGGCCATCAGCACTTTCCAGAAAGCAATTCAGGAAACAGTGGAGAAATTCCAGGAAGAAATGAACTCCTACATGCTCATCTCGGCTCCAG  1210
Ptr     ..............................................................................................................  1210
Ppy     ......................................................................................................A.......  1210
Mac     ...................A........................................................................CA........A.......  835
Cja     A...........................C......................AC......................T................CT.....G..A.......  1210
Mmu     ................A.......A.....................G..G.A...G...................T.......CT..A....CC.........A..G...  1210
```

```
Rno  ................A.......A....AT...............G..G.A...G.T.................T..G....CT..A....CC........A...G... 1210
Ocu  .........G.............................................G....A..............T........T.......CC..GG...T........ 1210
Eca  ......A....................T...........................G..........G........T................CC......CT........ 1210
Cfa  .....................A..............T.....G...........GG..........G........T........T.......CC........C.....C. 1210
Bta  ..TG.............A......T.............................GG.T..C.....G........T...........T....CC.........A...... 1210
Mdo  T..A.....G.......A...T..TT.G.AT......A........G....A..CGGT........G..T.....T........T.......CT..T.....T.....T. 1210
Tgu  ...G..CC.C........C......GC.GA.G.C...GG..GG..GG.GG....GG.G........G....G...G..G.....T.......C...G...........G. 892
Gga  ...C..CA.C........C..C..TGC..C...C...AGC..G...G.AG....GG.G........G........G..G.....T.......C.........T..C.... 1141

Hsa  CCATCCTGGGCACCAGTAACATGCCTGCTGCTGTGCCAGCCACCCAGCCGGGGACGCTGCAGCCACCCATGGTGCTCCTAGATTTCCCACCCCTCGCCTGCTTTCTCAAC 1320
Ptr  .......................................................................................................C...... 1320
Ppy  .........................................C.............A...............................................C...... 1320
Mac  ..........................A...T..................A.....................................................C...... 945
Cja  ....T.......G.............A................T.....A.....A...........G....................C..............C...... 1320
Mmu  ............G........C......CA.A..A........A..A..A..C..............G.........T....C.....G..T..T.....T..C...... 1320
Rno  ............G...C..............A...........A.....A.................A.........T....C........T...........C...... 1320
Ocu  ............G......GC.......C..C.........G.T.......................G...........G..C........A.....G..T..C...... 1320
Eca  ............G.......C..................T...T.....A..A..C........G...........TT.......T........T..............T 1320
Cfa  ............G.......C..........C.......T...T...........C.....................T....C...........T........C...... 1320
Bta  ..G.........G....GC.C....G........CT...T...T.....A..............G............T....C.....G.....T........C...... 1320
Mdo  .TGCT...C.G---...GCA..T.......T...C..T.TG..TG.A..T..A..CT.......C.......G...G..G.....T.....T..G..T..T..C...... 1317
Tgu  ..G.G.....G---G.C.CTGC.G.G.TGC.G.....C..GG.......T..C..A........C...........G..G..C.....G.....T........C.....T 999
Gga  .TG.G......---...GC.GCAG.G.TGC.A.....GT.AG.......A.....A....................G..G..C..T..G.....T........C.....T 1248

Hsa  AATATTCTGGTTGCCTTCAATGATCTGCGCCTCTGCTGCCCTGTGGCCCTGGCGCAGGATGTGACTGGGGCCTTGGAAGATGCCCTTGCCAAGGTAACTAAAATAATCCT 1430
Ptr  .............................................................................................................. 1430
Ppy  ..CTC.GG.TCGC.T............GC.......................GCAG.C..............................TTC.A................. 1430
Mac  ...........C.................................................................................................. 1055
Cja  ............................................A........C.....C.........T....A........................C.......... 1430
Mmu  ..C...T.......T.........T.A..T.....T...........T..A..A...............A........A........A..........C....C...T.. 1430
Rno  ..C...T.......T.........T.A..T.....T..............A..A........A..............G..C......ATG........C....C...... 1430
Ocu  ..CG.......C............C..................................A.....CC.....C....G..C.......T.C....C..C..G.CG..... 1430
Eca  ...........C....................T....................A..A............A..C....G....................C........... 1430
Cfa  .....................................................A...........GA...T.C....G....................C...G.G..... 1430
Bta  .GC........C..T............................................G.....CA.....C....G..C.......A.............G....... 1430
Mdo  ..C...........T.........T....T..T..T..T........T..A..C.............CTT.T.....G..........G.........A........AT. 1427
Tgu  GGGC.C..T...............C....G..G........CA.C........A.....C..C..C.CCTG.C....C.C......C..TG..AC.-------------- 1095
Gga  GGCC.C..T..C............C....G...........CA.C..G.....C.........G...CCTG.C..A.G..........G......G..C........... 1358

Hsa  GGCCTTCCATCGCGCTGAAGAGGCTGCCTTCAGCAGCGGGGAGCAAGAGCTCTTTGTCCAGTTCTGCACTGTCTTCCTGGAAGACCTTGTTCCGTATTTAAATCGCTGTC 1540
Ptr  .............................................................................................................. 1540
Ppy  ..........................T.........T.................C....................................................... 1540
Mac  ......................A.............A.................CA...................T.................................. 1165
Cja  ...T..T..............A................................C................................CA....A................ 1540
Mmu  ...A........A..C..........TG........T........T...A.....................C.....................T.....G..C....... 1540
Rno  A..A........T..A..........TG........T........T...A.....................C.....................T.....G..C....... 1540
Ocu  .........C.....C..G.....C..............C....GC........C................C.........G.....G.....C..CC.C........C. 1540
Eca  ............T.......................T..............T..C......................................T................ 1540
Cfa  ......T..C..G..A...........T.......C.........G...............................................T................ 1540
Bta  ............T..G....................T........................................................T..C............. 1540
Mdo  ...T..T..C..G..A..G..A..A........T..TCA...A.............................T............T.A.....T..CC.......A.... 1537
Tgu  ----------------------------------------------------------------------------------------------------------GCCA 1099
Gga  .........C..G..A..G.....G..A......G.GC.C.....G.....................G.A.CA...........T..GC....T..CC.G..C.....C. 1468

Hsa  TCCAAGTCCTTTTTCCACCAGCTCAGATAGCACAGACTTTAGGCATTCCTCCCACTCAGCTCTCCAAGTACGGTAACCTAGGGCATGTGAACATCGGCGCCATTCAGGAG 1650
Ptr  ...........................................T.AGAGAATG.AAATT..G------------------------------------------------ 1602
Ppy  ...........................................T------------------------------------------------------------------ 1584
```

```
Mac  ......................................................................T..A.....C..........................A... 1275
Cja  ....G....................................................................A...A.C........................G..... 1650
Mmu  .T...........C.................C...............T.A.....C........T...C....A.....T..A..C.........A.......C.....A 1650
Rno  .T...........C.................C...............T.A.....C............C....A.....T..A........T...A.......C.....A 1650
Ocu  ....G.....C..C..G..G..C...C....G.................A.......................GG....T.....C...G..G..A.......C...... 1650
Eca  ....G................................C......G....A.......................A.....C.....C...G..G..A...T.G.C...... 1650
Cfa  .........................A...........C......G....A...G...................A.....T..A..C......C...C...AG.C...... 1650
Bta  ................T....................C...........A.......................A.....G............G..A...T.G.C...... 1650
Mdo  ..........C..C........C.............T...G...G....A........A..........TT..G.....C..A.....C..T..TCAG.TTG.C...... 1647
Tgu  CA..TT.GG.GC.G..CAGCCAAAG.GAGC.TG.AG.AC.G.C.C.G..A------------------------------------------------------------ 1149
Gga  ....G..T..C.....C..G........C......G.GC.G.....C..C..GG.C.....GCAGCGC.....CC....G..CTGC..TG..G.G.A...TC.CAGA... 1578

Hsa  CCCCTCGCCTTTATCCTGCCAAAGAGAGAGACGCTTTTCACCCTGGATGACCAGGCG 1707
Ptr  --------------------------------------------------------- 1602
Ppy  --------------------------------------------------------- 1584
Mac  ...................................C..................... 1332
Cja  ...................................C..GG................. 1707
Mmu  ..T...........TT....T........A..AG.....TGTT.A.....G....A. 1707
Rno  ..T............T....T.........G.AG.....TGTT.A..........A. 1707
Ocu  ...........C........C..........T.G.C...T.......C.GAA...A. 1707
Eca  ..T........C........C.....G......G.C...TGT..A..C..GG...A. 1707
Cfa  ..T..G.....CC.......G.........CT.G.CC..TGT.....G...A...A. 1707
Bta  ..T..G........T.....C.........C..G.C...TGT.....C---A...A. 1704
Mdo  ..TT.G..T..C..TT.............AG.AG.....CTA........AA...GA 1704
Tgu  ------------------------------------------------------..T 1152
Gga  .TG..G...CC.G........GCACAG...G.AGAGC.GC.G.CC.G...GG.A.A. 1635
```
