# Supplementary material for: Comparative analyses of the Conserved Oligomeric Golgi (COG) complex in vertebrates
Source: BMC Evol Biol. 2010 Jul 15;10:212. doi: 10.1186/1471-2148-10-212 (PMC2927914; doi:10.1186/1471-2148-10-212)
Supplement: Additional file 4 — Primer pairs used in PCR reactions. This table provides the sequences of the primer pairs used to detect the presence of COG transcripts in 20 human tissues. [file 1471-2148-10-212-S4.DOC]

**Additional File 4**

| **Primer** | **Sequence (5'-3')** |
| --- | --- |
| **COG1-2F** | CAAGCCAGACTCCAGAATTG |
| **COG1-2R** | CGATGAAGGTTGCTGTTGAG |
| **COG2-1F*** | GTAGGATGAACCTGCCCAAG |
| **COG2-1R** | ACCCGCTTCCTACAGTCAGA |
| **COG3-1F** | ATCACTGTGCCTTGGTTCG |
| **COG3-1R** | ACACAGTTTCTCCAAAAGCTCA |
| **COG4-1F** | TCCACCGAATGGGTCCTA |
| **COG4-1R** | CAAGCTGACGAACTTTGCTG |
| **COG5-2F** | GCTGATTTTGCACAGATGGA |
| **COG5-2R** | ACTTGCCTGGAAGAGCAGAG |
| **COG6-1F** | CAACATAAACCTGAACAGGGCT |
| **COG6-1R** | TGGGGCTGACAGATAACGAT |
| **COG7-2F** | CGAGTACATCAGCAACATCG |
| **COG7-2R** | GTGCAATGCCAACTCTAAGG |
| **COG8-2F** | GATAGCACAGACTTTAGGCATTC |
| **COG8-2R** | CGTCTCTCTCTTTGGCAGGATA |
| **GAPDH-F** | GAGTCAACGGATTTGGTCGT |
| **GAPDH-R** | CCAGCATCGCCCCACTTGA |

*Primer pair for COG2 was obtained from qPrimerDepot (<http://primerdepot.nci.nih.gov/>; Cui W, Taub DD, Gardner K. [qPrimerDepot: a primer database for quantitative real time PCR.](http://www.ncbi.nlm.nih.gov/pubmed/17068075?ordinalpos=1&itool=EntrezSystem2.PEntrez.Pubmed.Pubmed_ResultsPanel.Pubmed_DefaultReportPanel.Pubmed_RVDocSum) *Nucleic Acids Res*. 2007, 35(Database issue):D805-809).
