# Supplementary figures and images for: Comparative analyses of the Conserved Oligomeric Golgi (COG) complex in vertebrates
Source: BMC Evol Biol. 2010 Jul 15;10:212. doi: 10.1186/1471-2148-10-212 (PMC2927914; doi:10.1186/1471-2148-10-212)

COG1

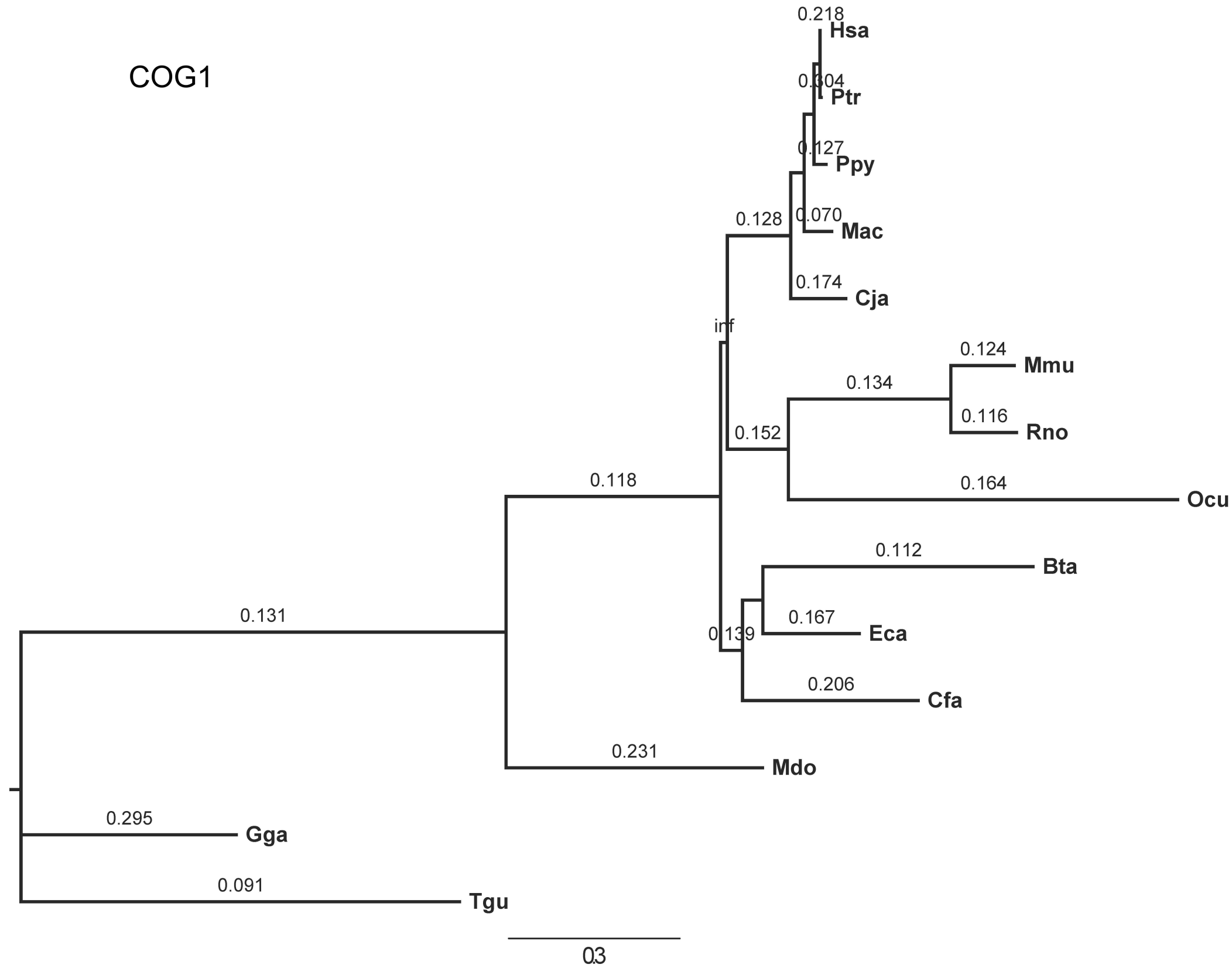

COG2

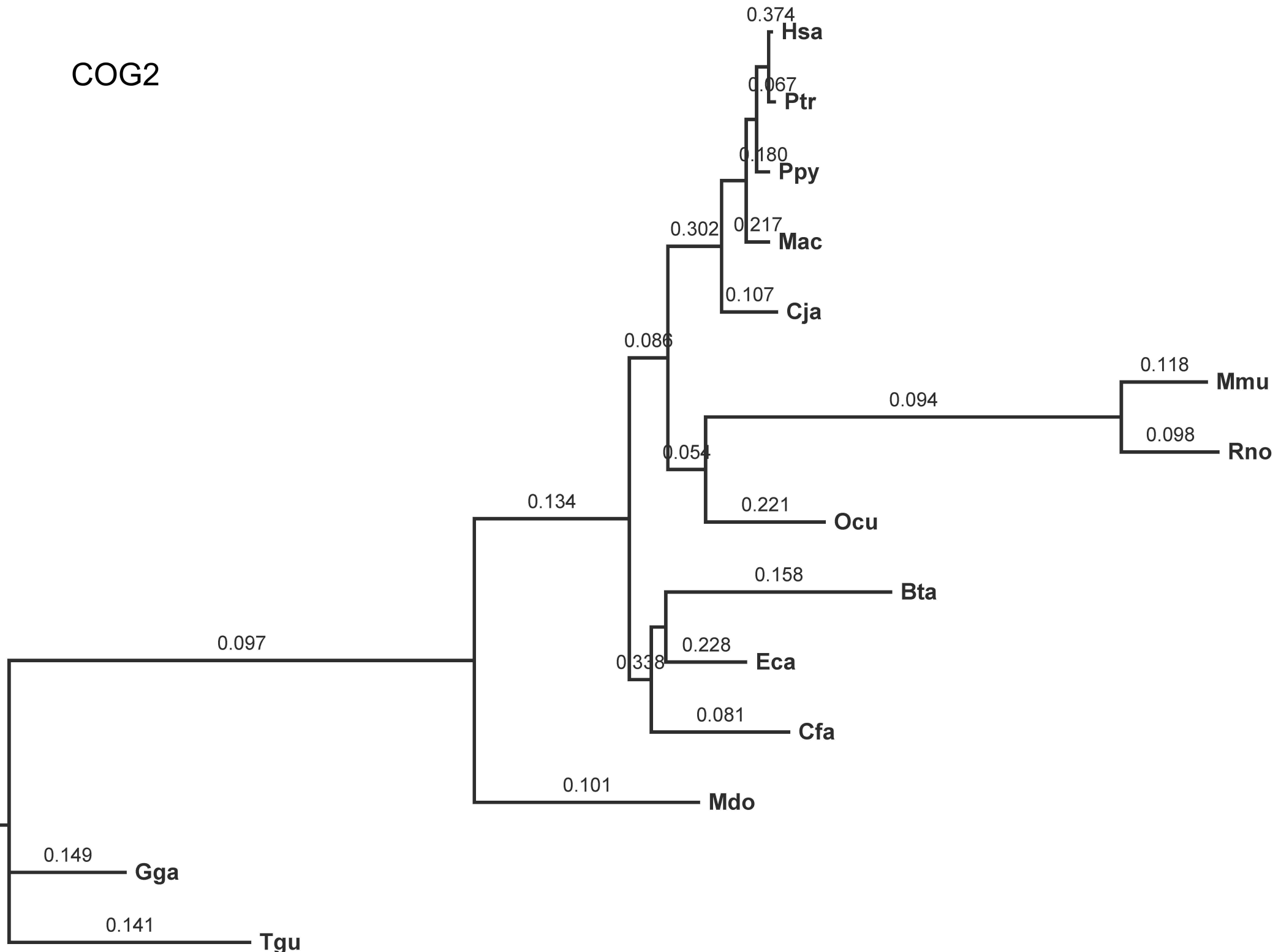

COG3

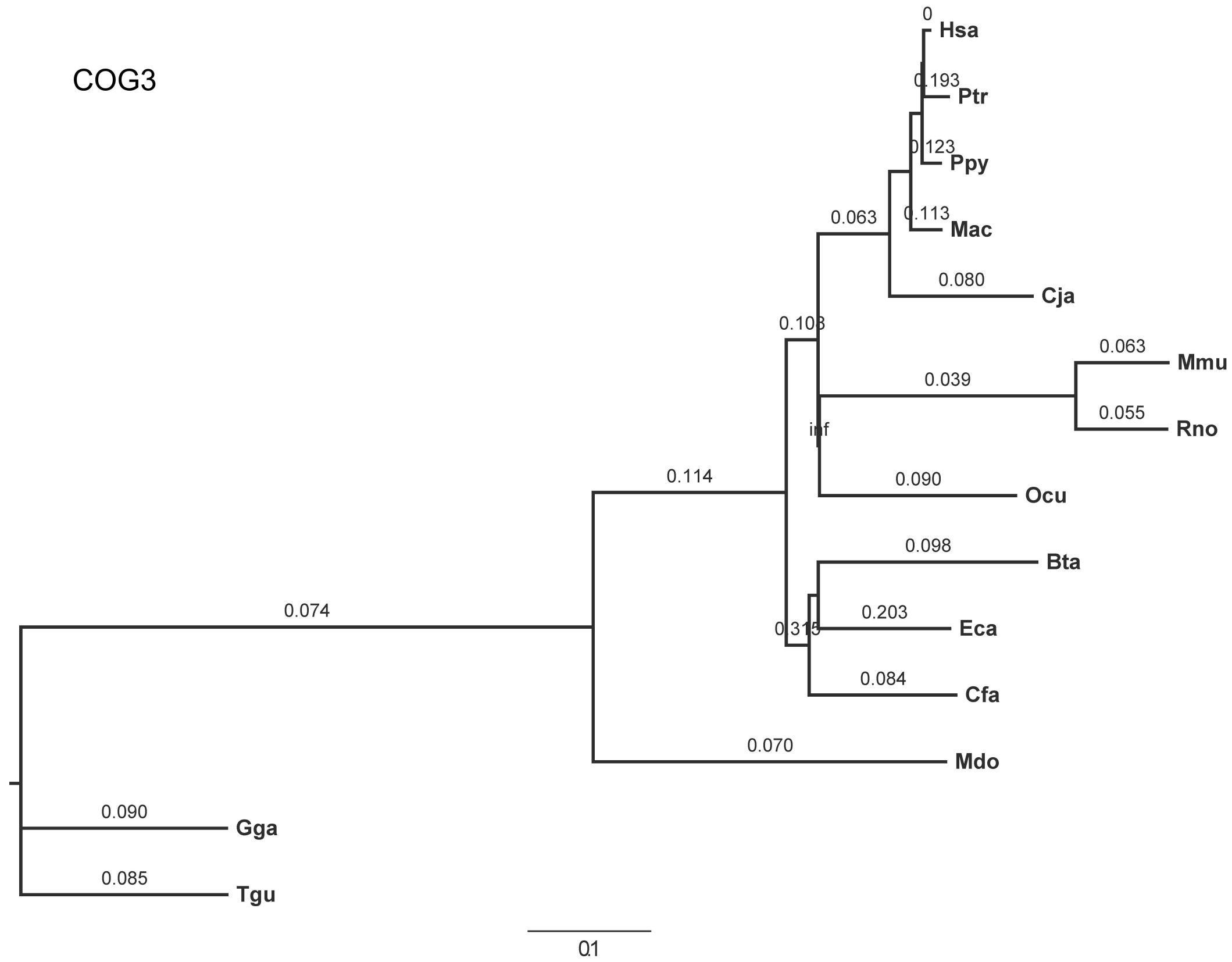

COG4

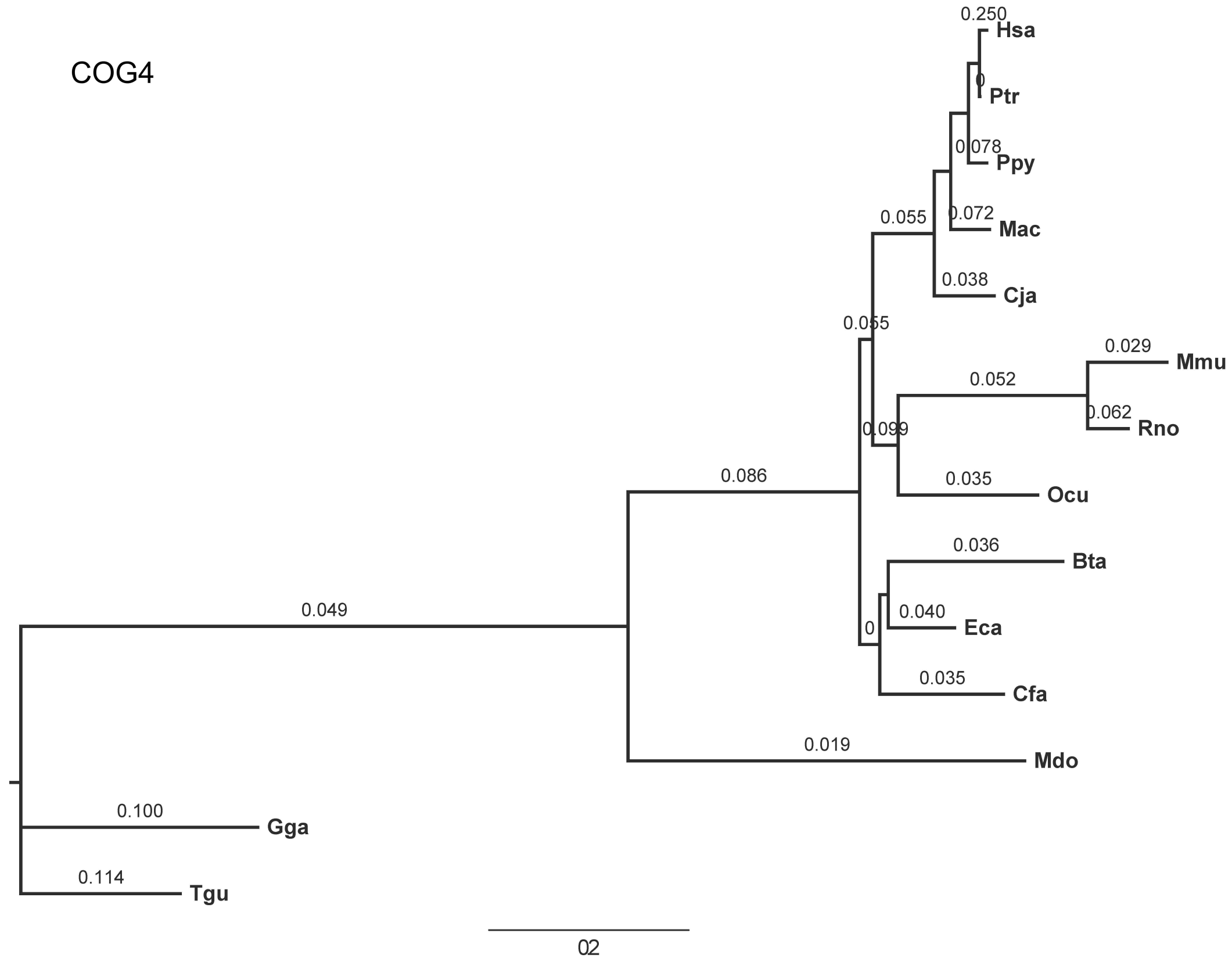

COG5

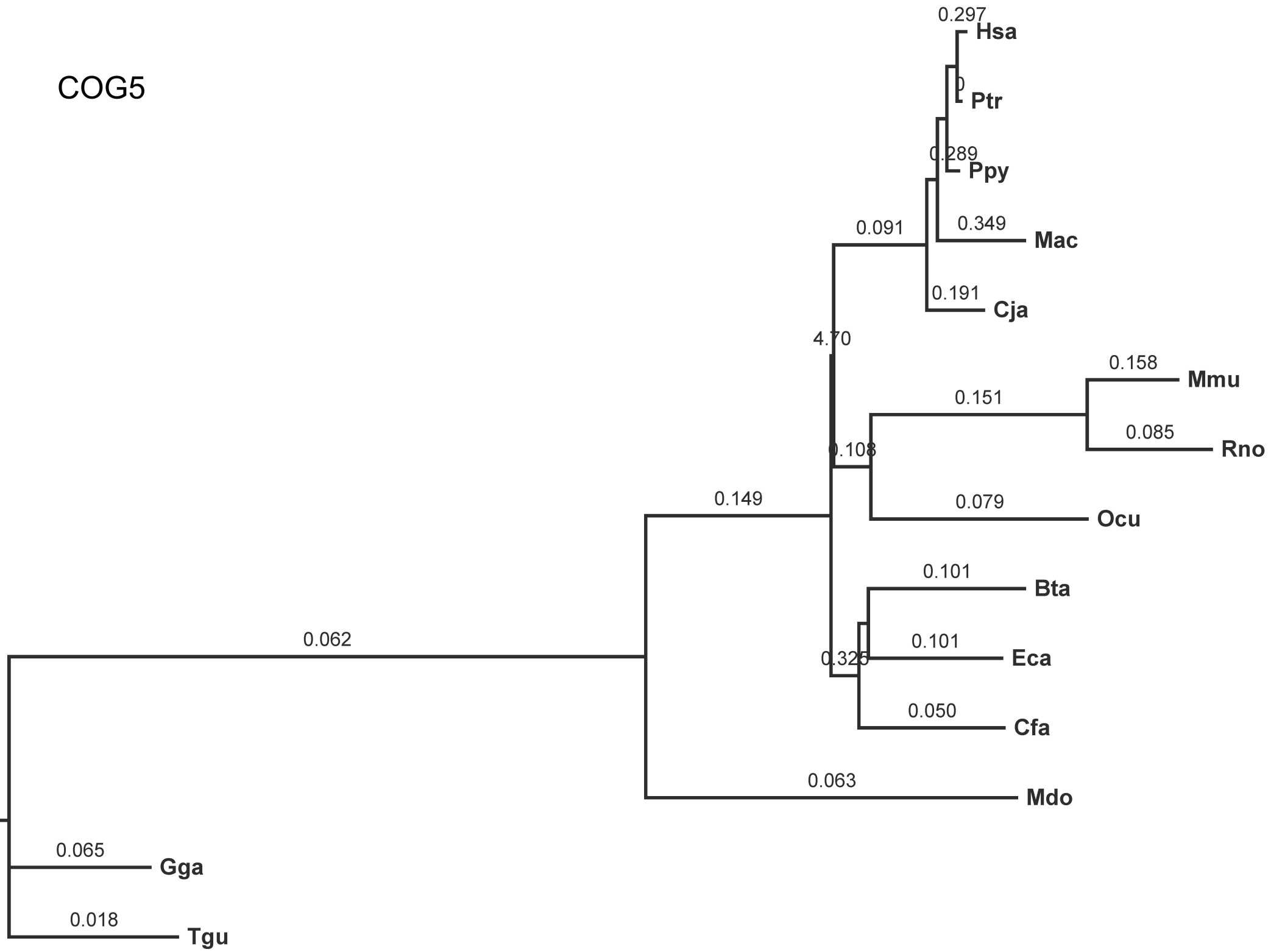

COG6

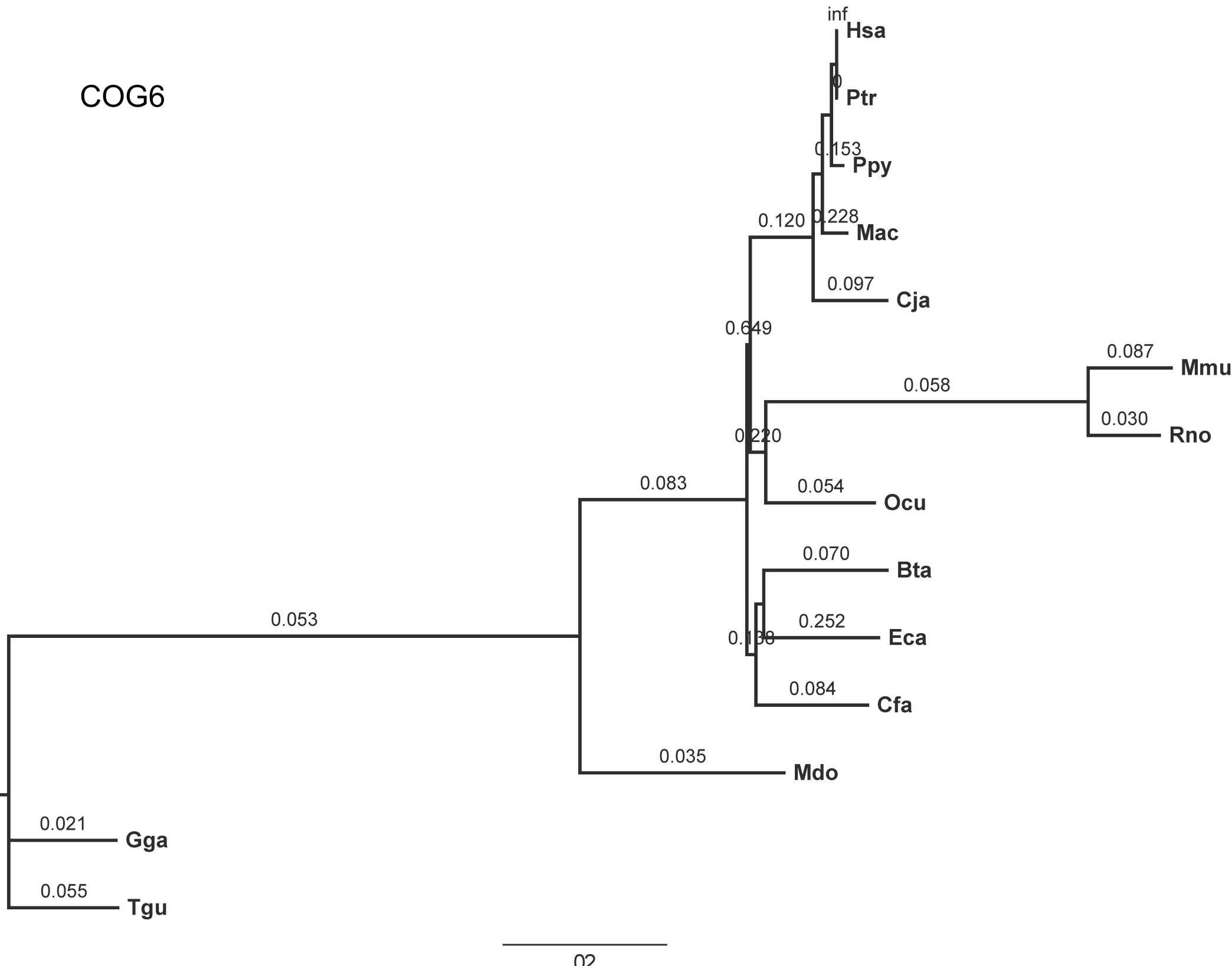

COG7

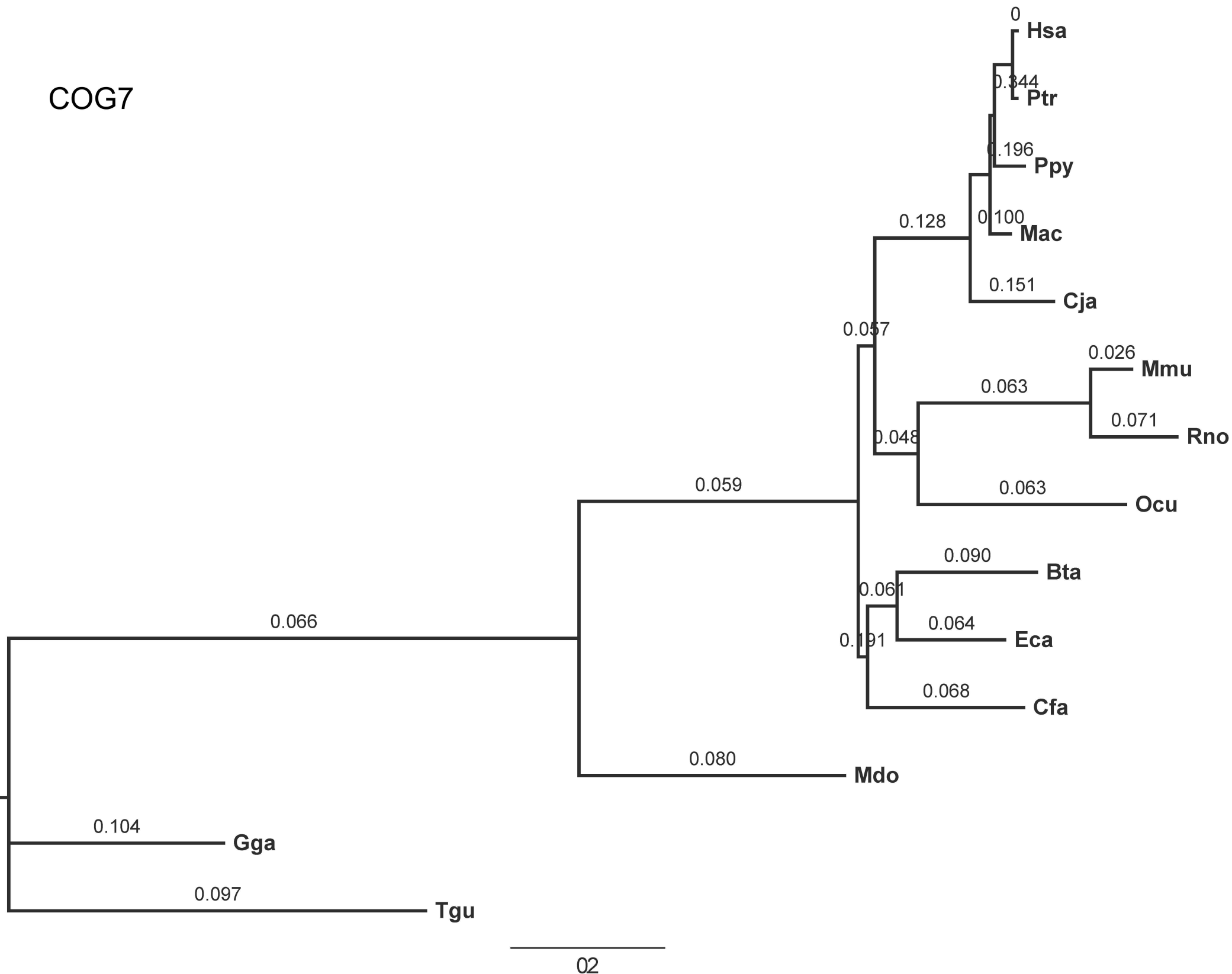

COG8

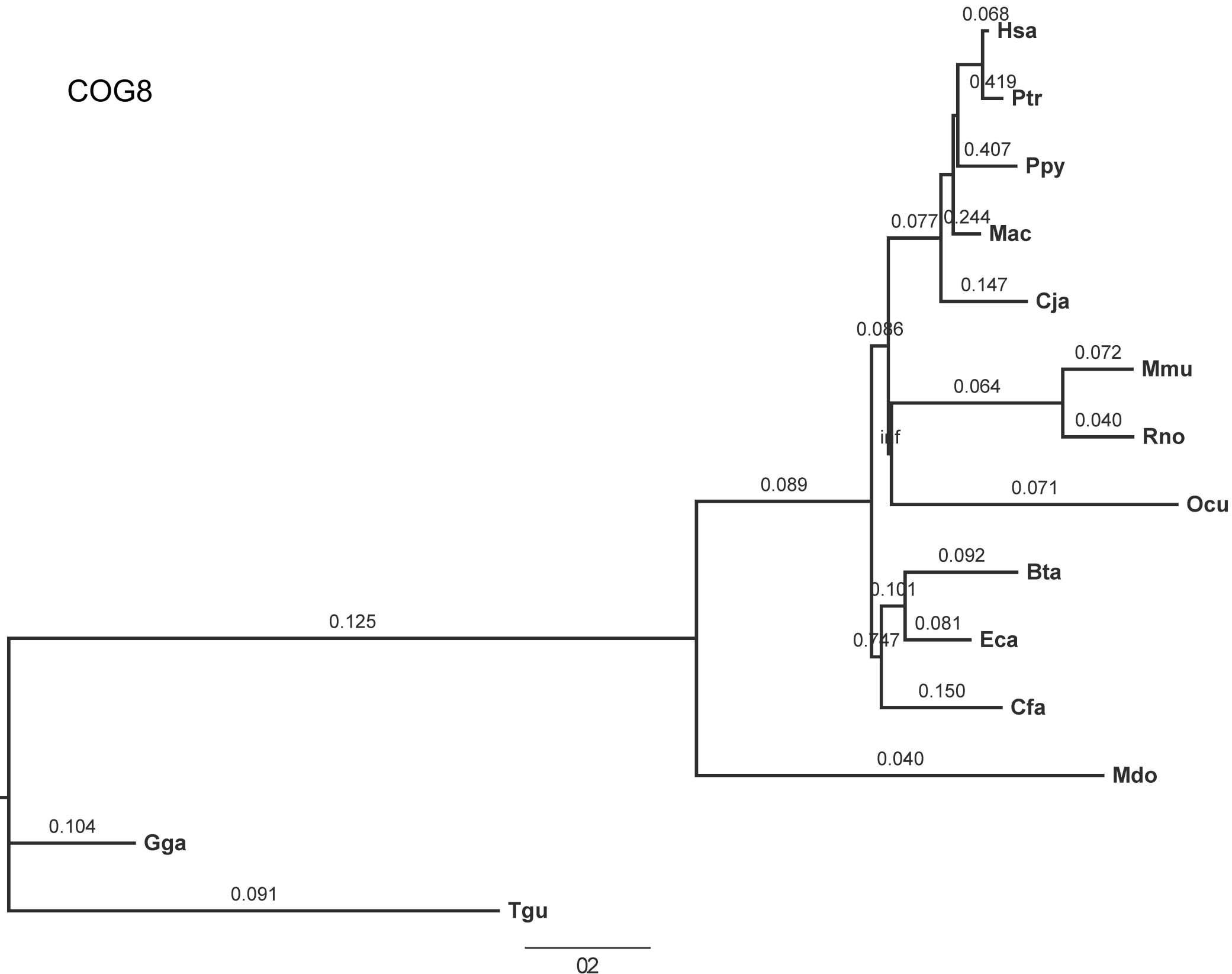

Supplement: Additional file 5 — Phylogenetic trees with branches drawn in proportion to their lengths, defined as the expected number of nucleotide substitutions per codon, for each COG gene. Values along each branch represent ω ratios (for simplicity of the figure some of them were not indicated) estimated under the free-ratio model. In COG5 phylogeny the branch leading to Euarchontoglires (primates and Glires) presents a ω ratio of 4.70 (dN = 0.0011; dS = 0.002). A LRT comparing the one-ratio model with the two-ratio model (ωbackground; ωEuarchontoglires), revealed that the estimated ω ratio for the Euarchontoglires branch (infinite, indicating the absence of synonymous substitutions) was not significantly higher than the background ratio (0.094). Moreover, the LRT comparing the two-ratio model with and without the constraint ωEuarchontoglires ≤ 1 revealed that this ratio was not significantly higher than 1 as well. [file 1471-2148-10-212-S5.PDF]

COG2 – dN tree

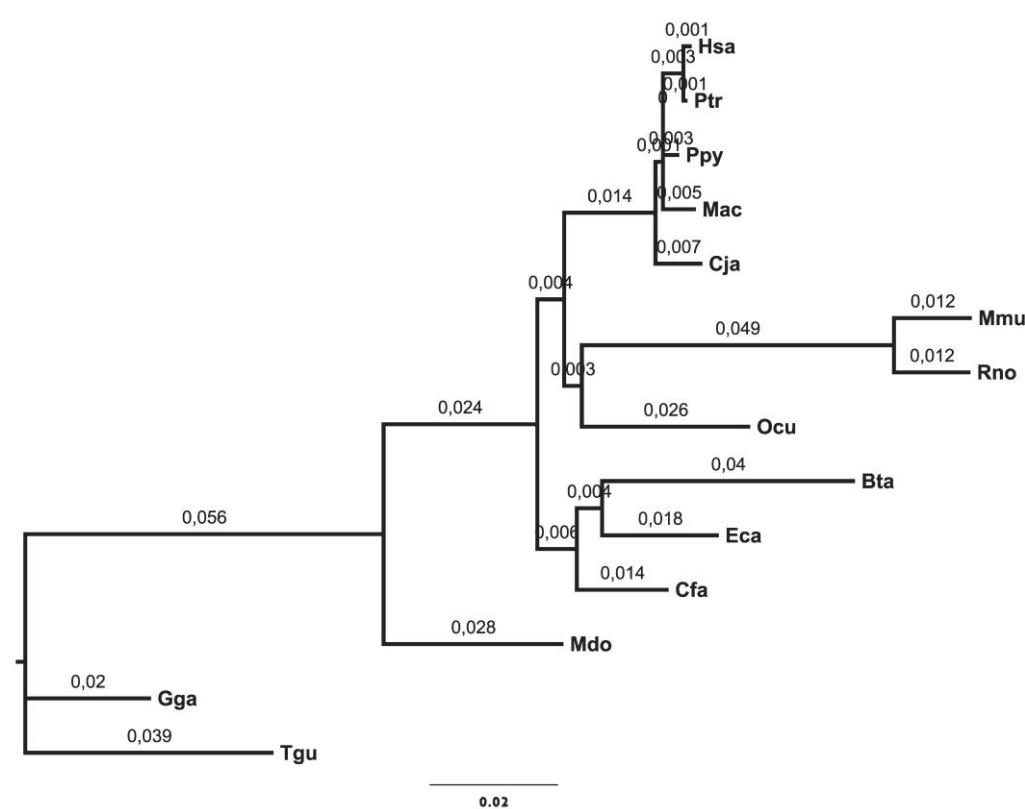

COG2 – dS tree

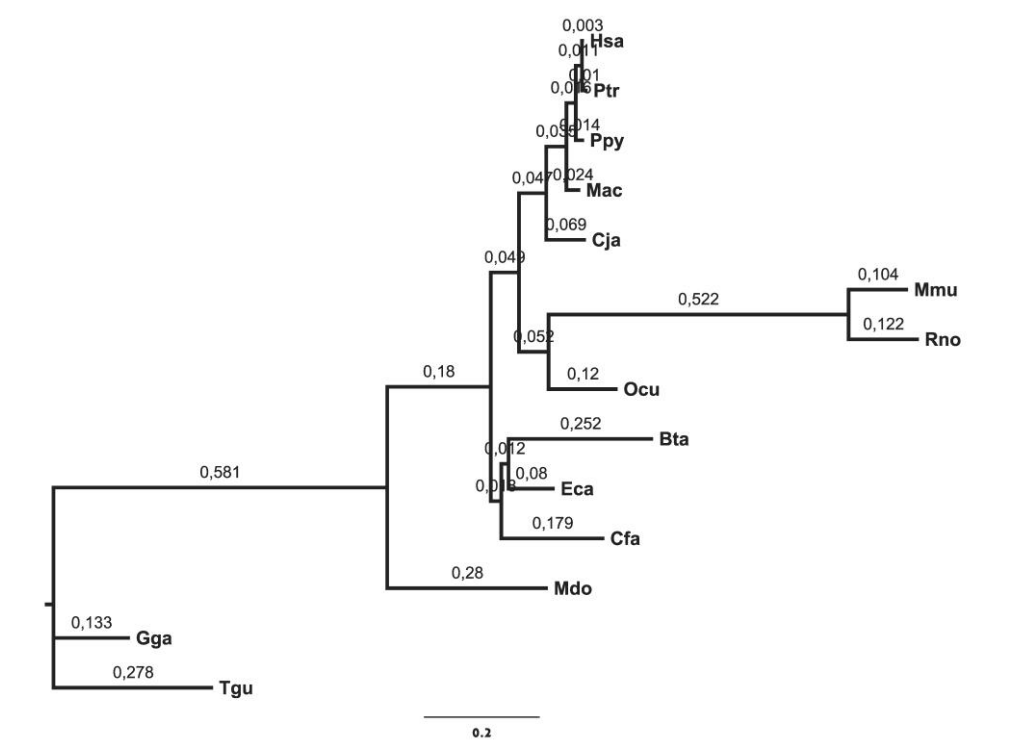

COG6 – dN tree

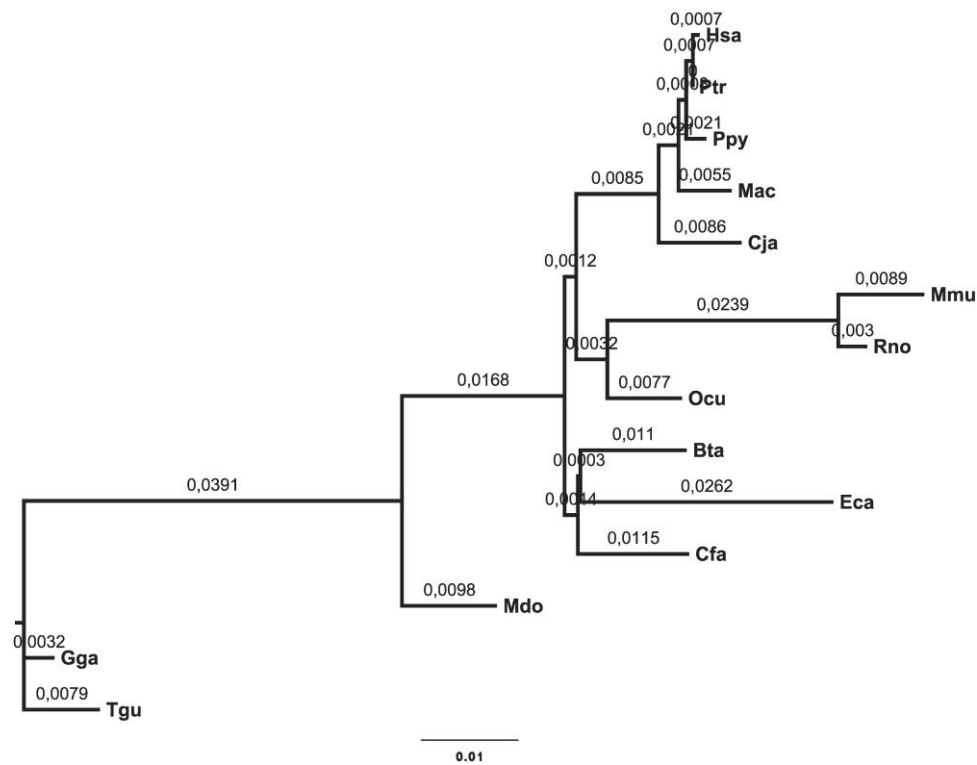

COG6 – dS tree

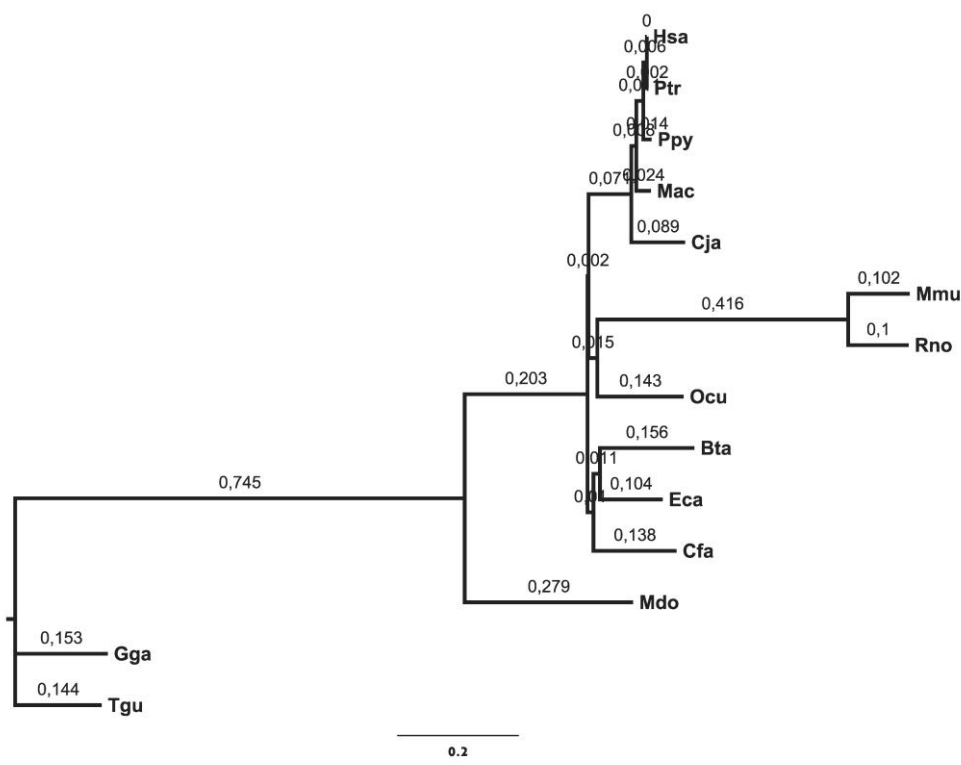

Supplement: Additional file 6 — COG2 and COG6 genes phylogeny with branch lengths defined as the estimated nonsynonymous substitutions rate (dN) or the estimated synonymous substitutions rate (dS). [file 1471-2148-10-212-S6.PDF]
